# Worldwide impact of disease attributable to low physical activity for diabetes and kidney diseases

**DOI:** 10.3389/fendo.2025.1499381

**Published:** 2025-05-05

**Authors:** Ping Ding, Lingyan Qi, Jinglei Wang, Min Tang, Yujie Weng

**Affiliations:** ^1^ Health Management Center, Ningbo Rehabilitation Hospital, Ningbo, Zhejiang, China; ^2^ Gynecology Department, Ningbo Rehabilitation Hospital, Ningbo, Zhejiang, China; ^3^ Traditional Chinese Medicine Department, Ningbo Rehabilitation Hospital, Ningbo, Zhejiang, China; ^4^ Neurorehabilitation Department, Ningbo Rehabilitation Hospital, Ningbo, Zhejiang, China; ^5^ Department of Gastroenterology, The Third Hospital of Haishu District, Ningbo, Zhejiang, China

**Keywords:** low physical activity (LPA), global burden of disease (GBD), annual percentage change (EAPC), disability-adjusted life year (DALY), diabetes and kidney diseases

## Abstract

**Background:**

We aimed to examine patterns of diabetes and kidney disease due to Low physical activity (LPA) in 204 countries between 1990 and 2021.

**Methods:**

Data were from the Global Burden of Disease (GBD) 2021. Age-standardized death rate (ASDR) and age standardized disability-adjusted life years (DALYs) were estimated. In addition, annual percentage changes (EAPC) were calculated.

**Results:**

The age-standardized DALY rate (EAPC = 0.89; 95% CI: 0.85, 0.93) and age-standardized death rate (ASDR) of diabetes and kidney disease due to LPA increased (EAPC = 0.34; 95% CI: 0.28,0.39) during 1990 to 2021. Among them, the age-standardized DALY rate and ASDR increased fastest in Eastern Europe (EAPC 2.43 and 3.79, respectively). Mauritius had the most significant increase in age-standardized DALY (EAPC = 3.40), while the Russian Federation had the most significant increase in ASDR (EAPC = 4.40). In 2021, both ASDR and age-standardized DALY rates per 100,000 people increased in women compared with men. The age-standardized DALY rate per 100,000 people generally showed an upward trend, with the highest in Oceania (285.33) and the highest ASDR in Southern Sub-Saharan Africa (10.56).

**Conclusions:**

The global ASDR and age-standardized DALY rates reveal a significant surge in diabetes and kidney disease attributable to LPA from 1990 to 2021. This escalation is evident across 21 regions, with notable increases observed in Eastern Europe, Sub-Saharan Africa, and Oceania.

## 1 Introduction

Low physical activity (LPA) is a prevalent risk factor linked to numerous major non-communicable diseases, such as diabetes and kidney disease (1). LPA and extended sitting durations are linked to metabolic impairments, including diabetes and renal damage, in diabetic patients (2). Research has demonstrated that engaging in physical activity elicits a beneficial effect on inflammation, blood pressure, lipid levels, metabolism, and cardiovascular health, particularly in patients suffering from kidney disease. In addition, it is noteworthy that diabetes serves as the predominant cause of kidney disease (3). This suggests that LPA-induced diabetes may exacerbate the progression of nephropathy. Grouping diabetes and nephropathy, when caused by LPA, under a single category would facilitate a comprehensive evaluation of the enduring impact of diabetes on renal health, as well as the influence of these two diseases individually and collectively. LPA places a substantial economic strain worldwide. The projected global cost of LPA to public healthcare systems from 2020 to 2030 is approximately US$ 300 billion (equivalent to roughly US$ 27 billion annually) if there is no reduction in LPA levels. The annual economic impact of Type 2 diabetes, attributable to physical inactivity, is estimated to be between US$1.9 and US$13.2 billion. It is evident that LPA contributes not only to the global economic burden but also adds to the disease burden by facilitating diabetes and kidney disease. The interplay between LPA-induced diabetes and kidney disease further exacerbates the global disease burden. This suggests that the rise in LPA-induced diabetes and kidney disease, along with their interconnectedness, could potentially precipitate a significant health crisis in the 21st century. An assessment is imperative to comprehend the current state of this disease burden.

LPA is widely regarded as a ignificant modifiable risk factor for numerous diseases (4). Physical activity is crucial for amplifying the effects of renal inflammation, oxidative stress, vascular function, immune response, and macromolecule metabolism (5). Presently, there is a paucity of epidemiological data exploring the relationship between LPA and diabetes, as well as kidney disease, on a global scale. In light of this, it is imperative to conduct an assessment of LPA-induced diabetes and renal disease to contribute to and fill the existing gaps in the literature.

This study leverages data from the global burden of disease to scrutinize trends in the burden of diabetes and kidney disease as attributed to LPA across 204 countries, spanning the period from 1990 to 2021. The findings are tailored to offer policymakers scientifically-grounded insights for optimal resource allocation and policy execution, achieved through a comparative analysis and exploration of potential causative factors.

## 2 Methods

### 2.1 Data sources

Data for the study came from the Global Burden of Disease 2021 (GBD) report, which provides the latest and most comprehensive estimates of the global burden of 371 diseases and 88 associated risk factors between 1990 and 2021. This information was obtained through the Global Health Data Exchange GBD Results Tool (https://vizhub.healthdata.org/gbd-results/). The study focused on diabetes and kidney disease and analyzed LPA, age-standardized death rate (ASDR) and age-standardized Disability-Adjusted Life years (DALY) in 204 countries around the world. Age categories range from 25 to 94 years in 5-year increments, with additional classifications for those 95 and older. This study is based on a publicly available database and does not require ethical approval.

According to the International Classification of Diseases, 10th Revision, diabetes and kidney disease are caused by Diabetes mellitus (type 1 diabetes, type 2 diabetes) and chronic kidney disease (chronic kidney disease caused by type 1 diabetes, chronic kidney disease caused by type 2 diabetes), represents chronic kidney disease caused by diabetes and hypertension). Chronic kidney disease, chronic kidney disease caused by glomerulonephritis), chronic kidney disease of unknown etiology (Acute glomerulonephritis), ICD-10 classification is D63.1, E10-E11.9, I12-I13.9, N00-N08.8, N15.0, N18-N18.9, P70.2. Q61-Q62.8.

### 2.2 Attributable risk factors

According to the standardized iteration instructions of the 2021 GBD (Global Physical Activity Questionnaire and International Physical Activity Questionnaire), physical activity intensity is quantified in Metabolic Equivalents (METs) and is used to assess the intensity, duration and frequency of activity in adults. Metabolic equivalents are a widely accepted measure of energy expenditure. The metabolic equivalent is equivalent to 1 kcal/kg/hour and represents the energy expenditure during sitting. Activity levels were divided into light activity levels (600-3999 meter minutes per week) and moderate activity levels (4000-7999 meter minutes per week). Furthermore, LPA is defined as 3,000 MET-min/week (6).

### 2.3 Statistical analysis

To assess the impact of kidney- and diabetes-related LPA at global, regional, and country levels, we used linear regression models to examine trends in ASDR and age-standardized DALY rates from 1990 to 2021. The model expresses the natural logarithm of the age-standardized rate (ASR) as Ln (ASR) = α + βX + ϵ, where X is the calendar year. This method helps calculate the annual percentage change (EAPC) and 95% confidence interval (CI) to evaluate the ASR trend over a specified time period. Depending on whether the EAPC and its 95% CI are greater than 0, less than 0, or contain 0, determine whether the trajectory of the ASR trend is increasing, decreasing, or stable (18, 19). The techniques used in this study have been described in detail in previous studies (7, 8). The DisMod-MR2.1 model and Bayesian regression technique were employed for data analysis in this study. These analytical methods have been frequently used in past GBD studies (9). The integration of DisMod-MR 2.1 modelling with Bayesian regression techniques offers a robust analytical framework for GBD research. DisMod-MR 2.1 is employed to produce consistent estimates of disease burden, while Bayesian regression techniques are utilized to further scrutinize temporal trends, spatial distributions, and uncertainty, thereby providing a more scientifically rigorous foundation for public health decision-making. R software (version 4.1.3) was used for statistical analysis.

## 3 Results

### 3.1 Global level

**Figure 1:**
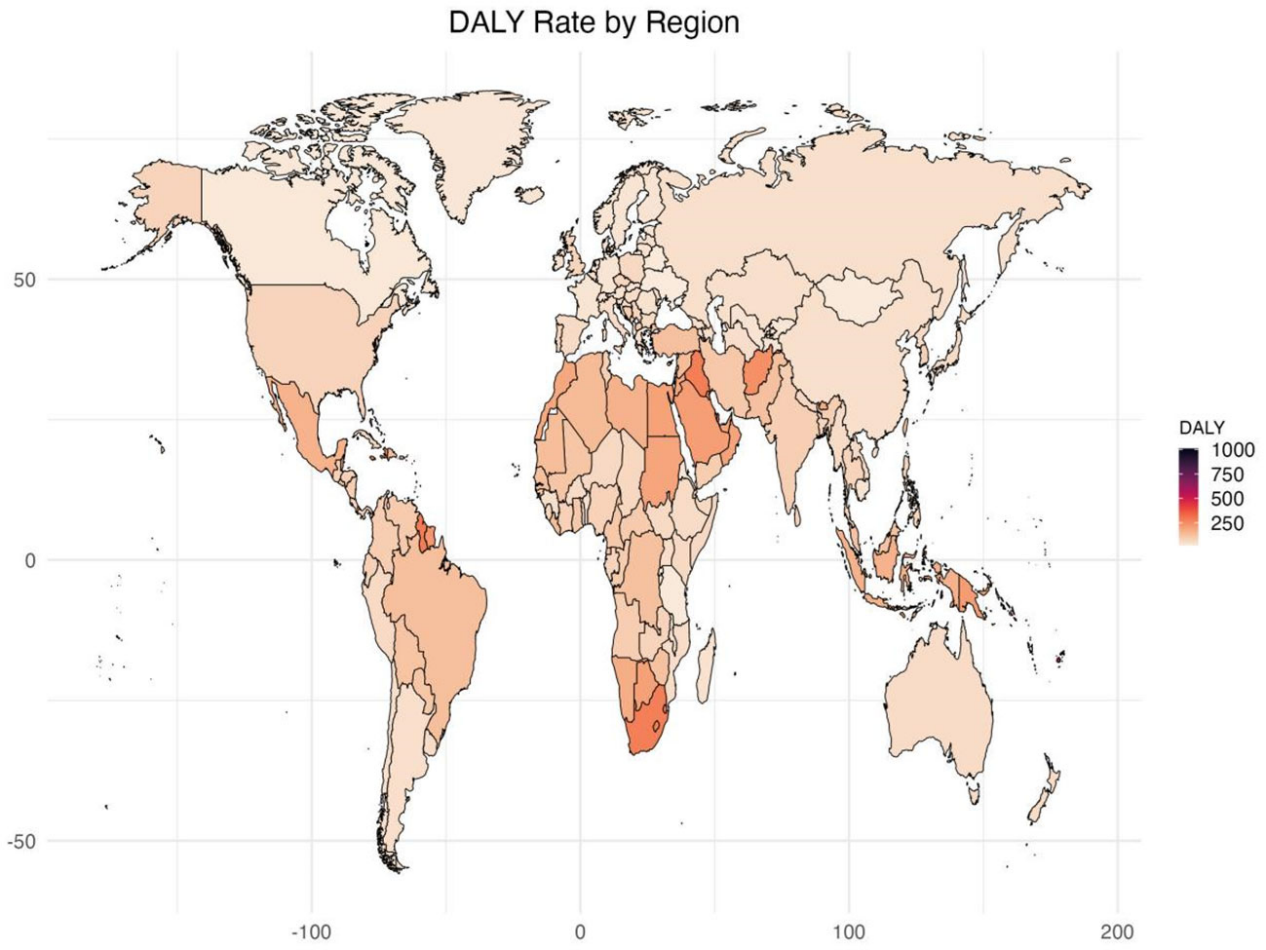
Age standardized rates of global burden of diabetes and kidney diseases due to Low physical activity in 2021, by locations. **(A)** Age-standardized DALY rate **(B)** ASDR.

**Figure 2:**
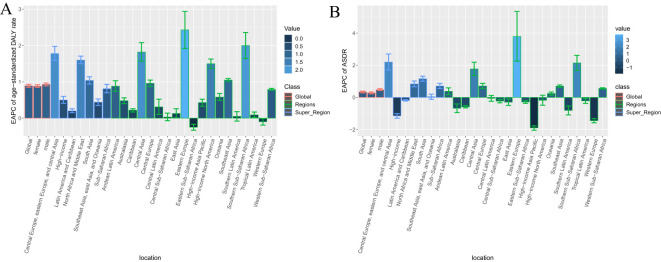
EAPC of age standardized rates of diabetes and kidney diseases due to Low physical activity from 1990-2021, by locations. **(A)** Age-standardized DALY rate **(B)** ASDR.

Globally, age-standardized DALY rates per 100,000 people for diabetes and nephropathy due to LPA in 2021 (75.08, 95% UI: 32.54, 115.76) and ASDR (2.30, 95% UI: 1.00,3.54) were the same as 1990 data (DALY rate: 55.70, 95% UI: 24.37, 84.15; ASDR: 2.06, 95% UI: 0.90,3.11) (**Table 1**, **Figure 1**, **Supplementary Figure S1**). The interval from 1990 to 2021 experienced an escalation in age-standardized DALY rate (EAPC = 0.89; 95% CI: 0.85,0.93; **Table 1**, **Figure 2A**) and ASDR (EAPC =0.34; 95% CI: 0.28,0.39; **Table 1**, **Figure 2B**) for diabetes and kidney diseases linked to LPA, signifying an upward trajectory in the global burden of these afflictions.

**Table 1:** The age-standardized death rate (ASDR) and age-standardized DALY rate diabetes and kidney diseases due to Low physical activity in 1990 and 2021and its temporal trends.

	ASDR			Age-standardized DALY rate (per 100000) No.95%UI		
location	1990 No. (95%UI)	2021 No. (95%UI)	1990-2021 EAPC No. (95%UI)	1990 No. (95%UI)	2021 No. (95%UI)	1990-2021 EAPC No. (95%UI)
Global						
both	69571.59 (30454.72,104756.41)	190132.24 (83163.12,291751.76)	3.30 (3.26,3.34)	55.70 (24.37,84.15)	75.08 (32.54,115.76)	0.89 (0.85,0.93)
female	45138.83 (20469.07,68662.31)	118032.62 (51334.74,182566.35)	3.14 (3.10,3.19)	63.45 (27.33,96.01)	85.34 (36.89,132.97)	0.88 (0.85,0.92)
male	24432.76 (10497.47,36441.58)	72099.62 (31828.39,111685.07)	3.57 (3.53,3.61)	46.49 (20.44,72.05)	63.68 (27.23,99.18)	0.93 (0.89,0.97)
Super_Region						
Central Europe, eastern Europe, and central Asia	2926.48 (1227.03,4442.33)	8853.65 (3822.21,13643.94)	3.53 (2.96,4.11)	26.04 (11.11,40.29)	45.92 (19.58,72.11)	1.78 (1.59,1.98)
High-income	21628.77 (9175.14,33220.04)	34875.22 (14445.88,55998.84)	1.48 (1.36,1.61)	47.27 (19.98,71.36)	57.58 (24.33,90.84)	0.50 (0.41,0.60)
Latin America and Caribbean	8204.48 (3500.54,12338.37)	24288.15 (10575.10,37400.73)	3.63 (3.57,3.69)	114.98 (49.46,175.54)	124.65 (53.79,194.62)	0.20 (0.15,0.26)
North Africa and Middle East	5095.37 (2200.83,7749.04)	16780.54 (7274.82,25926.92)	4.29 (4.11,4.47)	103.21 (45.57,162.43)	159.48 (68.33,246.15)	1.60 (1.50,1.71)
South Asia	10926.00 (4641.76,16551.98)	42922.64 (18744.35,66849.41)	4.61 (4.46,4.76)	64.72 (27.86,100.11)	90.90 (40.32,142.61)	1.04 (0.94,1.14)
Southeast Asia, east Asia, and Oceania	15417.24 (6590.18,23668.64)	47936.08 (20856.62,73392.54)	3.60 (3.51,3.70)	50.37 (21.14,78.25)	60.69 (25.96,94.04)	0.44 (0.35,0.53)
Sub-Saharan Africa	5373.26 (2160.27,8286.44)	14475.95 (6005.01,22180.24)	3.25 (3.16,3.35)	76.19 (32.10,117.51)	97.92 (39.97,149.83)	0.81 (0.70,0.93)
Regions						
Andean Latin America	421.69 (173.58,677.51)	1533.63 (678.26,2446.55)	4.37 (4.15,4.59)	56.16 (23.25,90.73)	75.75 (33.27,119.12)	0.88 (0.74,1.03)
Australasia	383.93 (160.40,598.97)	891.12 (376.49,1396.01)	2.56 (2.26,2.86)	45.74 (19.81,73.08)	54.64 (23.29,85.55)	0.48 (0.40,0.56)
Caribbean	1280.81 (542.55,1956.62)	2447.33 (1039.02,3809.89)	2.07 (2.00,2.14)	142.62 (60.47,217.76)	154.73 (67.50,245.11)	0.22 (0.17,0.26)
Central Asia	246.97 (104.95,385.13)	742.27 (312.17,1191.01)	3.28 (2.94,3.62)	23.05 (10.01,36.41)	42.13 (17.75,66.16)	1.82 (1.57,2.08)
Central Europe	1875.81 (800.30,2923.10)	3763.88 (1622.25,5799.20)	2.64 (2.41,2.86)	45.35 (19.24,70.77)	58.67 (25.42,92.67)	0.96 (0.87,1.05)
Central Latin America	2957.55 (1197.21,4583.12)	9858.32 (4246.84,15426.15)	3.99 (3.81,4.17)	110.94 (44.70,170.78)	126.76 (52.73,202.28)	0.31 (0.10,0.52)
Central Sub-Saharan Africa	769.92 (315.22,1245.63)	1819.31 (762.36,3092.36)	2.60 (2.43,2.77)	110.91 (44.52,181.39)	116.29 (47.85,191.74)	0.03 (-0.07,0.14)
East Asia	9450.85 (4010.82,14953.32)	27815.81 (12290.78,45037.46)	3.42 (3.29,3.56)	42.93 (18.06,67.17)	47.47 (19.88,74.07)	0.13 (0.01,0.26)
Eastern Europe	803.70 (334.54,1227.45)	4347.50 (1787.47,6825.04)	4.72 (3.13,6.33)	16.08 (6.67,25.05)	37.83 (15.51,59.20)	2.43 (1.92,2.94)
Eastern Sub-Saharan Africa	1312.08 (532.86,2064.25)	2745.27 (1076.35,4471.75)	2.23 (2.06,2.40)	50.52 (20.10,80.07)	49.65 (19.53,80.99)	-0.25 (-0.33,-0.17)
High-income Asia Pacific	2644.08 (1146.47,4168.88)	5198.35 (2159.60,8840.22)	2.26 (2.12,2.41)	53.06 (22.80,82.23)	62.83 (26.18,100.56)	0.43 (0.33,0.52)
High-income North America	5612.33 (2379.02,8941.70)	11706.67 (4873.57,18614.73)	1.93 (1.64,2.23)	42.46 (17.21,67.60)	70.90 (29.39,112.93)	1.50 (1.37,1.63)
North Africa and Middle East	5095.37 (2200.83,7749.04)	16780.54 (7274.82,25926.92)	4.29 (4.11,4.47)	103.21 (45.57,162.43)	159.48 (68.33,246.15)	1.60 (1.50,1.71)
Oceania	216.19 (88.69,355.51)	610.04 (256.58,1001.82)	3.38 (3.33,3.43)	234.01 (97.61,377.56)	285.33 (119.11,457.70)	0.58 (0.49,0.68)
South Asia	10926.00 (4641.76,16551.98)	42922.64 (18744.35,66849.41)	4.61 (4.46,4.76)	64.72 (27.86,100.11)	90.90 (40.32,142.61)	1.04 (0.94,1.14)
Southeast Asia	5750.20 (2380.80,9008.72)	19510.24 (8558.61,29903.39)	3.89 (3.82,3.96)	73.98 (32.02,115.33)	105.11 (44.70,162.16)	1.05 (1.02,1.09)
Southern Latin America	858.01 (355.36,1373.18)	1387.15 (559.99,2267.43)	1.69 (1.36,2.01)	46.63 (19.48,74.98)	47.80 (19.30,78.14)	0.05 (-0.08,0.18)
Southern Sub-Saharan Africa	1374.08 (594.99,2121.83)	5013.76 (2093.23,7552.12)	4.41 (3.94,4.88)	146.88 (63.48,225.28)	255.66 (109.27,386.16)	2.00 (1.64,2.36)
Tropical Latin America	3544.42 (1563.13,5343.81)	10448.88 (4256.25,16445.70)	3.67 (3.49,3.85)	124.54 (54.00,192.32)	127.52 (53.58,200.43)	0.09 (0.02,0.17)
Western Europe	12130.41 (5235.88,18546.88)	15691.94 (6654.55,24721.37)	0.95 (0.86,1.04)	47.34 (20.36,72.47)	47.61 (21.02,75.14)	-0.10 (-0.19, -0.01)
Western Sub-Saharan Africa	1917.17 (759.06,3017.63)	4897.60 (2006.32,7678.15)	3.05 (3.00,3.09)	66.07 (26.56,103.05)	85.05 (33.86,132.22)	0.79 (0.77,0.82)

### 3.2 Regional level

In 2021, Among the 21 geographical regions worldwide, Oceania had the highest age-standardized DALY rate for diabetes and kidney disease due to LPA per 100,000 people (285.33), followed by Sub-Saharan Africa (255.66) and North Africa and the Middle East and East (159.48). The lowest age-standardized DALYs were in Eastern Europe (37.83), Central Asia (42.13), and East Asia (47.47). From 1990 to 2021, the most significant increase in age-standardized DALY rate was in Eastern Europe (EAPC=2.43), followed by Sub-Saharan Africa (EAPC=2.00) and Central Asia (EAPC=1.82). In contrast, age-standardized DALY rates only showed a downward trend in Eastern Sub-Saharan Africa (EAPC=-0.25) and Western Europe (EAPC=-0.10) (**Table 1**, **Supplementary Table S5**, **Figures 1**, **2A**, **Supplementary Figure S1**).

In 2021, the regions with the highest ASDR of LPA-related diabetes and kidney disease per 100,000 people were Southern Sub-Saharan Africa (10.56), Oceania (9.41), and Central Sub-Saharan Africa (4.93). In comparison, High-income Asia Pacific (0.80), Central Asia (1.04) and Eastern Europe (1.18) are the regions with the lowest ASDR per 100,000 population. In particular, Eastern Europe, Southern Sub-Saharan Africa and Central Asia had the largest increases in ASDR, with EAPCs of 3.79, 2.14 and 1.77, respectively (see **Table 1**, **Supplementary Table S5**, **Figure 2B**, **Supplementary Figures S1, S2**).

### 3.3 National level

Among the 204 countries surveyed, Marshall Islands (1010.40), Federated States of Micronesia (703.44) and Kiribati (694.76) age-standardized disability-adjusted lives for diabetes and kidney disease due to LPA per 100,000 people in 2021 Year. In comparison, the countries with the lowest obesity rates are Ukraine (17.83), the United Republic of Tanzania (20.20) and Belarus (20.58) from 1990 to 2021, Mauritius (EAPC=3.40), Lesotho (EAPC=3.31) and Libya (EAPC=3.02) had the largest increases in age-standardized DALYs, while Cyprus (EAPC=-2.54) Ethiopia (EAPC=-1.67) and Rwanda (EAPC=-1.55) had the largest decreases in age-standardized DALYs (**Supplementary Tables S1, S6**, **Figure 1**).

In 2021, the countries with the highest ASDR for diabetes and kidney disease due to particulate air pollution per 100,000 people were the Marshall Islands (30.46), Fiji (25.07) and Kiribati (24.18). Conversely, the countries with the lowest ASDR are Ukraine (0.18), Belarus (0.36) and Mongolia (0.48). From 1990 to 2021, the largest increase in ASDR was in the Russian Federation (EAPC=4.40), followed by Mauritius (EAPC=4.02) and Georgia (EAPC=3.58), while the largest ASDR decrease was in Singapore (EAPC=-3.89), followed by Cyprus (EAPC=-3.40) and Switzerland (EAPC=-2.69) (**Supplementary Table S2, S6**, **Supplementary Figure S2**).

### 3.4 Trends in gender and age distribution

Gender and age differences exist in LPA-related diabetes and kidney disease. In 2021, age-standardized DALY rates (85.34, 95% UI:36.89,132.97) and ASDR (2.52, 95% UI:1.10,3.90) for LPA-related diabetes and kidney disease were more pronounced in female compared with male group. In contrast, male had lower age-standardized DALY rates (63.68,95% UI:27.23,99.18)) and ASDR 2.03, 95% UI: 0.89,3.16). ASDR and age-standardized DALY rates for diabetes and kidney disease due to LPA increased in both sexes from 1990 to 2021, with the increase more pronounced in male than in female (ASDR male EAPC =0.48; female EAPC =0.28) (age-standardized DALY rate male EAPC = 0.93; female EAPC = 0.88) (**Table 1**).

**Figure 3:**
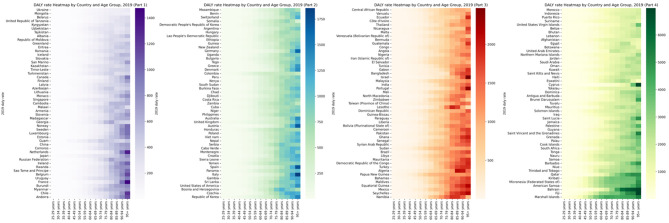
Global burden of DALY rate of diabetes and kidney diseases due to Low physical activity in 204 countries in 2021, by countries and ages.

**Figure 4:**
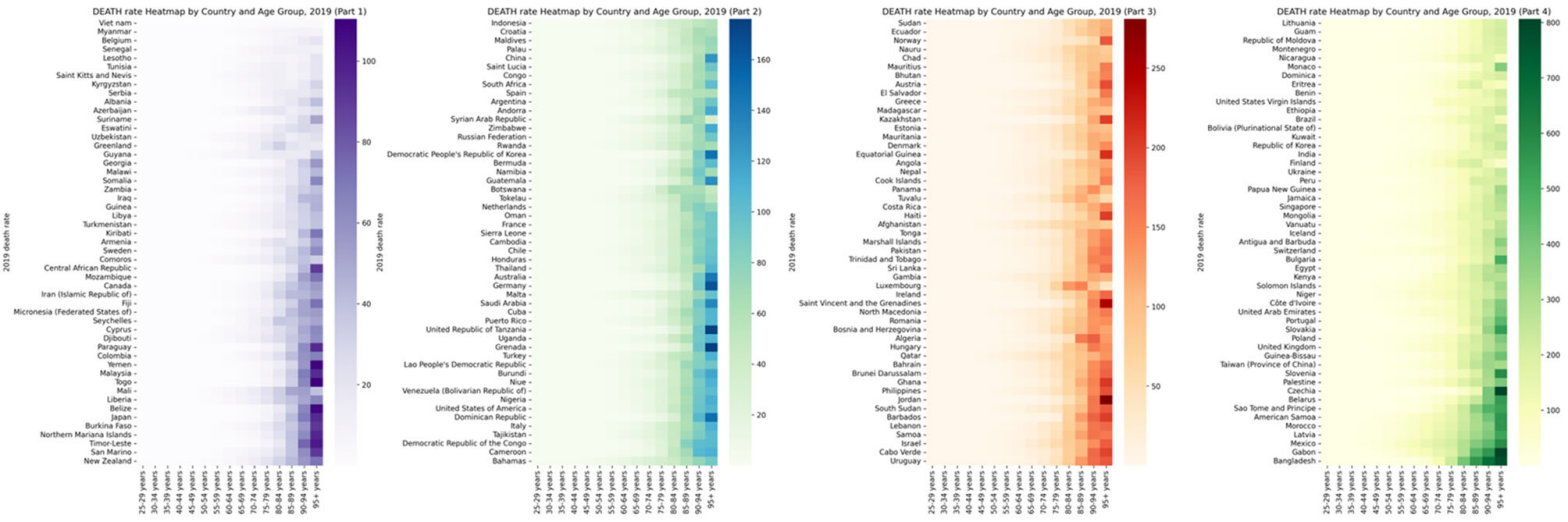
Global burden of Death rate of diabetes and kidney diseases due to Low physical activity in 204 countries in 2021, by countries and ages.

In 2021, the annual disability compensation rate and LPA mortality rate due to diabetes and kidney disease increased significantly in different regions and age groups. Across 204 countries around the world, those aged 90 and over have the highest mortality rates and the highest DALY rates of all age groups over 90. **Figures 3**, **4** and **Supplementary Tables S3 and S4** depict these observations.

### 3.5 Trends in the distribution of super region burdens and trends in sociodemographic index levels in 204 countries

In 2021, the age-standardized DALY rate of diabetes and kidney disease due to LPA per 100,000 people in the seven Super Regions peaked in North Africa and the Middle East, where the age-standardized DALY rate was 159.48. The highest ASDR also is in North Africa and the Middle East, with an ASDR of 4.50. In contrast, Central and Eastern Europe and Central Asia had the lowest age-standardized DALY rates and ASDR, with an age-standardized DALY rate of 45.92 and an ASDR of 1.32 (**Table 1**, **Supplementary Figure S3**).

From 1990 to 2021, the age-standardized DALY rates for diabetes and nephropathy attributable to LPA rose most pronouncedly in Central and Eastern Europe and Central Asia (EAPC=1.78) and least prominently in Latin America and the Caribbean (EAPC=0.20). The ASDR exhibited its greatest increase in Central and Eastern Europe and Central Asia (EAPC = 2.2), while the minimal rise was observed in high-income regions (EAPC = -1.15) (**Table 1**, **Supplementary Figure S4**). Among 204 countries analyzed, the EAPC of DALY for the global burden of diabetes and kidney diseases related to low physical activity in 2021 and the corresponding sociodemographic index revealed the slowest EAPC of DALY in nations such as Romania, Spain, Cook Islands, and Belarus, coupled with approximate sociodemographic index values of 0.75. Prior to the sociodemographic index reaching a value of 0.75, the EAPC of ASDR displayed a decreasing trajectory. Conversely, post-index values around 0.75, the trend in age-standardized DALY rates shifted upwards, with countries like the United Republic of Tanzania, Gambia, and the United Arab Emirates reporting notably higher DALY rates than anticipated. Notably, the correlations between the EAPC of ASDR and the sociodemographic index mirrored these trends (**Supplementary Figures S5, S6**).

## 4 Discussion

The study highlights that the global burden of diabetes and kidney disease caused by LPA has increased from 1990 to 2021, with 21 regions showing a sustained upward trend. Incidence is higher in women compared with men, with the fastest growing disease burden in Eastern Europe, the highest ASDR in Southern Sub-Saharan Africa, and the highest Age-Standardized DALY rates in Oceania. The burden of diabetes and kidney disease has been steadily increasing, particularly in Sub-Saharan Africa, Oceania, North Africa, and the Middle East (10, 11). This increase may be related to population aging and LPA-related lifestyle changes. Current projections from the International Diabetes Federation indicate that the prevalence of diabetes is increasing (12). A large number of observational studies have shown that Metabolic risk factors such as diabetes, hypertension, and obesity are the main causes of chronic kidney disease and end-stage renal disease (13). Increases in these metabolic risk factors may explain the significant increases in diabetes-related mortality and kidney disease-related DALYs.

Previous studies have uncovered the mechanisms connecting LPA to diabetes and kidney disease. There are several potential pathways that could explain the relationship between LPA and diabetes. Diabetes mellitus is primarily divided into type 1 and type 2. Type 1 diabetes mellitus, which accounts for 5-10% of cases, is induced by cell-mediated autoimmune defects that lead to the destruction of pancreatic β-cells, resulting in a deficiency in insulin production. On the other hand, type 2 diabetes mellitus, which constitutes 90-95% of cases, is predominantly due to the gradual decline in insulin secretion capacity and is typically accompanied by insulin resistance (14). A sedentary lifestyle, leading to obesity, and the subsequent lack of physical activity are significant risk factors for insulin resistance. This condition expedites beta cell dysfunction and apoptosis, which, in turn, initiate autoimmune responses and beta cell loss in genetically predisposed individuals (15). The pro-inflammatory state, characteristic of obesity, is known to induce insulin resistance in skeletal muscles and the liver, subsequently disrupting their normal metabolic functions (16). Myokines, including irisin, interleukin-6 (IL-6), and interleukin-15 (IL-15), have been demonstrated to be secreted by skeletal muscle in response to exercise. These myokines exert beneficial physiological and metabolic effects on distant tissues such as white adipose tissue, bone, liver, and immune system cells. These effects collectively drive a whole-body anti-inflammatory and insulin-sensitized state, optimizing overall energy expenditure (17). There exist several potential mechanisms underlying the association of LPA with kidney disease. The same physiological processes through which LPA instigates diabetes are operative in kidney disease. Regular physical activity has been shown to improve blood pressure, and it is widely acknowledged that hypertension is a significant contributor to kidney injury and the progression of CKD (18). LPA has the potential to induce insulin resistance, which in turn negatively impacts the glomerular filtration rate, ultimately leading to nephropathy (19). Insulin resistance is believed to be a key factor in the pathogenesis of metabolic syndrome (MS), a condition marked by a range of metabolic abnormalities such as obesity, hyperglycaemia, hypertension, and dyslipidaemia (19). The metabolic syndrome contributes to nephropathy through a variety of mechanisms. These include haemodynamic factors such as hypertension and glomerular hyperfiltration, but also extend to the chronic inflammatory milieu, insulin resistance, and adipokine dysregulation. Moreover, abdominal obesity, which increases intra-abdominal pressure, and the accumulation of free fatty acids and triglycerides in glomerular and tubulointerstitial cells, can contribute to both acute and chronic functional and structural renal injury (20).

The burden of diabetes and kidney disease has been steadily increasing, particularly in Sub-Saharan Africa, Oceania, North Africa, and the Middle East. The Global Burden of Disease 2021 emphasizes that due to poverty and harsh environment in Southern Sub-Saharan Africa and Oceania, the incidence of obesity, hypertension, diabetes, and cardiovascular diseases has increased significantly in the region (21). People with diabetes may develop serious complications such as cardiovascular disease, kidney disease, blindness, and amputation. Environmental toxins and groundwater with high fluoride content further increase the burden of kidney disease (22). Inadequate access to renal replacement therapy can lead to end-stage renal disease and premature death. In Eastern Europe, a shift in economic growth has precipitated an evolution in dietary habits towards high-fat and high-sugar diets. Concurrently, there is an increase in sedentary lifestyles and various psychosocial factors that have a significant impact on the rates of obesity and related morbidities. In Oceania, obesity is a key factor in the rising prevalence of diabetes (23–25), and economic inequality and limited health resources in low-income countries complicate screening and treatment (26, 27). Countries in Sub-Saharan Africa are challenged by shortages of healthcare infrastructure and nephrologists, highlighting the need to strengthen healthcare systems and infrastructure.

Consistent with previous research (28), the impact of physical inactivity in diabetes and kidney disease increased significantly over time in both men and women. Although the overall disease burden is higher in women than men, this increase is more pronounced in men. Possible explanations for this difference include physiological differences between the sexes, the influence of hormone levels and fat distribution patterns (29). Women generally consume more sugar than men, and a high -glycemic index diet can lead to increased abdominal fat, especially in sedentary women. Men tend to do more manual labor or participate in recreational sports, while women may have fewer opportunities to participate in these activities due to family and caregiving responsibilities. Interaction of physical inactivity and increased body mass index contributes to gender trends in disease burden. Mortality and DALY rates related to diabetes and kidney disease increase sharply with age, particularly among those over 90 years of age, which may be attributed to the relationship between physical activity and aging factors gap interaction (30). As older adults age, they increasingly rely on physical activity to maintain blood glucose homeostasis. This highlights the importance of staying physically active, especially for older adults and women who are more health conscious.

In our research, we observed a substantial increase in the burden of LPA-related diabetes and kidney disease in several regions, including Oceania, Southern Sub-Saharan Africa, North Africa and the Middle East, and Eastern Europe. Prior studies have demonstrated that in low-sociodemographic index countries, populations with lower incomes and education levels exhibit higher LPA rates (31, 32). Numerous factors influence physical activity levels, encompassing environmental conditions, support systems, and educational backgrounds (33–35). Notably, the influence of socioeconomic development on these factors is evident, with research indicating that older adults who engage in higher levels of physical activity have a reduced risk of mortality (36). In countries with a high sociodemographic index, factors related to diet may also exert an influence on the prevalence of diabetes and kidney disease, in addition to physical activity (37). Considering the extensive benefits of physical activity, it is imperative for mass media to bolster and enhance support systems for physical activity within communities and workplaces, particularly in low-income countries. Additionally, there is a need for intensified efforts to advocate for physical activity and healthier lifestyles.

We propose several recommendations to address the current state of disease burden. For high-income countries, there is a pressing need for greater understanding of the disease burden associated with diabetes and nephropathy due to LPA. This would facilitate informed decision-making on whether to allocate additional health resources in this area. Moreover, tailored recommendations regarding the types and intensities of physical activity suitable for different populations are required. For low-income countries, there is an urgent need to enhance disease communication. A nuanced understanding of effective health information dissemination channels within each country can lead to successful public health campaigns that increase awareness of LPA as a contributor to diabetes and kidney disease, thereby fostering exercise habits and reducing disease burden. It is imperative that policies be implemented to guarantee research into the appropriate forms of exercise for key demographics such as the elderly and women. Specifically, aerobic, balance, and flexibility exercises may offer feasible options for these groups.

This study offers critical baseline data concerning the global impact of LPA-related diabetes and kidney disease spanning 1990 to 2021. These findings carry significant weight in guiding resource distribution and the execution of intervention strategies. However, there are limitations that warrant consideration. Firstly, updates to GBD data are infrequent, with the most recent information only becoming available in 2021—this potentially fails to reflect current disease trends. Secondly, when evaluating the disease burden linked to LPA, it proves challenging to entirely rule out the potential interactions with other risk factors. Thirdly, while the statistical methodologies employed in GBD studies aid global comparisons between regions, they may contain certain limitations and biases related to data accessibility and processing. Diagnostic precision and confirmation bias at the point of disease registration present potential limitations in estimating cancer incidence and DALYs within GBD. For example, there can be variations in ICD coding practices documenting the underlying cause of death. Research assessing the concordance between death certificates and patient records has identified discrepancies in the reported underlying cause of death (38). These findings, while varying according to location and time, imply that the recording of diabetes as a cause of death may be either over-coded or under-coded, contingent on the specific locale. Fourth, there is a paucity of large-scale, high-quality, population-based studies on disease in certain countries or regions. Consequently, raw data from these areas may invariably contain biased information. It is therefore imperative to exercise caution when interpreting the results of studies, particularly when extrapolating data to countries or regions that are not part of the World Health Organisation and those with underdeveloped health systems. Fifth, the time frame of this study encompassed the COVID-19 pandemic. The pandemic could have potentially impacted data collection, as preventive and control measures such as home confinement might have led to sedentary behaviour and low physical activity (LPA). This in turn could have exacerbated the burden of disease to a certain extent. Therefore, prudence should be exercised when interpreting these results.

Our results show that age-standardized mortality and age-standardized DALY rates increased significantly in 21 regions and showed a continuous upward trend. Of particular concern is the notable escalation in burden, particularly in regions such as Eastern Europe, Sub-Saharan Africa, and Oceania. Our findings highlight the urgent need for targeted health initiatives and increased attention to high-risk groups such as women and the elderly.

## 5 Conclusion

The global ASDR and age-standardized DALY rates reveal a significant surge in diabetes and kidney disease attributable to LPA from 1990 to 2021. This escalation is evident across 21 regions, with notable increases observed in Eastern Europe, Sub-Saharan Africa, and Oceania.

## Data Availability

Existing datasets are available in a publicly accessible repository: Publicly available datasets were analyzed in this study. This data can be found here: the Global Health Data Exchange GBD Results Tool (https://vizhub.healthdata.org/gbd-results/).
