# Supplementary material for: Worldwide impact of disease attributable to low physical activity for diabetes and kidney diseases
Source: Front Endocrinol (Lausanne). 2025 May 5;16:1499381. doi: 10.3389/fendo.2025.1499381 (PMC12086069; doi:10.3389/fendo.2025.1499381)

**Supplementary Figure 1: Age standardized rates of global burden of diabetes and kidney diseases due to Low physical activity in 2021, by locations. (A) Age-standardized DALY rate (B) ASDR.**


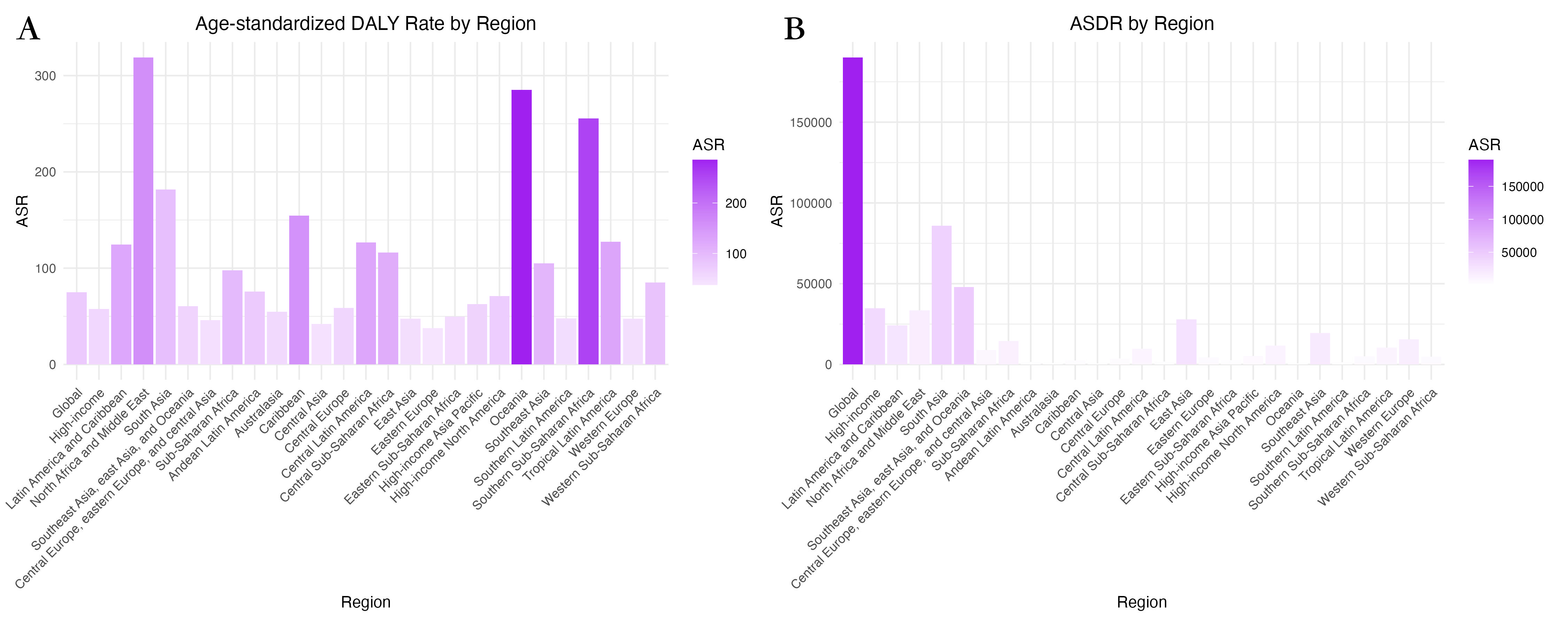


**Supplementary Figure 2: Age-standardized Death rate of global burden of diabetes and kidney diseases due to Low physical activity in 204 countries in 2021, by locations.**


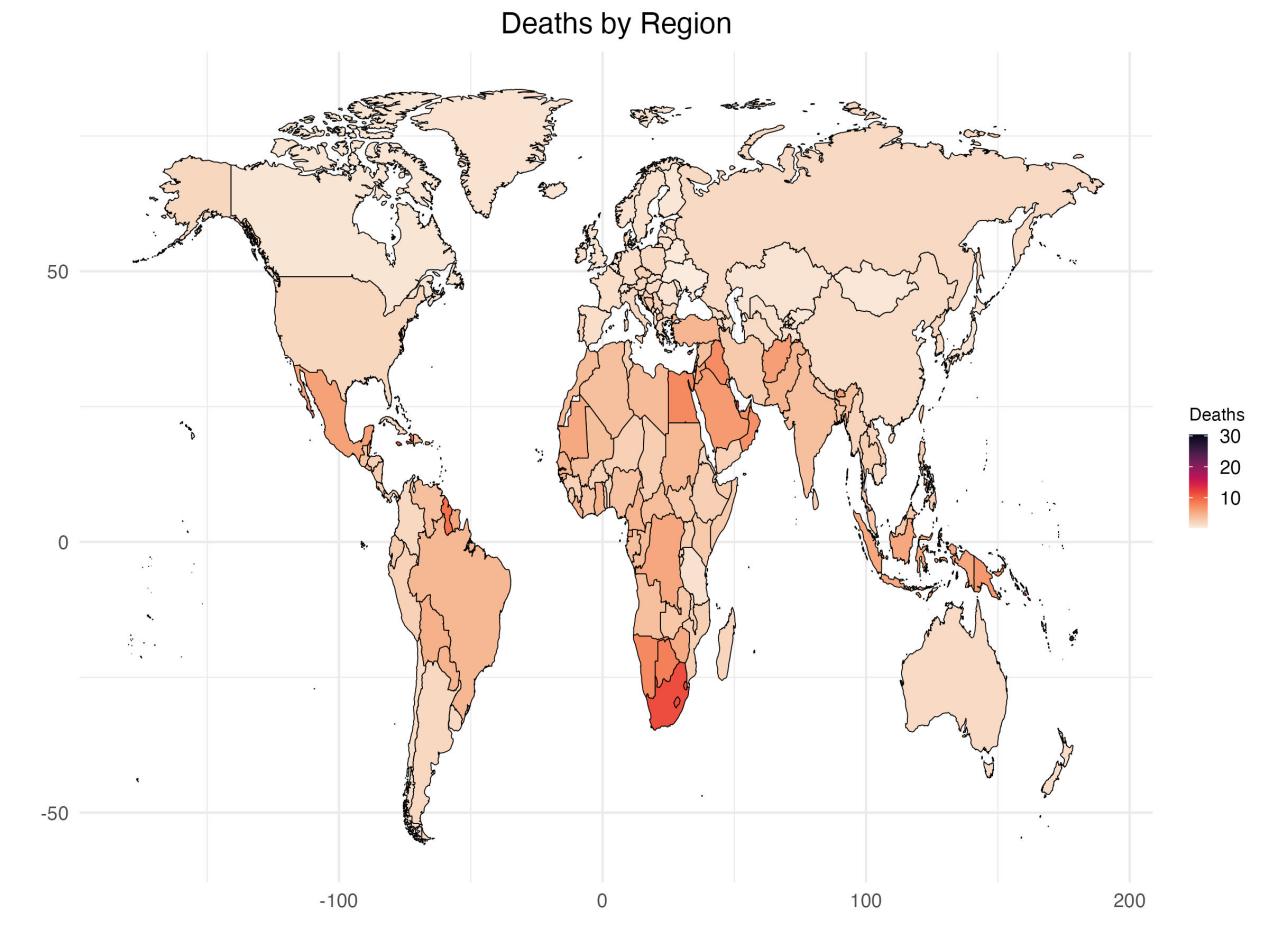


**Supplementary Figure 3: ASRs of global burden of diabetes and kidney diseases due to Low physical activity in 2021, by 7 super regions. (A) Age-standardized DALY rate (B) ASDR.**

**DALY = disability adjusted life-year. ASDR = age standardized deathrate. ASRs = age standardized rates.**


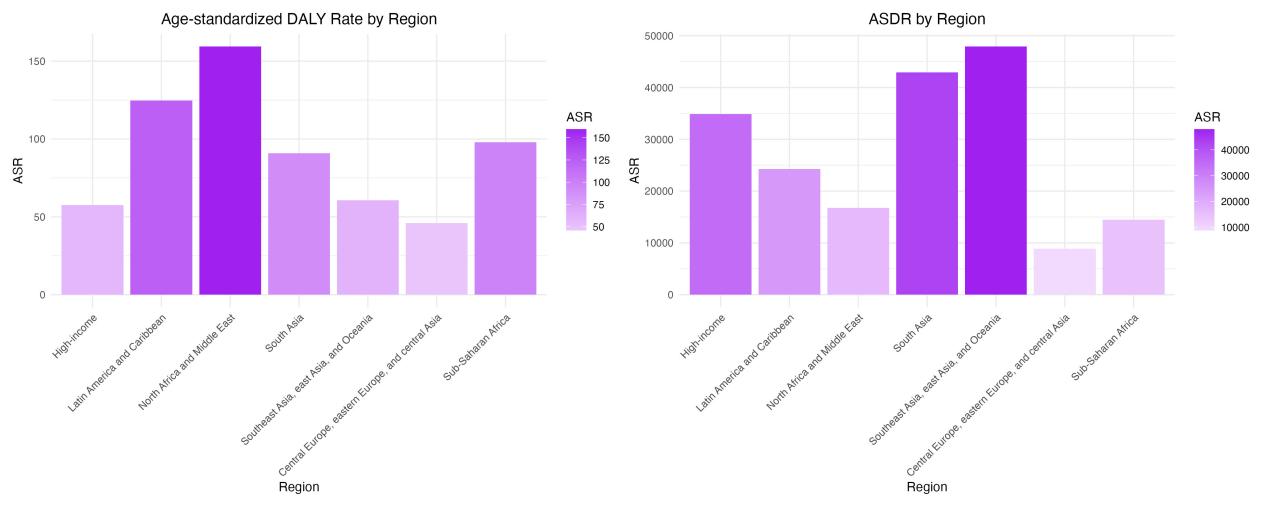


**Supplementary Figure 4: EAPC of ASRs of diabetes and kidney diseases due to Low physical activity from 1990-2021, by 7 super regions. (A) Age-standardized DALY rate (B) ASDR.**

**DALY = disability adjusted life-year. ASDR = age standardized deathrate. ASRs = age standardized rates.**


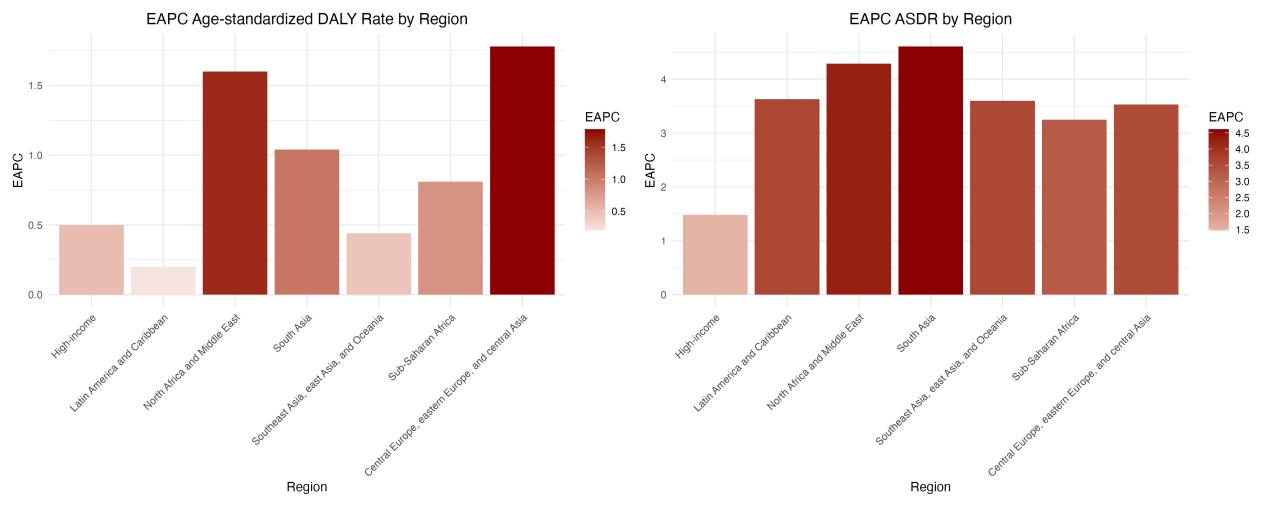


**Supplementary Figure 5: Scatter plot of correlation analysis between EAPC of DALY rate of global burden of diabetes and kidney diseases due to Low physical activity in 2021 and the level of SDI, by locations.**


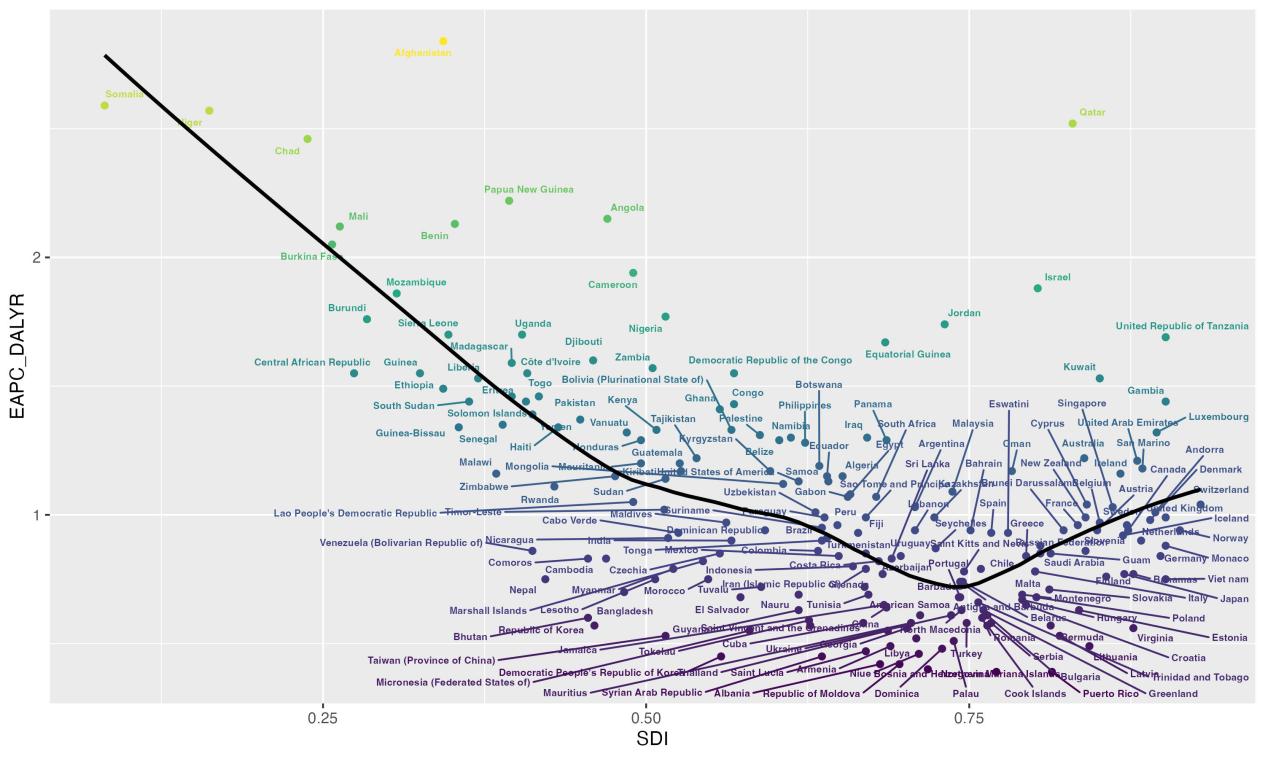


**Supplementary Figure 6: Scatter plot of correlation analysis between EAPC of Death rate of global burden of diabetes and kidney diseases due to Low physical activity in 2021 and the level of SDI, by locations.**


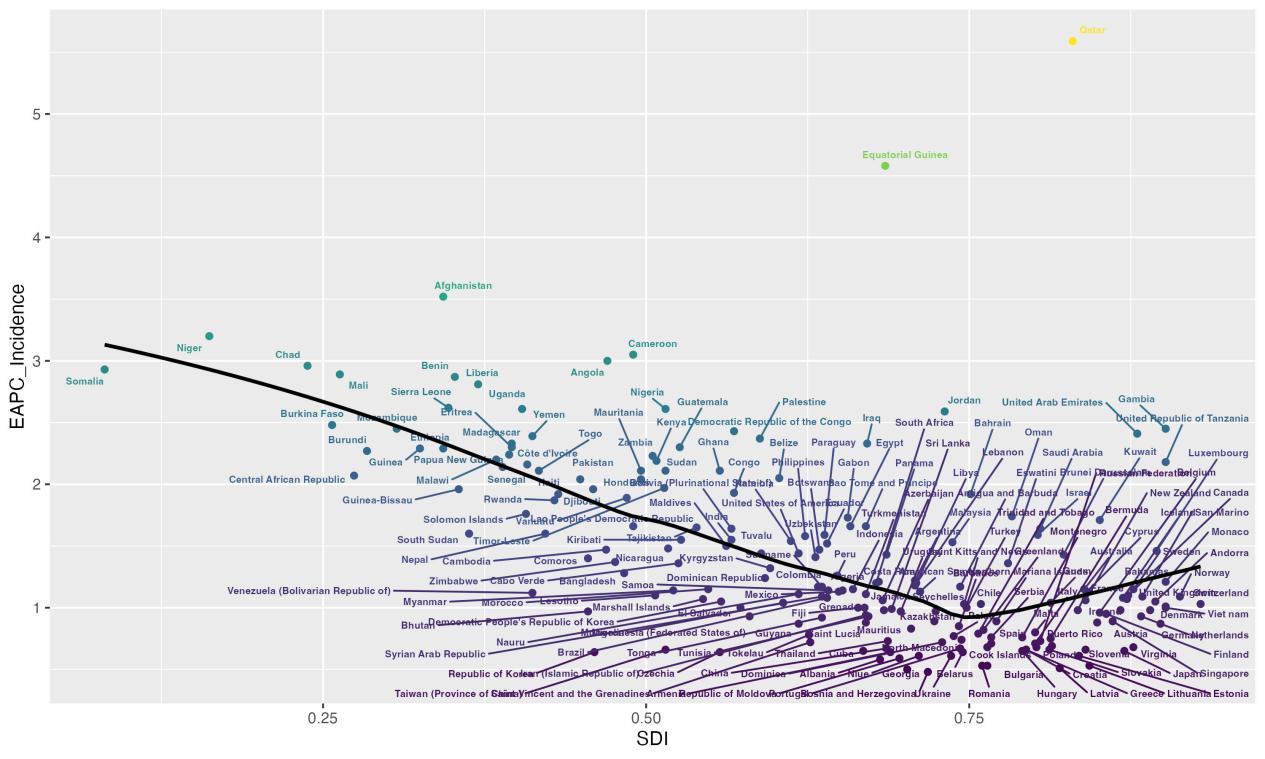

Supplement: Supplementary file 1 [file DataSheet1.docx]
