# Supplementary material for: Worldwide impact of disease attributable to low physical activity for diabetes and kidney diseases
Source: Front Endocrinol (Lausanne). 2025 May 5;16:1499381. doi: 10.3389/fendo.2025.1499381 (PMC12086069; doi:10.3389/fendo.2025.1499381)
Supplement: Supplementary file 2 [file Table1.doc]

Supplementary table1. The DALY cases and age-standardized DALY rate diabetes and kidney diseases due to Low physical activity in 1990 and 2021 and its temporal trends

| DALYs (Disability-Adjusted Life Years) |  | DALY No.(95%UI) | | | Age-standardized DALY rate (per 100000) No.95%UI | | |
| --- | --- | --- | --- | --- | --- | --- | --- |
| nation | sex | 1990 | 2021 | 1990-2021 EAPC No.(95%CI) | 1990 | 2021 | 1990-2021 EAPC No.(95%CI) |
| Afghanistan | both | 9029.08(3677.21,14323.94) | 25113.04(10594.79,40800.29) | 3.25(3.07,3.43) | 129.61(53.38,204.16) | 245.52(107.45,389.75) | 2.23(2.17,2.28) |
| Albania | both | 435.98(186.32,709.20) | 1281.57(564.08,2143.70) | 3.82(3.70,3.94) | 23.26(9.92,37.46) | 29.09(12.69,48.69) | 0.90(0.79,1.01) |
| Algeria | both | 8639.73(3571.19,14214.07) | 50598.89(21036.55,82055.90) | 6.01(5.86,6.17) | 76.27(30.91,124.21) | 143.24(58.74,227.00) | 2.25(2.14,2.37) |
| American Samoa | both | 105.97(48.90,164.76) | 349.22(153.20,551.42) | 3.70(3.26,4.15) | 433.80(199.65,671.31) | 693.29(305.25,1089.44) | 1.44(1.12,1.75) |
| Andorra | both | 22.12(9.17,36.61) | 67.32(29.68,111.19) | 3.39(3.19,3.60) | 41.17(16.91,68.58) | 42.73(18.70,70.78) | 0.25(0.10,0.41) |
| Angola | both | 2770.87(1131.06,4681.17) | 9982.90(3699.48,17050.08) | 4.20(4.01,4.39) | 80.79(31.50,133.26) | 93.28(35.23,158.25) | 0.35(0.28,0.43) |
| Antigua and Barbuda | both | 96.83(41.48,152.94) | 208.31(81.23,327.09) | 2.31(2.17,2.46) | 177.89(77.20,279.20) | 199.30(78.84,312.87) | 0.08(-0.05,0.21) |
| Argentina | both | 15733.86(6494.37,25593.42) | 27106.33(10746.16,45111.29) | 1.71(1.54,1.88) | 49.91(20.69,81.20) | 47.70(18.93,79.69) | -0.21(-0.36,-0.07) |
| Armenia | both | 954.33(399.07,1570.60) | 1874.93(757.84,3070.35) | 1.89(0.98,2.80) | 37.06(15.20,60.80) | 43.14(17.83,70.86) | 0.15(-0.61,0.93) |
| Australia | both | 9158.98(4019.37,14615.51) | 24975.44(10378.59,39573.94) | 3.15(3.06,3.25) | 46.96(20.39,74.80) | 55.23(23.16,87.31) | 0.42(0.33,0.51) |
| Austria | both | 4910.86(2062.81,7752.47) | 7927.87(3417.81,12801.36) | 2.22(1.86,2.59) | 39.07(16.33,61.20) | 39.37(16.52,63.19) | 0.50(0.15,0.84) |
| Azerbaijan | both | 1250.15(475.38,2121.72) | 4624.42(1752.10,8086.10) | 4.06(3.87,4.24) | 26.25(10.00,44.36) | 47.61(18.38,80.54) | 1.82(1.53,2.11) |
| Bahamas | both | 232.17(99.50,378.00) | 628.97(262.66,1035.24) | 3.18(3.05,3.30) | 153.14(66.51,248.84) | 155.26(64.15,256.08) | -0.12(-0.26,0.01) |
| Bahrain | both | 472.62(190.83,755.95) | 3307.45(1395.28,5307.52) | 6.39(6.26,6.53) | 318.49(130.98,500.25) | 432.30(183.84,677.78) | 0.72(0.42,1.03) |
| Bangladesh | both | 20629.53(8305.55,33667.13) | 92968.51(39235.93,151751.96) | 5.31(5.08,5.54) | 47.91(19.58,78.57) | 72.80(30.12,118.78) | 1.37(1.16,1.59) |
| Barbados | both | 836.90(376.45,1309.73) | 1437.53(650.50,2218.92) | 1.74(1.63,1.84) | 289.16(130.03,453.05) | 283.42(127.78,438.87) | -0.14(-0.30,0.02) |
| Belarus | both | 1884.67(786.65,3180.91) | 3323.70(1423.61,5489.45) | 1.19(0.88,1.51) | 14.64(6.14,24.51) | 20.58(8.76,34.13) | 0.51(0.22,0.79) |
| Belgium | both | 7146.48(2971.83,11333.34) | 10393.65(4603.87,16600.43) | 1.17(1.09,1.25) | 45.32(18.86,72.14) | 43.63(18.81,71.14) | -0.29(-0.39,-0.20) |
| Belize | both | 126.11(53.69,197.40) | 554.91(229.95,891.78) | 5.02(4.75,5.30) | 136.55(58.28,212.96) | 185.32(75.78,296.48) | 1.01(0.58,1.43) |
| Benin | both | 513.92(207.74,913.86) | 1909.70(714.01,3453.96) | 4.25(4.16,4.34) | 28.66(11.54,50.52) | 42.96(16.31,78.61) | 1.23(1.16,1.31) |
| Bermuda | both | 63.25(29.32,99.69) | 114.35(50.53,185.63) | 2.00(1.80,2.21) | 104.03(48.43,163.90) | 83.55(36.63,135.39) | -0.75(-0.91,-0.59) |
| Bhutan | both | 307.85(131.55,475.83) | 1059.98(460.30,1676.85) | 4.11(4.07,4.14) | 128.35(55.26,200.57) | 174.76(76.47,278.32) | 1.00(0.99,1.02) |
| Bolivia (Plurinational State of) | both | 2844.25(1079.65,4597.78) | 10323.56(3910.23,17742.23) | 4.46(4.36,4.56) | 94.80(35.34,152.96) | 118.45(45.35,203.80) | 0.76(0.73,0.79) |
| Bosnia and Herzegovina | both | 1697.55(736.89,2722.34) | 5742.28(2467.64,9378.20) | 4.48(4.09,4.87) | 44.25(19.20,71.18) | 88.89(38.51,144.48) | 2.70(2.45,2.95) |
| Botswana | both | 880.07(379.60,1471.12) | 2768.85(1133.19,4420.95) | 3.91(3.67,4.14) | 169.99(74.47,279.50) | 204.24(84.87,321.92) | 0.88(0.60,1.15) |
| Brazil | both | 107175.70(46388.91,167788.06) | 319135.01(133786.71,503028.30) | 3.63(3.56,3.71) | 126.22(54.91,194.59) | 127.91(53.57,200.89) | 0.05(-0.02,0.13) |
| Brunei Darussalam | both | 201.82(80.90,327.53) | 676.33(284.61,1115.84) | 4.09(3.96,4.22) | 211.13(84.97,344.64) | 196.91(79.98,326.24) | -0.15(-0.23,-0.07) |
| Bulgaria | both | 4963.36(1969.70,7908.62) | 8166.97(3364.18,13421.81) | 1.50(1.28,1.72) | 40.95(16.77,64.24) | 56.54(23.10,93.16) | 0.90(0.71,1.08) |
| Burkina Faso | both | 1949.84(797.53,3183.11) | 4685.72(1751.87,7969.37) | 2.84(2.69,2.99) | 50.71(20.86,82.23) | 56.10(20.87,93.66) | 0.26(0.19,0.34) |
| Burundi | both | 1184.55(471.42,2066.71) | 2069.15(764.09,3611.22) | 1.38(1.01,1.74) | 55.35(22.22,96.50) | 48.93(18.03,83.58) | -0.88(-1.05,-0.70) |
| Cabo Verde | both | 56.93(23.14,92.76) | 271.55(113.44,469.49) | 5.04(4.77,5.32) | 24.55(9.86,40.18) | 62.85(26.51,109.40) | 2.90(2.60,3.21) |
| Cambodia | both | 981.76(344.91,1694.95) | 3672.59(1406.54,6499.25) | 4.31(3.98,4.65) | 25.77(9.18,44.53) | 35.21(13.24,61.63) | 0.89(0.65,1.14) |
| Cameroon | both | 3072.95(1176.19,5133.22) | 12496.53(4954.42,20818.57) | 4.57(4.44,4.70) | 76.14(28.77,127.24) | 106.78(42.28,177.90) | 1.03(0.86,1.20) |
| Canada | both | 6702.58(2412.88,11187.57) | 19979.74(7582.89,35419.06) | 3.15(2.88,3.43) | 20.54(7.52,33.98) | 26.37(10.25,47.20) | 0.34(0.08,0.61) |
| Central African Republic | both | 1003.49(430.12,1641.75) | 2214.97(842.80,3900.28) | 2.53(2.45,2.60) | 92.61(39.28,154.58) | 101.64(40.98,177.88) | 0.24(0.18,0.30) |
| Chad | both | 1198.93(462.11,1954.18) | 3674.97(1411.24,6024.34) | 3.62(3.51,3.74) | 45.12(17.43,73.44) | 67.11(26.60,109.38) | 1.24(1.01,1.46) |
| Chile | both | 3768.74(1556.60,6389.25) | 12051.14(5058.89,19992.99) | 3.90(3.70,4.09) | 38.55(16.02,65.97) | 46.43(19.51,76.97) | 0.65(0.43,0.86) |
| China | both | 313717.49(129417.06,500063.45) | 946540.59(392536.83,1488766.20) | 3.48(3.40,3.57) | 41.16(17.46,64.91) | 45.97(19.24,72.17) | 0.19(0.08,0.31) |
| Colombia | both | 15597.76(6692.03,24652.60) | 51465.32(22113.89,84069.81) | 3.60(3.48,3.73) | 86.28(36.93,137.46) | 92.88(39.76,151.96) | -0.18(-0.35,-0.01) |
| Comoros | both | 76.20(30.49,127.68) | 231.75(87.91,404.56) | 3.61(3.44,3.77) | 42.73(16.85,70.59) | 50.42(19.11,87.28) | 0.45(0.33,0.57) |
| Congo | both | 965.61(373.12,1606.67) | 2657.43(1062.69,4458.95) | 3.10(2.79,3.41) | 94.06(37.02,155.76) | 98.71(37.67,165.29) | -0.05(-0.21,0.10) |
| Cook Islands | both | 43.32(16.75,69.50) | 85.66(36.65,138.58) | 2.07(1.97,2.17) | 340.79(131.85,541.59) | 336.49(144.63,544.31) | -0.19(-0.27,-0.11) |
| Costa Rica | both | 836.99(331.21,1330.42) | 3935.56(1549.74,6435.17) | 4.86(4.66,5.05) | 48.31(19.17,76.44) | 71.42(28.38,116.34) | 0.97(0.76,1.19) |
| Croatia | both | 2533.46(1071.11,4109.99) | 5607.15(2499.14,9165.96) | 2.36(2.17,2.55) | 44.58(18.74,72.25) | 58.70(26.00,95.14) | 0.54(0.33,0.76) |
| Cuba | both | 7736.38(3111.69,12201.01) | 14271.82(5910.78,23158.50) | 2.04(1.75,2.32) | 75.93(30.50,119.97) | 73.48(30.26,118.49) | -0.12(-0.41,0.17) |
| Cyprus | both | 1134.00(482.97,1812.44) | 1706.56(712.60,2786.09) | 0.94(0.79,1.08) | 174.19(74.27,276.31) | 87.49(35.92,141.66) | -2.54(-2.68,-2.40) |
| Czechia | both | 6720.15(2761.77,10912.46) | 16526.99(7522.04,26528.38) | 4.05(3.52,4.59) | 47.72(19.82,77.14) | 72.68(32.59,115.98) | 2.32(1.90,2.75) |
| Côte d'Ivoire | both | 2149.96(823.93,3669.49) | 8935.98(3509.59,15030.57) | 4.52(4.33,4.71) | 60.69(23.86,101.83) | 85.27(34.34,144.57) | 1.07(0.91,1.22) |
| Democratic People's Republic of Korea | both | 7474.69(3039.28,12298.76) | 19725.99(8083.37,32292.68) | 3.20(3.06,3.34) | 50.59(20.90,85.41) | 60.89(25.60,99.96) | 0.65(0.55,0.74) |
| Democratic Republic of the Congo | both | 16255.75(6523.04,26663.57) | 40174.85(15947.76,70449.99) | 2.73(2.52,2.94) | 123.69(48.63,203.18) | 126.21(50.85,215.07) | -0.03(-0.17,0.10) |
| Denmark | both | 2279.57(977.72,3538.31) | 4802.10(2051.20,7790.03) | 2.34(2.09,2.60) | 27.27(11.55,42.54) | 38.24(16.15,61.73) | 1.04(0.74,1.34) |
| Djibouti | both | 43.29(16.00,78.33) | 293.34(107.17,500.32) | 6.37(6.27,6.47) | 39.30(14.45,69.47) | 55.16(20.54,96.16) | 1.08(0.98,1.17) |
| Dominica | both | 91.31(37.26,148.20) | 139.26(54.90,226.03) | 1.14(1.04,1.23) | 153.52(62.72,246.45) | 173.16(68.61,279.33) | 0.16(0.07,0.24) |
| Dominican Republic | both | 2960.16(1198.35,4754.83) | 13161.20(5522.22,21216.56) | 5.19(5.05,5.34) | 82.45(33.65,132.99) | 131.12(55.27,210.83) | 1.84(1.71,1.96) |
| Ecuador | both | 2787.67(1143.29,4477.04) | 13666.13(5337.18,22885.09) | 5.22(4.68,5.77) | 54.16(22.19,87.80) | 84.23(33.01,140.67) | 1.32(0.82,1.82) |
| Egypt | both | 27416.18(11668.13,42260.59) | 117868.95(50392.30,194865.97) | 5.05(4.93,5.16) | 109.49(46.90,168.00) | 200.08(87.49,317.77) | 2.37(2.17,2.56) |
| El Salvador | both | 1619.45(671.04,2625.32) | 7265.35(2948.61,12355.01) | 4.90(4.60,5.20) | 55.06(23.10,89.85) | 117.54(47.61,199.89) | 2.40(2.14,2.66) |
| Equatorial Guinea | both | 161.78(64.49,273.66) | 612.92(223.43,1068.52) | 4.83(4.40,5.26) | 92.01(36.90,154.14) | 129.66(48.39,224.42) | 1.35(1.10,1.60) |
| Eritrea | both | 253.63(95.55,450.06) | 787.48(292.38,1445.90) | 4.18(4.01,4.35) | 26.78(9.90,49.61) | 33.24(12.40,60.19) | 0.79(0.75,0.83) |
| Estonia | both | 323.94(130.73,543.69) | 1092.27(435.17,1784.22) | 3.87(3.54,4.19) | 15.72(6.28,26.67) | 37.74(14.72,61.58) | 2.60(2.32,2.89) |
| Eswatini | both | 425.55(179.42,700.55) | 1421.78(573.93,2399.98) | 4.23(3.49,4.97) | 163.14(69.49,268.62) | 269.65(110.11,453.81) | 1.99(1.37,2.63) |
| Ethiopia | both | 15214.03(6116.86,24794.46) | 22636.74(8300.94,37497.76) | 0.86(0.53,1.18) | 82.27(32.76,134.46) | 55.51(20.50,91.55) | -1.67(-1.88,-1.47) |
| Fiji | both | 1666.56(726.15,2722.35) | 5150.55(2215.76,8488.94) | 3.72(3.46,3.98) | 436.27(186.83,720.36) | 650.36(284.94,1055.20) | 1.12(0.88,1.37) |
| Finland | both | 2164.43(924.98,3373.66) | 4535.93(1883.36,7317.82) | 2.40(2.16,2.64) | 29.90(12.76,46.96) | 36.24(14.90,59.85) | 0.54(0.32,0.76) |
| France | both | 28059.22(12240.95,44407.48) | 51866.29(22882.86,83344.12) | 2.09(1.78,2.39) | 32.27(14.21,51.29) | 34.12(15.00,55.20) | 0.18(-0.15,0.51) |
| Gabon | both | 300.84(114.72,505.31) | 700.31(276.09,1204.84) | 2.59(2.46,2.73) | 58.29(21.96,100.16) | 76.71(29.70,133.91) | 0.74(0.56,0.92) |
| Gambia | both | 129.00(51.04,215.62) | 642.11(250.40,1065.25) | 5.23(4.98,5.47) | 41.51(16.58,68.59) | 69.96(27.40,117.55) | 1.60(1.47,1.72) |
| Georgia | both | 1621.49(676.94,2660.17) | 3110.54(1231.55,5154.60) | 2.78(2.35,3.21) | 26.34(11.08,42.62) | 51.25(20.29,85.59) | 2.86(2.45,3.28) |
| Germany | both | 49928.32(21726.63,78367.08) | 71538.32(31463.20,117775.27) | 0.97(0.72,1.23) | 37.06(16.05,58.30) | 33.94(14.49,55.71) | -0.63(-0.90,-0.37) |
| Ghana | both | 3518.57(1383.44,5680.63) | 16388.43(6443.66,27155.01) | 5.36(5.14,5.58) | 62.71(24.53,100.88) | 105.89(41.94,171.00) | 1.97(1.80,2.13) |
| Greece | both | 5920.01(2594.61,9662.08) | 11175.92(4479.74,18273.89) | 1.93(1.60,2.27) | 39.31(16.95,64.49) | 42.24(17.00,69.01) | -0.02(-0.36,0.31) |
| Greenland | both | 8.16(3.15,12.88) | 18.64(7.42,31.22) | 2.66(2.40,2.93) | 30.05(11.91,47.70) | 29.53(12.16,48.64) | -0.20(-0.44,0.03) |
| Grenada | both | 156.08(62.39,260.58) | 257.91(106.37,424.43) | 1.94(1.78,2.10) | 212.06(85.32,352.01) | 236.20(99.25,386.34) | 0.49(0.31,0.67) |
| Guam | both | 65.32(28.32,104.22) | 147.16(59.18,240.32) | 2.66(2.51,2.82) | 96.22(40.84,151.80) | 69.85(28.08,114.02) | -0.89(-1.16,-0.61) |
| Guatemala | both | 984.41(368.84,1709.51) | 8980.96(3155.84,14858.62) | 7.30(7.01,7.60) | 33.80(12.73,56.55) | 83.68(29.71,140.94) | 2.61(2.33,2.88) |
| Guinea | both | 1069.77(422.23,1781.26) | 2510.31(987.31,4356.32) | 2.57(2.31,2.83) | 34.95(13.82,58.10) | 48.49(17.83,83.33) | 1.04(0.92,1.15) |
| Guinea-Bissau | both | 305.22(116.66,523.75) | 758.34(281.90,1244.91) | 3.01(2.97,3.05) | 82.58(31.19,140.59) | 112.32(42.72,187.14) | 1.02(0.90,1.14) |
| Guyana | both | 821.05(332.26,1317.28) | 1863.71(747.13,3115.41) | 2.70(2.43,2.96) | 220.13(90.02,351.13) | 290.18(115.63,476.77) | 0.76(0.42,1.10) |
| Haiti | both | 7066.55(2709.24,11612.23) | 17348.52(6978.16,29463.66) | 3.02(2.95,3.09) | 226.71(87.01,374.17) | 246.10(99.71,414.61) | 0.35(0.30,0.40) |
| Honduras | both | 874.89(335.18,1524.53) | 5356.09(2156.14,9036.28) | 6.22(6.08,6.36) | 43.30(16.83,74.66) | 84.87(34.79,143.23) | 2.29(2.14,2.43) |
| Hungary | both | 6102.39(2572.28,9755.37) | 10942.00(4455.63,17765.18) | 2.17(1.94,2.39) | 40.76(16.94,65.68) | 53.73(21.88,87.21) | 1.06(0.84,1.29) |
| Iceland | both | 66.42(28.68,105.26) | 194.10(81.98,305.33) | 3.58(3.48,3.67) | 22.63(9.65,35.87) | 33.49(14.06,53.07) | 1.30(1.24,1.36) |
| India | both | 271016.15(116097.95,419484.52) | 996772.03(460023.22,1567939.86) | 4.26(4.12,4.41) | 66.34(28.01,102.66) | 89.94(41.37,140.79) | 0.95(0.79,1.11) |
| Indonesia | both | 99585.43(41994.11,153803.06) | 368337.31(161239.96,568639.47) | 4.04(3.94,4.14) | 107.86(45.83,166.73) | 162.42(71.17,249.42) | 1.29(1.25,1.34) |
| Iran (Islamic Republic of) | both | 14831.71(6273.35,23544.59) | 83156.48(35798.31,132406.63) | 6.14(5.98,6.30) | 61.75(26.16,98.80) | 107.58(46.27,171.61) | 2.13(1.99,2.27) |
| Iraq | both | 16175.35(6672.83,25591.92) | 70947.17(30065.05,110640.06) | 4.93(4.76,5.09) | 199.65(81.28,315.60) | 282.24(121.62,444.39) | 1.01(0.89,1.13) |
| Ireland | both | 1829.89(789.06,2842.34) | 3162.91(1318.89,5078.84) | 1.90(1.73,2.07) | 44.49(19.27,69.57) | 39.54(16.70,63.03) | -0.31(-0.41,-0.21) |
| Israel | both | 3180.50(1269.66,5054.80) | 8214.57(3260.33,13053.06) | 2.72(2.09,3.36) | 66.33(26.58,105.80) | 63.72(25.36,101.10) | -0.46(-1.07,0.15) |
| Italy | both | 61082.91(26337.35,93912.78) | 81826.95(35999.63,131323.57) | 1.18(1.07,1.28) | 66.87(28.81,102.65) | 52.29(22.59,83.69) | -0.66(-0.74,-0.58) |
| Jamaica | both | 4278.29(1829.94,6708.10) | 8060.59(3616.27,12603.67) | 1.75(1.52,1.98) | 237.57(101.71,373.21) | 259.73(116.69,406.56) | -0.02(-0.24,0.20) |
| Japan | both | 83364.29(35406.90,131036.73) | 181679.47(75644.54,306070.21) | 2.51(2.40,2.62) | 49.70(20.97,78.19) | 56.88(23.89,94.67) | 0.38(0.29,0.48) |
| Jordan | both | 2271.44(960.38,3744.95) | 13590.65(5600.82,22636.24) | 5.59(5.40,5.79) | 185.93(77.67,304.66) | 189.15(76.37,309.49) | -0.24(-0.54,0.07) |
| Kazakhstan | both | 3368.28(1414.22,5659.23) | 8267.20(3446.81,13802.76) | 2.36(2.07,2.66) | 26.85(11.24,43.54) | 46.16(19.15,77.24) | 1.28(1.05,1.51) |
| Kenya | both | 3440.76(1382.77,5470.21) | 13874.82(5458.59,23043.49) | 4.83(4.74,4.92) | 44.22(18.49,70.73) | 64.17(24.70,106.42) | 1.44(1.33,1.54) |
| Kiribati | both | 199.07(84.62,309.51) | 544.18(230.27,891.10) | 3.38(3.26,3.51) | 506.97(219.56,797.21) | 694.76(294.06,1126.88) | 0.97(0.79,1.15) |
| Kuwait | both | 1129.53(494.57,1789.04) | 8767.98(3831.58,14277.26) | 6.99(6.73,7.25) | 164.80(74.24,255.38) | 248.38(109.20,398.61) | 1.37(1.13,1.60) |
| Kyrgyzstan | both | 515.34(217.24,857.29) | 1427.05(572.64,2466.96) | 2.67(2.42,2.92) | 17.88(7.43,29.51) | 30.95(12.88,52.66) | 1.32(1.07,1.56) |
| Lao People's Democratic Republic | both | 1038.24(392.96,1731.71) | 2461.59(974.77,4231.34) | 2.48(2.24,2.73) | 52.86(20.16,88.13) | 58.18(22.85,101.52) | 0.08(-0.05,0.21) |
| Latvia | both | 706.38(292.40,1164.09) | 1747.29(727.03,2844.04) | 2.87(2.64,3.11) | 19.77(8.31,32.25) | 44.40(18.53,71.52) | 2.51(2.26,2.77) |
| Lebanon | both | 2930.44(1252.16,4791.28) | 10275.17(4295.08,16736.90) | 4.53(4.39,4.67) | 139.51(59.16,228.34) | 168.27(70.42,273.66) | 0.87(0.74,0.99) |
| Lesotho | both | 360.31(146.66,627.59) | 935.93(376.76,1655.01) | 3.67(3.10,4.23) | 48.97(19.77,83.06) | 106.20(42.96,181.36) | 3.31(2.78,3.83) |
| Liberia | both | 959.19(378.49,1614.82) | 2937.12(1221.16,4838.99) | 3.88(3.68,4.08) | 85.04(33.89,140.42) | 130.48(55.21,219.20) | 1.54(1.40,1.68) |
| Libya | both | 1437.22(625.09,2277.53) | 8897.36(3772.93,14500.90) | 6.58(6.43,6.73) | 76.66(33.42,120.23) | 164.28(70.20,263.48) | 3.02(2.80,3.23) |
| Lithuania | both | 808.98(333.61,1344.17) | 2452.93(1015.62,4016.15) | 3.36(3.07,3.65) | 17.95(7.42,29.59) | 42.12(17.15,69.13) | 2.46(2.13,2.79) |
| Luxembourg | both | 216.73(87.20,340.18) | 381.28(159.66,615.48) | 1.78(1.62,1.94) | 39.69(15.85,62.08) | 34.98(14.53,56.63) | -0.51(-0.59,-0.43) |
| Madagascar | both | 1707.21(620.81,2980.90) | 4051.47(1605.82,7232.51) | 2.83(2.57,3.08) | 37.33(13.92,64.59) | 41.72(17.42,71.10) | 0.31(0.26,0.37) |
| Malawi | both | 1463.34(570.25,2524.82) | 3314.09(1307.77,5878.07) | 2.34(2.19,2.49) | 42.27(16.91,71.73) | 49.42(19.39,86.92) | 0.27(0.05,0.48) |
| Malaysia | both | 9235.69(3908.82,14589.28) | 33695.73(14077.72,53469.39) | 4.24(4.08,4.39) | 102.99(44.28,161.51) | 119.33(48.94,190.09) | 0.33(0.19,0.48) |
| Maldives | both | 194.32(82.33,295.03) | 508.22(224.25,801.00) | 2.75(2.39,3.11) | 211.16(88.30,323.22) | 152.81(68.04,240.45) | -1.36(-1.60,-1.11) |
| Mali | both | 2964.37(1358.29,4774.84) | 9658.37(4001.92,16041.24) | 4.17(4.05,4.30) | 80.54(36.35,131.91) | 114.64(47.35,191.42) | 1.23(1.16,1.29) |
| Malta | both | 336.07(146.87,521.56) | 770.95(333.61,1220.79) | 2.75(2.48,3.01) | 80.51(35.26,124.41) | 79.30(34.38,124.88) | -0.07(-0.33,0.18) |
| Marshall Islands | both | 101.86(44.63,159.98) | 410.22(178.62,643.69) | 4.67(4.42,4.91) | 585.22(258.49,924.91) | 1010.40(430.69,1569.41) | 1.70(1.44,1.96) |
| Mauritania | both | 1035.91(403.93,1687.60) | 2867.35(1211.14,4683.68) | 3.20(3.06,3.35) | 107.41(41.13,172.75) | 135.25(56.70,219.98) | 0.55(0.46,0.63) |
| Mauritius | both | 850.06(359.22,1371.09) | 4632.04(1961.30,7523.59) | 6.73(6.04,7.43) | 118.24(49.93,186.15) | 248.83(105.57,397.92) | 3.40(2.70,4.11) |
| Mexico | both | 61607.59(24555.52,97023.20) | 203175.57(84645.49,330400.22) | 3.93(3.63,4.22) | 150.87(59.50,236.83) | 160.69(66.74,260.76) | 0.13(-0.13,0.40) |
| Micronesia (Federated States of) | both | 237.04(100.52,371.38) | 568.45(247.05,884.16) | 2.91(2.75,3.08) | 474.76(202.14,740.23) | 703.44(308.71,1086.47) | 1.28(1.02,1.54) |
| Monaco | both | 16.16(6.99,26.08) | 33.93(14.39,55.02) | 2.39(2.31,2.47) | 21.98(9.33,35.42) | 34.57(14.06,56.62) | 1.50(1.44,1.55) |
| Mongolia | both | 139.21(56.46,245.75) | 466.49(199.99,803.50) | 3.89(3.51,4.28) | 13.61(5.39,23.96) | 20.71(8.04,35.14) | 1.25(1.13,1.37) |
| Montenegro | both | 272.86(110.95,435.80) | 652.39(271.47,1067.79) | 2.97(2.80,3.14) | 45.06(18.65,71.30) | 67.16(27.88,110.22) | 1.33(1.26,1.41) |
| Morocco | both | 11655.82(4810.09,18730.18) | 65148.67(27718.82,102995.77) | 5.97(5.85,6.10) | 82.66(34.34,131.91) | 186.93(79.12,296.15) | 2.87(2.79,2.96) |
| Mozambique | both | 1985.82(732.51,3440.69) | 5224.44(1988.44,9032.02) | 3.59(3.43,3.75) | 37.89(14.30,65.53) | 52.94(20.24,91.63) | 1.49(1.34,1.64) |
| Myanmar | both | 9587.77(3525.10,17359.50) | 25522.70(9527.59,43991.10) | 2.99(2.69,3.29) | 44.14(17.02,76.57) | 55.68(20.52,95.25) | 0.61(0.42,0.81) |
| Namibia | both | 815.25(350.55,1306.64) | 2225.27(902.97,3619.68) | 2.92(2.60,3.25) | 135.31(56.87,218.01) | 174.13(70.61,279.29) | 0.59(0.29,0.89) |
| Nauru | both | 14.93(5.65,25.27) | 27.72(10.63,45.36) | 1.71(1.49,1.93) | 297.56(112.87,499.41) | 443.62(172.35,719.87) | 1.15(0.97,1.33) |
| Nepal | both | 3365.66(1355.45,5588.04) | 14356.07(5390.97,24647.44) | 5.03(4.75,5.32) | 40.49(15.96,68.08) | 65.58(25.02,112.07) | 1.63(1.39,1.87) |
| Netherlands | both | 7823.00(3144.73,12421.46) | 10319.41(4248.40,17009.47) | 0.74(0.53,0.94) | 38.02(15.21,60.60) | 27.87(11.54,46.33) | -1.20(-1.41,-1.00) |
| New Zealand | both | 1566.20(656.23,2500.22) | 4366.67(1925.53,6962.94) | 3.28(3.16,3.40) | 39.59(16.59,62.72) | 51.59(22.92,82.29) | 0.82(0.72,0.93) |
| Nicaragua | both | 918.20(363.22,1526.32) | 4599.85(1760.97,8044.36) | 5.35(5.14,5.56) | 61.15(24.17,102.62) | 94.23(36.02,164.70) | 1.46(1.29,1.64) |
| Niger | both | 1232.20(470.12,2062.23) | 5040.17(1986.64,8481.53) | 4.69(4.62,4.75) | 48.19(18.92,78.72) | 64.95(25.79,110.44) | 0.96(0.89,1.02) |
| Nigeria | both | 29367.12(12237.72,46589.45) | 69739.14(27080.96,113559.33) | 2.66(2.51,2.81) | 71.82(30.35,113.12) | 82.15(32.18,132.71) | 0.33(0.27,0.38) |
| Niue | both | 5.10(2.10,8.18) | 8.30(3.56,13.19) | 1.30(1.18,1.42) | 234.34(96.16,376.22) | 383.34(165.40,607.60) | 1.48(1.34,1.61) |
| North Macedonia | both | 1021.19(418.05,1686.59) | 2934.18(1214.65,4759.97) | 3.64(3.33,3.95) | 57.89(23.70,94.97) | 92.20(38.99,149.17) | 1.59(1.27,1.90) |
| Northern Mariana Islands | both | 36.70(15.04,59.30) | 116.23(50.97,184.46) | 4.02(3.63,4.42) | 193.18(82.92,312.88) | 212.90(95.43,330.71) | 0.26(0.09,0.44) |
| Norway | both | 2125.14(861.96,3413.73) | 3427.03(1416.80,5470.46) | 1.39(1.25,1.54) | 30.51(12.25,49.12) | 33.61(13.84,53.40) | 0.20(0.04,0.36) |
| Oman | both | 929.06(391.50,1530.13) | 3872.10(1610.63,6106.44) | 4.62(4.33,4.91) | 144.65(61.07,239.54) | 197.33(83.76,310.73) | 1.27(1.11,1.43) |
| Pakistan | both | 37957.76(16412.37,61201.07) | 145396.58(62463.26,234577.39) | 4.26(4.13,4.39) | 70.75(30.22,113.82) | 124.88(52.81,199.25) | 1.71(1.46,1.96) |
| Palau | both | 23.53(9.57,38.09) | 76.37(32.02,125.85) | 3.63(3.40,3.86) | 236.79(97.66,382.17) | 327.71(134.80,541.01) | 1.13(1.01,1.25) |
| Palestine | both | 1439.68(597.87,2251.23) | 5211.77(2286.21,8043.77) | 4.32(4.24,4.39) | 176.53(73.82,275.22) | 216.98(94.18,333.55) | 0.74(0.59,0.88) |
| Panama | both | 759.25(305.65,1200.36) | 3616.14(1527.31,6009.62) | 5.08(4.91,5.25) | 52.04(20.92,82.56) | 81.53(34.31,135.87) | 1.35(1.17,1.52) |
| Papua New Guinea | both | 3532.50(1375.89,6081.33) | 12220.31(4803.31,20180.46) | 4.11(3.96,4.27) | 180.38(70.74,305.97) | 213.44(83.00,350.13) | 0.50(0.46,0.54) |
| Paraguay | both | 1271.12(469.38,2150.60) | 6313.67(2568.49,10809.12) | 5.52(5.28,5.76) | 59.86(22.20,101.37) | 111.39(45.09,189.82) | 2.25(2.03,2.48) |
| Peru | both | 5333.08(2194.64,8767.83) | 20212.27(8800.87,33051.57) | 4.36(4.17,4.55) | 47.01(19.47,76.72) | 60.89(26.43,99.44) | 0.65(0.46,0.84) |
| Philippines | both | 13063.38(5276.54,21260.60) | 56123.01(22454.95,90486.88) | 5.01(4.92,5.10) | 49.54(20.02,81.48) | 72.09(29.32,116.83) | 1.40(1.34,1.46) |
| Poland | both | 24707.40(10783.04,40136.35) | 47112.00(20407.20,74912.02) | 2.30(2.06,2.53) | 56.25(24.27,90.76) | 64.30(27.93,103.53) | 0.52(0.34,0.70) |
| Portugal | both | 10353.71(4359.07,16133.20) | 19116.31(7887.73,30613.67) | 1.77(1.50,2.03) | 74.36(31.36,115.89) | 74.10(31.24,119.22) | -0.34(-0.58,-0.10) |
| Puerto Rico | both | 6039.05(2549.86,9408.68) | 11641.56(4959.94,18896.87) | 1.96(1.69,2.23) | 169.29(71.80,263.05) | 164.87(70.78,270.23) | -0.26(-0.44,-0.09) |
| Qatar | both | 262.99(106.75,411.61) | 3289.54(1372.17,5482.60) | 9.06(8.78,9.34) | 282.93(117.09,439.07) | 353.45(146.51,562.66) | 0.66(0.02,1.31) |
| Republic of Korea | both | 20785.12(8692.39,32805.20) | 77306.15(32266.34,130838.05) | 4.10(3.91,4.30) | 72.06(30.99,113.05) | 84.05(34.98,140.41) | 0.12(-0.13,0.36) |
| Republic of Moldova | both | 980.41(408.15,1668.68) | 2249.23(916.36,3774.62) | 2.29(1.97,2.61) | 23.07(9.59,38.29) | 37.50(15.16,62.82) | 1.21(0.93,1.49) |
| Romania | both | 8862.24(3697.11,14076.34) | 15245.70(6226.30,24290.41) | 2.00(1.83,2.18) | 32.28(13.45,50.59) | 40.56(16.43,65.23) | 0.93(0.76,1.11) |
| Russian Federation | both | 31190.82(12860.17,49574.39) | 112272.16(45571.83,176495.48) | 3.75(3.08,4.42) | 17.66(7.50,28.06) | 45.65(18.47,72.36) | 2.74(2.13,3.36) |
| Rwanda | both | 1391.47(545.93,2458.85) | 2381.00(843.07,4488.56) | 1.20(0.62,1.78) | 54.96(20.79,94.80) | 44.20(15.68,81.36) | -1.55(-1.89,-1.20) |
| Saint Kitts and Nevis | both | 80.14(34.44,130.60) | 127.08(52.67,212.35) | 1.91(1.56,2.26) | 212.38(91.89,338.23) | 192.43(80.97,318.23) | 0.06(-0.13,0.25) |
| Saint Lucia | both | 214.75(86.63,337.61) | 476.06(198.31,776.40) | 2.13(1.89,2.37) | 255.59(102.83,404.08) | 199.43(83.57,323.52) | -1.47(-1.70,-1.24) |
| Saint Vincent and the Grenadines | both | 176.77(76.34,283.87) | 311.20(131.90,506.21) | 1.66(1.43,1.88) | 253.05(109.17,406.34) | 221.31(93.18,361.24) | -0.72(-0.92,-0.51) |
| Samoa | both | 242.30(111.60,377.80) | 663.01(295.91,1069.22) | 3.32(3.25,3.39) | 276.56(126.75,436.99) | 438.35(195.44,708.33) | 1.50(1.43,1.56) |
| San Marino | both | 10.69(4.44,16.79) | 25.53(10.37,42.39) | 3.18(2.99,3.37) | 29.41(12.19,46.11) | 33.94(13.60,57.49) | 0.76(0.65,0.88) |
| Sao Tome and Principe | both | 17.98(7.16,30.00) | 46.49(18.41,79.17) | 2.98(2.87,3.09) | 30.16(12.00,51.26) | 44.99(17.47,75.87) | 1.24(1.15,1.32) |
| Saudi Arabia | both | 7169.22(3005.71,11492.22) | 46540.25(18715.11,74790.24) | 5.91(5.76,6.07) | 126.12(53.89,202.23) | 212.81(88.32,338.71) | 1.53(1.42,1.64) |
| Senegal | both | 2611.22(1080.42,4247.35) | 9360.18(4115.10,15350.68) | 4.30(4.19,4.41) | 85.16(35.47,138.55) | 125.88(54.68,206.01) | 1.27(1.19,1.36) |
| Serbia | both | 5864.87(2390.08,9446.01) | 12482.06(5153.11,20599.98) | 2.35(2.14,2.56) | 58.79(24.20,93.97) | 72.45(29.82,120.91) | 0.57(0.47,0.67) |
| Seychelles | both | 35.80(15.09,57.83) | 137.34(55.87,228.83) | 4.59(4.51,4.68) | 63.45(26.54,102.25) | 125.48(51.06,207.27) | 2.38(2.22,2.55) |
| Sierra Leone | both | 953.75(397.18,1596.48) | 2671.41(1092.12,4378.51) | 3.47(3.41,3.53) | 49.76(20.57,82.09) | 74.31(30.21,122.63) | 1.45(1.26,1.63) |
| Singapore | both | 1628.58(695.25,2579.96) | 4596.54(1936.96,7710.50) | 3.53(3.41,3.64) | 72.54(31.20,115.72) | 53.10(22.00,89.30) | -0.96(-1.15,-0.77) |
| Slovakia | both | 2035.18(851.28,3253.62) | 3486.62(1434.85,5827.92) | 1.80(1.72,1.88) | 33.72(14.08,54.21) | 36.09(14.76,60.64) | 0.24(0.20,0.29) |
| Slovenia | both | 921.41(378.70,1521.25) | 1722.59(707.81,2809.82) | 1.28(0.99,1.57) | 37.13(15.15,61.30) | 37.13(15.34,60.35) | -0.91(-1.22,-0.59) |
| Solomon Islands | both | 360.43(141.03,616.63) | 1316.57(523.87,2142.19) | 4.19(4.08,4.30) | 246.31(100.80,413.89) | 342.58(139.53,556.25) | 1.08(0.99,1.17) |
| Somalia | both | 1125.55(394.04,2024.91) | 3340.68(1196.91,5861.16) | 3.73(3.62,3.84) | 55.01(20.40,95.23) | 63.50(22.68,110.02) | 0.52(0.43,0.61) |
| South Africa | both | 33469.14(14420.23,51638.13) | 125244.15(54515.89,189103.56) | 4.43(4.08,4.77) | 165.25(71.89,254.38) | 280.38(121.54,426.01) | 1.87(1.52,2.23) |
| South Sudan | both | 1063.21(394.18,1880.90) | 2091.53(745.39,3824.66) | 2.09(1.77,2.41) | 45.71(16.92,81.87) | 61.49(21.29,107.28) | 0.88(0.72,1.05) |
| Spain | both | 36530.77(15492.19,58125.69) | 59036.68(24581.93,95418.16) | 1.46(1.33,1.59) | 65.78(27.89,103.76) | 57.85(24.12,93.29) | -0.58(-0.65,-0.51) |
| Sri Lanka | both | 4415.75(1649.81,7601.07) | 21665.89(8289.93,36740.99) | 5.70(5.54,5.86) | 45.59(17.11,78.65) | 80.55(31.12,133.49) | 2.30(2.12,2.49) |
| Sudan | both | 10691.56(4785.82,16910.91) | 42642.47(19341.68,65453.39) | 4.56(4.40,4.72) | 111.00(49.33,174.60) | 194.70(86.90,300.76) | 1.95(1.85,2.04) |
| Suriname | both | 411.49(178.58,663.38) | 1465.73(625.80,2326.60) | 4.60(4.41,4.80) | 158.10(67.77,253.22) | 226.78(96.50,362.70) | 1.40(1.25,1.55) |
| Sweden | both | 4142.90(1794.45,6611.84) | 7510.74(3138.86,12369.83) | 2.09(1.99,2.19) | 26.47(11.42,41.94) | 34.33(14.39,55.41) | 1.04(0.94,1.13) |
| Switzerland | both | 5050.77(2072.23,7837.65) | 8361.14(3177.91,13707.47) | 1.45(1.30,1.60) | 46.38(19.01,72.26) | 45.48(17.24,75.09) | -0.26(-0.38,-0.14) |
| Syrian Arab Republic | both | 4975.73(2035.15,7914.17) | 17744.20(7451.98,28762.22) | 4.06(3.90,4.22) | 97.67(40.22,155.15) | 136.18(57.43,220.02) | 0.76(0.58,0.94) |
| Taiwan (Province of China) | both | 19150.46(8149.44,29983.72) | 45987.14(19904.82,72907.24) | 2.24(1.92,2.57) | 124.50(53.08,196.24) | 108.60(46.89,172.97) | -1.08(-1.40,-0.76) |
| Tajikistan | both | 598.83(240.75,962.51) | 1890.31(735.26,3284.77) | 3.52(3.35,3.69) | 22.66(9.16,36.01) | 34.54(13.90,59.36) | 1.20(0.97,1.43) |
| Thailand | both | 19482.03(7557.85,33084.06) | 83233.39(35469.25,135649.53) | 4.38(4.23,4.53) | 61.05(23.89,101.78) | 76.44(32.64,124.69) | 0.28(0.12,0.44) |
| Timor-Leste | both | 66.03(24.87,118.24) | 321.81(123.20,565.75) | 5.60(5.40,5.81) | 26.62(10.16,47.05) | 39.27(15.10,67.39) | 1.35(1.12,1.58) |
| Togo | both | 343.02(133.70,593.44) | 1525.97(599.79,2478.46) | 4.85(4.80,4.90) | 33.12(12.95,57.08) | 48.61(18.38,80.15) | 1.18(1.08,1.28) |
| Tokelau | both | 2.99(1.20,4.98) | 4.31(1.77,7.00) | 1.03(0.93,1.13) | 225.03(90.85,376.12) | 292.92(119.56,475.88) | 0.78(0.65,0.91) |
| Tonga | both | 157.32(66.14,251.10) | 296.80(127.50,468.24) | 1.98(1.86,2.10) | 273.89(113.39,444.16) | 364.33(155.94,571.48) | 0.90(0.81,0.99) |
| Trinidad and Tobago | both | 3738.63(1645.37,5701.86) | 8295.41(3763.44,13442.80) | 2.48(2.36,2.60) | 452.67(199.41,686.61) | 421.24(192.46,679.54) | -0.45(-0.58,-0.33) |
| Tunisia | both | 2024.82(797.80,3293.95) | 11665.81(4610.75,19545.64) | 5.79(5.67,5.91) | 44.94(17.86,72.72) | 90.78(36.23,150.16) | 2.35(2.23,2.46) |
| Türkiye | both | 37477.37(16172.27,58472.86) | 114383.79(47521.08,185863.26) | 3.99(3.69,4.29) | 120.09(51.69,186.97) | 125.18(51.88,202.88) | 0.45(0.14,0.76) |
| Turkmenistan | both | 412.88(167.47,668.77) | 1922.31(763.73,3236.16) | 4.70(4.42,4.98) | 22.69(9.27,36.81) | 48.73(19.72,81.74) | 2.09(1.84,2.34) |
| Tuvalu | both | 16.79(6.75,27.01) | 32.26(12.52,50.10) | 2.10(2.05,2.15) | 242.42(97.93,387.43) | 302.53(122.00,472.22) | 0.70(0.61,0.79) |
| Uganda | both | 2315.35(830.41,4261.51) | 6488.77(2189.98,11617.64) | 2.92(2.73,3.12) | 40.77(14.66,74.29) | 50.92(17.51,92.39) | 0.32(0.09,0.55) |
| Ukraine | both | 8403.41(3615.37,14156.50) | 14003.95(5293.24,23622.11) | 1.24(1.03,1.46) | 11.80(5.09,19.31) | 17.83(6.73,30.02) | 0.99(0.83,1.15) |
| United Arab Emirates | both | 666.35(268.31,1134.84) | 8142.96(3203.51,13061.99) | 8.76(8.55,8.97) | 158.65(64.39,266.41) | 196.01(79.37,305.22) | 1.88(1.39,2.37) |
| United Kingdom | both | 47903.84(21659.62,73524.26) | 87621.44(38721.31,139736.91) | 1.90(1.62,2.18) | 53.34(23.96,82.21) | 74.35(32.28,120.95) | 0.95(0.71,1.19) |
| United Republic of Tanzania | both | 1717.10(686.95,2909.33) | 4588.84(1767.55,8331.86) | 2.88(2.74,3.01) | 17.89(6.95,29.89) | 20.20(7.62,37.10) | 0.10(-0.01,0.20) |
| United States of America | both | 145621.09(60469.08,229748.13) | 447071.53(185554.08,710772.77) | 3.53(3.39,3.68) | 44.76(18.23,70.82) | 76.34(31.80,120.96) | 1.58(1.45,1.71) |
| United States Virgin Islands | both | 97.68(40.08,156.21) | 230.59(95.01,374.60) | 2.85(2.62,3.08) | 126.81(53.08,197.40) | 125.77(50.99,203.97) | -0.06(-0.17,0.06) |
| Uruguay | both | 1595.14(654.97,2575.98) | 3088.25(1323.08,5111.51) | 2.24(2.14,2.34) | 40.28(16.58,64.46) | 55.98(23.89,93.50) | 1.18(1.08,1.28) |
| Uzbekistan | both | 1662.93(657.82,2774.68) | 10023.84(4316.24,17078.54) | 5.77(5.54,6.00) | 14.83(5.85,24.70) | 38.18(16.49,63.48) | 3.00(2.67,3.33) |
| Vanuatu | both | 37.06(14.83,67.74) | 139.24(55.89,244.42) | 4.04(3.87,4.21) | 66.01(26.73,118.91) | 87.21(34.30,149.49) | 0.65(0.55,0.75) |
| Venezuela (Bolivarian Republic of) | both | 6663.92(2684.87,10813.30) | 29956.08(12870.36,49788.27) | 5.02(4.84,5.19) | 71.15(28.34,115.18) | 100.80(43.48,166.32) | 1.02(0.84,1.20) |
| Viet Nam | both | 17974.77(6998.12,31329.09) | 61363.93(23195.94,105329.92) | 4.19(4.01,4.37) | 46.91(18.56,81.34) | 65.83(25.64,111.74) | 1.29(1.16,1.42) |
| Yemen | both | 2987.60(1253.13,4879.88) | 13245.53(5634.29,21152.38) | 4.97(4.76,5.17) | 63.48(26.22,105.00) | 93.15(39.94,147.96) | 1.30(1.17,1.43) |
| Zambia | both | 1832.96(726.51,3053.13) | 4938.93(1887.64,8563.58) | 2.84(2.59,3.09) | 69.84(27.69,115.73) | 76.28(28.88,129.58) | -0.11(-0.27,0.06) |
| Zimbabwe | both | 2441.78(976.19,4090.27) | 7626.01(2929.98,12834.65) | 3.95(3.46,4.44) | 68.96(27.34,114.37) | 123.24(47.58,204.26) | 2.41(1.89,2.94) |
| Afghanistan | female | 6337.91(2607.21,10350.53) | 19627.62(8379.43,31596.28) | 3.63(3.48,3.78) | 186.96(75.77,301.00) | 348.49(153.61,570.80) | 2.18(2.12,2.24) |
| Albania | female | 285.55(113.84,475.90) | 855.51(357.59,1458.36) | 3.87(3.75,4.00) | 27.85(11.19,45.82) | 36.73(15.22,62.39) | 1.11(1.00,1.22) |
| Algeria | female | 5487.42(2312.68,9137.43) | 32523.75(14154.03,52801.12) | 6.06(5.93,6.18) | 98.10(40.25,158.93) | 189.40(80.37,305.68) | 2.50(2.35,2.65) |
| American Samoa | female | 49.61(22.09,77.13) | 194.83(85.33,309.93) | 4.31(3.83,4.80) | 418.53(188.10,652.28) | 781.77(345.03,1243.27) | 1.94(1.62,2.26) |
| Andorra | female | 10.12(4.34,16.44) | 35.58(15.14,57.58) | 3.97(3.72,4.23) | 38.10(16.42,61.88) | 44.20(18.57,72.72) | 0.61(0.52,0.70) |
| Angola | female | 1223.93(492.23,2140.60) | 5099.21(1868.50,8708.67) | 4.64(4.51,4.76) | 67.91(27.59,118.60) | 82.19(30.40,138.43) | 0.46(0.39,0.53) |
| Antigua and Barbuda | female | 67.29(29.13,105.95) | 143.09(56.21,235.07) | 2.19(2.04,2.34) | 217.50(93.96,346.31) | 254.92(100.07,415.95) | 0.15(-0.03,0.32) |
| Argentina | female | 9886.81(4085.83,16531.39) | 17794.13(7253.20,29798.75) | 1.84(1.66,2.02) | 54.83(22.79,90.99) | 54.70(22.09,92.49) | -0.09(-0.21,0.04) |
| Armenia | female | 721.55(300.04,1174.52) | 1336.23(550.32,2273.79) | 1.62(0.69,2.55) | 47.07(19.61,77.07) | 52.49(21.95,88.71) | -0.03(-0.83,0.77) |
| Australia | female | 5350.27(2337.44,8492.52) | 13447.09(5655.80,21460.09) | 2.96(2.84,3.08) | 48.35(21.30,77.54) | 55.48(23.29,88.03) | 0.41(0.31,0.52) |
| Austria | female | 3639.85(1538.29,5737.14) | 5078.43(2172.69,8181.90) | 1.69(1.35,2.03) | 44.58(18.61,70.76) | 43.56(18.17,71.17) | 0.33(0.02,0.64) |
| Azerbaijan | female | 917.47(361.54,1586.96) | 3395.53(1278.38,5946.45) | 3.98(3.79,4.18) | 31.47(12.45,54.43) | 61.06(23.21,105.76) | 1.96(1.62,2.31) |
| Bahamas | female | 174.51(73.74,281.42) | 432.89(187.01,726.56) | 2.83(2.67,2.98) | 202.59(85.94,325.88) | 192.82(83.90,318.05) | -0.33(-0.49,-0.18) |
| Bahrain | female | 253.71(100.03,398.97) | 1703.18(723.70,2765.25) | 6.19(6.05,6.33) | 357.14(146.62,562.17) | 516.70(223.79,827.29) | 0.89(0.56,1.23) |
| Bangladesh | female | 13832.44(5540.81,23005.82) | 74303.59(31200.51,122657.31) | 6.10(5.84,6.36) | 75.85(30.68,126.53) | 121.22(51.14,199.98) | 1.69(1.46,1.92) |
| Barbados | female | 621.02(278.16,973.15) | 997.46(454.27,1571.55) | 1.60(1.50,1.70) | 370.04(165.31,578.73) | 359.19(163.96,560.13) | -0.07(-0.21,0.07) |
| Belarus | female | 1461.58(584.80,2490.44) | 2379.51(1005.42,3974.75) | 0.97(0.67,1.27) | 17.39(7.06,29.40) | 23.10(9.68,39.01) | 0.31(0.04,0.59) |
| Belgium | female | 5030.04(2082.59,8177.32) | 6306.37(2773.84,10201.57) | 0.65(0.56,0.73) | 52.31(21.38,84.71) | 47.99(20.70,78.33) | -0.48(-0.58,-0.38) |
| Belize | female | 95.35(40.33,152.63) | 407.00(162.89,662.26) | 4.92(4.68,5.17) | 204.81(87.04,330.73) | 270.70(108.77,441.19) | 0.93(0.52,1.34) |
| Benin | female | 305.31(124.12,529.61) | 1245.60(461.67,2194.67) | 4.61(4.55,4.66) | 32.79(13.34,57.88) | 50.83(19.01,93.20) | 1.37(1.30,1.44) |
| Bermuda | female | 43.17(18.68,67.35) | 73.51(31.83,119.59) | 1.60(1.37,1.84) | 122.21(53.24,189.64) | 97.49(42.89,160.35) | -0.96(-1.18,-0.75) |
| Bhutan | female | 177.51(76.30,276.40) | 597.35(262.28,953.30) | 3.98(3.94,4.01) | 143.64(60.51,226.36) | 198.82(87.82,318.85) | 0.98(0.94,1.03) |
| Bolivia (Plurinational State of) | female | 1860.80(674.09,3255.47) | 6519.14(2586.42,11034.47) | 4.29(4.18,4.39) | 116.29(41.87,200.26) | 141.29(58.13,236.00) | 0.63(0.57,0.68) |
| Bosnia and Herzegovina | female | 1189.10(498.09,1963.93) | 3842.72(1584.07,6207.45) | 4.29(3.91,4.67) | 53.92(22.65,89.39) | 104.13(42.04,170.53) | 2.53(2.31,2.76) |
| Botswana | female | 542.66(235.82,921.76) | 1752.07(719.86,2773.48) | 4.46(3.96,4.97) | 186.34(80.91,311.80) | 223.40(92.46,351.21) | 1.26(0.76,1.78) |
| Brazil | female | 67958.08(28582.71,106766.70) | 193772.45(79967.74,311807.78) | 3.40(3.31,3.49) | 148.36(62.52,231.69) | 139.33(57.65,224.52) | -0.28(-0.36,-0.20) |
| Brunei Darussalam | female | 113.02(46.64,183.82) | 364.08(152.98,604.94) | 3.84(3.73,3.94) | 246.20(100.35,392.28) | 212.66(85.98,353.58) | -0.54(-0.62,-0.46) |
| Bulgaria | female | 3402.63(1405.51,5517.71) | 5617.41(2344.17,9520.43) | 1.46(1.20,1.71) | 51.11(20.46,80.88) | 66.31(28.13,111.43) | 0.65(0.46,0.84) |
| Burkina Faso | female | 1100.90(453.70,1834.29) | 2762.09(1073.52,4760.30) | 2.99(2.86,3.12) | 55.10(21.92,92.45) | 61.40(23.90,103.60) | 0.29(0.22,0.35) |
| Burundi | female | 727.31(263.86,1319.94) | 1205.19(446.01,2127.24) | 1.10(0.77,1.44) | 61.56(22.37,108.98) | 57.68(21.12,101.13) | -0.72(-0.92,-0.53) |
| Cabo Verde | female | 42.64(17.53,69.43) | 210.55(88.60,373.87) | 5.20(4.83,5.56) | 32.14(13.08,52.45) | 82.57(34.51,145.48) | 2.97(2.61,3.33) |
| Cambodia | female | 711.52(266.02,1267.79) | 2724.08(968.79,5033.19) | 4.37(4.03,4.70) | 31.54(11.52,58.52) | 42.67(15.02,80.30) | 0.83(0.58,1.08) |
| Cameroon | female | 1806.70(742.60,3167.39) | 7397.84(3048.76,12540.19) | 4.67(4.54,4.79) | 87.31(36.13,151.78) | 121.94(51.78,207.98) | 1.09(0.93,1.25) |
| Canada | female | 4305.70(1594.61,7556.12) | 11656.01(4245.21,20743.20) | 2.79(2.49,3.08) | 22.13(8.20,38.51) | 27.93(10.00,49.63) | 0.26(0.02,0.51) |
| Central African Republic | female | 603.29(244.10,992.82) | 1440.90(526.92,2569.21) | 2.79(2.73,2.85) | 100.74(41.95,166.95) | 117.46(45.20,215.60) | 0.45(0.35,0.56) |
| Chad | female | 718.99(275.10,1211.25) | 2172.22(873.55,3690.54) | 3.57(3.46,3.69) | 52.19(19.56,90.60) | 86.35(34.60,147.30) | 1.65(1.43,1.87) |
| Chile | female | 2684.47(1107.47,4406.54) | 8470.01(3601.91,14134.56) | 3.80(3.59,4.01) | 49.34(20.31,81.62) | 59.02(25.07,98.94) | 0.57(0.35,0.79) |
| China | female | 204315.02(83016.88,328405.82) | 570448.71(236149.50,908558.84) | 3.22(3.12,3.32) | 49.87(20.59,79.98) | 52.33(21.53,82.65) | -0.02(-0.16,0.12) |
| Colombia | female | 10217.40(4190.64,16292.69) | 32090.26(13888.97,51950.62) | 3.38(3.25,3.51) | 109.64(44.24,175.07) | 106.37(45.97,172.40) | -0.57(-0.76,-0.38) |
| Comoros | female | 50.52(18.92,92.20) | 166.47(63.48,300.74) | 3.97(3.85,4.09) | 54.40(21.02,99.55) | 65.41(24.76,119.84) | 0.60(0.50,0.69) |
| Congo | female | 486.45(194.09,852.45) | 1411.75(528.03,2400.08) | 3.29(3.12,3.45) | 85.57(32.22,147.91) | 101.61(37.52,174.54) | 0.41(0.30,0.53) |
| Cook Islands | female | 27.83(10.67,45.05) | 54.14(23.39,87.10) | 2.09(2.02,2.16) | 459.05(174.81,744.67) | 415.43(179.31,665.71) | -0.45(-0.51,-0.38) |
| Costa Rica | female | 577.76(218.95,963.05) | 2582.04(1000.52,4311.23) | 4.61(4.38,4.83) | 64.40(24.15,109.36) | 86.18(33.40,144.26) | 0.58(0.33,0.82) |
| Croatia | female | 1833.41(771.23,3011.50) | 3892.36(1764.08,6455.77) | 2.14(1.93,2.34) | 51.48(21.63,84.33) | 68.17(30.13,111.46) | 0.51(0.29,0.73) |
| Cuba | female | 5843.02(2337.30,9214.00) | 10190.93(4212.35,16682.29) | 1.83(1.53,2.12) | 112.87(44.99,178.82) | 99.59(40.73,165.43) | -0.42(-0.73,-0.11) |
| Cyprus | female | 708.82(306.47,1130.04) | 940.55(395.27,1533.33) | 0.53(0.37,0.69) | 187.00(75.79,298.81) | 89.20(37.78,146.38) | -2.88(-3.10,-2.66) |
| Czechia | female | 4788.19(1964.50,7698.95) | 10711.49(4802.14,17235.80) | 3.64(3.16,4.12) | 55.41(22.88,89.95) | 81.42(35.84,130.84) | 2.12(1.74,2.51) |
| Côte d'Ivoire | female | 965.90(382.50,1634.14) | 4738.01(1919.86,8237.80) | 5.44(5.19,5.68) | 59.51(24.01,100.39) | 94.66(37.54,167.51) | 1.79(1.60,1.98) |
| Democratic People's Republic of Korea | female | 5377.02(2071.20,9171.50) | 13874.61(5696.03,23264.68) | 3.14(2.96,3.31) | 57.54(22.19,97.46) | 71.87(29.04,121.10) | 0.77(0.66,0.87) |
| Democratic Republic of the Congo | female | 7323.61(2867.63,12384.05) | 21003.71(8060.13,36485.13) | 3.27(3.19,3.36) | 99.16(38.27,164.84) | 114.86(44.47,196.49) | 0.45(0.39,0.51) |
| Denmark | female | 1377.33(591.37,2195.39) | 2613.75(1149.06,4265.22) | 1.94(1.68,2.21) | 28.01(11.86,44.57) | 37.58(16.29,61.68) | 0.84(0.58,1.10) |
| Djibouti | female | 25.41(8.97,48.34) | 160.44(57.40,284.44) | 6.15(6.07,6.24) | 44.91(15.21,84.14) | 63.21(22.73,114.86) | 1.13(1.04,1.22) |
| Dominica | female | 75.96(31.01,123.27) | 111.35(46.87,187.87) | 1.03(0.95,1.10) | 208.85(85.28,342.66) | 250.41(105.74,424.86) | 0.38(0.31,0.45) |
| Dominican Republic | female | 1758.25(731.48,2884.22) | 7537.18(3193.28,12296.06) | 4.97(4.83,5.11) | 97.76(41.12,160.93) | 145.20(61.71,236.62) | 1.57(1.42,1.72) |
| Ecuador | female | 1430.58(541.76,2434.86) | 6677.37(2581.08,11734.92) | 5.05(4.58,5.52) | 54.66(20.51,94.08) | 77.94(30.09,137.65) | 1.03(0.60,1.46) |
| Egypt | female | 19324.35(7781.94,29577.76) | 80842.31(35718.72,133800.89) | 5.01(4.88,5.15) | 164.47(67.19,249.03) | 318.04(138.81,516.20) | 2.75(2.49,3.01) |
| El Salvador | female | 1054.82(405.88,1706.56) | 4952.17(1991.67,8462.80) | 5.04(4.83,5.26) | 66.77(25.67,108.38) | 137.99(55.65,236.43) | 2.29(2.10,2.47) |
| Equatorial Guinea | female | 80.66(31.40,136.39) | 332.19(110.98,598.71) | 5.05(4.80,5.31) | 81.02(32.18,138.46) | 118.62(41.20,213.48) | 1.50(1.34,1.66) |
| Eritrea | female | 171.41(63.56,319.37) | 552.82(213.37,1056.97) | 4.43(4.22,4.63) | 30.81(10.56,57.48) | 38.76(14.91,72.42) | 0.92(0.84,1.00) |
| Estonia | female | 247.70(100.56,424.25) | 793.05(314.20,1343.95) | 3.75(3.45,4.05) | 18.04(7.27,30.80) | 42.59(16.50,72.97) | 2.59(2.33,2.85) |
| Eswatini | female | 255.92(106.43,425.94) | 907.29(351.57,1578.17) | 4.53(3.75,5.31) | 171.10(69.86,286.72) | 282.97(110.57,491.08) | 2.05(1.36,2.75) |
| Ethiopia | female | 9093.30(3322.71,15452.71) | 13742.85(5447.49,22391.43) | 0.83(0.50,1.15) | 101.24(37.87,170.55) | 66.59(26.89,112.58) | -1.87(-2.09,-1.66) |
| Fiji | female | 1073.75(441.79,1838.77) | 3430.60(1482.91,5751.53) | 3.81(3.52,4.11) | 545.08(222.22,939.45) | 812.15(351.67,1381.63) | 1.17(0.90,1.43) |
| Finland | female | 1462.34(622.41,2353.82) | 2636.09(1119.55,4451.68) | 1.85(1.59,2.12) | 31.86(13.19,50.75) | 38.63(16.05,66.47) | 0.52(0.28,0.77) |
| France | female | 17443.55(7414.81,27640.30) | 28435.26(12390.96,45439.58) | 1.67(1.39,1.97) | 32.22(13.61,50.70) | 31.91(14.06,51.15) | -0.03(-0.34,0.28) |
| Gabon | female | 194.99(68.01,348.72) | 465.78(185.05,800.99) | 2.65(2.44,2.86) | 66.32(23.06,122.06) | 90.26(36.19,156.36) | 0.87(0.60,1.14) |
| Gambia | female | 79.11(28.96,139.28) | 442.27(178.20,749.92) | 5.66(5.37,5.96) | 51.69(19.09,91.53) | 90.73(37.35,154.51) | 1.73(1.58,1.88) |
| Georgia | female | 1090.92(452.28,1823.54) | 2051.15(831.79,3421.70) | 2.67(2.24,3.11) | 28.24(11.71,46.95) | 55.34(22.41,93.45) | 2.83(2.41,3.25) |
| Germany | female | 38480.78(16352.71,61084.39) | 45762.52(19705.60,76233.27) | 0.35(0.12,0.59) | 43.16(18.05,68.80) | 37.50(16.06,61.82) | -0.79(-1.04,-0.53) |
| Ghana | female | 2203.56(861.30,3712.69) | 9987.87(4042.21,16582.25) | 5.17(4.97,5.37) | 74.03(29.55,125.52) | 114.23(47.12,191.10) | 1.56(1.44,1.69) |
| Greece | female | 3982.38(1751.52,6366.07) | 6901.60(2828.25,11359.18) | 1.62(1.30,1.94) | 47.29(20.77,75.91) | 47.57(19.97,78.88) | -0.25(-0.55,0.05) |
| Greenland | female | 5.27(2.20,8.49) | 9.78(3.88,16.37) | 1.71(1.40,2.02) | 35.28(14.98,57.09) | 32.06(13.17,53.57) | -0.62(-0.89,-0.36) |
| Grenada | female | 126.86(51.61,212.52) | 184.37(78.29,296.25) | 1.72(1.52,1.92) | 295.15(120.55,490.83) | 307.90(131.05,496.73) | 0.36(0.19,0.54) |
| Guam | female | 51.37(21.73,82.16) | 105.81(43.24,181.43) | 2.25(2.09,2.41) | 150.28(63.41,238.08) | 96.58(39.75,164.10) | -1.40(-1.71,-1.09) |
| Guatemala | female | 676.01(261.79,1196.17) | 6703.74(2541.45,11562.22) | 7.49(7.18,7.80) | 44.28(17.33,76.82) | 115.73(43.66,197.59) | 2.71(2.41,3.01) |
| Guinea | female | 783.37(293.87,1349.44) | 1887.99(738.11,3299.59) | 2.64(2.36,2.92) | 50.66(19.10,87.73) | 72.23(27.75,124.05) | 1.15(1.06,1.24) |
| Guinea-Bissau | female | 169.63(65.64,289.49) | 505.23(188.47,852.57) | 3.57(3.48,3.66) | 89.37(34.63,152.98) | 136.59(52.01,235.70) | 1.41(1.26,1.56) |
| Guyana | female | 618.41(248.16,1019.62) | 1350.79(535.01,2196.74) | 2.47(2.17,2.77) | 315.43(127.31,531.96) | 389.72(158.62,630.98) | 0.47(0.09,0.86) |
| Haiti | female | 5802.09(2253.58,9657.86) | 14446.15(5617.89,25000.99) | 3.05(2.98,3.12) | 352.61(136.13,584.51) | 385.21(152.87,666.56) | 0.38(0.32,0.44) |
| Honduras | female | 588.78(219.80,1032.47) | 3850.99(1541.30,6815.81) | 6.32(6.16,6.49) | 55.81(21.35,98.87) | 112.95(44.68,197.37) | 2.32(2.11,2.53) |
| Hungary | female | 4452.63(1919.48,7171.87) | 7576.94(3089.59,12776.94) | 1.98(1.75,2.21) | 49.33(21.36,81.34) | 61.81(25.39,105.21) | 0.88(0.68,1.08) |
| Iceland | female | 38.39(15.62,61.21) | 102.72(41.80,162.37) | 3.24(3.15,3.32) | 23.67(9.77,37.74) | 34.22(14.08,53.34) | 1.20(1.13,1.26) |
| India | female | 143566.86(60732.54,221973.51) | 582863.13(260883.26,920764.79) | 4.69(4.52,4.85) | 72.37(30.27,112.79) | 100.08(44.85,157.65) | 1.03(0.84,1.22) |
| Indonesia | female | 61700.42(25789.22,95105.66) | 216373.13(93245.73,339151.40) | 3.80(3.68,3.92) | 125.51(52.63,194.29) | 181.11(78.64,281.32) | 1.08(1.01,1.15) |
| Iran (Islamic Republic of) | female | 9612.66(4416.74,15208.19) | 56279.97(24018.90,88935.84) | 6.26(6.10,6.42) | 82.02(37.08,129.43) | 144.48(62.11,227.65) | 2.09(1.98,2.20) |
| Iraq | female | 9306.47(4040.65,14745.57) | 40238.48(16524.07,62887.40) | 4.88(4.76,5.01) | 224.39(97.44,357.10) | 314.93(130.34,492.91) | 1.02(0.94,1.10) |
| Ireland | female | 1069.96(468.61,1642.06) | 1721.92(749.23,2722.26) | 1.74(1.56,1.92) | 45.98(20.00,70.24) | 40.77(17.67,65.26) | -0.24(-0.37,-0.12) |
| Israel | female | 2013.63(846.06,3163.98) | 5007.23(2130.53,8275.37) | 2.58(1.93,3.24) | 77.05(32.03,122.50) | 69.60(28.83,114.81) | -0.66(-1.26,-0.05) |
| Italy | female | 42303.70(18301.83,64689.02) | 50291.02(21695.76,81835.44) | 0.77(0.66,0.87) | 78.25(33.70,118.32) | 56.49(24.12,91.72) | -0.94(-1.03,-0.86) |
| Jamaica | female | 3339.24(1455.91,5129.77) | 6121.40(2695.79,9534.98) | 1.60(1.38,1.82) | 348.83(154.09,538.53) | 375.55(166.58,581.75) | -0.15(-0.36,0.07) |
| Japan | female | 45447.39(19900.92,72752.89) | 96786.48(39266.75,161800.55) | 2.49(2.39,2.58) | 47.83(20.96,76.29) | 53.19(21.52,87.21) | 0.34(0.21,0.46) |
| Jordan | female | 1529.88(628.33,2462.40) | 7713.82(3226.02,12774.96) | 4.90(4.65,5.15) | 256.63(106.82,411.33) | 231.28(97.73,379.33) | -0.73(-1.20,-0.25) |
| Kazakhstan | female | 2608.93(1060.10,4291.58) | 5999.22(2447.07,10243.56) | 2.12(1.82,2.42) | 33.20(13.48,55.21) | 56.11(23.18,94.70) | 1.16(0.92,1.41) |
| Kenya | female | 2188.00(842.13,3522.02) | 8518.08(3372.26,14527.63) | 4.84(4.70,4.97) | 53.73(21.84,87.63) | 71.28(27.35,121.51) | 1.31(1.14,1.48) |
| Kiribati | female | 126.32(54.34,200.52) | 370.78(157.53,608.02) | 3.74(3.62,3.85) | 584.41(252.07,919.92) | 839.46(349.19,1394.15) | 1.23(1.09,1.37) |
| Kuwait | female | 556.78(244.05,858.23) | 4425.59(1961.89,7200.91) | 7.20(6.81,7.58) | 219.79(96.77,343.14) | 284.63(129.47,457.50) | 0.99(0.78,1.20) |
| Kyrgyzstan | female | 377.16(166.12,636.38) | 1019.17(399.21,1741.83) | 2.63(2.38,2.89) | 21.10(9.25,35.31) | 37.74(15.08,64.41) | 1.43(1.18,1.68) |
| Lao People's Democratic Republic | female | 685.84(252.25,1192.24) | 1734.75(647.18,2957.45) | 2.70(2.50,2.91) | 65.68(24.38,112.79) | 77.13(29.55,133.31) | 0.28(0.18,0.39) |
| Latvia | female | 540.61(221.89,897.94) | 1272.84(538.20,2070.90) | 2.79(2.58,3.00) | 23.57(9.84,39.07) | 51.10(21.54,84.04) | 2.44(2.20,2.67) |
| Lebanon | female | 1374.70(556.80,2287.14) | 4599.21(1882.06,7450.45) | 4.14(4.05,4.22) | 126.60(51.59,208.41) | 139.30(56.92,224.66) | 0.35(0.30,0.40) |
| Lesotho | female | 265.52(105.38,469.90) | 727.40(277.12,1308.51) | 4.01(3.33,4.68) | 53.16(21.66,94.13) | 123.27(47.83,217.91) | 3.70(3.10,4.30) |
| Liberia | female | 630.00(254.34,1032.42) | 2066.10(848.84,3442.90) | 4.11(3.95,4.27) | 120.52(49.58,195.84) | 188.27(76.24,312.23) | 1.62(1.46,1.78) |
| Libya | female | 828.91(376.60,1335.89) | 5427.27(2384.30,8842.36) | 6.85(6.66,7.03) | 93.42(41.78,150.88) | 199.06(87.11,325.53) | 3.06(2.82,3.29) |
| Lithuania | female | 563.83(220.65,936.55) | 1610.00(662.54,2640.06) | 3.22(2.97,3.48) | 20.00(7.91,33.19) | 43.23(17.59,70.73) | 2.26(1.95,2.56) |
| Luxembourg | female | 147.19(58.69,231.54) | 231.89(101.35,371.97) | 1.43(1.28,1.57) | 43.89(17.76,68.75) | 39.12(17.04,63.76) | -0.43(-0.52,-0.35) |
| Madagascar | female | 1063.81(367.72,1985.29) | 2724.00(1061.38,4932.15) | 3.03(2.81,3.25) | 46.19(16.99,82.19) | 52.17(21.11,92.11) | 0.33(0.26,0.39) |
| Malawi | female | 611.43(222.41,1142.41) | 1308.15(477.01,2438.90) | 2.16(1.99,2.34) | 32.97(11.73,60.11) | 35.30(13.28,64.49) | -0.02(-0.17,0.13) |
| Malaysia | female | 5558.77(2402.85,9070.30) | 18773.63(7800.34,30338.12) | 3.94(3.77,4.12) | 120.07(52.23,194.95) | 133.38(55.21,213.67) | 0.23(0.07,0.38) |
| Maldives | female | 109.98(47.26,166.96) | 260.92(117.65,413.09) | 2.47(2.12,2.83) | 276.57(120.69,420.61) | 173.56(78.27,271.85) | -1.89(-2.21,-1.58) |
| Mali | female | 1731.90(711.83,2869.10) | 5741.93(2222.34,9747.50) | 4.16(4.08,4.25) | 93.89(38.72,155.79) | 140.33(55.07,241.15) | 1.35(1.24,1.46) |
| Malta | female | 223.95(98.82,345.02) | 448.49(198.40,712.36) | 2.26(1.98,2.54) | 93.62(41.42,144.56) | 83.99(36.32,132.96) | -0.38(-0.65,-0.10) |
| Marshall Islands | female | 62.19(28.15,94.93) | 281.23(117.11,461.10) | 5.01(4.72,5.31) | 715.49(324.52,1099.05) | 1415.06(578.58,2297.34) | 2.09(1.72,2.46) |
| Mauritania | female | 602.61(247.31,979.44) | 1705.11(734.10,2857.75) | 3.25(3.15,3.36) | 118.79(47.84,193.06) | 160.21(69.29,268.17) | 0.78(0.71,0.85) |
| Mauritius | female | 535.97(211.72,879.88) | 2770.52(1086.13,4519.72) | 6.34(5.67,7.02) | 135.31(52.74,220.22) | 271.99(108.48,442.34) | 3.07(2.40,3.74) |
| Mexico | female | 41773.31(16834.19,65068.67) | 128756.72(53797.90,210091.77) | 3.61(3.34,3.88) | 200.16(78.93,313.57) | 190.29(78.16,310.75) | -0.29(-0.55,-0.04) |
| Micronesia (Federated States of) | female | 127.78(55.96,202.57) | 329.13(144.28,510.40) | 3.16(2.93,3.40) | 503.49(219.83,796.28) | 790.69(340.36,1213.23) | 1.51(1.23,1.80) |
| Monaco | female | 9.67(4.03,15.79) | 18.87(7.70,31.02) | 2.13(2.09,2.18) | 22.26(9.62,35.93) | 35.54(14.69,58.54) | 1.56(1.53,1.59) |
| Mongolia | female | 85.79(33.74,153.98) | 288.21(112.10,500.82) | 3.85(3.47,4.22) | 15.17(5.97,27.02) | 22.32(8.55,38.40) | 1.09(0.97,1.21) |
| Montenegro | female | 182.20(75.21,292.21) | 446.38(186.33,728.71) | 2.96(2.74,3.18) | 52.50(21.37,84.15) | 80.72(33.32,132.60) | 1.42(1.30,1.54) |
| Morocco | female | 7368.29(3054.23,11908.95) | 43056.60(18185.03,69262.79) | 6.15(6.04,6.26) | 103.25(43.33,165.83) | 241.80(101.87,386.70) | 3.04(2.93,3.14) |
| Mozambique | female | 1133.46(449.70,1958.51) | 2919.00(1076.48,5194.92) | 3.47(3.27,3.66) | 40.42(16.43,70.02) | 52.24(19.23,93.97) | 1.20(1.01,1.39) |
| Myanmar | female | 6509.91(2337.86,11984.85) | 18095.73(6903.90,32886.08) | 3.07(2.81,3.34) | 55.22(20.50,99.53) | 67.94(26.03,119.81) | 0.48(0.31,0.64) |
| Namibia | female | 476.61(189.30,798.13) | 1281.31(500.34,2163.36) | 2.84(2.52,3.15) | 142.04(55.44,234.84) | 171.00(65.40,285.64) | 0.33(0.04,0.63) |
| Nauru | female | 9.04(3.53,14.80) | 19.92(7.54,33.10) | 2.33(2.06,2.61) | 407.87(159.54,664.37) | 579.22(222.65,945.70) | 1.04(0.86,1.21) |
| Nepal | female | 2040.07(827.21,3486.98) | 9173.00(3429.82,16122.20) | 5.14(4.82,5.47) | 49.55(20.03,85.57) | 78.84(30.69,135.96) | 1.53(1.29,1.78) |
| Netherlands | female | 5393.91(2157.07,8627.54) | 5901.36(2412.59,9864.81) | 0.11(-0.10,0.32) | 42.45(16.96,67.78) | 28.44(11.79,47.45) | -1.49(-1.70,-1.29) |
| New Zealand | female | 960.72(410.14,1484.46) | 2412.47(1111.44,3809.55) | 2.90(2.77,3.02) | 43.09(18.25,66.54) | 53.76(24.60,85.55) | 0.70(0.60,0.80) |
| Nicaragua | female | 627.52(256.37,1046.71) | 3151.51(1198.10,5545.00) | 5.28(5.11,5.46) | 77.04(31.53,130.02) | 117.00(44.15,204.47) | 1.32(1.16,1.48) |
| Niger | female | 690.93(258.72,1208.82) | 3268.68(1272.76,5583.13) | 5.12(5.05,5.18) | 55.34(20.82,93.85) | 81.34(31.52,137.46) | 1.18(1.09,1.27) |
| Nigeria | female | 16214.76(7082.31,26193.58) | 42100.41(16727.56,70423.30) | 3.00(2.74,3.26) | 79.74(34.99,127.68) | 91.77(35.96,152.10) | 0.37(0.33,0.41) |
| Niue | female | 3.57(1.47,5.80) | 5.70(2.41,9.46) | 1.28(1.18,1.38) | 292.16(122.27,475.60) | 492.59(208.03,818.16) | 1.57(1.44,1.70) |
| North Macedonia | female | 709.31(299.66,1148.34) | 2062.50(889.40,3378.77) | 3.65(3.31,3.98) | 76.14(32.51,123.75) | 119.84(51.56,197.65) | 1.53(1.23,1.83) |
| Northern Mariana Islands | female | 21.58(9.20,35.32) | 71.28(30.76,113.93) | 4.08(3.72,4.45) | 274.12(115.41,443.53) | 269.90(113.88,426.00) | -0.10(-0.21,0.02) |
| Norway | female | 1250.22(495.68,2042.34) | 1810.42(735.32,2930.49) | 1.05(0.87,1.23) | 30.20(12.13,49.23) | 32.40(13.13,51.93) | 0.12(-0.03,0.27) |
| Oman | female | 531.56(225.32,862.04) | 1859.07(787.11,2927.07) | 4.00(3.66,4.33) | 180.30(75.79,292.02) | 211.06(89.37,328.02) | 0.57(0.43,0.70) |
| Pakistan | female | 19310.72(8091.55,32085.46) | 82759.36(33986.74,133259.69) | 4.61(4.46,4.76) | 82.62(34.32,136.66) | 152.30(62.45,242.01) | 1.84(1.58,2.10) |
| Palau | female | 15.39(6.34,24.94) | 49.24(21.27,84.41) | 3.65(3.50,3.80) | 304.93(126.12,493.31) | 457.10(188.36,781.00) | 1.44(1.33,1.54) |
| Palestine | female | 999.47(418.46,1568.30) | 3585.37(1554.36,5593.56) | 4.35(4.24,4.46) | 218.16(92.56,342.62) | 278.48(120.48,433.36) | 0.93(0.78,1.07) |
| Panama | female | 514.98(230.36,841.64) | 2348.75(950.48,3997.95) | 4.91(4.74,5.08) | 70.32(31.04,115.12) | 102.01(40.98,174.55) | 1.07(0.91,1.24) |
| Papua New Guinea | female | 1992.19(803.14,3507.66) | 7587.80(3020.82,12707.61) | 4.43(4.29,4.57) | 204.10(83.73,358.79) | 269.86(106.57,449.33) | 0.88(0.83,0.93) |
| Paraguay | female | 809.78(291.06,1430.73) | 3865.10(1524.68,6623.08) | 5.32(5.04,5.61) | 71.51(25.81,126.78) | 128.12(50.10,219.27) | 2.07(1.80,2.35) |
| Peru | female | 2904.60(1174.44,4925.35) | 11767.48(4854.77,19987.79) | 4.44(4.21,4.66) | 49.91(20.44,85.33) | 67.71(27.81,114.92) | 0.68(0.46,0.90) |
| Philippines | female | 8274.92(3337.74,13953.34) | 36258.72(14410.85,59401.14) | 5.08(5.00,5.16) | 62.53(24.39,106.87) | 85.59(34.53,141.30) | 1.21(1.14,1.28) |
| Poland | female | 17797.24(7605.22,29270.90) | 30892.88(13383.62,50231.46) | 1.97(1.76,2.19) | 67.58(28.83,109.77) | 71.04(30.78,115.05) | 0.24(0.07,0.41) |
| Portugal | female | 6542.94(2825.86,10094.95) | 11710.25(4995.20,18666.28) | 1.65(1.40,1.91) | 79.95(34.77,122.83) | 78.12(33.13,126.60) | -0.42(-0.64,-0.19) |
| Puerto Rico | female | 3997.16(1681.61,6201.23) | 7284.58(3139.02,12123.66) | 1.75(1.44,2.05) | 206.26(87.09,321.12) | 186.79(77.98,314.01) | -0.52(-0.71,-0.33) |
| Qatar | female | 109.06(47.61,173.12) | 1408.19(605.97,2289.77) | 8.79(8.58,9.00) | 332.63(142.42,528.46) | 456.56(196.96,738.08) | 1.05(0.32,1.79) |
| Republic of Korea | female | 12253.95(5199.25,19726.81) | 45905.37(18490.25,77473.42) | 4.11(3.91,4.32) | 73.63(31.85,116.87) | 90.86(37.71,152.92) | 0.29(0.05,0.52) |
| Republic of Moldova | female | 724.42(296.30,1223.84) | 1598.27(616.58,2678.39) | 2.15(1.83,2.47) | 27.97(11.27,46.70) | 44.44(17.58,75.60) | 1.15(0.87,1.42) |
| Romania | female | 6116.26(2618.69,9976.00) | 10445.33(4157.83,17097.20) | 1.94(1.80,2.08) | 39.66(16.87,64.27) | 48.60(19.41,79.50) | 0.81(0.67,0.96) |
| Russian Federation | female | 24362.14(9910.12,39655.60) | 85607.58(34041.40,134480.01) | 3.64(2.95,4.33) | 20.38(8.31,32.64) | 53.55(21.32,84.94) | 2.73(2.12,3.34) |
| Rwanda | female | 860.42(309.71,1646.50) | 1489.09(505.25,2985.81) | 1.28(0.74,1.83) | 60.24(20.82,112.84) | 46.10(16.19,89.68) | -1.69(-2.03,-1.34) |
| Saint Kitts and Nevis | female | 60.69(26.14,96.91) | 82.68(34.82,133.45) | 1.45(1.10,1.79) | 282.16(122.24,452.31) | 228.79(97.67,362.39) | -0.32(-0.52,-0.12) |
| Saint Lucia | female | 165.94(69.66,260.89) | 338.24(138.82,541.83) | 1.69(1.40,1.97) | 345.31(147.41,541.85) | 265.57(108.65,424.39) | -1.65(-1.92,-1.38) |
| Saint Vincent and the Grenadines | female | 142.83(60.35,232.07) | 213.88(97.02,345.10) | 1.08(0.83,1.33) | 359.82(152.07,581.66) | 305.31(138.64,491.95) | -0.85(-1.09,-0.62) |
| Samoa | female | 170.27(76.93,271.63) | 509.66(228.63,810.01) | 3.63(3.52,3.74) | 381.71(173.36,605.29) | 671.51(299.89,1069.42) | 1.86(1.77,1.94) |
| San Marino | female | 6.42(2.70,10.34) | 14.46(5.72,24.35) | 2.75(2.60,2.89) | 31.12(13.18,50.33) | 36.51(14.68,60.77) | 0.68(0.57,0.80) |
| Sao Tome and Principe | female | 13.38(5.25,22.90) | 35.09(13.82,61.72) | 3.00(2.91,3.09) | 39.14(15.34,66.75) | 61.85(24.56,108.63) | 1.34(1.19,1.49) |
| Saudi Arabia | female | 3763.69(1539.34,5978.67) | 23118.18(9268.46,37685.14) | 5.68(5.52,5.85) | 155.87(64.84,245.80) | 253.83(105.90,401.12) | 1.42(1.30,1.54) |
| Senegal | female | 1393.85(583.91,2342.30) | 5609.53(2290.14,9568.40) | 4.67(4.51,4.84) | 92.11(38.08,155.35) | 144.37(59.67,251.49) | 1.46(1.33,1.59) |
| Serbia | female | 3998.55(1650.95,6389.46) | 8561.72(3587.32,14150.65) | 2.30(2.06,2.54) | 71.30(28.58,113.82) | 88.35(36.94,144.52) | 0.48(0.34,0.61) |
| Seychelles | female | 23.20(10.01,37.66) | 88.03(34.77,146.47) | 4.59(4.48,4.70) | 70.28(30.17,113.57) | 148.58(59.16,245.34) | 2.67(2.52,2.83) |
| Sierra Leone | female | 521.11(223.10,839.80) | 1739.86(684.28,2850.43) | 4.12(4.03,4.21) | 55.96(24.14,90.01) | 96.57(38.44,159.35) | 1.98(1.74,2.21) |
| Singapore | female | 962.14(383.30,1587.76) | 2301.97(991.61,3836.64) | 2.99(2.86,3.13) | 80.55(32.36,132.42) | 52.61(22.62,87.38) | -1.27(-1.45,-1.08) |
| Slovakia | female | 1423.47(608.91,2393.30) | 2458.97(990.14,4221.39) | 1.80(1.74,1.87) | 39.82(17.05,65.95) | 43.14(17.18,73.24) | 0.27(0.21,0.32) |
| Slovenia | female | 680.21(285.85,1132.68) | 1151.66(467.11,1881.33) | 0.92(0.61,1.23) | 44.03(18.34,73.26) | 43.70(17.71,71.54) | -0.89(-1.18,-0.59) |
| Solomon Islands | female | 194.41(76.96,331.67) | 796.31(316.86,1301.36) | 4.59(4.44,4.75) | 290.95(113.09,482.09) | 407.98(160.00,670.55) | 1.10(0.95,1.26) |
| Somalia | female | 674.54(252.77,1256.42) | 2248.15(816.18,4159.66) | 4.11(3.98,4.24) | 60.62(23.01,108.84) | 71.89(26.02,129.18) | 0.62(0.52,0.72) |
| South Africa | female | 21295.41(9445.18,32759.77) | 81213.25(34258.57,126488.44) | 4.57(4.20,4.93) | 181.50(80.77,278.91) | 309.10(131.21,479.78) | 1.98(1.64,2.31) |
| South Sudan | female | 498.56(186.87,883.02) | 1163.76(399.66,2180.79) | 2.74(2.37,3.11) | 51.01(18.71,88.25) | 70.30(23.95,126.82) | 1.00(0.83,1.16) |
| Spain | female | 23769.76(9771.90,37967.19) | 35719.07(14919.20,58811.02) | 1.24(1.12,1.36) | 72.07(29.75,115.38) | 62.12(25.93,101.40) | -0.61(-0.69,-0.54) |
| Sri Lanka | female | 2994.97(1112.33,5072.31) | 16480.42(6355.57,27913.45) | 6.06(5.88,6.25) | 62.48(22.91,108.87) | 108.16(41.87,179.97) | 2.19(2.01,2.37) |
| Sudan | female | 6087.30(2720.77,9506.80) | 23665.41(10740.16,36884.73) | 4.46(4.31,4.62) | 131.76(58.76,206.87) | 229.13(103.74,357.89) | 1.93(1.87,2.00) |
| Suriname | female | 246.20(106.37,397.11) | 843.26(354.08,1329.85) | 4.48(4.27,4.69) | 185.51(80.13,296.99) | 244.86(102.94,385.39) | 1.13(0.97,1.29) |
| Sweden | female | 2592.77(1126.86,4233.17) | 4188.45(1830.14,6926.11) | 1.62(1.51,1.73) | 28.33(12.23,46.33) | 35.04(15.13,56.75) | 0.79(0.69,0.89) |
| Switzerland | female | 3362.46(1392.28,5309.75) | 4902.84(1995.06,7900.42) | 1.04(0.88,1.21) | 50.98(21.22,79.29) | 49.16(19.77,79.32) | -0.26(-0.42,-0.11) |
| Syrian Arab Republic | female | 3055.07(1247.91,4965.30) | 10750.72(4674.52,17240.00) | 3.94(3.76,4.13) | 125.48(52.16,202.34) | 170.97(75.82,276.54) | 0.62(0.44,0.80) |
| Taiwan (Province of China) | female | 11292.69(4758.49,18231.56) | 25008.33(10676.53,40925.47) | 1.92(1.60,2.24) | 156.22(67.02,250.40) | 108.98(46.55,178.29) | -1.90(-2.23,-1.57) |
| Tajikistan | female | 401.44(162.04,664.24) | 1292.56(474.05,2323.77) | 3.56(3.34,3.78) | 26.54(10.77,43.67) | 44.68(17.39,79.84) | 1.55(1.31,1.80) |
| Thailand | female | 13512.79(4675.60,23888.17) | 55511.57(22750.59,90359.38) | 4.18(3.99,4.37) | 76.86(27.74,134.89) | 91.95(37.69,148.97) | 0.08(-0.12,0.28) |
| Timor-Leste | female | 51.82(19.27,93.07) | 252.51(95.83,459.82) | 5.63(5.42,5.84) | 41.51(15.00,74.52) | 60.18(22.58,107.39) | 1.25(1.05,1.45) |
| Togo | female | 205.56(79.82,359.18) | 988.17(410.02,1670.22) | 5.22(5.19,5.25) | 37.39(14.98,64.35) | 53.94(21.75,91.85) | 1.16(1.10,1.23) |
| Tokelau | female | 2.18(0.88,3.73) | 3.07(1.26,5.06) | 0.98(0.87,1.08) | 314.39(127.59,539.98) | 406.34(166.38,676.54) | 0.75(0.63,0.87) |
| Tonga | female | 124.13(52.04,199.34) | 227.55(98.96,355.88) | 1.92(1.84,1.99) | 415.45(174.72,663.93) | 527.86(230.05,827.91) | 0.78(0.73,0.83) |
| Trinidad and Tobago | female | 2440.07(1086.72,3753.17) | 4795.33(2009.53,7658.58) | 2.01(1.89,2.12) | 561.53(249.01,862.87) | 472.38(198.99,744.78) | -0.84(-0.97,-0.72) |
| Tunisia | female | 1072.97(454.59,1802.32) | 6483.59(2602.99,10715.31) | 5.97(5.86,6.07) | 48.25(20.47,80.17) | 95.55(38.58,156.50) | 2.30(2.21,2.39) |
| Türkiye | female | 25232.29(10625.73,39898.65) | 75217.80(33841.76,119442.34) | 4.04(3.71,4.37) | 149.04(64.08,234.93) | 151.75(68.18,241.18) | 0.52(0.15,0.90) |
| Turkmenistan | female | 284.97(113.68,476.08) | 1298.96(503.87,2161.51) | 4.65(4.35,4.95) | 26.13(10.62,43.12) | 57.51(22.70,95.50) | 2.19(1.93,2.45) |
| Tuvalu | female | 12.72(4.91,20.35) | 23.86(9.40,38.67) | 2.02(1.96,2.08) | 322.41(124.67,510.50) | 423.53(170.06,683.14) | 0.86(0.78,0.94) |
| Uganda | female | 1232.50(458.01,2263.78) | 3864.80(1350.73,7263.20) | 3.30(3.07,3.52) | 41.83(15.49,77.06) | 52.01(18.24,98.51) | 0.32(0.06,0.58) |
| Ukraine | female | 6616.62(2830.52,11271.46) | 10407.46(3765.46,18025.85) | 1.07(0.85,1.29) | 14.27(6.04,24.45) | 21.39(8.05,36.91) | 0.94(0.77,1.11) |
| United Arab Emirates | female | 277.80(115.89,456.21) | 2335.11(933.05,3837.40) | 7.72(7.45,7.98) | 193.67(80.87,320.90) | 403.86(166.68,654.06) | 3.78(3.30,4.27) |
| United Kingdom | female | 27533.51(12354.53,43295.31) | 44914.58(20398.47,70600.21) | 1.59(1.31,1.88) | 52.53(23.56,82.28) | 72.99(31.61,116.93) | 1.02(0.77,1.27) |
| United Republic of Tanzania | female | 1060.50(417.48,1855.00) | 3068.28(1155.75,5767.33) | 3.30(3.20,3.39) | 21.24(8.02,37.97) | 25.28(9.09,47.36) | 0.43(0.36,0.50) |
| United States of America | female | 97556.35(41443.02,152477.85) | 264074.73(110745.20,423215.50) | 3.11(2.99,3.24) | 51.48(21.83,79.83) | 83.87(35.26,135.77) | 1.46(1.33,1.59) |
| United States Virgin Islands | female | 77.81(32.56,124.78) | 158.16(66.04,263.12) | 2.33(2.09,2.58) | 179.65(76.01,282.18) | 161.06(64.99,262.04) | -0.43(-0.56,-0.31) |
| Uruguay | female | 1042.27(415.33,1706.04) | 2005.25(884.90,3387.72) | 2.25(2.15,2.34) | 45.97(18.59,74.54) | 62.46(27.87,103.62) | 1.14(1.03,1.25) |
| Uzbekistan | female | 1367.32(510.52,2355.78) | 8203.75(3542.76,14486.49) | 5.74(5.52,5.96) | 20.57(7.66,35.66) | 56.04(23.93,95.93) | 3.17(2.82,3.52) |
| Vanuatu | female | 24.27(9.85,45.24) | 100.69(40.64,179.51) | 4.36(4.18,4.53) | 94.32(38.41,170.70) | 123.17(47.74,217.49) | 0.63(0.52,0.74) |
| Venezuela (Bolivarian Republic of) | female | 4495.24(1895.79,7176.60) | 19480.56(8300.57,32258.79) | 4.85(4.61,5.08) | 90.19(37.02,143.29) | 119.87(50.97,199.21) | 0.79(0.55,1.03) |
| Viet Nam | female | 12609.20(4778.62,21860.78) | 40813.32(15473.44,71775.98) | 4.02(3.84,4.20) | 55.13(20.97,95.82) | 75.16(28.91,131.46) | 1.20(1.05,1.35) |
| Yemen | female | 1848.43(777.68,2971.40) | 8566.25(3661.66,13652.73) | 5.15(4.91,5.39) | 74.11(31.23,120.28) | 115.83(49.72,182.91) | 1.54(1.42,1.65) |
| Zambia | female | 919.24(357.83,1569.03) | 2559.99(943.40,4549.88) | 3.07(2.75,3.38) | 73.25(28.08,127.88) | 74.48(28.34,133.34) | -0.30(-0.48,-0.12) |
| Zimbabwe | female | 1526.44(623.79,2572.04) | 5690.93(2226.97,10057.13) | 4.93(4.32,5.56) | 81.15(32.78,137.50) | 156.26(58.38,266.24) | 3.04(2.38,3.71) |
| Afghanistan | male | 2691.17(1032.00,4676.57) | 5485.42(2142.87,9379.42) | 2.14(1.88,2.39) | 76.34(29.32,131.54) | 118.93(48.20,199.51) | 1.49(1.43,1.55) |
| Albania | male | 150.44(55.09,263.04) | 426.06(159.91,790.03) | 3.73(3.60,3.85) | 18.58(6.85,32.71) | 20.60(7.67,37.54) | 0.43(0.33,0.53) |
| Algeria | male | 3152.32(1244.36,5491.99) | 18075.13(7388.77,29667.69) | 5.94(5.73,6.14) | 57.03(23.24,98.55) | 102.22(43.18,167.47) | 2.00(1.86,2.15) |
| American Samoa | male | 56.36(24.23,87.89) | 154.39(65.23,247.44) | 3.05(2.63,3.48) | 449.29(187.79,698.69) | 606.79(252.55,969.19) | 0.87(0.55,1.20) |
| Andorra | male | 12.00(4.57,20.98) | 31.74(12.51,53.93) | 2.82(2.60,3.04) | 44.34(16.52,76.60) | 41.35(16.32,70.33) | -0.09(-0.32,0.15) |
| Angola | male | 1546.93(569.55,2930.36) | 4883.69(1788.90,8858.23) | 3.80(3.55,4.06) | 95.26(36.34,166.37) | 109.66(43.22,197.78) | 0.38(0.26,0.49) |
| Antigua and Barbuda | male | 29.53(11.31,51.92) | 65.22(24.59,115.84) | 2.63(2.30,2.95) | 128.15(48.70,224.47) | 136.10(52.41,237.33) | 0.20(-0.03,0.44) |
| Argentina | male | 5847.05(2447.72,10266.44) | 9312.19(3347.20,17181.51) | 1.48(1.32,1.63) | 43.35(18.34,74.53) | 38.79(13.88,70.28) | -0.38(-0.55,-0.21) |
| Armenia | male | 232.78(86.72,424.39) | 538.70(196.48,998.71) | 2.65(1.79,3.52) | 23.00(8.47,40.73) | 30.26(11.29,54.01) | 0.65(-0.04,1.35) |
| Australia | male | 3808.70(1693.86,6246.86) | 11528.36(4741.82,18820.65) | 3.40(3.29,3.51) | 45.68(20.16,74.71) | 54.92(22.52,90.02) | 0.38(0.27,0.49) |
| Austria | male | 1271.01(488.87,2133.84) | 2849.44(1085.00,4775.66) | 3.42(2.99,3.85) | 28.98(11.41,48.43) | 34.00(13.04,56.67) | 1.06(0.65,1.47) |
| Azerbaijan | male | 332.68(121.88,633.17) | 1228.90(461.90,2259.16) | 4.25(4.07,4.44) | 18.82(7.02,33.92) | 30.51(11.84,55.43) | 1.60(1.38,1.83) |
| Bahamas | male | 57.67(23.52,104.62) | 196.07(75.03,340.42) | 4.09(4.00,4.17) | 89.40(37.48,160.88) | 110.00(42.15,189.92) | 0.60(0.51,0.70) |
| Bahrain | male | 218.91(86.36,362.10) | 1604.28(676.54,2643.84) | 6.63(6.44,6.82) | 291.61(114.11,475.07) | 369.72(154.92,596.37) | 0.55(0.29,0.81) |
| Bangladesh | male | 6797.09(2593.40,12429.75) | 18664.93(6945.49,34985.36) | 3.11(2.89,3.34) | 27.13(10.00,50.39) | 27.74(10.41,51.56) | -0.36(-0.66,-0.07) |
| Barbados | male | 215.88(83.14,352.33) | 440.07(175.84,733.05) | 2.09(1.89,2.29) | 178.19(70.43,290.65) | 191.75(76.18,315.05) | -0.08(-0.31,0.15) |
| Belarus | male | 423.09(173.99,758.92) | 944.19(359.44,1651.39) | 1.86(1.51,2.22) | 9.77(4.13,17.66) | 16.82(6.55,28.73) | 1.13(0.80,1.46) |
| Belgium | male | 2116.44(891.33,3476.72) | 4087.28(1711.55,6796.00) | 2.18(2.10,2.26) | 34.93(14.58,56.77) | 38.93(16.42,64.29) | 0.25(0.15,0.34) |
| Belize | male | 30.76(12.07,51.85) | 147.91(58.41,261.82) | 5.31(4.92,5.71) | 68.68(26.99,114.55) | 101.74(40.74,178.50) | 1.27(0.75,1.80) |
| Benin | male | 208.61(70.53,418.26) | 664.10(233.08,1369.67) | 3.66(3.51,3.82) | 24.23(8.11,50.24) | 33.09(11.19,66.63) | 0.87(0.79,0.96) |
| Bermuda | male | 20.07(7.63,34.69) | 40.84(15.85,70.07) | 2.83(2.58,3.08) | 80.44(30.66,135.74) | 67.46(26.40,114.78) | -0.19(-0.38,0.00) |
| Bhutan | male | 130.33(54.88,212.16) | 462.63(192.02,772.28) | 4.28(4.19,4.37) | 114.86(49.91,184.44) | 152.12(63.55,255.22) | 1.01(0.98,1.04) |
| Bolivia (Plurinational State of) | male | 983.45(355.94,1690.71) | 3804.42(1438.16,6842.63) | 4.78(4.66,4.90) | 69.30(26.59,120.41) | 91.34(36.19,165.31) | 1.04(0.98,1.10) |
| Bosnia and Herzegovina | male | 508.46(199.35,906.21) | 1899.55(732.01,3404.84) | 4.90(4.47,5.34) | 30.32(12.15,55.43) | 67.69(26.43,118.90) | 3.17(2.83,3.51) |
| Botswana | male | 337.41(124.69,574.50) | 1016.79(420.79,1679.87) | 3.08(2.70,3.45) | 148.48(55.45,242.86) | 177.12(72.98,295.40) | 0.29(-0.14,0.72) |
| Brazil | male | 39217.62(16941.32,62544.49) | 125362.56(51497.01,199913.71) | 4.02(3.95,4.10) | 100.34(43.62,158.17) | 113.80(46.63,180.64) | 0.57(0.49,0.65) |
| Brunei Darussalam | male | 88.81(36.34,160.14) | 312.25(127.09,531.53) | 4.40(4.22,4.57) | 177.17(70.45,328.37) | 182.09(73.71,301.80) | 0.38(0.21,0.55) |
| Bulgaria | male | 1560.73(590.89,2653.32) | 2549.56(899.91,4496.66) | 1.60(1.43,1.77) | 28.72(10.76,47.17) | 42.99(14.74,74.31) | 1.30(1.13,1.46) |
| Burkina Faso | male | 848.94(317.56,1549.65) | 1923.62(648.03,3613.64) | 2.63(2.45,2.82) | 45.56(16.73,82.23) | 49.84(16.62,93.52) | 0.24(0.15,0.32) |
| Burundi | male | 457.23(164.98,844.18) | 863.96(270.84,1667.74) | 1.81(1.38,2.23) | 47.87(17.30,89.99) | 40.56(13.27,77.24) | -0.93(-1.09,-0.76) |
| Cabo Verde | male | 14.29(5.14,26.10) | 61.01(19.91,113.40) | 4.46(4.08,4.84) | 14.29(5.09,26.07) | 34.90(11.72,64.62) | 2.50(2.16,2.83) |
| Cambodia | male | 270.25(82.97,573.35) | 948.51(326.43,2082.60) | 4.16(3.82,4.50) | 17.59(5.55,36.40) | 23.38(7.99,51.50) | 0.88(0.65,1.11) |
| Cameroon | male | 1266.25(441.98,2424.91) | 5098.69(1686.45,9714.98) | 4.44(4.30,4.59) | 64.25(22.34,117.96) | 88.80(30.59,168.28) | 0.89(0.72,1.06) |
| Canada | male | 2396.88(840.78,4280.22) | 8323.73(2903.02,16430.71) | 3.73(3.48,3.98) | 18.41(6.29,33.74) | 24.59(8.81,49.08) | 0.50(0.22,0.78) |
| Central African Republic | male | 400.20(150.26,784.94) | 774.08(281.59,1504.41) | 2.08(1.91,2.25) | 83.54(30.02,165.57) | 82.44(31.09,143.43) | -0.12(-0.18,-0.06) |
| Chad | male | 479.94(178.82,840.92) | 1502.75(531.89,2636.78) | 3.69(3.57,3.82) | 37.32(14.08,65.81) | 50.67(17.98,89.37) | 0.87(0.64,1.10) |
| Chile | male | 1084.27(409.04,2034.11) | 3581.14(1300.70,6716.47) | 4.13(3.96,4.31) | 25.34(9.67,47.23) | 31.35(11.46,58.90) | 0.85(0.64,1.05) |
| China | male | 109402.47(42036.69,187657.60) | 376091.87(150747.58,629031.83) | 3.93(3.84,4.03) | 32.31(12.72,54.34) | 40.16(16.37,66.24) | 0.57(0.48,0.66) |
| Colombia | male | 5380.36(2147.63,9096.83) | 19375.07(7961.57,33651.70) | 4.01(3.88,4.14) | 61.34(24.27,103.33) | 76.92(31.25,133.34) | 0.42(0.26,0.58) |
| Comoros | male | 25.68(8.03,50.22) | 65.28(21.80,121.55) | 2.76(2.47,3.05) | 30.20(10.28,57.43) | 32.91(10.86,61.43) | -0.01(-0.23,0.20) |
| Congo | male | 479.16(185.68,851.20) | 1245.67(452.42,2260.64) | 2.88(2.34,3.41) | 104.83(42.33,184.97) | 95.56(34.11,172.76) | -0.59(-0.89,-0.29) |
| Cook Islands | male | 15.48(6.21,27.87) | 31.52(12.31,54.80) | 2.03(1.83,2.23) | 234.09(95.25,419.02) | 251.79(99.22,446.19) | 0.00(-0.15,0.15) |
| Costa Rica | male | 259.23(95.47,450.32) | 1353.51(492.94,2440.57) | 5.36(5.19,5.52) | 31.30(11.67,55.01) | 53.97(20.19,96.28) | 1.61(1.43,1.78) |
| Croatia | male | 700.05(276.05,1209.11) | 1714.79(703.17,2972.55) | 2.90(2.74,3.07) | 32.50(12.41,55.90) | 44.71(18.78,77.47) | 0.85(0.65,1.05) |
| Cuba | male | 1893.36(742.00,3227.27) | 4080.88(1505.06,7071.15) | 2.63(2.37,2.89) | 37.93(14.92,64.02) | 44.60(16.68,77.60) | 0.55(0.30,0.80) |
| Cyprus | male | 425.18(167.60,709.11) | 766.01(303.11,1315.13) | 1.52(1.34,1.71) | 159.22(60.24,264.45) | 83.48(32.33,139.18) | -2.07(-2.17,-1.97) |
| Czechia | male | 1931.96(729.38,3222.82) | 5815.50(2476.73,9635.68) | 4.96(4.30,5.63) | 35.36(13.79,58.45) | 60.69(25.77,101.90) | 2.90(2.37,3.44) |
| Côte d'Ivoire | male | 1184.06(447.72,2154.89) | 4197.97(1529.91,7427.84) | 3.67(3.45,3.90) | 61.43(23.47,110.10) | 75.77(27.99,132.98) | 0.36(0.17,0.54) |
| Democratic People's Republic of Korea | male | 2097.66(841.49,3947.66) | 5851.37(2199.08,10374.23) | 3.36(3.32,3.41) | 39.31(15.71,70.27) | 45.56(17.86,80.42) | 0.54(0.48,0.59) |
| Democratic Republic of the Congo | male | 8932.14(3563.43,15466.20) | 19171.14(7565.35,32289.39) | 2.19(1.83,2.54) | 149.71(58.03,251.42) | 145.71(57.28,244.85) | -0.28(-0.45,-0.12) |
| Denmark | male | 902.24(349.23,1456.12) | 2188.35(889.16,3642.88) | 2.89(2.63,3.14) | 26.46(10.19,43.30) | 39.19(15.93,65.21) | 1.26(0.91,1.61) |
| Djibouti | male | 17.88(5.91,33.12) | 132.90(46.29,264.62) | 6.65(6.54,6.76) | 33.58(11.68,60.33) | 47.72(16.38,90.82) | 1.06(0.96,1.16) |
| Dominica | male | 15.35(4.76,28.13) | 27.92(9.54,55.33) | 1.65(1.31,1.98) | 68.46(20.82,123.20) | 83.72(31.06,157.55) | 0.49(0.40,0.58) |
| Dominican Republic | male | 1201.91(453.36,2048.91) | 5624.03(2306.90,9707.31) | 5.52(5.33,5.70) | 66.72(25.44,114.10) | 115.10(47.66,199.08) | 2.17(2.04,2.30) |
| Ecuador | male | 1357.09(545.14,2320.55) | 6988.77(2665.39,12541.32) | 5.40(4.78,6.02) | 53.67(21.35,90.94) | 91.30(34.78,162.90) | 1.63(1.05,2.20) |
| Egypt | male | 8091.83(3086.58,13022.17) | 37026.64(14137.85,64303.39) | 5.12(5.02,5.23) | 61.45(23.59,99.58) | 117.87(46.03,203.09) | 2.31(2.19,2.43) |
| El Salvador | male | 564.63(210.85,1033.95) | 2313.18(817.56,4293.64) | 4.63(4.17,5.10) | 41.65(15.60,75.01) | 89.23(31.90,166.77) | 2.44(2.02,2.86) |
| Equatorial Guinea | male | 81.12(29.43,148.83) | 280.73(104.87,528.51) | 4.57(3.91,5.25) | 107.11(42.23,191.64) | 146.97(54.38,266.88) | 1.19(0.79,1.60) |
| Eritrea | male | 82.22(21.31,182.70) | 234.66(75.19,487.06) | 3.64(3.51,3.77) | 20.35(6.35,46.30) | 24.28(8.11,49.00) | 0.48(0.41,0.56) |
| Estonia | male | 76.24(27.41,138.17) | 299.22(120.98,518.00) | 4.21(3.78,4.64) | 11.48(4.11,20.23) | 29.90(12.11,51.55) | 2.73(2.36,3.11) |
| Eswatini | male | 169.64(65.63,303.23) | 514.49(181.74,948.66) | 3.74(3.04,4.44) | 151.79(57.98,264.12) | 242.92(89.66,433.02) | 1.84(1.30,2.37) |
| Ethiopia | male | 6120.73(2289.27,12208.55) | 8893.89(3156.60,15912.13) | 0.90(0.57,1.23) | 64.10(24.31,121.17) | 44.70(15.13,80.74) | -1.43(-1.67,-1.20) |
| Fiji | male | 592.80(216.22,1041.76) | 1719.95(730.81,3050.86) | 3.54(3.34,3.74) | 331.93(126.61,566.71) | 479.05(198.18,852.83) | 0.97(0.73,1.20) |
| Finland | male | 702.09(285.14,1255.83) | 1899.84(777.98,3175.27) | 3.32(3.13,3.51) | 26.35(10.94,45.87) | 33.78(14.07,56.64) | 0.75(0.58,0.92) |
| France | male | 10615.67(4607.45,17367.48) | 23431.03(10715.93,38803.86) | 2.67(2.34,3.00) | 32.84(14.37,53.66) | 37.19(17.03,61.08) | 0.38(0.03,0.73) |
| Gabon | male | 105.86(37.22,202.56) | 234.53(76.86,493.29) | 2.49(2.25,2.73) | 49.27(17.59,93.59) | 62.39(20.10,133.12) | 0.65(0.55,0.75) |
| Gambia | male | 49.89(17.94,97.25) | 199.84(69.26,379.98) | 4.41(4.23,4.59) | 31.57(12.36,57.85) | 46.38(15.79,88.89) | 1.11(1.01,1.21) |
| Georgia | male | 530.57(199.22,890.29) | 1059.39(401.19,1800.40) | 2.98(2.54,3.43) | 23.70(9.13,39.02) | 45.77(17.37,77.25) | 2.88(2.48,3.28) |
| Germany | male | 11447.54(4258.43,20014.60) | 25775.80(9535.62,45637.58) | 2.49(2.24,2.75) | 24.81(9.17,43.43) | 29.11(11.12,51.41) | 0.17(-0.11,0.44) |
| Ghana | male | 1315.01(497.81,2267.33) | 6400.56(2422.28,11112.81) | 5.68(5.39,5.98) | 50.46(19.53,87.16) | 94.00(36.47,158.38) | 2.45(2.19,2.70) |
| Greece | male | 1937.64(787.52,3385.48) | 4274.31(1600.87,7239.37) | 2.49(2.14,2.84) | 29.39(12.05,51.30) | 36.34(13.73,61.53) | 0.48(0.09,0.87) |
| Greenland | male | 2.88(1.05,5.37) | 8.86(3.18,15.86) | 4.03(3.82,4.25) | 23.48(8.46,41.62) | 27.50(9.99,48.00) | 0.67(0.48,0.86) |
| Grenada | male | 29.22(10.70,51.77) | 73.54(25.84,138.11) | 2.64(2.25,3.04) | 97.53(35.77,173.14) | 156.31(59.40,282.97) | 1.45(0.63,2.28) |
| Guam | male | 13.95(5.22,25.22) | 41.34(15.40,70.33) | 3.92(3.75,4.09) | 43.23(16.28,77.66) | 42.21(16.11,73.51) | 0.34(0.12,0.56) |
| Guatemala | male | 308.40(105.60,603.34) | 2277.23(836.62,4528.97) | 6.80(6.52,7.07) | 22.77(7.57,43.52) | 46.51(16.85,94.86) | 2.16(1.94,2.38) |
| Guinea | male | 286.39(94.16,548.25) | 622.32(213.28,1222.50) | 2.38(2.18,2.58) | 19.45(6.04,37.38) | 24.94(8.49,50.27) | 0.79(0.69,0.89) |
| Guinea-Bissau | male | 135.58(45.77,253.43) | 253.11(93.35,453.23) | 2.09(2.02,2.17) | 74.75(27.00,134.29) | 80.76(30.95,138.76) | 0.23(0.15,0.32) |
| Guyana | male | 202.63(72.15,353.88) | 512.93(187.18,957.26) | 3.38(3.19,3.57) | 117.65(42.37,204.98) | 176.88(67.44,313.74) | 1.48(1.23,1.73) |
| Haiti | male | 1264.46(460.03,2346.42) | 2902.37(956.39,5272.60) | 2.86(2.74,2.99) | 90.88(33.09,167.29) | 93.36(32.42,172.31) | 0.20(0.08,0.33) |
| Honduras | male | 286.11(99.32,547.01) | 1505.09(557.83,2871.02) | 5.99(5.80,6.17) | 30.27(10.75,57.15) | 52.54(19.12,97.13) | 2.11(1.97,2.26) |
| Hungary | male | 1649.76(603.22,2792.70) | 3365.06(1232.58,5589.50) | 2.62(2.39,2.86) | 28.18(10.00,46.86) | 42.56(15.85,70.36) | 1.54(1.28,1.81) |
| Iceland | male | 28.02(11.85,45.66) | 91.38(36.25,155.35) | 3.99(3.88,4.09) | 21.53(9.03,35.31) | 33.06(13.10,57.16) | 1.46(1.39,1.53) |
| India | male | 127449.29(52181.83,205689.28) | 413908.90(183687.32,677635.77) | 3.73(3.59,3.86) | 60.35(25.12,96.07) | 78.67(34.29,127.46) | 0.79(0.65,0.92) |
| Indonesia | male | 37885.01(15699.76,59968.73) | 151964.18(62923.08,247943.69) | 4.41(4.31,4.52) | 88.82(37.42,139.12) | 142.47(60.99,226.27) | 1.62(1.57,1.66) |
| Iran (Islamic Republic of) | male | 5219.04(1972.76,8938.32) | 26876.50(10076.96,45126.09) | 5.90(5.74,6.06) | 42.26(15.71,69.45) | 70.55(26.71,117.07) | 2.07(1.91,2.23) |
| Iraq | male | 6868.88(2614.12,11219.93) | 30708.69(13040.31,51152.48) | 4.98(4.77,5.20) | 174.59(67.19,285.82) | 249.51(106.67,413.61) | 1.01(0.84,1.18) |
| Ireland | male | 759.93(328.40,1246.53) | 1440.99(595.83,2375.38) | 2.10(1.93,2.27) | 43.16(18.58,70.39) | 38.75(15.81,63.93) | -0.38(-0.49,-0.28) |
| Israel | male | 1166.86(462.51,2036.10) | 3207.33(1218.83,5256.25) | 2.96(2.36,3.56) | 53.55(21.33,93.11) | 56.16(21.49,92.60) | -0.18(-0.79,0.44) |
| Italy | male | 18779.21(7649.06,29834.12) | 31535.93(13434.43,52849.44) | 1.94(1.83,2.06) | 50.76(21.11,80.51) | 47.46(19.82,79.16) | -0.05(-0.16,0.06) |
| Jamaica | male | 939.05(346.97,1546.25) | 1939.20(802.71,3337.12) | 2.22(1.84,2.61) | 111.50(41.51,186.70) | 134.07(55.20,230.58) | 0.51(0.14,0.88) |
| Japan | male | 37916.90(15326.36,59968.47) | 84892.99(32539.80,140750.56) | 2.54(2.40,2.69) | 51.52(20.98,80.56) | 60.78(23.47,101.49) | 0.43(0.36,0.50) |
| Jordan | male | 741.55(314.60,1290.02) | 5876.82(2275.23,9925.16) | 6.77(6.47,7.08) | 116.65(49.04,203.73) | 152.38(57.23,260.37) | 0.81(0.72,0.89) |
| Kazakhstan | male | 759.36(288.42,1356.53) | 2267.98(841.76,4257.36) | 3.09(2.82,3.36) | 16.39(6.24,29.30) | 31.39(11.68,57.34) | 1.76(1.55,1.96) |
| Kenya | male | 1252.76(490.36,2183.02) | 5356.74(2068.03,9466.16) | 4.82(4.73,4.91) | 34.41(13.66,60.89) | 55.75(21.05,98.84) | 1.55(1.36,1.74) |
| Kiribati | male | 72.75(28.53,122.82) | 173.40(68.80,303.56) | 2.73(2.55,2.90) | 413.92(160.72,685.62) | 501.22(199.63,870.18) | 0.39(0.13,0.65) |
| Kuwait | male | 572.75(251.79,944.07) | 4342.39(1874.35,7185.56) | 6.81(6.60,7.01) | 130.25(56.67,212.06) | 221.65(96.33,364.05) | 1.61(1.32,1.91) |
| Kyrgyzstan | male | 138.18(54.91,244.95) | 407.87(143.35,753.84) | 2.77(2.51,3.03) | 13.10(5.17,22.74) | 22.13(8.39,39.49) | 1.15(0.90,1.41) |
| Lao People's Democratic Republic | male | 352.40(128.13,682.28) | 726.85(269.35,1370.41) | 2.00(1.67,2.33) | 38.21(13.65,71.75) | 37.07(13.62,72.80) | -0.33(-0.52,-0.14) |
| Latvia | male | 165.78(66.51,285.34) | 474.46(185.28,816.23) | 3.12(2.78,3.47) | 13.15(5.03,22.54) | 33.49(13.09,58.34) | 2.74(2.40,3.09) |
| Lebanon | male | 1555.73(657.77,2676.78) | 5675.97(2350.95,9500.32) | 4.87(4.66,5.07) | 153.54(66.16,260.52) | 202.06(83.11,339.82) | 1.34(1.14,1.55) |
| Lesotho | male | 94.80(32.11,178.73) | 208.53(70.32,435.77) | 2.60(2.33,2.87) | 41.38(14.48,76.51) | 68.83(26.16,132.69) | 1.97(1.70,2.23) |
| Liberia | male | 329.20(121.03,594.88) | 871.02(326.61,1551.05) | 3.37(3.03,3.71) | 54.88(20.56,99.87) | 76.03(29.12,132.52) | 1.17(1.02,1.32) |
| Libya | male | 608.31(241.17,1033.62) | 3470.08(1300.84,5895.23) | 6.19(6.07,6.32) | 62.32(24.89,103.14) | 130.16(52.04,221.24) | 2.83(2.64,3.03) |
| Lithuania | male | 245.15(98.34,440.71) | 842.93(335.95,1416.04) | 3.65(3.29,4.01) | 14.57(5.90,26.10) | 40.32(16.14,67.78) | 2.86(2.48,3.24) |
| Luxembourg | male | 69.55(29.47,113.86) | 149.39(57.29,244.72) | 2.44(2.22,2.66) | 33.67(14.54,55.34) | 30.92(11.82,50.50) | -0.42(-0.54,-0.30) |
| Madagascar | male | 643.40(209.72,1187.31) | 1327.47(467.14,2506.60) | 2.43(2.12,2.75) | 28.68(9.22,54.10) | 30.08(11.12,58.49) | 0.15(0.09,0.21) |
| Malawi | male | 851.92(327.71,1572.82) | 2005.94(733.16,3795.80) | 2.45(2.29,2.62) | 53.17(20.16,93.03) | 68.50(25.82,124.01) | 0.60(0.33,0.88) |
| Malaysia | male | 3676.92(1442.17,6127.45) | 14922.10(6298.23,24622.21) | 4.64(4.51,4.77) | 84.45(33.42,141.59) | 105.48(44.74,171.78) | 0.55(0.41,0.69) |
| Maldives | male | 84.34(34.10,133.49) | 247.30(104.06,395.15) | 3.08(2.71,3.45) | 163.90(64.99,259.44) | 137.36(58.96,220.45) | -0.81(-1.02,-0.61) |
| Mali | male | 1232.47(541.71,2131.29) | 3916.44(1517.05,6936.73) | 4.19(4.00,4.38) | 67.26(28.89,117.78) | 90.49(35.70,161.15) | 1.11(1.03,1.18) |
| Malta | male | 112.13(46.48,181.49) | 322.46(126.32,542.39) | 3.53(3.28,3.77) | 63.18(26.19,101.03) | 73.36(29.22,121.04) | 0.45(0.24,0.67) |
| Marshall Islands | male | 39.67(15.28,66.23) | 128.99(50.01,241.61) | 4.02(3.84,4.19) | 449.41(172.63,758.67) | 616.60(235.61,1185.63) | 1.08(0.96,1.20) |
| Mauritania | male | 433.30(168.29,739.50) | 1162.24(457.01,1982.66) | 3.12(2.92,3.33) | 94.51(35.33,159.61) | 110.35(41.80,186.82) | 0.31(0.14,0.47) |
| Mauritius | male | 314.09(110.01,563.60) | 1861.52(716.41,3253.94) | 7.40(6.65,8.15) | 97.33(36.50,170.44) | 219.20(86.00,370.00) | 3.93(3.18,4.69) |
| Mexico | male | 19834.28(7855.03,33289.47) | 74418.85(28986.66,127427.50) | 4.54(4.20,4.89) | 100.63(39.55,167.14) | 126.47(49.10,217.48) | 0.82(0.53,1.11) |
| Micronesia (Federated States of) | male | 109.26(43.91,180.75) | 239.32(96.93,391.04) | 2.60(2.52,2.68) | 447.05(184.42,751.88) | 605.84(248.68,973.88) | 0.92(0.72,1.13) |
| Monaco | male | 6.49(2.84,10.95) | 15.07(6.20,25.76) | 2.74(2.61,2.87) | 22.01(9.62,37.18) | 33.73(13.49,57.97) | 1.39(1.29,1.49) |
| Mongolia | male | 53.42(18.48,103.77) | 178.27(64.55,338.04) | 3.97(3.56,4.38) | 12.10(4.28,22.82) | 18.54(6.75,34.03) | 1.31(1.18,1.44) |
| Montenegro | male | 90.66(35.61,156.43) | 206.02(81.08,362.21) | 3.00(2.90,3.10) | 34.97(14.58,59.82) | 49.29(19.40,85.35) | 1.24(1.12,1.36) |
| Morocco | male | 4287.53(1735.92,7347.35) | 22092.07(9086.46,37048.55) | 5.64(5.42,5.86) | 62.07(25.69,106.96) | 131.54(54.15,221.73) | 2.59(2.47,2.71) |
| Mozambique | male | 852.36(260.87,1660.26) | 2305.44(749.18,4388.07) | 3.75(3.58,3.92) | 35.06(11.36,66.51) | 53.22(18.03,100.27) | 1.81(1.63,1.99) |
| Myanmar | male | 3077.86(915.00,6386.43) | 7426.98(2422.08,15251.92) | 2.79(2.40,3.17) | 31.07(9.08,62.77) | 38.77(12.84,79.19) | 0.70(0.43,0.96) |
| Namibia | male | 338.63(147.53,578.42) | 943.96(350.15,1599.17) | 3.04(2.71,3.38) | 125.79(52.25,209.89) | 175.68(66.93,294.66) | 0.93(0.62,1.23) |
| Nauru | male | 5.90(1.92,10.80) | 7.81(3.04,13.85) | 0.45(0.26,0.65) | 214.53(70.76,385.28) | 278.30(109.23,485.41) | 0.56(0.40,0.72) |
| Nepal | male | 1325.59(509.65,2518.21) | 5183.08(1983.10,9853.34) | 4.86(4.62,5.09) | 31.74(12.16,59.85) | 50.50(18.99,93.76) | 1.65(1.43,1.87) |
| Netherlands | male | 2429.08(855.90,4326.04) | 4418.05(1557.66,7792.08) | 1.86(1.64,2.08) | 30.44(10.61,52.71) | 27.19(9.55,48.08) | -0.56(-0.75,-0.36) |
| New Zealand | male | 605.48(232.50,1047.08) | 1954.20(848.78,3352.00) | 3.82(3.67,3.97) | 36.23(14.04,61.71) | 49.41(21.43,84.32) | 0.90(0.75,1.06) |
| Nicaragua | male | 290.67(102.81,547.03) | 1448.34(505.06,2734.06) | 5.49(5.19,5.78) | 43.78(16.80,81.39) | 66.39(23.15,124.85) | 1.54(1.31,1.77) |
| Niger | male | 541.27(190.17,1015.56) | 1771.49(664.83,3317.84) | 4.02(3.93,4.10) | 41.40(15.62,75.37) | 47.53(17.49,86.47) | 0.51(0.45,0.57) |
| Nigeria | male | 13152.37(5064.55,22937.41) | 27638.73(10154.43,46862.26) | 2.23(2.13,2.32) | 64.04(24.23,107.35) | 71.00(25.58,120.67) | 0.22(0.16,0.27) |
| Niue | male | 1.54(0.59,2.53) | 2.60(0.98,4.86) | 1.35(1.14,1.56) | 164.49(64.21,270.44) | 257.61(97.12,466.96) | 1.33(1.15,1.51) |
| North Macedonia | male | 311.89(113.34,547.41) | 871.68(305.22,1589.30) | 3.63(3.37,3.89) | 37.76(13.67,64.69) | 58.54(20.83,106.01) | 1.55(1.24,1.86) |
| Northern Mariana Islands | male | 15.11(5.17,27.54) | 44.95(17.31,77.55) | 3.93(3.48,4.39) | 135.64(50.94,236.24) | 160.24(66.10,275.64) | 0.51(0.26,0.76) |
| Norway | male | 874.92(344.65,1472.87) | 1616.62(674.73,2646.22) | 1.84(1.72,1.96) | 30.54(12.15,50.90) | 34.97(14.76,57.19) | 0.30(0.12,0.48) |
| Oman | male | 397.50(159.30,721.17) | 2013.02(812.46,3256.85) | 5.30(5.07,5.53) | 119.70(49.78,212.91) | 194.69(80.76,316.42) | 2.01(1.81,2.21) |
| Pakistan | male | 18647.05(7474.72,31477.62) | 62637.22(24140.79,110731.16) | 3.85(3.74,3.96) | 61.81(24.59,103.83) | 100.01(39.48,177.78) | 1.43(1.20,1.67) |
| Palau | male | 8.14(3.06,14.82) | 27.13(10.90,47.30) | 3.60(3.21,3.99) | 166.26(63.38,302.23) | 211.10(87.95,367.78) | 0.69(0.53,0.85) |
| Palestine | male | 440.21(165.78,751.48) | 1626.40(644.59,2715.94) | 4.24(4.15,4.34) | 127.46(49.67,217.76) | 152.55(59.42,253.81) | 0.53(0.37,0.69) |
| Panama | male | 244.27(84.29,443.05) | 1267.40(484.36,2330.58) | 5.42(5.23,5.62) | 33.88(11.57,63.08) | 59.71(22.77,109.20) | 1.77(1.56,1.97) |
| Papua New Guinea | male | 1540.31(553.44,3009.69) | 4632.51(1596.99,8628.32) | 3.66(3.48,3.83) | 159.07(59.00,294.68) | 163.18(57.20,298.46) | 0.02(-0.03,0.07) |
| Paraguay | male | 461.34(174.16,814.39) | 2448.57(840.29,4706.61) | 5.85(5.68,6.02) | 46.81(17.38,83.34) | 92.42(32.73,172.11) | 2.55(2.40,2.71) |
| Peru | male | 2428.48(979.32,4100.26) | 8444.79(3358.40,15177.85) | 4.27(4.10,4.44) | 43.68(17.89,75.22) | 53.15(21.07,94.46) | 0.61(0.44,0.78) |
| Philippines | male | 4788.45(1804.30,8304.07) | 19864.30(7536.68,36040.91) | 4.89(4.77,5.01) | 36.74(13.92,65.20) | 54.81(21.70,97.84) | 1.50(1.42,1.59) |
| Poland | male | 6910.16(2717.09,12056.98) | 16219.11(6834.27,27013.35) | 3.01(2.71,3.31) | 39.82(15.52,68.89) | 54.28(22.88,89.70) | 1.10(0.89,1.31) |
| Portugal | male | 3810.77(1553.90,6448.83) | 7406.06(2995.79,12285.13) | 1.95(1.66,2.24) | 67.31(27.42,112.95) | 68.86(28.08,115.21) | -0.23(-0.49,0.03) |
| Puerto Rico | male | 2041.89(875.83,3298.95) | 4356.98(1670.28,7083.88) | 2.36(2.15,2.57) | 125.27(53.94,200.94) | 138.73(54.45,229.47) | 0.21(0.06,0.36) |
| Qatar | male | 153.93(58.35,250.80) | 1881.34(751.43,3216.84) | 9.24(8.88,9.61) | 261.11(102.52,412.07) | 295.77(117.58,487.36) | 0.31(-0.28,0.90) |
| Republic of Korea | male | 8531.18(3501.99,14702.30) | 31400.77(13287.14,55461.60) | 4.09(3.91,4.27) | 68.93(29.57,113.71) | 75.37(31.58,132.92) | -0.07(-0.31,0.18) |
| Republic of Moldova | male | 255.99(103.96,463.88) | 650.96(254.14,1135.47) | 2.66(2.34,2.99) | 15.85(6.51,27.66) | 27.67(10.86,47.55) | 1.40(1.09,1.71) |
| Romania | male | 2745.98(1111.48,4614.29) | 4800.37(1890.13,8039.81) | 2.16(1.90,2.42) | 23.12(9.56,38.88) | 30.49(11.79,51.48) | 1.17(0.92,1.42) |
| Russian Federation | male | 6828.68(2549.83,11987.51) | 26664.57(10708.15,45153.96) | 4.10(3.49,4.72) | 12.90(4.96,21.32) | 31.27(12.76,51.53) | 2.63(2.06,3.21) |
| Rwanda | male | 531.05(172.99,1130.94) | 891.91(309.57,1879.65) | 1.06(0.42,1.70) | 48.36(16.21,100.54) | 41.74(14.12,91.13) | -1.35(-1.70,-1.00) |
| Saint Kitts and Nevis | male | 19.45(7.66,34.02) | 44.40(16.29,79.29) | 2.98(2.60,3.36) | 124.99(48.89,209.26) | 149.91(57.52,261.53) | 0.86(0.67,1.05) |
| Saint Lucia | male | 48.81(18.78,87.72) | 137.82(53.31,249.63) | 3.41(3.23,3.58) | 139.91(52.27,253.03) | 125.67(48.05,231.56) | -0.53(-0.68,-0.38) |
| Saint Vincent and the Grenadines | male | 33.94(12.87,60.04) | 97.31(36.29,170.09) | 3.37(3.20,3.55) | 115.69(44.18,200.11) | 139.09(51.13,241.50) | 0.46(0.31,0.62) |
| Samoa | male | 72.03(27.72,126.83) | 153.35(61.33,276.12) | 2.42(2.34,2.51) | 169.41(64.64,293.10) | 207.07(80.39,370.70) | 0.58(0.52,0.64) |
| San Marino | male | 4.28(1.69,6.98) | 11.08(4.30,19.35) | 3.74(3.44,4.05) | 27.30(10.75,44.37) | 31.06(11.99,54.95) | 0.88(0.71,1.06) |
| Sao Tome and Principe | male | 4.59(1.46,8.98) | 11.40(3.91,21.41) | 2.91(2.74,3.09) | 20.32(6.65,39.07) | 25.98(8.88,48.96) | 1.08(0.91,1.25) |
| Saudi Arabia | male | 3405.52(1401.04,5856.77) | 23422.08(9201.28,39176.82) | 6.16(5.97,6.34) | 105.50(43.09,179.07) | 185.90(76.98,306.40) | 1.69(1.60,1.78) |
| Senegal | male | 1217.37(465.09,2052.57) | 3750.65(1502.45,6438.02) | 3.81(3.74,3.87) | 78.03(30.08,130.01) | 105.04(43.15,181.29) | 0.97(0.92,1.02) |
| Serbia | male | 1866.32(635.00,3351.74) | 3920.34(1435.00,6703.61) | 2.48(2.33,2.63) | 42.23(15.21,75.38) | 52.43(19.09,91.56) | 0.89(0.79,0.98) |
| Seychelles | male | 12.60(4.82,21.85) | 49.30(19.46,84.98) | 4.60(4.49,4.71) | 53.63(20.80,90.23) | 97.69(38.10,169.13) | 2.00(1.82,2.17) |
| Sierra Leone | male | 432.64(162.94,822.55) | 931.55(342.30,1716.98) | 2.47(2.27,2.67) | 43.82(16.19,80.00) | 51.81(19.71,92.19) | 0.57(0.48,0.66) |
| Singapore | male | 666.45(277.75,1129.97) | 2294.56(882.13,4064.35) | 4.15(4.06,4.24) | 62.45(26.03,106.65) | 53.13(20.41,94.82) | -0.57(-0.76,-0.37) |
| Slovakia | male | 611.71(225.62,1053.79) | 1027.65(381.48,1785.78) | 1.80(1.68,1.92) | 25.08(9.29,43.23) | 26.45(9.68,45.07) | 0.25(0.21,0.30) |
| Slovenia | male | 241.21(94.72,417.23) | 570.93(224.62,1008.98) | 2.17(1.90,2.44) | 25.54(9.69,44.61) | 29.50(11.71,51.55) | -0.41(-0.75,-0.07) |
| Solomon Islands | male | 166.02(60.14,299.35) | 520.26(207.14,878.05) | 3.66(3.60,3.72) | 210.08(81.62,379.38) | 277.79(112.62,469.82) | 0.92(0.88,0.97) |
| Somalia | male | 451.01(155.89,890.37) | 1092.53(355.62,2193.35) | 3.07(2.99,3.16) | 48.74(16.28,93.92) | 50.95(17.02,97.06) | 0.19(0.12,0.27) |
| South Africa | male | 12173.73(4875.68,19332.04) | 44030.90(18843.44,70550.58) | 4.17(3.84,4.49) | 143.08(58.59,226.59) | 237.06(99.04,373.61) | 1.65(1.28,2.03) |
| South Sudan | male | 564.64(200.52,1099.69) | 927.77(325.10,1884.75) | 1.44(1.17,1.71) | 41.71(14.62,82.90) | 53.38(18.41,106.56) | 0.70(0.56,0.84) |
| Spain | male | 12761.01(5621.01,20946.67) | 23317.61(9593.85,38556.14) | 1.82(1.66,1.98) | 56.26(24.76,91.48) | 53.13(21.95,86.82) | -0.40(-0.49,-0.30) |
| Sri Lanka | male | 1420.77(488.26,2721.73) | 5185.47(1862.75,10728.20) | 4.71(4.57,4.86) | 28.98(9.76,54.86) | 44.31(16.05,92.14) | 1.88(1.71,2.05) |
| Sudan | male | 4604.26(1962.72,7494.64) | 18977.06(8389.84,29982.32) | 4.68(4.51,4.85) | 92.13(38.95,151.64) | 165.01(72.23,258.21) | 2.01(1.88,2.14) |
| Suriname | male | 165.29(66.21,279.45) | 622.47(248.62,1038.60) | 4.77(4.58,4.97) | 128.78(52.34,218.61) | 203.96(83.06,340.94) | 1.69(1.54,1.85) |
| Sweden | male | 1550.13(605.03,2539.33) | 3322.29(1309.62,5769.10) | 2.78(2.64,2.93) | 23.89(9.27,39.34) | 33.42(13.08,57.18) | 1.38(1.26,1.51) |
| Switzerland | male | 1688.31(689.19,2779.39) | 3458.30(1243.81,5868.46) | 2.15(2.03,2.26) | 39.52(15.95,64.58) | 41.63(15.15,70.85) | -0.07(-0.18,0.03) |
| Syrian Arab Republic | male | 1920.67(729.85,3227.66) | 6993.48(2764.86,11902.09) | 4.24(4.10,4.39) | 72.68(28.17,120.66) | 105.85(43.28,176.11) | 1.03(0.88,1.19) |
| Taiwan (Province of China) | male | 7857.77(3190.83,12560.92) | 20978.80(8810.46,34732.76) | 2.66(2.31,3.01) | 96.26(39.95,151.57) | 107.14(44.68,175.90) | -0.18(-0.50,0.13) |
| Tajikistan | male | 197.39(73.08,372.55) | 597.75(194.31,1066.08) | 3.43(3.31,3.54) | 17.91(6.70,32.91) | 23.90(8.46,41.63) | 0.73(0.49,0.97) |
| Thailand | male | 5969.24(2307.21,10172.29) | 27721.82(11310.08,48710.04) | 4.81(4.67,4.96) | 42.14(16.18,70.00) | 57.15(23.58,99.39) | 0.68(0.55,0.81) |
| Timor-Leste | male | 14.22(4.23,27.80) | 69.30(21.62,137.57) | 5.52(5.34,5.70) | 12.14(3.90,24.69) | 17.91(5.37,35.51) | 1.32(1.09,1.54) |
| Togo | male | 137.46(49.33,255.88) | 537.81(154.93,1034.98) | 4.25(4.14,4.36) | 28.20(9.81,52.19) | 40.08(11.81,76.25) | 0.99(0.83,1.15) |
| Tokelau | male | 0.81(0.29,1.49) | 1.24(0.50,2.22) | 1.18(1.08,1.28) | 124.77(46.93,225.50) | 174.73(70.19,311.59) | 1.00(0.89,1.12) |
| Tonga | male | 33.18(12.77,58.52) | 69.25(26.01,119.87) | 2.21(1.94,2.47) | 122.70(46.71,215.35) | 180.38(68.52,314.06) | 1.10(0.86,1.34) |
| Trinidad and Tobago | male | 1298.56(521.46,2136.14) | 3500.08(1546.71,5942.47) | 3.26(3.13,3.39) | 332.96(134.50,553.08) | 366.61(162.19,622.62) | 0.18(0.04,0.31) |
| Tunisia | male | 951.84(367.19,1645.09) | 5182.22(2015.36,8932.13) | 5.59(5.45,5.72) | 41.86(16.27,70.76) | 86.46(33.73,148.00) | 2.40(2.26,2.55) |
| Türkiye | male | 12245.08(4850.48,20530.39) | 39165.99(14598.51,65924.60) | 3.88(3.64,4.13) | 85.26(34.70,143.82) | 93.34(35.84,158.06) | 0.23(0.04,0.42) |
| Turkmenistan | male | 127.91(52.99,226.53) | 623.35(231.20,1144.88) | 4.82(4.57,5.08) | 17.92(7.42,30.84) | 38.31(15.05,67.20) | 1.98(1.71,2.24) |
| Tuvalu | male | 4.08(1.52,7.16) | 8.40(3.08,14.81) | 2.34(2.29,2.38) | 136.98(52.12,240.99) | 168.86(63.74,289.15) | 0.67(0.57,0.77) |
| Uganda | male | 1082.85(340.49,2350.79) | 2623.97(873.23,5640.15) | 2.44(2.26,2.62) | 39.52(13.57,75.77) | 49.35(16.70,102.34) | 0.32(0.14,0.50) |
| Ukraine | male | 1786.79(677.96,3376.09) | 3596.49(1299.83,6451.09) | 1.81(1.61,2.02) | 7.58(2.96,13.39) | 12.46(4.52,22.42) | 1.25(1.08,1.41) |
| United Arab Emirates | male | 388.54(145.37,679.19) | 5807.84(2285.37,9716.43) | 9.31(9.08,9.53) | 133.22(52.57,229.75) | 144.65(56.52,234.86) | 1.01(0.57,1.45) |
| United Kingdom | male | 20370.33(8593.70,31399.57) | 42706.87(18544.07,68462.61) | 2.27(1.99,2.54) | 55.59(23.75,85.64) | 76.22(32.15,124.07) | 0.80(0.57,1.04) |
| United Republic of Tanzania | male | 656.60(234.89,1276.92) | 1520.56(504.03,2998.24) | 2.11(1.87,2.35) | 14.28(5.09,26.92) | 14.42(4.55,28.81) | -0.54(-0.75,-0.32) |
| United States of America | male | 48064.74(18635.62,83190.58) | 182996.80(73624.54,309765.22) | 4.27(4.02,4.52) | 36.40(14.20,62.61) | 68.59(27.45,114.76) | 1.89(1.70,2.07) |
| United States Virgin Islands | male | 19.87(7.58,34.44) | 72.43(27.21,131.90) | 4.41(4.18,4.63) | 62.10(22.97,109.13) | 87.24(33.40,158.95) | 1.21(1.04,1.37) |
| Uruguay | male | 552.87(214.61,936.08) | 1083.00(401.86,1886.78) | 2.23(2.09,2.37) | 32.55(13.20,55.11) | 47.34(17.35,83.15) | 1.29(1.18,1.40) |
| Uzbekistan | male | 295.61(99.20,569.74) | 1820.10(597.48,3548.33) | 5.88(5.61,6.16) | 6.67(2.33,12.85) | 16.74(5.53,31.96) | 2.95(2.58,3.33) |
| Vanuatu | male | 12.80(4.24,26.37) | 38.55(12.45,77.07) | 3.34(3.20,3.48) | 42.19(13.90,82.08) | 48.94(16.19,97.42) | 0.24(0.15,0.33) |
| Venezuela (Bolivarian Republic of) | male | 2168.67(831.78,4031.48) | 10475.52(3907.23,19806.50) | 5.33(5.18,5.49) | 49.58(18.80,91.55) | 78.23(28.95,144.19) | 1.43(1.28,1.58) |
| Viet Nam | male | 5365.57(1874.35,9784.35) | 20550.61(7242.86,36337.41) | 4.56(4.38,4.75) | 34.52(11.68,63.19) | 51.98(18.94,95.42) | 1.52(1.42,1.63) |
| Yemen | male | 1139.17(447.62,2000.10) | 4679.29(1922.39,8203.79) | 4.66(4.50,4.81) | 52.88(20.96,94.46) | 69.57(28.12,120.01) | 0.89(0.75,1.03) |
| Zambia | male | 913.72(348.00,1601.59) | 2378.94(936.39,4413.42) | 2.61(2.39,2.84) | 66.71(25.79,118.32) | 78.21(28.90,143.44) | 0.10(-0.11,0.31) |
| Zimbabwe | male | 915.33(358.01,1644.90) | 1935.09(759.08,3414.45) | 1.97(1.54,2.40) | 54.12(21.32,95.24) | 74.61(27.37,129.92) | 0.93(0.54,1.32) |

Supplementary table 2 The death cases and ASDR diabetes and kidney diseases due to Low physical activity in 1990 and 2021 and its temporal trends.

| Deaths |  | Deaths No.(95%UI) | | | ASDR (per 100000) No.95%UI | | |
| --- | --- | --- | --- | --- | --- | --- | --- |
| nation | sex | 1990 | 2021 | 1990-2021 EAPC No.(95%CI) | 1990 | 2021 | 1990-2021 EAPC No.(95%CI) |
| Afghanistan | both | 247.45(97.15,404.51) | 485.13(209.43,798.85) | 2.23(2.11,2.34) | 3.99(1.56,6.44) | 6.09(2.55,9.85) | 1.56(1.47,1.66) |
| Albania | both | 10.06(4.16,16.59) | 26.13(10.54,43.69) | 3.63(3.33,3.94) | 0.61(0.26,1.00) | 0.62(0.25,1.03) | 0.35(0.07,0.64) |
| Algeria | both | 199.40(77.75,348.58) | 967.43(422.08,1556.86) | 5.48(5.25,5.72) | 2.48(0.97,4.23) | 3.56(1.54,5.81) | 1.70(1.46,1.95) |
| American Samoa | both | 2.83(1.25,4.38) | 8.90(4.02,14.25) | 3.57(3.09,4.05) | 14.35(6.41,22.08) | 20.18(9.12,32.12) | 1.05(0.69,1.41) |
| Andorra | both | 0.86(0.35,1.49) | 2.26(1.01,3.76) | 3.16(2.82,3.51) | 1.85(0.80,3.17) | 1.26(0.56,2.12) | -0.89(-1.12,-0.65) |
| Angola | both | 90.41(34.52,152.75) | 286.01(106.44,508.29) | 3.69(3.48,3.91) | 3.39(1.33,5.68) | 3.62(1.48,6.42) | 0.05(-0.06,0.15) |
| Antigua and Barbuda | both | 4.17(1.76,6.55) | 6.68(2.77,10.51) | 1.17(0.98,1.37) | 7.23(3.08,11.39) | 7.15(3.01,11.34) | -0.34(-0.53,-0.15) |
| Argentina | both | 668.13(268.08,1097.05) | 981.00(393.48,1591.83) | 1.37(1.01,1.73) | 2.25(0.91,3.75) | 1.68(0.67,2.72) | -0.85(-1.13,-0.58) |
| Armenia | both | 27.37(11.20,44.09) | 52.72(21.33,86.08) | 1.98(0.68,3.30) | 1.14(0.46,1.85) | 1.23(0.49,1.98) | -0.09(-1.22,1.05) |
| Australia | both | 331.80(139.49,517.33) | 774.91(326.22,1220.20) | 2.65(2.35,2.95) | 1.77(0.75,2.75) | 1.47(0.62,2.31) | -0.69(-0.96,-0.43) |
| Austria | both | 242.37(102.77,375.04) | 390.27(165.23,655.37) | 2.66(2.13,3.19) | 1.89(0.80,2.95) | 1.64(0.71,2.74) | 0.45(-0.07,0.98) |
| Azerbaijan | both | 32.71(12.37,54.66) | 107.69(41.77,187.42) | 3.77(3.37,4.16) | 0.75(0.29,1.25) | 1.28(0.52,2.14) | 1.75(1.33,2.16) |
| Bahamas | both | 7.77(3.38,12.24) | 16.13(6.39,26.72) | 2.13(1.91,2.35) | 5.60(2.44,8.76) | 4.51(1.78,7.48) | -1.01(-1.24,-0.77) |
| Bahrain | both | 15.23(6.45,23.83) | 86.22(36.38,137.32) | 5.40(5.21,5.60) | 14.66(5.98,23.46) | 19.03(8.04,30.23) | 0.66(0.20,1.13) |
| Bangladesh | both | 783.30(335.69,1271.51) | 3241.53(1279.33,5425.84) | 4.73(4.48,4.99) | 2.14(0.88,3.49) | 3.08(1.20,5.16) | 1.04(0.57,1.50) |
| Barbados | both | 35.02(15.88,53.71) | 50.36(22.14,80.49) | 1.01(0.81,1.21) | 11.41(5.15,17.51) | 9.67(4.22,15.39) | -0.57(-0.84,-0.29) |
| Belarus | both | 36.47(15.78,59.86) | 59.92(24.55,97.72) | -0.08(-1.16,1.01) | 0.29(0.12,0.46) | 0.36(0.15,0.59) | -0.89(-1.93,0.17) |
| Belgium | both | 297.00(120.42,467.83) | 293.43(133.03,476.04) | 0.25(0.07,0.42) | 1.85(0.75,2.93) | 0.94(0.43,1.51) | -2.06(-2.29,-1.84) |
| Belize | both | 4.88(2.02,7.60) | 16.83(7.13,27.31) | 4.16(3.76,4.57) | 5.37(2.22,8.37) | 6.31(2.68,10.28) | 0.56(-0.02,1.14) |
| Benin | both | 20.84(7.83,37.06) | 64.95(24.69,119.02) | 3.82(3.74,3.89) | 1.29(0.48,2.31) | 1.77(0.63,3.22) | 1.02(0.87,1.17) |
| Bermuda | both | 2.55(1.16,3.94) | 3.69(1.55,5.87) | 1.25(1.00,1.49) | 4.47(2.04,6.98) | 2.39(1.01,3.79) | -2.09(-2.24,-1.94) |
| Bhutan | both | 8.74(3.71,14.28) | 33.30(14.60,55.68) | 4.54(4.47,4.60) | 4.63(1.98,7.64) | 6.06(2.69,10.13) | 0.90(0.88,0.93) |
| Bolivia (Plurinational State of) | both | 111.31(42.11,180.62) | 371.49(143.39,630.53) | 4.31(4.16,4.45) | 4.19(1.61,6.76) | 4.78(1.85,8.05) | 0.53(0.48,0.57) |
| Bosnia and Herzegovina | both | 46.05(19.52,74.48) | 189.20(78.67,316.76) | 5.49(4.99,6.00) | 1.36(0.57,2.21) | 2.88(1.21,4.77) | 3.06(2.72,3.39) |
| Botswana | both | 32.84(13.82,54.46) | 93.30(38.28,148.06) | 3.67(3.36,3.98) | 7.43(3.15,12.32) | 8.20(3.37,12.95) | 0.68(0.35,1.02) |
| Brazil | both | 3496.57(1544.73,5270.26) | 10212.34(4153.71,16027.17) | 3.63(3.45,3.81) | 4.75(2.09,7.22) | 4.24(1.72,6.67) | -0.27(-0.43,-0.11) |
| Brunei Darussalam | both | 7.40(2.94,12.13) | 14.94(5.97,24.27) | 2.53(2.41,2.65) | 9.33(3.80,15.36) | 6.20(2.50,9.78) | -0.91(-1.13,-0.69) |
| Bulgaria | both | 144.32(57.70,230.55) | 232.67(96.60,382.45) | 1.34(0.97,1.72) | 1.34(0.53,2.15) | 1.54(0.63,2.53) | 0.25(-0.08,0.57) |
| Burkina Faso | both | 74.28(28.93,120.06) | 152.96(59.23,256.37) | 2.32(2.23,2.42) | 2.34(0.90,3.89) | 2.25(0.83,3.83) | -0.18(-0.25,-0.11) |
| Burundi | both | 48.32(19.21,84.77) | 75.84(26.85,135.09) | 1.01(0.66,1.36) | 2.59(1.00,4.53) | 2.28(0.85,4.16) | -0.88(-1.06,-0.70) |
| Cabo Verde | both | 1.73(0.68,2.85) | 9.53(3.70,17.60) | 5.37(4.88,5.86) | 0.72(0.28,1.20) | 2.33(0.90,4.34) | 3.43(2.97,3.89) |
| Cambodia | both | 37.17(13.24,64.22) | 120.27(41.25,218.12) | 3.84(3.51,4.18) | 1.15(0.41,2.01) | 1.36(0.45,2.44) | 0.43(0.15,0.70) |
| Cameroon | both | 113.89(42.86,193.35) | 405.47(157.42,701.56) | 4.16(4.03,4.30) | 3.43(1.29,5.81) | 4.45(1.69,7.50) | 0.77(0.60,0.94) |
| Canada | both | 319.72(119.51,550.26) | 556.16(208.92,984.15) | 1.44(0.85,2.03) | 1.01(0.37,1.74) | 0.65(0.25,1.15) | -1.81(-2.39,-1.23) |
| Central African Republic | both | 31.21(13.14,52.67) | 57.55(21.72,101.58) | 1.94(1.87,2.01) | 3.75(1.55,6.47) | 3.74(1.41,6.74) | -0.03(-0.11,0.06) |
| Chad | both | 43.19(15.99,71.66) | 109.18(40.11,184.59) | 2.99(2.82,3.16) | 1.82(0.69,3.04) | 2.48(0.93,4.23) | 0.96(0.71,1.22) |
| Chile | both | 126.92(54.34,215.59) | 305.01(113.46,517.52) | 3.04(2.66,3.43) | 1.40(0.59,2.41) | 1.15(0.43,1.95) | -0.44(-0.85,-0.03) |
| China | both | 8570.99(3588.98,13780.37) | 25807.14(11234.59,41523.47) | 3.55(3.42,3.68) | 1.44(0.60,2.32) | 1.37(0.60,2.21) | -0.20(-0.39,0.00) |
| Colombia | both | 386.72(165.29,604.35) | 932.93(408.06,1483.11) | 2.32(2.04,2.60) | 2.46(1.05,3.88) | 1.67(0.73,2.65) | -1.87(-2.14,-1.61) |
| Comoros | both | 2.82(1.10,4.71) | 7.99(2.94,14.11) | 3.34(3.21,3.47) | 1.88(0.72,3.15) | 2.03(0.78,3.63) | 0.11(0.01,0.20) |
| Congo | both | 32.75(12.89,54.02) | 77.52(28.67,132.10) | 2.58(2.33,2.84) | 3.93(1.56,6.62) | 3.85(1.35,6.86) | -0.27(-0.40,-0.14) |
| Cook Islands | both | 1.42(0.57,2.30) | 2.66(1.13,4.41) | 1.88(1.77,1.99) | 12.76(5.09,20.54) | 10.34(4.41,17.19) | -0.87(-0.99,-0.74) |
| Costa Rica | both | 22.44(8.85,36.12) | 105.01(43.32,171.60) | 4.51(3.97,5.04) | 1.37(0.54,2.20) | 1.90(0.79,3.10) | 0.43(-0.11,0.97) |
| Croatia | both | 76.55(32.45,117.79) | 200.99(87.82,329.86) | 2.68(2.20,3.16) | 1.47(0.61,2.28) | 1.93(0.85,3.17) | 0.15(-0.39,0.69) |
| Cuba | both | 252.77(103.74,395.36) | 335.22(139.58,539.00) | 1.06(0.50,1.63) | 2.56(1.05,3.97) | 1.62(0.68,2.60) | -1.37(-1.92,-0.81) |
| Cyprus | both | 57.25(25.24,90.13) | 70.56(29.55,113.75) | 0.21(0.02,0.41) | 11.00(4.69,17.21) | 4.22(1.76,6.89) | -3.40(-3.63,-3.17) |
| Czechia | both | 213.31(85.05,343.25) | 542.69(230.24,853.67) | 5.13(4.07,6.19) | 1.53(0.61,2.44) | 2.25(0.95,3.56) | 3.11(2.22,4.01) |
| Côte d'Ivoire | both | 68.40(26.06,117.87) | 257.75(99.66,451.93) | 4.22(3.95,4.49) | 2.55(1.01,4.44) | 3.23(1.21,5.75) | 0.73(0.52,0.94) |
| Democratic People's Republic of Korea | both | 230.47(92.13,403.16) | 531.36(219.14,879.32) | 2.96(2.76,3.17) | 1.88(0.75,3.32) | 1.76(0.73,2.91) | -0.05(-0.23,0.13) |
| Democratic Republic of the Congo | both | 596.99(238.45,978.54) | 1352.48(541.30,2358.13) | 2.43(2.27,2.59) | 5.83(2.27,9.43) | 5.48(2.20,9.46) | -0.31(-0.45,-0.17) |
| Denmark | both | 98.47(42.20,152.72) | 218.13(96.82,356.89) | 2.55(2.09,3.02) | 1.12(0.47,1.73) | 1.54(0.69,2.53) | 1.09(0.57,1.62) |
| Djibouti | both | 1.58(0.57,2.82) | 10.21(3.72,18.32) | 6.23(6.13,6.33) | 1.90(0.71,3.35) | 2.62(0.91,4.79) | 1.02(0.93,1.12) |
| Dominica | both | 3.97(1.59,6.41) | 5.50(2.32,9.12) | 0.79(0.68,0.89) | 6.81(2.72,10.90) | 7.30(3.08,12.23) | -0.02(-0.13,0.10) |
| Dominican Republic | both | 86.75(35.32,136.59) | 325.26(137.07,529.51) | 4.75(4.43,5.07) | 2.84(1.17,4.57) | 3.36(1.41,5.46) | 1.13(0.86,1.40) |
| Ecuador | both | 96.93(40.84,159.33) | 419.75(169.35,710.29) | 4.93(4.20,5.67) | 2.07(0.86,3.42) | 2.74(1.08,4.65) | 0.92(0.23,1.61) |
| Egypt | both | 936.84(394.27,1437.33) | 3372.34(1438.04,5516.27) | 4.69(4.47,4.90) | 4.89(2.01,7.61) | 7.62(3.24,12.40) | 2.17(1.85,2.48) |
| El Salvador | both | 55.08(22.97,90.25) | 267.86(106.31,464.75) | 5.24(4.83,5.66) | 1.95(0.81,3.22) | 4.14(1.68,7.21) | 2.41(2.06,2.77) |
| Equatorial Guinea | both | 5.75(2.31,9.73) | 19.78(7.20,34.08) | 4.58(4.13,5.04) | 4.01(1.60,6.84) | 5.52(1.98,9.59) | 1.28(1.02,1.55) |
| Eritrea | both | 8.47(3.08,15.53) | 26.49(9.85,48.22) | 4.17(4.03,4.31) | 1.21(0.42,2.30) | 1.50(0.55,2.82) | 0.77(0.70,0.84) |
| Estonia | both | 6.55(2.79,10.41) | 37.16(15.52,59.84) | 5.22(4.39,6.07) | 0.32(0.14,0.51) | 1.12(0.46,1.79) | 3.39(2.62,4.15) |
| Eswatini | both | 16.22(6.73,27.57) | 49.01(19.01,84.13) | 3.90(3.17,4.64) | 7.23(3.12,12.21) | 11.06(4.36,19.31) | 1.80(1.15,2.46) |
| Ethiopia | both | 546.55(216.11,907.51) | 813.82(308.66,1390.64) | 0.85(0.55,1.16) | 3.64(1.36,6.21) | 2.38(0.92,4.15) | -1.82(-2.02,-1.62) |
| Fiji | both | 51.87(21.81,84.50) | 165.32(72.87,273.83) | 3.83(3.56,4.09) | 16.38(6.78,27.04) | 25.07(11.21,41.92) | 1.18(0.86,1.49) |
| Finland | both | 64.50(28.18,102.67) | 92.35(38.61,151.50) | 1.11(0.70,1.53) | 0.88(0.39,1.41) | 0.56(0.24,0.91) | -1.50(-1.85,-1.15) |
| France | both | 1362.44(599.76,2181.02) | 2360.54(985.83,3823.11) | 1.97(1.56,2.37) | 1.50(0.66,2.41) | 1.16(0.49,1.86) | -0.73(-1.25,-0.21) |
| Gabon | both | 12.80(4.66,22.18) | 25.97(9.42,46.57) | 2.17(2.01,2.32) | 2.85(1.01,4.98) | 3.63(1.29,6.63) | 0.63(0.42,0.84) |
| Gambia | both | 4.49(1.77,7.59) | 21.68(8.28,38.93) | 5.21(4.93,5.49) | 1.82(0.72,3.08) | 2.86(1.11,5.15) | 1.37(1.24,1.51) |
| Georgia | both | 43.12(18.18,69.11) | 91.98(37.25,150.09) | 3.81(3.04,4.58) | 0.72(0.30,1.15) | 1.44(0.58,2.37) | 3.58(2.83,4.32) |
| Germany | both | 2580.42(1077.89,4025.42) | 3082.07(1326.90,5175.43) | 0.51(0.30,0.71) | 1.87(0.78,2.95) | 1.20(0.51,2.02) | -1.60(-1.78,-1.42) |
| Ghana | both | 119.05(44.23,195.45) | 533.57(207.15,877.57) | 5.37(5.19,5.55) | 2.72(1.03,4.53) | 4.50(1.77,7.45) | 1.95(1.79,2.10) |
| Greece | both | 244.00(105.86,394.65) | 467.43(194.97,783.59) | 1.92(1.18,2.65) | 1.72(0.74,2.79) | 1.37(0.58,2.31) | -1.06(-1.70,-0.42) |
| Greenland | both | 0.33(0.13,0.53) | 0.45(0.19,0.74) | 1.05(0.84,1.26) | 1.50(0.63,2.49) | 0.89(0.37,1.45) | -1.77(-1.90,-1.64) |
| Grenada | both | 7.29(2.96,12.15) | 8.65(3.53,14.05) | 0.99(0.73,1.25) | 8.96(3.64,14.86) | 9.16(3.84,14.75) | 0.50(0.17,0.82) |
| Guam | both | 2.16(0.92,3.48) | 3.44(1.43,5.73) | 1.79(1.57,2.01) | 4.18(1.72,6.84) | 1.58(0.66,2.64) | -2.60(-2.95,-2.25) |
| Guatemala | both | 33.35(12.88,55.90) | 313.48(120.78,543.73) | 7.22(6.81,7.62) | 1.51(0.59,2.59) | 3.19(1.23,5.60) | 1.88(1.48,2.29) |
| Guinea | both | 42.21(16.36,73.00) | 92.06(32.21,162.19) | 2.60(2.23,2.97) | 1.57(0.60,2.77) | 2.11(0.75,3.75) | 1.05(0.89,1.21) |
| Guinea-Bissau | both | 10.15(3.86,17.56) | 21.71(8.16,35.94) | 2.55(2.48,2.62) | 3.39(1.27,5.72) | 4.42(1.68,7.40) | 0.92(0.79,1.06) |
| Guyana | both | 25.61(10.61,40.29) | 51.24(21.34,84.49) | 2.31(1.98,2.64) | 7.79(3.24,12.20) | 9.22(3.92,15.11) | 0.42(-0.01,0.86) |
| Haiti | both | 233.12(89.72,380.10) | 490.96(194.31,842.49) | 2.55(2.47,2.63) | 8.88(3.34,14.31) | 8.55(3.42,14.59) | -0.03(-0.09,0.03) |
| Honduras | both | 20.16(7.60,33.86) | 130.27(55.11,220.60) | 6.42(6.16,6.68) | 1.14(0.43,1.90) | 2.36(1.02,4.01) | 2.47(2.19,2.75) |
| Hungary | both | 173.54(70.60,279.49) | 309.57(127.40,482.02) | 2.40(2.01,2.80) | 1.19(0.48,1.91) | 1.40(0.58,2.18) | 0.90(0.53,1.28) |
| Iceland | both | 2.24(0.95,3.58) | 4.62(1.89,7.47) | 2.57(2.45,2.68) | 0.72(0.30,1.15) | 0.66(0.27,1.07) | -0.09(-0.24,0.06) |
| India | both | 8723.82(3690.90,13300.88) | 35008.87(15169.20,54415.19) | 4.75(4.55,4.95) | 2.62(1.10,4.05) | 3.63(1.57,5.63) | 1.21(0.96,1.47) |
| Indonesia | both | 3044.80(1268.60,4747.98) | 10366.86(4627.09,15966.67) | 3.85(3.73,3.97) | 3.78(1.60,5.97) | 5.49(2.46,8.42) | 1.20(1.11,1.29) |
| Iran (Islamic Republic of) | both | 381.80(168.94,603.05) | 1868.09(796.45,2907.66) | 5.96(5.73,6.19) | 2.11(0.89,3.38) | 2.78(1.21,4.36) | 1.27(1.07,1.47) |
| Iraq | both | 459.21(188.65,751.30) | 1408.47(607.30,2294.34) | 3.56(3.25,3.86) | 6.20(2.54,10.21) | 7.39(3.17,11.99) | 0.23(-0.02,0.47) |
| Ireland | both | 70.27(30.92,107.16) | 86.68(36.86,140.56) | 0.99(0.81,1.18) | 1.78(0.78,2.71) | 0.99(0.42,1.61) | -1.53(-1.74,-1.33) |
| Israel | both | 131.69(57.64,207.29) | 351.90(143.74,581.00) | 2.75(1.88,3.62) | 2.94(1.29,4.69) | 2.46(1.00,4.04) | -0.96(-1.79,-0.13) |
| Italy | both | 2684.87(1175.27,4186.09) | 3469.31(1486.62,5501.69) | 1.15(1.02,1.28) | 2.92(1.28,4.55) | 1.71(0.74,2.69) | -1.51(-1.62,-1.41) |
| Jamaica | both | 174.81(74.94,269.34) | 313.65(136.23,503.85) | 1.63(1.33,1.92) | 9.43(4.05,14.48) | 9.68(4.24,15.57) | -0.20(-0.48,0.07) |
| Japan | both | 2025.71(870.96,3212.15) | 3667.31(1505.24,6611.69) | 2.17(1.81,2.53) | 1.29(0.54,2.08) | 0.65(0.27,1.14) | -2.05(-2.41,-1.68) |
| Jordan | both | 71.84(30.11,115.44) | 299.56(120.66,486.99) | 4.19(3.89,4.48) | 7.18(3.04,11.37) | 5.63(2.36,9.16) | -1.23(-1.68,-0.78) |
| Kazakhstan | both | 61.65(24.88,100.33) | 123.64(49.74,206.93) | 1.12(0.61,1.63) | 0.51(0.21,0.84) | 0.80(0.33,1.33) | 0.43(-0.05,0.92) |
| Kenya | both | 127.96(53.23,206.43) | 485.65(188.28,819.16) | 4.68(4.57,4.78) | 1.93(0.80,3.17) | 2.82(1.06,4.62) | 1.51(1.40,1.62) |
| Kiribati | both | 6.06(2.56,9.64) | 15.31(6.39,24.84) | 3.06(2.95,3.16) | 18.09(7.53,29.39) | 24.18(10.16,40.07) | 0.88(0.69,1.06) |
| Kuwait | both | 19.47(8.77,29.76) | 95.42(40.82,151.30) | 5.04(4.52,5.55) | 4.17(1.91,6.49) | 4.37(1.86,6.95) | -0.07(-0.58,0.45) |
| Kyrgyzstan | both | 9.67(3.85,15.80) | 25.38(10.35,42.35) | 1.95(1.49,2.42) | 0.35(0.14,0.56) | 0.62(0.26,1.01) | 0.95(0.49,1.41) |
| Lao People's Democratic Republic | both | 35.86(13.41,61.90) | 72.69(28.30,127.66) | 1.98(1.81,2.15) | 2.11(0.78,3.65) | 2.00(0.78,3.55) | -0.39(-0.49,-0.29) |
| Latvia | both | 14.87(6.12,23.68) | 51.29(20.84,87.03) | 4.07(3.48,4.66) | 0.42(0.17,0.66) | 1.14(0.47,1.91) | 3.21(2.62,3.79) |
| Lebanon | both | 89.86(37.04,152.45) | 280.04(116.51,463.57) | 4.36(4.09,4.63) | 4.96(2.01,8.21) | 4.27(1.76,7.09) | -0.18(-0.36,0.00) |
| Lesotho | both | 17.05(6.82,29.44) | 40.48(15.39,71.62) | 3.37(2.72,4.02) | 2.54(1.02,4.27) | 5.38(2.18,9.57) | 3.37(2.76,3.98) |
| Liberia | both | 31.92(13.05,53.28) | 78.00(32.09,132.71) | 3.04(2.89,3.20) | 3.33(1.38,5.53) | 4.57(1.91,7.90) | 1.21(1.05,1.37) |
| Libya | both | 37.12(15.63,61.01) | 174.37(71.92,291.85) | 5.96(5.68,6.24) | 2.22(0.93,3.66) | 3.93(1.65,6.43) | 2.77(2.43,3.10) |
| Lithuania | both | 14.16(6.03,22.61) | 66.41(27.99,107.98) | 4.33(3.44,5.23) | 0.32(0.13,0.50) | 1.01(0.42,1.64) | 2.95(2.03,3.88) |
| Luxembourg | both | 9.10(3.66,14.71) | 11.91(5.06,19.53) | 0.95(0.82,1.08) | 1.73(0.69,2.80) | 0.95(0.40,1.55) | -1.88(-2.04,-1.71) |
| Madagascar | both | 67.39(25.11,116.56) | 133.46(53.83,234.71) | 2.22(1.97,2.47) | 1.73(0.65,3.01) | 1.86(0.74,3.33) | 0.18(0.12,0.24) |
| Malawi | both | 58.18(22.39,100.05) | 127.74(50.22,225.24) | 2.24(2.07,2.41) | 1.99(0.80,3.42) | 2.26(0.84,4.09) | 0.20(-0.02,0.42) |
| Malaysia | both | 275.97(117.83,432.26) | 725.54(299.09,1150.94) | 2.93(2.69,3.18) | 3.32(1.42,5.21) | 2.85(1.17,4.59) | -0.74(-0.95,-0.54) |
| Maldives | both | 5.77(2.47,8.82) | 10.89(4.81,17.06) | 1.63(1.34,1.91) | 7.51(3.17,11.64) | 3.88(1.75,6.10) | -2.49(-2.69,-2.28) |
| Mali | both | 89.33(39.25,143.43) | 243.60(96.96,421.03) | 3.66(3.51,3.81) | 3.01(1.32,5.07) | 3.67(1.40,6.12) | 0.74(0.65,0.82) |
| Malta | both | 15.23(6.69,23.28) | 25.39(10.80,39.59) | 1.64(1.31,1.96) | 3.87(1.71,5.93) | 2.21(0.94,3.47) | -1.81(-2.13,-1.48) |
| Marshall Islands | both | 2.79(1.20,4.40) | 9.69(3.88,16.24) | 4.13(3.95,4.31) | 18.99(8.25,29.80) | 30.46(11.94,50.28) | 1.44(1.14,1.73) |
| Mauritania | both | 36.90(13.82,60.50) | 96.28(37.81,161.60) | 2.99(2.87,3.11) | 4.36(1.66,7.21) | 5.31(2.08,8.90) | 0.39(0.29,0.49) |
| Mauritius | both | 27.01(11.35,43.31) | 168.16(71.04,267.25) | 7.51(6.56,8.48) | 4.13(1.72,6.50) | 9.50(4.00,15.05) | 4.02(3.07,4.99) |
| Mexico | both | 2167.94(874.01,3361.17) | 6863.88(2844.94,11047.50) | 3.91(3.63,4.19) | 6.06(2.41,9.38) | 5.81(2.41,9.43) | -0.09(-0.34,0.16) |
| Micronesia (Federated States of) | both | 7.39(3.10,11.75) | 14.82(6.21,23.63) | 2.23(2.06,2.41) | 16.51(7.04,26.12) | 22.40(9.35,36.06) | 0.96(0.67,1.26) |
| Monaco | both | 0.59(0.24,0.97) | 1.05(0.40,1.76) | 1.98(1.76,2.21) | 0.69(0.28,1.14) | 0.82(0.32,1.35) | 0.72(0.57,0.87) |
| Mongolia | both | 3.36(1.28,6.07) | 8.92(3.46,15.46) | 3.06(2.69,3.43) | 0.36(0.14,0.63) | 0.48(0.19,0.82) | 0.83(0.71,0.95) |
| Montenegro | both | 7.46(2.94,12.19) | 18.43(7.40,29.55) | 3.28(3.12,3.45) | 1.29(0.52,2.13) | 2.06(0.82,3.31) | 1.64(1.37,1.91) |
| Morocco | both | 268.86(105.38,452.15) | 1178.34(482.62,1896.93) | 5.31(5.09,5.54) | 2.14(0.84,3.70) | 3.87(1.56,6.20) | 2.30(2.12,2.48) |
| Mozambique | both | 77.18(29.46,133.57) | 182.08(66.83,318.81) | 3.25(3.09,3.41) | 1.80(0.68,3.13) | 2.37(0.86,4.13) | 1.34(1.18,1.51) |
| Myanmar | both | 339.05(127.88,601.18) | 809.43(299.28,1453.33) | 2.61(2.40,2.82) | 1.79(0.73,3.14) | 1.99(0.72,3.57) | 0.15(0.01,0.29) |
| Namibia | both | 30.52(12.95,48.64) | 81.94(31.95,133.63) | 2.86(2.49,3.23) | 5.93(2.43,9.68) | 7.50(2.96,12.16) | 0.53(0.21,0.86) |
| Nauru | both | 0.42(0.16,0.71) | 0.75(0.29,1.27) | 1.56(1.33,1.78) | 10.30(3.88,17.12) | 14.66(5.89,24.63) | 1.03(0.86,1.19) |
| Nepal | both | 103.35(39.99,170.08) | 439.13(171.23,776.13) | 5.11(4.82,5.41) | 1.52(0.58,2.58) | 2.38(0.93,4.33) | 1.55(1.28,1.82) |
| Netherlands | both | 376.06(143.77,609.64) | 415.91(169.47,692.32) | 0.30(0.01,0.59) | 1.83(0.70,2.95) | 1.01(0.41,1.68) | -1.97(-2.29,-1.65) |
| New Zealand | both | 52.14(22.38,80.19) | 116.20(48.44,188.26) | 2.05(1.57,2.54) | 1.34(0.58,2.05) | 1.26(0.53,2.04) | -0.70(-1.16,-0.24) |
| Nicaragua | both | 26.54(10.91,44.63) | 119.60(45.44,209.75) | 5.07(4.71,5.44) | 2.00(0.79,3.32) | 2.70(1.01,4.74) | 1.17(0.89,1.45) |
| Niger | both | 36.23(13.70,61.14) | 132.36(51.39,229.32) | 4.33(4.25,4.41) | 1.86(0.69,3.04) | 2.26(0.88,3.91) | 0.63(0.54,0.72) |
| Nigeria | both | 1089.41(467.68,1768.39) | 2265.39(880.77,3649.13) | 2.24(2.12,2.35) | 3.13(1.32,5.03) | 3.38(1.28,5.47) | 0.16(0.12,0.19) |
| Niue | both | 0.19(0.07,0.32) | 0.27(0.11,0.45) | 1.06(0.98,1.14) | 8.27(3.25,13.41) | 13.08(5.44,21.60) | 1.40(1.25,1.56) |
| North Macedonia | both | 31.12(12.88,51.86) | 89.48(38.45,149.58) | 3.79(3.39,4.20) | 1.92(0.79,3.15) | 3.34(1.42,5.57) | 1.89(1.37,2.43) |
| Northern Mariana Islands | both | 0.98(0.39,1.61) | 3.16(1.39,4.97) | 4.11(3.77,4.45) | 7.63(3.08,12.78) | 7.34(3.25,11.55) | -0.18(-0.42,0.06) |
| Norway | both | 66.71(27.25,107.83) | 106.43(42.50,168.80) | 1.43(0.93,1.93) | 0.86(0.35,1.39) | 0.85(0.34,1.35) | -0.07(-0.57,0.43) |
| Oman | both | 29.67(12.52,50.41) | 94.79(39.44,152.85) | 4.15(3.84,4.46) | 5.52(2.36,9.27) | 7.10(2.86,11.51) | 1.36(1.04,1.68) |
| Pakistan | both | 1306.80(554.54,2100.96) | 4199.81(1769.92,6748.67) | 3.49(3.28,3.70) | 2.78(1.16,4.51) | 4.51(1.87,7.38) | 1.32(1.04,1.60) |
| Palau | both | 0.71(0.29,1.17) | 2.04(0.84,3.35) | 3.31(3.08,3.53) | 8.24(3.48,13.73) | 11.11(4.48,18.18) | 1.15(0.99,1.31) |
| Palestine | both | 55.26(23.52,88.54) | 142.99(59.48,221.64) | 3.27(3.12,3.43) | 7.78(3.33,12.47) | 7.92(3.26,12.25) | 0.15(-0.10,0.40) |
| Panama | both | 24.04(10.01,38.36) | 112.55(48.98,189.34) | 5.02(4.70,5.34) | 1.76(0.74,2.83) | 2.48(1.08,4.20) | 1.02(0.71,1.33) |
| Papua New Guinea | both | 100.72(38.83,174.55) | 287.48(109.68,499.75) | 3.40(3.27,3.53) | 6.39(2.50,10.80) | 6.54(2.43,11.15) | 0.02(-0.02,0.07) |
| Paraguay | both | 47.86(17.87,81.33) | 236.54(89.03,405.98) | 5.64(5.31,5.98) | 2.41(0.91,4.08) | 4.43(1.66,7.59) | 2.36(2.08,2.63) |
| Peru | both | 213.45(87.46,353.62) | 742.39(331.40,1245.10) | 4.07(3.81,4.34) | 2.01(0.82,3.31) | 2.25(1.00,3.75) | 0.06(-0.21,0.33) |
| Philippines | both | 421.90(162.35,693.51) | 1844.82(756.08,2968.84) | 5.02(4.96,5.09) | 1.95(0.75,3.27) | 2.69(1.11,4.42) | 1.32(1.24,1.39) |
| Poland | both | 661.05(272.39,1043.93) | 1218.74(516.68,1926.77) | 2.25(1.90,2.59) | 1.53(0.63,2.41) | 1.55(0.66,2.44) | 0.10(-0.13,0.33) |
| Portugal | both | 427.65(181.11,652.11) | 720.86(302.50,1147.65) | 1.42(1.03,1.81) | 3.24(1.39,4.94) | 2.19(0.91,3.47) | -1.70(-2.12,-1.27) |
| Puerto Rico | both | 239.80(99.13,365.82) | 418.84(174.66,679.06) | 1.54(1.27,1.81) | 7.11(2.97,10.97) | 4.88(2.05,7.87) | -1.40(-1.60,-1.20) |
| Qatar | both | 7.08(2.84,11.15) | 53.37(22.16,88.91) | 7.34(6.83,7.84) | 12.23(5.02,19.30) | 12.62(5.31,20.63) | 0.03(-0.90,0.96) |
| Republic of Korea | both | 573.05(245.86,911.23) | 1471.63(592.08,2461.81) | 2.67(2.15,3.19) | 2.51(1.11,4.06) | 1.60(0.64,2.69) | -1.92(-2.51,-1.32) |
| Republic of Moldova | both | 18.67(7.79,30.19) | 41.93(17.03,68.16) | 1.69(0.72,2.67) | 0.47(0.20,0.76) | 0.69(0.28,1.11) | 0.37(-0.58,1.34) |
| Romania | both | 209.08(86.39,330.13) | 354.20(145.50,561.84) | 2.04(1.58,2.52) | 0.82(0.34,1.30) | 0.86(0.36,1.36) | 0.40(-0.06,0.86) |
| Russian Federation | both | 592.42(243.29,893.67) | 3944.62(1604.47,6264.11) | 5.49(3.74,7.28) | 0.34(0.14,0.51) | 1.60(0.64,2.54) | 4.40(2.70,6.13) |
| Rwanda | both | 54.13(20.94,94.06) | 93.37(32.80,178.31) | 1.21(0.62,1.80) | 2.61(0.98,4.55) | 2.17(0.76,4.17) | -1.41(-1.75,-1.07) |
| Saint Kitts and Nevis | both | 3.28(1.43,5.26) | 3.67(1.51,6.07) | 0.82(0.52,1.12) | 8.81(3.89,13.96) | 6.88(2.81,11.11) | -0.22(-0.52,0.07) |
| Saint Lucia | both | 8.28(3.38,13.30) | 15.62(6.43,25.60) | 1.25(0.88,1.63) | 10.92(4.50,17.47) | 6.79(2.77,11.12) | -2.64(-3.03,-2.26) |
| Saint Vincent and the Grenadines | both | 7.28(3.17,11.79) | 10.15(4.15,16.63) | 0.87(0.55,1.18) | 11.08(4.74,17.78) | 7.80(3.19,12.92) | -1.40(-1.72,-1.09) |
| Samoa | both | 7.14(3.20,11.47) | 17.59(7.49,28.40) | 2.96(2.87,3.04) | 9.31(4.15,14.94) | 13.16(5.53,21.35) | 1.12(1.05,1.20) |
| San Marino | both | 0.44(0.18,0.71) | 0.64(0.24,1.13) | 2.14(1.64,2.65) | 1.15(0.46,1.86) | 0.59(0.23,1.02) | -1.24(-1.62,-0.86) |
| Sao Tome and Principe | both | 0.67(0.25,1.18) | 1.29(0.46,2.24) | 2.11(2.06,2.15) | 1.26(0.47,2.28) | 1.58(0.56,2.73) | 0.77(0.69,0.86) |
| Saudi Arabia | both | 208.61(83.47,340.49) | 914.56(367.11,1482.33) | 4.45(4.24,4.65) | 4.42(1.80,7.11) | 6.38(2.71,10.38) | 0.92(0.73,1.10) |
| Senegal | both | 86.61(36.04,140.80) | 279.18(112.91,474.94) | 3.91(3.80,4.03) | 3.29(1.35,5.27) | 4.46(1.76,7.70) | 0.90(0.82,0.98) |
| Serbia | both | 183.99(77.51,288.46) | 392.69(160.75,642.47) | 2.63(2.41,2.85) | 2.21(0.93,3.51) | 2.19(0.89,3.58) | -0.12(-0.28,0.04) |
| Seychelles | both | 1.20(0.50,1.90) | 3.34(1.34,5.49) | 3.68(3.51,3.86) | 2.13(0.90,3.38) | 3.41(1.36,5.57) | 1.84(1.62,2.05) |
| Sierra Leone | both | 34.74(13.94,58.25) | 79.53(31.16,134.81) | 2.73(2.64,2.81) | 2.07(0.82,3.47) | 2.72(1.04,4.58) | 1.02(0.79,1.25) |
| Singapore | both | 37.92(15.85,60.95) | 44.46(18.49,74.48) | 0.69(0.19,1.19) | 2.02(0.84,3.32) | 0.54(0.23,0.91) | -3.89(-4.47,-3.31) |
| Slovakia | both | 62.80(26.12,101.83) | 85.70(34.62,142.49) | 1.15(1.06,1.24) | 1.06(0.44,1.73) | 0.88(0.35,1.45) | -0.48(-0.55,-0.40) |
| Slovenia | both | 26.49(11.03,42.44) | 48.62(19.79,79.44) | 0.47(-0.15,1.09) | 1.07(0.45,1.73) | 0.90(0.36,1.48) | -2.29(-2.96,-1.61) |
| Solomon Islands | both | 10.95(4.40,18.86) | 36.37(14.29,60.06) | 3.87(3.77,3.97) | 9.18(3.81,15.79) | 11.69(4.63,19.29) | 0.79(0.71,0.87) |
| Somalia | both | 40.53(14.63,72.84) | 111.64(39.67,194.79) | 3.56(3.47,3.65) | 2.54(1.01,4.41) | 2.84(1.02,5.28) | 0.48(0.37,0.59) |
| South Africa | both | 1182.97(515.11,1822.79) | 4483.00(1890.20,6787.45) | 4.53(4.06,5.01) | 6.47(2.85,9.98) | 11.39(4.79,17.28) | 2.04(1.56,2.52) |
| South Sudan | both | 45.00(16.45,79.02) | 78.81(27.46,139.68) | 1.68(1.44,1.91) | 2.22(0.83,4.11) | 2.89(1.01,5.15) | 0.80(0.66,0.94) |
| Spain | both | 1423.80(601.51,2274.92) | 1660.41(676.24,2764.51) | 0.48(0.32,0.65) | 2.62(1.12,4.18) | 1.18(0.49,1.94) | -2.66(-2.81,-2.52) |
| Sri Lanka | both | 142.43(54.53,237.08) | 589.07(221.03,1038.71) | 5.33(5.07,5.58) | 1.73(0.67,3.06) | 2.39(0.91,4.27) | 1.80(1.51,2.10) |
| Sudan | both | 244.60(107.33,402.90) | 728.93(336.27,1215.72) | 3.62(3.39,3.84) | 2.90(1.27,4.77) | 4.13(1.88,6.82) | 1.32(1.12,1.52) |
| Suriname | both | 11.95(5.12,18.78) | 33.91(14.01,54.68) | 3.93(3.66,4.20) | 5.02(2.15,7.98) | 5.55(2.29,9.03) | 0.67(0.46,0.88) |
| Sweden | both | 161.54(65.89,257.32) | 274.81(113.38,458.94) | 1.88(1.63,2.13) | 0.94(0.39,1.50) | 0.97(0.40,1.60) | 0.28(0.04,0.51) |
| Switzerland | both | 210.63(85.92,325.66) | 206.12(82.66,345.14) | -0.29(-0.46,-0.12) | 1.81(0.74,2.79) | 0.83(0.33,1.36) | -2.69(-2.87,-2.52) |
| Syrian Arab Republic | both | 146.25(56.37,243.58) | 367.82(158.98,608.99) | 2.78(2.46,3.10) | 3.40(1.33,5.60) | 3.58(1.53,5.84) | -0.29(-0.55,-0.03) |
| Taiwan (Province of China) | both | 649.40(282.56,1027.76) | 1477.31(618.87,2392.23) | 1.91(1.46,2.36) | 4.99(2.14,7.86) | 3.33(1.40,5.39) | -2.06(-2.48,-1.64) |
| Tajikistan | both | 17.07(6.81,27.24) | 39.96(14.75,67.97) | 2.41(2.20,2.62) | 0.68(0.27,1.09) | 0.86(0.34,1.49) | 0.52(0.13,0.91) |
| Thailand | both | 689.84(261.78,1138.07) | 2675.84(1142.23,4436.33) | 3.89(3.67,4.11) | 2.46(0.95,4.09) | 2.46(1.05,4.08) | -0.61(-0.83,-0.38) |
| Timor-Leste | both | 2.12(0.79,3.77) | 8.53(3.04,15.00) | 5.07(4.81,5.33) | 1.07(0.39,1.89) | 1.17(0.41,2.07) | 0.39(0.12,0.66) |
| Togo | both | 13.04(5.04,22.80) | 53.03(19.23,91.75) | 4.55(4.48,4.61) | 1.54(0.57,2.71) | 2.21(0.83,3.91) | 1.10(0.97,1.23) |
| Tokelau | both | 0.10(0.04,0.17) | 0.13(0.05,0.21) | 0.91(0.78,1.03) | 7.35(3.11,12.61) | 8.59(3.51,14.15) | 0.46(0.33,0.60) |
| Tonga | both | 4.83(1.91,7.66) | 9.16(3.83,14.63) | 2.03(1.85,2.21) | 9.43(3.79,14.95) | 11.81(4.93,18.94) | 0.78(0.65,0.91) |
| Trinidad and Tobago | both | 125.05(54.09,188.13) | 252.46(111.51,415.17) | 2.08(1.91,2.26) | 16.42(7.15,24.55) | 13.11(5.76,21.67) | -0.90(-1.05,-0.75) |
| Tunisia | both | 56.45(22.39,92.72) | 272.84(116.56,469.54) | 5.35(5.22,5.48) | 1.52(0.61,2.46) | 2.35(1.02,4.06) | 1.59(1.46,1.72) |
| Türkiye | both | 1521.95(656.48,2386.81) | 3603.82(1479.96,5871.14) | 3.49(3.07,3.91) | 5.51(2.39,8.63) | 4.32(1.78,7.06) | -0.25(-0.68,0.19) |
| Turkmenistan | both | 11.36(4.61,18.33) | 52.95(20.04,87.32) | 4.44(4.06,4.83) | 0.67(0.27,1.08) | 1.49(0.58,2.43) | 1.93(1.56,2.30) |
| Tuvalu | both | 0.53(0.21,0.87) | 0.98(0.38,1.54) | 1.99(1.92,2.06) | 8.75(3.46,14.04) | 10.28(4.05,16.44) | 0.54(0.44,0.63) |
| Uganda | both | 96.05(33.57,176.61) | 256.81(88.36,475.24) | 2.80(2.62,2.99) | 1.98(0.71,3.67) | 2.46(0.85,4.49) | 0.35(0.12,0.58) |
| Ukraine | both | 120.57(47.87,196.51) | 146.17(53.82,249.01) | -0.66(-1.30,-0.02) | 0.16(0.07,0.27) | 0.18(0.07,0.30) | -0.90(-1.48,-0.32) |
| United Arab Emirates | both | 16.88(6.57,28.82) | 111.28(44.40,180.59) | 7.16(6.83,7.50) | 5.64(2.21,9.70) | 6.91(2.86,11.03) | 2.84(2.04,3.65) |
| United Kingdom | both | 1592.33(683.95,2446.56) | 1365.05(554.56,2080.03) | -0.26(-0.61,0.09) | 1.65(0.71,2.53) | 0.87(0.35,1.32) | -1.94(-2.27,-1.62) |
| United Republic of Tanzania | both | 69.93(26.93,118.96) | 182.34(65.48,342.77) | 2.82(2.68,2.95) | 0.87(0.32,1.52) | 0.95(0.33,1.78) | -0.03(-0.15,0.09) |
| United States of America | both | 5292.15(2253.27,8441.36) | 11149.88(4606.83,17752.87) | 1.96(1.68,2.25) | 1.56(0.67,2.50) | 1.77(0.74,2.79) | -0.05(-0.34,0.25) |
| United States Virgin Islands | both | 3.09(1.33,4.77) | 5.66(2.43,9.21) | 1.92(1.63,2.20) | 4.88(2.10,7.62) | 3.14(1.36,5.10) | -1.37(-1.60,-1.13) |
| Uruguay | both | 62.93(24.95,101.79) | 101.05(40.64,169.67) | 1.57(1.48,1.66) | 1.59(0.63,2.59) | 1.58(0.64,2.61) | 0.01(-0.08,0.11) |
| Uzbekistan | both | 40.67(15.55,68.78) | 239.03(102.98,414.35) | 5.41(5.02,5.79) | 0.38(0.15,0.64) | 1.02(0.43,1.74) | 2.96(2.49,3.44) |
| Vanuatu | both | 1.30(0.53,2.40) | 4.58(1.81,7.98) | 3.85(3.73,3.97) | 2.90(1.14,5.29) | 3.58(1.39,6.09) | 0.51(0.43,0.59) |
| Venezuela (Bolivarian Republic of) | both | 221.28(87.77,357.76) | 1012.72(418.46,1700.41) | 5.02(4.77,5.27) | 2.62(1.03,4.20) | 3.62(1.51,5.99) | 0.95(0.69,1.20) |
| Viet Nam | both | 718.77(284.49,1252.33) | 2087.59(824.53,3639.46) | 3.66(3.55,3.77) | 2.03(0.80,3.58) | 2.52(0.99,4.46) | 0.85(0.77,0.93) |
| Yemen | both | 78.76(30.51,139.40) | 259.08(99.92,442.61) | 4.03(3.87,4.18) | 2.05(0.81,3.60) | 2.29(0.90,3.89) | 0.38(0.24,0.52) |
| Zambia | both | 67.05(26.34,113.35) | 156.63(56.77,274.42) | 2.37(2.14,2.60) | 3.00(1.18,5.12) | 3.03(1.14,5.26) | -0.36(-0.53,-0.18) |
| Zimbabwe | both | 94.49(36.83,158.87) | 266.04(100.31,462.93) | 3.57(2.95,4.20) | 3.15(1.28,5.29) | 5.25(2.04,8.91) | 2.25(1.66,2.84) |
| Afghanistan | female | 174.81(67.14,289.18) | 407.33(171.76,685.78) | 2.80(2.71,2.90) | 5.89(2.25,9.63) | 9.35(3.90,15.32) | 1.70(1.61,1.79) |
| Albania | female | 6.51(2.72,10.91) | 17.09(7.09,28.84) | 3.71(3.38,4.05) | 0.67(0.28,1.12) | 0.73(0.30,1.23) | 0.74(0.41,1.07) |
| Algeria | female | 122.17(48.88,210.92) | 633.69(275.54,1036.96) | 5.75(5.56,5.95) | 3.37(1.38,5.76) | 5.21(2.21,8.50) | 2.39(2.01,2.77) |
| American Samoa | female | 1.29(0.56,2.01) | 5.01(2.22,8.14) | 4.29(3.75,4.83) | 13.22(5.66,20.87) | 22.55(10.17,36.84) | 1.66(1.28,2.05) |
| Andorra | female | 0.39(0.16,0.67) | 1.27(0.53,2.25) | 3.99(3.56,4.42) | 1.65(0.70,2.87) | 1.24(0.53,2.19) | -0.58(-0.75,-0.40) |
| Angola | female | 37.80(14.49,67.21) | 139.31(51.68,242.96) | 4.11(3.95,4.28) | 2.69(1.03,4.68) | 3.01(1.11,5.33) | 0.12(0.02,0.22) |
| Antigua and Barbuda | female | 2.88(1.22,4.51) | 4.52(1.86,7.01) | 0.97(0.73,1.21) | 8.30(3.57,13.15) | 8.65(3.57,13.50) | -0.35(-0.61,-0.08) |
| Argentina | female | 420.60(170.06,708.47) | 636.72(264.08,1072.16) | 1.45(1.07,1.83) | 2.38(0.96,3.97) | 1.78(0.74,2.97) | -0.87(-1.14,-0.59) |
| Armenia | female | 20.97(8.59,34.09) | 37.70(15.60,60.88) | 1.68(0.36,3.02) | 1.43(0.59,2.35) | 1.45(0.60,2.36) | -0.30(-1.47,0.89) |
| Australia | female | 200.40(83.65,312.40) | 423.26(172.36,686.46) | 2.48(2.14,2.82) | 1.74(0.73,2.72) | 1.37(0.56,2.17) | -0.75(-1.05,-0.46) |
| Austria | female | 185.66(78.13,288.48) | 257.93(112.06,445.66) | 2.15(1.63,2.68) | 2.12(0.90,3.33) | 1.68(0.73,2.86) | 0.15(-0.36,0.66) |
| Azerbaijan | female | 23.41(9.11,40.02) | 74.11(29.00,127.34) | 3.51(3.09,3.93) | 0.84(0.32,1.45) | 1.49(0.59,2.56) | 1.76(1.26,2.26) |
| Bahamas | female | 5.88(2.55,9.27) | 10.75(4.47,17.59) | 1.65(1.38,1.92) | 7.16(3.10,11.28) | 5.25(2.18,8.56) | -1.30(-1.56,-1.03) |
| Bahrain | female | 8.11(3.30,13.00) | 47.54(19.80,75.14) | 5.50(5.26,5.73) | 15.27(6.27,24.03) | 21.44(9.00,34.38) | 0.86(0.36,1.36) |
| Bangladesh | female | 548.52(223.37,906.30) | 2659.09(1092.00,4667.22) | 5.42(5.17,5.68) | 3.69(1.52,6.31) | 5.38(2.18,9.42) | 1.24(0.81,1.66) |
| Barbados | female | 26.17(11.54,41.13) | 35.43(16.00,56.00) | 0.94(0.72,1.17) | 14.15(6.25,22.01) | 11.98(5.41,19.01) | -0.43(-0.71,-0.15) |
| Belarus | female | 29.31(12.46,49.76) | 42.05(17.41,70.08) | -0.60(-1.68,0.50) | 0.34(0.15,0.57) | 0.36(0.15,0.61) | -1.55(-2.57,-0.52) |
| Belgium | female | 223.16(88.65,357.20) | 185.17(76.49,297.52) | -0.33(-0.52,-0.15) | 2.10(0.85,3.37) | 0.92(0.40,1.48) | -2.49(-2.73,-2.26) |
| Belize | female | 3.60(1.48,5.77) | 11.88(4.55,19.19) | 4.01(3.61,4.40) | 7.65(3.13,12.22) | 8.88(3.41,14.23) | 0.55(-0.02,1.12) |
| Benin | female | 11.94(4.42,21.32) | 42.42(15.76,79.39) | 4.33(4.22,4.43) | 1.42(0.53,2.51) | 2.02(0.74,3.80) | 1.17(1.01,1.32) |
| Bermuda | female | 1.73(0.77,2.72) | 2.38(0.99,3.95) | 0.77(0.48,1.06) | 4.97(2.21,7.83) | 2.54(1.08,4.24) | -2.53(-2.74,-2.31) |
| Bhutan | female | 4.97(2.02,8.11) | 18.61(7.47,31.07) | 4.39(4.35,4.44) | 4.91(2.07,8.09) | 6.71(2.69,11.20) | 0.95(0.90,1.00) |
| Bolivia (Plurinational State of) | female | 75.75(26.11,126.28) | 249.52(102.66,411.46) | 4.20(4.05,4.35) | 5.22(1.85,8.68) | 5.95(2.41,9.99) | 0.46(0.40,0.52) |
| Bosnia and Herzegovina | female | 34.12(14.52,56.55) | 132.37(52.22,224.91) | 5.25(4.78,5.72) | 1.70(0.70,2.83) | 3.39(1.33,5.69) | 2.81(2.50,3.12) |
| Botswana | female | 20.52(8.71,35.19) | 60.48(24.33,95.58) | 4.29(3.72,4.87) | 8.07(3.33,14.03) | 8.85(3.60,14.00) | 1.04(0.47,1.60) |
| Brazil | female | 2257.95(980.06,3480.76) | 6304.44(2530.39,9726.18) | 3.40(3.20,3.59) | 5.53(2.38,8.63) | 4.51(1.81,6.95) | -0.66(-0.83,-0.49) |
| Brunei Darussalam | female | 4.27(1.76,6.88) | 8.52(3.32,13.99) | 2.27(2.18,2.36) | 10.84(4.38,17.48) | 6.73(2.58,11.21) | -1.33(-1.59,-1.08) |
| Bulgaria | female | 100.95(43.62,161.60) | 160.14(67.58,271.24) | 1.21(0.81,1.60) | 1.66(0.68,2.61) | 1.71(0.71,2.88) | -0.22(-0.55,0.10) |
| Burkina Faso | female | 41.54(16.88,69.33) | 87.03(32.08,149.62) | 2.34(2.24,2.45) | 2.53(0.96,4.36) | 2.35(0.86,4.20) | -0.31(-0.40,-0.22) |
| Burundi | female | 28.98(10.71,53.52) | 43.84(15.38,78.57) | 0.78(0.46,1.11) | 2.81(1.01,5.08) | 2.63(0.90,4.93) | -0.74(-0.94,-0.54) |
| Cabo Verde | female | 1.22(0.50,2.14) | 7.45(2.92,14.24) | 5.77(5.16,6.38) | 0.88(0.36,1.56) | 2.87(1.12,5.43) | 3.51(2.97,4.06) |
| Cambodia | female | 26.80(9.30,51.58) | 89.97(28.57,166.27) | 3.92(3.57,4.28) | 1.37(0.45,2.63) | 1.61(0.51,2.98) | 0.37(0.08,0.65) |
| Cameroon | female | 66.97(26.96,115.57) | 244.35(97.76,423.78) | 4.31(4.17,4.45) | 3.92(1.55,6.92) | 5.06(2.02,8.60) | 0.81(0.63,0.99) |
| Canada | female | 216.65(76.71,384.76) | 346.02(120.65,601.88) | 1.20(0.59,1.81) | 1.08(0.38,1.91) | 0.68(0.24,1.17) | -1.90(-2.47,-1.34) |
| Central African Republic | female | 18.26(7.47,31.26) | 36.48(13.82,70.09) | 2.20(2.11,2.30) | 3.89(1.57,6.72) | 4.06(1.51,7.91) | 0.12(-0.01,0.25) |
| Chad | female | 25.70(9.21,44.46) | 64.60(25.14,114.06) | 3.00(2.83,3.18) | 2.08(0.74,3.64) | 3.22(1.25,5.88) | 1.45(1.19,1.70) |
| Chile | female | 88.98(37.00,150.12) | 209.23(81.33,352.67) | 2.94(2.54,3.33) | 1.73(0.70,2.93) | 1.35(0.52,2.26) | -0.65(-1.05,-0.25) |
| China | female | 5669.80(2336.02,9133.57) | 15056.90(6439.40,24894.25) | 3.05(2.89,3.20) | 1.63(0.68,2.66) | 1.41(0.60,2.33) | -0.59(-0.81,-0.37) |
| Colombia | female | 264.02(108.06,416.14) | 569.59(253.77,914.88) | 2.02(1.73,2.31) | 3.22(1.32,5.09) | 1.81(0.81,2.91) | -2.41(-2.68,-2.14) |
| Comoros | female | 1.84(0.71,3.40) | 5.59(2.00,10.47) | 3.63(3.55,3.72) | 2.33(0.89,4.37) | 2.53(0.93,4.83) | 0.18(0.12,0.24) |
| Congo | female | 16.65(6.60,28.84) | 41.69(15.08,74.91) | 2.78(2.63,2.92) | 3.58(1.27,6.27) | 3.90(1.35,7.20) | 0.13(0.01,0.25) |
| Cook Islands | female | 0.90(0.35,1.43) | 1.65(0.65,2.74) | 1.88(1.77,1.98) | 16.54(6.48,26.08) | 12.18(4.80,20.23) | -1.16(-1.26,-1.06) |
| Costa Rica | female | 15.53(6.31,26.46) | 67.77(26.36,114.27) | 4.20(3.59,4.81) | 1.82(0.75,3.09) | 2.20(0.87,3.69) | -0.08(-0.69,0.53) |
| Croatia | female | 56.75(24.34,88.99) | 144.73(63.70,242.32) | 2.48(1.97,2.98) | 1.66(0.70,2.62) | 2.16(0.96,3.56) | 0.05(-0.52,0.62) |
| Cuba | female | 190.12(79.08,301.38) | 230.88(98.51,382.79) | 0.75(0.17,1.33) | 3.77(1.58,5.96) | 2.03(0.87,3.35) | -1.86(-2.43,-1.28) |
| Cyprus | female | 36.94(15.23,59.63) | 41.06(16.22,68.48) | -0.05(-0.27,0.16) | 11.60(4.71,18.78) | 4.29(1.70,7.27) | -3.80(-4.14,-3.46) |
| Czechia | female | 156.47(62.88,252.67) | 355.51(153.07,561.89) | 4.71(3.72,5.71) | 1.74(0.70,2.80) | 2.35(1.01,3.69) | 2.78(1.94,3.62) |
| Côte d'Ivoire | female | 29.85(11.89,51.77) | 137.78(50.53,253.63) | 5.35(4.99,5.70) | 2.45(0.94,4.34) | 3.54(1.33,6.66) | 1.49(1.23,1.75) |
| Democratic People's Republic of Korea | female | 168.69(62.37,288.63) | 390.88(158.59,670.61) | 2.99(2.74,3.24) | 2.04(0.78,3.57) | 1.99(0.81,3.44) | 0.09(-0.10,0.28) |
| Democratic Republic of the Congo | female | 247.79(93.11,424.04) | 708.22(253.53,1263.24) | 3.27(3.18,3.37) | 4.41(1.70,7.46) | 4.85(1.82,8.68) | 0.27(0.22,0.33) |
| Denmark | female | 61.80(27.86,99.58) | 121.61(52.04,201.68) | 2.16(1.66,2.67) | 1.12(0.51,1.80) | 1.40(0.60,2.33) | 0.76(0.24,1.28) |
| Djibouti | female | 0.92(0.31,1.76) | 5.58(1.94,10.48) | 6.04(5.95,6.13) | 2.10(0.72,3.90) | 2.94(1.04,5.59) | 1.11(1.02,1.20) |
| Dominica | female | 3.23(1.34,5.31) | 4.42(1.92,7.40) | 0.78(0.66,0.89) | 8.69(3.59,14.14) | 9.53(4.17,15.95) | 0.03(-0.07,0.13) |
| Dominican Republic | female | 52.65(21.70,85.88) | 198.40(83.74,328.57) | 4.71(4.36,5.07) | 3.48(1.45,5.68) | 3.87(1.63,6.40) | 0.90(0.57,1.22) |
| Ecuador | female | 50.33(18.88,85.11) | 204.45(74.13,377.92) | 4.65(4.00,5.30) | 2.10(0.79,3.62) | 2.46(0.88,4.55) | 0.45(-0.16,1.06) |
| Egypt | female | 670.04(269.48,1019.74) | 2308.80(1012.20,3840.07) | 4.61(4.37,4.86) | 7.83(3.21,12.25) | 14.01(5.97,23.09) | 2.92(2.53,3.31) |
| El Salvador | female | 35.45(13.44,56.89) | 181.35(70.46,320.65) | 5.48(5.16,5.81) | 2.32(0.88,3.72) | 4.80(1.88,8.47) | 2.40(2.13,2.66) |
| Equatorial Guinea | female | 2.77(1.06,4.87) | 10.07(3.29,19.12) | 4.69(4.43,4.96) | 3.38(1.29,6.20) | 4.72(1.52,8.93) | 1.35(1.19,1.52) |
| Eritrea | female | 5.82(2.09,11.08) | 19.17(7.37,36.23) | 4.45(4.27,4.63) | 1.38(0.46,2.65) | 1.74(0.60,3.35) | 0.88(0.82,0.94) |
| Estonia | female | 5.00(2.08,7.97) | 27.76(11.25,46.14) | 5.17(4.34,6.01) | 0.35(0.15,0.57) | 1.19(0.48,1.93) | 3.27(2.51,4.03) |
| Eswatini | female | 9.98(4.14,17.26) | 31.97(11.93,57.57) | 4.15(3.39,4.92) | 7.46(3.00,13.26) | 11.50(4.42,20.68) | 1.84(1.11,2.57) |
| Ethiopia | female | 313.85(118.29,539.06) | 465.04(180.12,792.89) | 0.70(0.36,1.04) | 4.33(1.60,7.51) | 2.73(1.05,4.73) | -2.08(-2.32,-1.84) |
| Fiji | female | 32.40(13.25,56.72) | 109.88(47.13,184.60) | 4.03(3.72,4.35) | 19.37(7.85,33.13) | 29.57(12.72,50.50) | 1.24(0.91,1.58) |
| Finland | female | 48.40(21.28,77.54) | 56.15(23.06,94.47) | 0.41(-0.07,0.90) | 0.96(0.42,1.52) | 0.53(0.22,0.89) | -1.94(-2.37,-1.50) |
| France | female | 912.23(398.42,1481.76) | 1416.67(590.56,2314.08) | 1.57(1.18,1.96) | 1.49(0.66,2.39) | 1.04(0.44,1.67) | -1.06(-1.58,-0.54) |
| Gabon | female | 8.07(2.77,15.14) | 16.90(6.05,29.79) | 2.25(2.01,2.50) | 3.05(1.04,5.95) | 3.96(1.39,7.21) | 0.69(0.39,0.99) |
| Gambia | female | 2.76(1.01,4.79) | 15.01(5.73,26.77) | 5.65(5.33,5.98) | 2.15(0.79,3.71) | 3.57(1.35,6.45) | 1.55(1.38,1.72) |
| Georgia | female | 28.90(11.97,47.74) | 60.31(23.62,98.51) | 3.69(2.88,4.51) | 0.74(0.31,1.22) | 1.47(0.60,2.42) | 3.49(2.69,4.30) |
| Germany | female | 2102.33(905.72,3323.64) | 2104.30(855.10,3526.00) | -0.07(-0.26,0.12) | 2.20(0.93,3.44) | 1.29(0.55,2.14) | -1.86(-2.04,-1.67) |
| Ghana | female | 73.28(28.13,125.22) | 326.06(135.08,564.39) | 5.23(5.08,5.38) | 3.09(1.19,5.36) | 4.74(1.98,8.10) | 1.60(1.50,1.71) |
| Greece | female | 167.60(73.63,266.40) | 295.55(126.69,506.73) | 1.61(0.86,2.36) | 2.04(0.89,3.23) | 1.54(0.66,2.64) | -1.20(-1.82,-0.58) |
| Greenland | female | 0.23(0.09,0.37) | 0.23(0.10,0.38) | -0.16(-0.41,0.09) | 1.72(0.73,2.86) | 0.90(0.37,1.46) | -2.36(-2.53,-2.18) |
| Grenada | female | 6.08(2.42,10.24) | 6.24(2.67,9.99) | 0.88(0.56,1.21) | 12.10(4.96,20.43) | 10.66(4.57,17.07) | 0.12(-0.15,0.40) |
| Guam | female | 1.70(0.72,2.74) | 2.34(1.00,3.84) | 1.19(0.96,1.42) | 6.53(2.70,10.73) | 2.00(0.87,3.33) | -3.36(-3.75,-2.97) |
| Guatemala | female | 22.20(8.79,38.26) | 231.80(87.59,392.22) | 7.46(7.02,7.90) | 1.88(0.76,3.32) | 4.32(1.65,7.34) | 2.07(1.63,2.51) |
| Guinea | female | 29.79(11.02,55.19) | 68.05(24.38,119.33) | 2.74(2.34,3.15) | 2.20(0.79,3.99) | 3.07(1.14,5.57) | 1.15(1.01,1.29) |
| Guinea-Bissau | female | 5.41(1.91,9.32) | 14.54(5.55,25.43) | 3.27(3.13,3.42) | 3.59(1.32,6.13) | 5.32(2.00,9.23) | 1.34(1.17,1.51) |
| Guyana | female | 18.77(7.52,29.33) | 36.10(14.95,59.42) | 2.03(1.65,2.41) | 10.79(4.40,17.19) | 11.81(5.01,19.36) | 0.07(-0.41,0.56) |
| Haiti | female | 189.21(76.48,310.65) | 409.25(156.28,723.12) | 2.62(2.53,2.71) | 13.25(5.19,21.74) | 13.50(5.36,23.70) | 0.18(0.10,0.26) |
| Honduras | female | 13.51(5.11,23.20) | 96.04(39.20,167.15) | 6.55(6.21,6.90) | 1.44(0.54,2.44) | 3.21(1.33,5.57) | 2.63(2.21,3.04) |
| Hungary | female | 132.86(54.64,214.52) | 213.40(90.24,343.44) | 2.05(1.66,2.43) | 1.45(0.60,2.32) | 1.48(0.64,2.38) | 0.40(0.04,0.76) |
| Iceland | female | 1.32(0.51,2.20) | 2.41(0.99,3.98) | 2.05(1.91,2.19) | 0.72(0.28,1.19) | 0.60(0.25,0.98) | -0.55(-0.69,-0.41) |
| India | female | 4718.23(1928.22,7459.19) | 20954.89(9389.29,32428.43) | 5.15(4.92,5.38) | 2.85(1.17,4.52) | 3.99(1.80,6.21) | 1.20(0.91,1.48) |
| Indonesia | female | 1927.40(801.34,3034.05) | 6239.71(2721.38,9852.63) | 3.64(3.46,3.82) | 4.44(1.82,7.01) | 6.15(2.73,9.61) | 0.99(0.86,1.12) |
| Iran (Islamic Republic of) | female | 248.96(109.34,393.04) | 1224.49(535.32,1922.69) | 5.96(5.73,6.19) | 2.74(1.22,4.31) | 3.70(1.63,5.84) | 1.32(1.13,1.50) |
| Iraq | female | 259.03(106.61,415.88) | 786.36(332.55,1269.38) | 3.56(3.32,3.80) | 6.70(2.74,10.78) | 7.83(3.26,12.57) | 0.22(0.04,0.41) |
| Ireland | female | 42.58(18.26,63.77) | 45.12(19.19,74.75) | 0.63(0.41,0.85) | 1.79(0.78,2.68) | 0.88(0.38,1.45) | -1.83(-2.05,-1.61) |
| Israel | female | 82.85(35.62,131.98) | 224.71(86.71,383.98) | 2.80(1.91,3.70) | 3.39(1.44,5.30) | 2.66(1.04,4.52) | -1.17(-2.00,-0.33) |
| Italy | female | 1926.98(823.05,3035.46) | 2185.11(918.78,3548.71) | 0.72(0.60,0.85) | 3.35(1.42,5.26) | 1.67(0.70,2.71) | -2.04(-2.15,-1.94) |
| Jamaica | female | 134.04(56.86,205.91) | 238.19(106.14,388.26) | 1.49(1.21,1.78) | 13.24(5.68,20.19) | 13.41(5.94,21.78) | -0.38(-0.66,-0.10) |
| Japan | female | 1232.93(543.76,1975.94) | 2158.34(837.16,3902.44) | 2.08(1.70,2.46) | 1.28(0.56,2.04) | 0.55(0.22,0.98) | -2.59(-2.99,-2.19) |
| Jordan | female | 50.38(20.76,81.28) | 176.32(76.52,292.78) | 3.43(2.96,3.90) | 10.23(4.18,16.35) | 7.37(3.22,11.97) | -1.57(-2.30,-0.83) |
| Kazakhstan | female | 49.66(20.21,82.16) | 89.25(35.66,155.53) | 0.72(0.20,1.25) | 0.64(0.26,1.06) | 0.91(0.36,1.57) | 0.07(-0.43,0.58) |
| Kenya | female | 75.87(31.79,125.42) | 291.33(109.27,502.35) | 4.94(4.75,5.13) | 2.21(0.92,3.72) | 3.00(1.14,5.25) | 1.48(1.26,1.70) |
| Kiribati | female | 3.82(1.65,6.13) | 10.59(4.34,17.72) | 3.47(3.38,3.56) | 20.11(8.53,33.79) | 28.64(11.95,48.59) | 1.16(1.01,1.31) |
| Kuwait | female | 11.21(4.97,17.30) | 40.95(18.41,64.70) | 4.27(3.86,4.68) | 5.76(2.56,8.81) | 4.46(1.98,7.06) | -0.79(-1.26,-0.32) |
| Kyrgyzstan | female | 7.21(2.96,11.71) | 17.81(7.60,29.67) | 1.74(1.26,2.23) | 0.41(0.17,0.66) | 0.72(0.31,1.20) | 0.89(0.43,1.35) |
| Lao People's Democratic Republic | female | 23.66(8.54,40.42) | 50.93(19.66,93.52) | 2.17(2.01,2.32) | 2.57(0.93,4.48) | 2.58(0.97,4.75) | -0.23(-0.32,-0.13) |
| Latvia | female | 11.77(4.83,19.13) | 39.12(16.06,66.17) | 4.05(3.50,4.60) | 0.49(0.20,0.80) | 1.26(0.52,2.14) | 3.03(2.47,3.59) |
| Lebanon | female | 43.37(18.15,71.81) | 122.80(49.61,199.58) | 3.63(3.46,3.80) | 4.52(1.89,7.47) | 3.43(1.39,5.59) | -0.91(-0.97,-0.85) |
| Lesotho | female | 12.59(5.08,22.56) | 32.13(12.25,59.60) | 3.77(3.00,4.55) | 2.67(1.09,4.72) | 6.12(2.43,11.33) | 3.78(3.08,4.48) |
| Liberia | female | 20.51(8.24,33.61) | 55.36(21.71,96.38) | 3.40(3.25,3.55) | 4.63(1.89,7.67) | 6.60(2.60,11.55) | 1.36(1.18,1.55) |
| Libya | female | 21.58(9.18,35.25) | 107.77(42.82,180.04) | 6.25(5.93,6.57) | 2.64(1.12,4.31) | 4.77(1.98,7.98) | 2.91(2.54,3.29) |
| Lithuania | female | 10.02(4.05,15.91) | 44.29(18.80,72.65) | 4.15(3.29,5.02) | 0.35(0.14,0.56) | 0.98(0.40,1.60) | 2.55(1.65,3.46) |
| Luxembourg | female | 6.44(2.56,10.43) | 7.43(3.09,12.52) | 0.55(0.37,0.73) | 1.85(0.74,3.02) | 0.94(0.39,1.57) | -2.07(-2.28,-1.86) |
| Madagascar | female | 40.38(14.95,72.21) | 88.48(34.76,161.46) | 2.50(2.28,2.73) | 2.08(0.77,3.66) | 2.27(0.86,4.19) | 0.20(0.13,0.28) |
| Malawi | female | 23.74(8.36,44.37) | 52.40(18.99,97.00) | 2.26(2.10,2.42) | 1.53(0.52,2.89) | 1.66(0.61,3.16) | 0.04(-0.12,0.21) |
| Malaysia | female | 170.56(74.55,280.63) | 418.23(172.72,676.56) | 2.71(2.48,2.96) | 3.94(1.73,6.52) | 3.38(1.38,5.53) | -0.62(-0.81,-0.43) |
| Maldives | female | 3.13(1.32,4.83) | 5.56(2.48,8.86) | 1.40(1.08,1.73) | 9.67(4.15,14.86) | 4.25(1.91,6.78) | -3.11(-3.39,-2.84) |
| Mali | female | 52.12(21.16,86.84) | 151.02(58.34,263.00) | 3.77(3.67,3.86) | 3.50(1.43,6.12) | 4.73(1.84,8.43) | 1.01(0.86,1.16) |
| Malta | female | 10.53(4.65,16.27) | 16.85(7.41,26.80) | 1.33(0.97,1.70) | 4.48(1.99,6.87) | 2.46(1.09,3.93) | -2.13(-2.48,-1.77) |
| Marshall Islands | female | 1.80(0.80,2.86) | 7.16(2.72,12.44) | 4.47(4.23,4.70) | 23.54(10.48,37.33) | 45.64(17.05,77.29) | 1.99(1.57,2.40) |
| Mauritania | female | 21.83(8.55,37.27) | 55.44(22.23,91.72) | 2.87(2.78,2.97) | 4.79(1.93,8.08) | 6.18(2.47,10.30) | 0.60(0.49,0.72) |
| Mauritius | female | 17.16(6.77,27.94) | 102.32(41.82,167.56) | 7.19(6.23,8.16) | 4.59(1.82,7.37) | 10.18(4.19,16.55) | 3.75(2.81,4.71) |
| Mexico | female | 1482.31(564.72,2305.55) | 4254.90(1674.51,6890.72) | 3.49(3.20,3.78) | 8.38(3.17,13.15) | 6.69(2.61,10.80) | -0.72(-1.00,-0.44) |
| Micronesia (Federated States of) | female | 4.02(1.74,6.34) | 8.83(3.71,14.13) | 2.56(2.31,2.82) | 17.13(7.40,27.04) | 25.07(10.45,40.33) | 1.24(0.89,1.58) |
| Monaco | female | 0.36(0.14,0.63) | 0.59(0.22,1.05) | 1.67(1.50,1.85) | 0.65(0.25,1.14) | 0.77(0.29,1.38) | 0.70(0.61,0.80) |
| Mongolia | female | 1.95(0.70,3.51) | 5.27(1.95,9.57) | 3.06(2.71,3.41) | 0.36(0.13,0.64) | 0.48(0.18,0.88) | 0.82(0.67,0.97) |
| Montenegro | female | 4.86(2.02,8.01) | 12.48(4.83,20.57) | 3.29(3.05,3.53) | 1.42(0.58,2.35) | 2.32(0.91,3.83) | 1.73(1.49,1.97) |
| Morocco | female | 164.03(66.97,270.23) | 775.86(308.37,1263.40) | 5.64(5.44,5.84) | 2.57(1.05,4.22) | 4.88(1.96,7.88) | 2.54(2.35,2.73) |
| Mozambique | female | 43.34(18.18,74.64) | 103.98(39.23,190.58) | 3.30(3.11,3.48) | 1.89(0.78,3.30) | 2.35(0.82,4.28) | 1.13(0.92,1.35) |
| Myanmar | female | 228.65(82.53,417.90) | 570.64(206.45,1053.85) | 2.69(2.50,2.89) | 2.18(0.80,3.90) | 2.35(0.84,4.36) | -0.01(-0.14,0.12) |
| Namibia | female | 18.34(7.01,30.80) | 49.23(17.61,83.76) | 2.80(2.43,3.17) | 6.22(2.37,10.51) | 7.44(2.75,12.64) | 0.28(-0.05,0.61) |
| Nauru | female | 0.24(0.09,0.41) | 0.54(0.21,0.93) | 2.31(2.04,2.59) | 13.93(5.43,22.85) | 18.69(7.20,31.60) | 0.89(0.72,1.05) |
| Nepal | female | 61.92(24.42,104.11) | 274.78(104.33,486.50) | 5.15(4.84,5.47) | 1.82(0.72,3.11) | 2.80(1.06,5.05) | 1.44(1.18,1.70) |
| Netherlands | female | 281.04(106.87,469.54) | 263.33(105.55,448.39) | -0.20(-0.51,0.10) | 2.08(0.79,3.47) | 1.02(0.41,1.72) | -2.31(-2.63,-1.99) |
| New Zealand | female | 31.27(13.68,48.20) | 62.70(25.84,98.62) | 1.70(1.21,2.20) | 1.33(0.59,2.05) | 1.19(0.49,1.87) | -0.81(-1.25,-0.38) |
| Nicaragua | female | 17.95(7.29,30.25) | 80.53(30.20,142.94) | 5.00(4.66,5.34) | 2.38(0.95,4.01) | 3.21(1.20,5.66) | 1.09(0.81,1.36) |
| Niger | female | 19.45(7.10,32.73) | 83.45(31.32,145.48) | 4.79(4.70,4.88) | 2.01(0.73,3.62) | 2.76(1.06,4.86) | 0.94(0.81,1.07) |
| Nigeria | female | 617.89(267.84,1011.05) | 1320.78(483.60,2219.59) | 2.29(2.09,2.49) | 3.33(1.43,5.42) | 3.71(1.38,6.28) | 0.28(0.24,0.32) |
| Niue | female | 0.14(0.05,0.23) | 0.19(0.08,0.33) | 1.04(0.99,1.09) | 9.87(3.85,16.26) | 16.16(6.56,27.02) | 1.51(1.35,1.66) |
| North Macedonia | female | 21.82(9.06,36.46) | 64.03(27.07,105.33) | 3.82(3.38,4.26) | 2.52(1.05,4.20) | 4.23(1.79,6.96) | 1.72(1.22,2.23) |
| Northern Mariana Islands | female | 0.58(0.24,0.95) | 1.90(0.82,3.02) | 4.09(3.80,4.39) | 10.45(4.35,17.48) | 8.84(3.82,13.96) | -0.66(-0.87,-0.45) |
| Norway | female | 42.21(16.55,68.35) | 61.15(24.52,105.40) | 1.20(0.64,1.77) | 0.85(0.33,1.36) | 0.79(0.32,1.33) | -0.23(-0.74,0.28) |
| Oman | female | 17.54(7.36,29.62) | 46.11(18.54,73.42) | 3.37(2.98,3.76) | 6.45(2.73,10.87) | 6.78(2.75,11.03) | 0.48(0.19,0.77) |
| Pakistan | female | 694.18(292.85,1171.07) | 2590.81(1014.45,4139.78) | 3.97(3.74,4.20) | 3.45(1.43,5.85) | 5.92(2.18,9.62) | 1.49(1.19,1.79) |
| Palau | female | 0.46(0.19,0.77) | 1.37(0.55,2.31) | 3.51(3.36,3.66) | 10.35(4.25,17.51) | 16.48(6.40,27.71) | 1.77(1.62,1.92) |
| Palestine | female | 36.88(15.58,60.12) | 98.45(40.14,152.68) | 3.45(3.26,3.64) | 9.01(3.82,14.80) | 9.47(3.77,14.71) | 0.30(0.08,0.52) |
| Panama | female | 16.59(7.02,26.82) | 72.08(30.01,123.73) | 4.81(4.48,5.13) | 2.37(1.00,3.87) | 2.96(1.26,5.12) | 0.67(0.36,0.98) |
| Papua New Guinea | female | 53.20(21.54,97.58) | 165.26(61.91,285.31) | 3.65(3.54,3.76) | 6.66(2.77,11.54) | 7.64(2.88,13.10) | 0.39(0.32,0.47) |
| Paraguay | female | 30.70(10.84,54.44) | 147.92(54.62,263.08) | 5.50(5.12,5.89) | 2.81(0.98,4.95) | 5.00(1.84,8.91) | 2.19(1.86,2.53) |
| Peru | female | 118.88(48.25,199.54) | 450.01(191.27,773.06) | 4.17(3.85,4.49) | 2.15(0.87,3.62) | 2.55(1.08,4.36) | 0.11(-0.21,0.43) |
| Philippines | female | 271.81(102.41,467.26) | 1212.47(473.12,2078.84) | 5.06(5.00,5.12) | 2.60(0.94,4.56) | 3.20(1.27,5.52) | 0.96(0.87,1.05) |
| Poland | female | 479.61(203.46,759.70) | 821.13(343.49,1326.72) | 1.98(1.64,2.31) | 1.77(0.75,2.78) | 1.60(0.68,2.55) | -0.32(-0.55,-0.08) |
| Portugal | female | 279.72(119.30,431.71) | 466.83(197.06,763.05) | 1.39(1.03,1.75) | 3.42(1.45,5.22) | 2.22(0.92,3.55) | -1.84(-2.26,-1.43) |
| Puerto Rico | female | 157.74(66.82,242.92) | 252.09(108.59,415.13) | 1.21(0.91,1.52) | 8.44(3.58,13.10) | 5.00(2.17,8.20) | -1.91(-2.13,-1.69) |
| Qatar | female | 2.87(1.17,4.70) | 24.38(10.16,40.36) | 7.64(7.04,8.25) | 12.66(5.06,20.33) | 14.74(6.21,24.00) | 0.57(-0.46,1.61) |
| Republic of Korea | female | 353.75(153.78,568.11) | 908.72(370.66,1536.66) | 2.61(2.06,3.17) | 2.51(1.08,4.10) | 1.55(0.64,2.61) | -2.08(-2.71,-1.45) |
| Republic of Moldova | female | 13.88(5.66,23.47) | 29.10(12.22,48.62) | 1.42(0.46,2.40) | 0.55(0.23,0.92) | 0.76(0.32,1.26) | 0.16(-0.76,1.10) |
| Romania | female | 143.63(58.48,222.14) | 234.90(99.40,378.13) | 1.82(1.37,2.28) | 0.98(0.41,1.52) | 0.94(0.40,1.50) | 0.00(-0.44,0.45) |
| Russian Federation | female | 482.39(201.81,745.45) | 3199.49(1262.71,5102.42) | 5.43(3.67,7.22) | 0.39(0.16,0.60) | 1.88(0.74,3.01) | 4.35(2.69,6.05) |
| Rwanda | female | 32.71(11.32,62.83) | 58.53(19.46,118.03) | 1.37(0.80,1.94) | 2.79(0.90,5.33) | 2.21(0.74,4.49) | -1.60(-1.95,-1.24) |
| Saint Kitts and Nevis | female | 2.49(1.08,3.93) | 2.36(0.99,3.80) | 0.38(0.04,0.73) | 11.11(4.79,17.60) | 7.56(3.15,12.09) | -0.62(-0.97,-0.26) |
| Saint Lucia | female | 6.28(2.55,10.08) | 11.07(4.61,17.56) | 0.73(0.30,1.17) | 13.71(5.59,22.12) | 8.41(3.53,13.31) | -2.94(-3.39,-2.49) |
| Saint Vincent and the Grenadines | female | 5.92(2.49,9.64) | 6.89(3.13,11.14) | 0.24(-0.13,0.62) | 14.98(6.37,24.30) | 10.09(4.57,16.31) | -1.64(-2.01,-1.26) |
| Samoa | female | 4.92(2.27,7.89) | 13.57(5.75,22.07) | 3.37(3.25,3.48) | 12.11(5.53,19.37) | 19.46(8.22,31.62) | 1.56(1.46,1.65) |
| San Marino | female | 0.28(0.11,0.47) | 0.39(0.14,0.73) | 1.34(0.94,1.74) | 1.20(0.48,2.02) | 0.64(0.24,1.18) | -1.58(-1.88,-1.27) |
| Sao Tome and Principe | female | 0.49(0.18,0.88) | 0.91(0.35,1.63) | 1.92(1.87,1.98) | 1.47(0.55,2.65) | 1.96(0.72,3.59) | 0.84(0.64,1.04) |
| Saudi Arabia | female | 117.73(48.97,190.98) | 471.87(185.97,772.55) | 4.14(3.91,4.37) | 5.57(2.33,9.05) | 7.83(3.16,12.53) | 0.84(0.65,1.02) |
| Senegal | female | 46.50(18.70,79.53) | 173.11(70.62,304.21) | 4.38(4.20,4.57) | 3.59(1.44,6.07) | 5.23(2.08,9.29) | 1.13(0.98,1.27) |
| Serbia | female | 127.69(51.17,202.44) | 269.86(106.80,447.18) | 2.47(2.21,2.73) | 2.61(1.04,4.14) | 2.56(1.01,4.24) | -0.33(-0.53,-0.13) |
| Seychelles | female | 0.80(0.32,1.30) | 2.26(0.92,3.71) | 3.77(3.57,3.97) | 2.34(0.93,3.81) | 3.96(1.61,6.55) | 2.12(1.91,2.32) |
| Sierra Leone | female | 18.61(7.20,30.63) | 52.40(19.29,91.10) | 3.49(3.31,3.67) | 2.27(0.83,3.74) | 3.50(1.27,6.18) | 1.60(1.31,1.90) |
| Singapore | female | 25.51(10.49,41.91) | 28.39(12.27,49.69) | 0.58(0.09,1.07) | 2.41(0.98,4.01) | 0.60(0.26,1.05) | -4.10(-4.67,-3.52) |
| Slovakia | female | 44.42(18.75,74.69) | 60.27(24.52,102.52) | 1.10(1.01,1.18) | 1.22(0.52,2.05) | 0.98(0.40,1.66) | -0.68(-0.75,-0.60) |
| Slovenia | female | 20.41(8.31,33.18) | 32.33(13.06,54.16) | -0.06(-0.72,0.59) | 1.28(0.51,2.08) | 0.93(0.38,1.53) | -2.74(-3.41,-2.07) |
| Solomon Islands | female | 5.65(2.14,9.63) | 21.40(8.18,36.18) | 4.30(4.15,4.45) | 10.58(4.20,17.64) | 13.55(5.13,22.94) | 0.78(0.63,0.92) |
| Somalia | female | 23.59(8.86,43.58) | 75.15(26.60,138.29) | 4.03(3.91,4.14) | 2.73(1.03,4.97) | 3.17(1.15,6.05) | 0.59(0.47,0.71) |
| South Africa | female | 760.09(336.33,1186.83) | 3001.64(1293.56,4635.53) | 4.72(4.21,5.23) | 6.94(3.06,10.83) | 12.37(5.35,19.04) | 2.13(1.66,2.61) |
| South Sudan | female | 20.87(7.65,36.92) | 42.24(14.43,77.02) | 2.20(1.88,2.52) | 2.40(0.90,4.34) | 3.26(1.11,5.96) | 0.96(0.81,1.11) |
| Spain | female | 1003.84(409.82,1600.34) | 1065.84(416.35,1874.55) | 0.15(-0.01,0.32) | 2.94(1.21,4.66) | 1.14(0.45,1.96) | -3.14(-3.29,-3.00) |
| Sri Lanka | female | 94.53(33.88,163.60) | 449.66(173.16,822.01) | 5.76(5.49,6.02) | 2.32(0.83,4.44) | 3.10(1.17,5.71) | 1.62(1.34,1.90) |
| Sudan | female | 144.38(64.16,240.46) | 417.20(193.35,696.14) | 3.49(3.29,3.68) | 3.54(1.56,5.87) | 5.13(2.38,8.37) | 1.39(1.25,1.54) |
| Suriname | female | 7.45(3.10,11.84) | 20.33(8.65,33.13) | 3.85(3.56,4.15) | 6.03(2.53,9.67) | 6.01(2.57,9.78) | 0.36(0.13,0.58) |
| Sweden | female | 108.28(44.78,176.54) | 166.65(66.22,286.79) | 1.51(1.22,1.80) | 1.00(0.41,1.63) | 0.97(0.39,1.64) | 0.05(-0.21,0.30) |
| Switzerland | female | 149.23(60.91,235.03) | 127.91(53.32,219.27) | -0.75(-0.92,-0.57) | 1.96(0.79,3.05) | 0.81(0.34,1.38) | -3.01(-3.18,-2.84) |
| Syrian Arab Republic | female | 89.27(35.37,149.27) | 219.44(93.08,373.39) | 2.58(2.22,2.94) | 4.43(1.78,7.43) | 4.93(2.15,8.28) | -0.18(-0.45,0.08) |
| Taiwan (Province of China) | female | 398.09(170.61,640.26) | 831.79(343.03,1369.33) | 1.56(1.12,2.00) | 6.22(2.64,9.96) | 3.33(1.38,5.45) | -2.88(-3.34,-2.41) |
| Tajikistan | female | 11.29(4.47,18.34) | 26.84(10.05,49.17) | 2.38(2.18,2.59) | 0.76(0.30,1.24) | 1.10(0.41,2.02) | 0.96(0.56,1.37) |
| Thailand | female | 493.12(173.10,855.24) | 1872.28(738.43,3155.58) | 3.74(3.49,3.99) | 3.08(1.09,5.24) | 3.03(1.19,5.12) | -0.73(-0.99,-0.46) |
| Timor-Leste | female | 1.65(0.61,3.00) | 6.73(2.48,12.48) | 5.12(4.85,5.38) | 1.62(0.58,2.93) | 1.77(0.63,3.22) | 0.35(0.12,0.57) |
| Togo | female | 7.70(3.01,13.39) | 34.99(13.93,61.87) | 5.05(5.01,5.09) | 1.70(0.64,3.01) | 2.42(0.89,4.36) | 1.10(1.00,1.20) |
| Tokelau | female | 0.07(0.03,0.12) | 0.09(0.04,0.16) | 0.85(0.74,0.97) | 10.22(4.25,17.91) | 11.81(4.65,20.13) | 0.40(0.26,0.53) |
| Tonga | female | 3.79(1.54,6.05) | 7.18(3.03,11.67) | 2.09(1.97,2.22) | 13.94(5.69,22.56) | 16.83(7.14,27.44) | 0.70(0.63,0.76) |
| Trinidad and Tobago | female | 81.00(35.98,124.91) | 146.33(63.40,238.77) | 1.64(1.48,1.81) | 19.67(8.62,30.42) | 14.09(6.10,22.96) | -1.32(-1.47,-1.17) |
| Tunisia | female | 28.70(11.90,49.35) | 146.53(59.96,243.27) | 5.55(5.44,5.66) | 1.55(0.64,2.62) | 2.30(0.94,3.78) | 1.50(1.37,1.63) |
| Türkiye | female | 1046.52(453.10,1668.55) | 2431.23(1046.99,3942.48) | 3.69(3.17,4.21) | 6.71(2.96,10.66) | 5.16(2.24,8.34) | -0.03(-0.59,0.53) |
| Turkmenistan | female | 8.03(3.16,13.22) | 35.81(13.55,60.29) | 4.31(3.89,4.72) | 0.77(0.30,1.27) | 1.70(0.66,2.80) | 1.98(1.61,2.35) |
| Tuvalu | female | 0.40(0.15,0.64) | 0.73(0.28,1.19) | 1.96(1.88,2.03) | 11.26(4.28,18.21) | 13.88(5.29,23.04) | 0.68(0.58,0.78) |
| Uganda | female | 50.92(17.93,96.91) | 157.54(53.99,305.55) | 3.26(3.03,3.50) | 2.01(0.69,3.94) | 2.51(0.85,4.86) | 0.36(0.08,0.63) |
| Ukraine | female | 96.17(39.11,161.80) | 110.68(40.23,197.51) | -0.88(-1.54,-0.22) | 0.20(0.08,0.33) | 0.21(0.08,0.37) | -1.05(-1.63,-0.47) |
| United Arab Emirates | female | 8.07(3.35,13.28) | 45.50(17.63,75.64) | 7.08(6.53,7.62) | 6.78(2.77,11.45) | 19.94(8.54,32.56) | 5.94(5.10,6.78) |
| United Kingdom | female | 979.52(431.43,1493.48) | 764.53(321.24,1173.75) | -0.42(-0.77,-0.07) | 1.58(0.70,2.40) | 0.81(0.35,1.24) | -1.92(-2.25,-1.59) |
| United Republic of Tanzania | female | 42.71(15.75,77.93) | 122.24(44.78,228.35) | 3.28(3.19,3.38) | 1.01(0.36,1.90) | 1.16(0.41,2.19) | 0.28(0.20,0.36) |
| United States of America | female | 3660.34(1530.48,5747.34) | 6207.09(2509.30,10033.92) | 1.26(0.90,1.62) | 1.74(0.73,2.72) | 1.70(0.68,2.73) | -0.51(-0.87,-0.15) |
| United States Virgin Islands | female | 2.40(1.06,3.85) | 3.56(1.43,5.87) | 1.24(0.93,1.55) | 6.40(2.84,10.35) | 3.36(1.36,5.47) | -2.06(-2.33,-1.80) |
| Uruguay | female | 42.87(16.00,72.57) | 68.44(27.13,113.04) | 1.58(1.49,1.68) | 1.81(0.68,3.03) | 1.69(0.68,2.74) | -0.14(-0.24,-0.04) |
| Uzbekistan | female | 34.03(12.91,58.26) | 191.91(79.19,342.60) | 5.22(4.86,5.58) | 0.52(0.20,0.89) | 1.44(0.60,2.54) | 3.02(2.55,3.49) |
| Vanuatu | female | 0.86(0.35,1.58) | 3.35(1.28,5.93) | 4.19(4.06,4.32) | 4.13(1.62,7.68) | 5.04(1.94,9.00) | 0.47(0.39,0.55) |
| Venezuela (Bolivarian Republic of) | female | 150.49(61.67,242.70) | 650.46(272.17,1101.56) | 4.82(4.49,5.15) | 3.30(1.32,5.30) | 4.10(1.70,6.96) | 0.60(0.25,0.94) |
| Viet Nam | female | 519.15(199.82,914.56) | 1470.06(546.81,2744.14) | 3.60(3.47,3.72) | 2.34(0.90,4.13) | 2.86(1.05,5.30) | 0.79(0.69,0.90) |
| Yemen | female | 48.79(19.54,81.55) | 167.65(68.29,290.76) | 4.17(3.98,4.37) | 2.26(0.91,3.82) | 2.82(1.17,4.78) | 0.76(0.64,0.88) |
| Zambia | female | 31.84(12.11,55.57) | 80.25(29.56,145.82) | 2.74(2.43,3.05) | 3.06(1.13,5.44) | 2.92(1.11,5.34) | -0.54(-0.75,-0.33) |
| Zimbabwe | female | 60.52(23.62,104.66) | 201.52(76.48,363.56) | 4.51(3.74,5.27) | 3.64(1.51,6.42) | 6.70(2.53,11.95) | 2.96(2.22,3.71) |
| Afghanistan | male | 72.64(25.68,141.30) | 77.80(27.80,139.27) | 0.22(0.08,0.36) | 2.32(0.82,4.46) | 2.24(0.80,4.00) | -0.02(-0.10,0.07) |
| Albania | male | 3.54(1.26,6.39) | 9.03(3.49,16.88) | 3.49(3.24,3.74) | 0.60(0.21,1.07) | 0.48(0.18,0.90) | -0.73(-0.94,-0.52) |
| Algeria | male | 77.23(28.66,143.07) | 333.74(136.70,587.29) | 5.01(4.70,5.32) | 1.88(0.69,3.44) | 2.38(0.97,4.18) | 1.00(0.76,1.24) |
| American Samoa | male | 1.55(0.64,2.44) | 3.89(1.64,6.05) | 2.80(2.36,3.24) | 15.57(6.37,25.23) | 17.83(7.45,27.90) | 0.38(0.03,0.73) |
| Andorra | male | 0.47(0.16,0.86) | 0.99(0.39,1.80) | 2.29(1.95,2.64) | 2.06(0.71,3.69) | 1.28(0.50,2.33) | -1.19(-1.53,-0.85) |
| Angola | male | 52.62(19.37,95.59) | 146.71(55.07,268.31) | 3.35(3.07,3.62) | 4.21(1.55,7.76) | 4.57(1.77,8.38) | 0.15(0.01,0.28) |
| Antigua and Barbuda | male | 1.29(0.46,2.29) | 2.16(0.81,3.71) | 1.68(1.27,2.09) | 5.71(2.04,9.99) | 5.25(1.91,8.81) | -0.01(-0.37,0.36) |
| Argentina | male | 247.52(100.14,432.06) | 344.28(116.66,621.48) | 1.23(0.91,1.56) | 2.05(0.81,3.56) | 1.51(0.51,2.73) | -0.81(-1.11,-0.52) |
| Armenia | male | 6.40(2.33,11.42) | 15.02(5.81,26.39) | 2.87(1.62,4.14) | 0.70(0.25,1.23) | 0.88(0.34,1.53) | 0.47(-0.56,1.50) |
| Australia | male | 131.39(57.02,216.80) | 351.66(136.61,558.20) | 2.88(2.60,3.16) | 1.83(0.78,3.03) | 1.57(0.61,2.50) | -0.78(-1.03,-0.52) |
| Austria | male | 56.71(22.29,97.52) | 132.33(47.08,236.98) | 3.91(3.34,4.48) | 1.39(0.53,2.36) | 1.52(0.54,2.71) | 1.16(0.60,1.72) |
| Azerbaijan | male | 9.29(3.43,17.71) | 33.59(12.33,61.51) | 4.38(4.01,4.76) | 0.62(0.23,1.10) | 0.99(0.38,1.81) | 1.73(1.41,2.04) |
| Bahamas | male | 1.90(0.80,3.47) | 5.38(2.11,9.40) | 3.36(3.23,3.49) | 3.36(1.45,6.04) | 3.52(1.36,6.16) | -0.03(-0.20,0.14) |
| Bahrain | male | 7.12(2.74,11.74) | 38.68(15.80,64.77) | 5.29(5.09,5.48) | 14.45(5.28,24.51) | 16.82(6.61,28.92) | 0.35(-0.06,0.75) |
| Bangladesh | male | 234.78(81.93,443.37) | 582.44(195.95,1168.01) | 2.56(2.24,2.89) | 1.05(0.37,2.03) | 1.02(0.33,2.13) | -0.67(-1.26,-0.07) |
| Barbados | male | 8.86(3.42,14.53) | 14.93(5.78,25.79) | 1.20(0.87,1.52) | 7.21(2.81,11.75) | 6.59(2.54,11.34) | -0.65(-0.97,-0.32) |
| Belarus | male | 7.16(2.80,12.45) | 17.87(6.63,30.19) | 1.49(0.43,2.56) | 0.18(0.07,0.31) | 0.37(0.14,0.65) | 0.97(-0.11,2.07) |
| Belgium | male | 73.84(31.15,121.66) | 108.26(45.32,183.08) | 1.61(1.42,1.80) | 1.38(0.58,2.30) | 0.94(0.39,1.59) | -1.13(-1.38,-0.88) |
| Belize | male | 1.27(0.50,2.15) | 4.96(2.17,8.45) | 4.56(4.05,5.08) | 2.97(1.15,5.10) | 3.79(1.64,6.65) | 0.80(0.14,1.46) |
| Benin | male | 8.90(3.01,18.25) | 22.53(8.02,45.16) | 3.01(2.93,3.09) | 1.15(0.38,2.46) | 1.41(0.43,3.02) | 0.59(0.44,0.74) |
| Bermuda | male | 0.82(0.31,1.38) | 1.31(0.53,2.17) | 2.24(1.91,2.57) | 3.74(1.38,6.33) | 2.16(0.87,3.58) | -1.19(-1.46,-0.93) |
| Bhutan | male | 3.76(1.59,6.40) | 14.69(6.04,26.09) | 4.72(4.59,4.85) | 4.44(1.85,7.60) | 5.43(2.23,9.80) | 0.80(0.75,0.86) |
| Bolivia (Plurinational State of) | male | 35.56(13.44,63.91) | 121.97(45.68,223.80) | 4.54(4.38,4.70) | 2.91(1.05,5.30) | 3.33(1.24,6.04) | 0.70(0.62,0.78) |
| Bosnia and Herzegovina | male | 11.92(4.77,21.46) | 56.82(20.96,103.71) | 6.12(5.51,6.74) | 0.83(0.33,1.51) | 2.09(0.78,3.78) | 3.77(3.29,4.24) |
| Botswana | male | 12.32(4.67,20.85) | 32.82(13.27,54.81) | 2.68(2.25,3.11) | 6.52(2.41,10.64) | 7.09(2.91,11.90) | 0.08(-0.37,0.54) |
| Brazil | male | 1238.61(540.40,1928.54) | 3907.90(1652.57,6185.95) | 4.04(3.87,4.21) | 3.79(1.65,5.78) | 3.87(1.62,6.14) | 0.33(0.17,0.49) |
| Brunei Darussalam | male | 3.13(1.22,5.77) | 6.42(2.62,10.90) | 2.90(2.66,3.14) | 7.70(2.96,14.12) | 5.85(2.26,9.86) | -0.14(-0.48,0.21) |
| Bulgaria | male | 43.37(16.55,73.65) | 72.53(26.88,120.43) | 1.65(1.31,1.98) | 0.94(0.33,1.60) | 1.26(0.45,2.12) | 1.01(0.69,1.33) |
| Burkina Faso | male | 32.74(11.43,59.79) | 65.93(22.49,124.87) | 2.30(2.18,2.41) | 2.10(0.75,3.92) | 2.10(0.72,3.97) | 0.00(-0.06,0.06) |
| Burundi | male | 19.34(6.90,37.08) | 32.00(9.93,64.19) | 1.34(0.94,1.75) | 2.30(0.81,4.46) | 1.92(0.64,3.89) | -0.97(-1.12,-0.82) |
| Cabo Verde | male | 0.52(0.18,1.04) | 2.08(0.69,4.00) | 3.92(3.34,4.51) | 0.51(0.17,1.02) | 1.37(0.43,2.68) | 2.44(1.84,3.03) |
| Cambodia | male | 10.37(3.25,21.92) | 30.29(9.59,63.99) | 3.62(3.33,3.92) | 0.82(0.24,1.64) | 0.92(0.27,2.03) | 0.36(0.13,0.59) |
| Cameroon | male | 46.93(15.69,89.10) | 161.12(52.86,316.68) | 3.95(3.82,4.08) | 2.90(0.93,5.42) | 3.63(1.19,7.06) | 0.58(0.43,0.74) |
| Canada | male | 103.07(35.25,193.94) | 210.13(73.25,417.34) | 1.90(1.34,2.46) | 0.89(0.27,1.73) | 0.62(0.21,1.22) | -1.66(-2.26,-1.07) |
| Central African Republic | male | 12.95(4.73,24.58) | 21.08(7.44,38.60) | 1.53(1.39,1.67) | 3.61(1.26,7.16) | 3.29(1.14,5.83) | -0.30(-0.34,-0.25) |
| Chad | male | 17.49(6.04,32.45) | 44.58(15.48,80.02) | 2.96(2.79,3.14) | 1.53(0.55,2.82) | 1.83(0.62,3.31) | 0.46(0.20,0.72) |
| Chile | male | 37.94(13.83,70.21) | 95.79(31.48,188.41) | 3.29(2.92,3.66) | 0.98(0.35,1.87) | 0.88(0.28,1.75) | -0.09(-0.50,0.33) |
| China | male | 2901.19(1127.56,4933.19) | 10750.24(4284.02,18060.29) | 4.38(4.27,4.49) | 1.27(0.50,2.12) | 1.43(0.59,2.39) | 0.46(0.27,0.66) |
| Colombia | male | 122.70(48.92,205.50) | 363.33(141.49,624.86) | 2.88(2.61,3.16) | 1.62(0.62,2.77) | 1.49(0.58,2.55) | -1.01(-1.28,-0.74) |
| Comoros | male | 0.98(0.31,1.94) | 2.40(0.76,4.69) | 2.72(2.49,2.94) | 1.37(0.46,2.65) | 1.41(0.43,2.90) | -0.15(-0.32,0.02) |
| Congo | male | 16.10(6.45,29.44) | 35.82(12.82,66.35) | 2.35(1.88,2.83) | 4.41(1.82,7.86) | 3.79(1.27,7.37) | -0.76(-1.00,-0.51) |
| Cook Islands | male | 0.52(0.21,0.94) | 1.01(0.37,1.78) | 1.89(1.70,2.09) | 9.15(3.57,16.48) | 8.24(3.04,14.64) | -0.58(-0.77,-0.40) |
| Costa Rica | male | 6.91(2.54,11.87) | 37.24(13.42,67.13) | 5.08(4.66,5.51) | 0.89(0.33,1.54) | 1.52(0.55,2.75) | 1.20(0.77,1.64) |
| Croatia | male | 19.80(7.80,33.80) | 56.26(22.59,97.09) | 3.27(2.84,3.71) | 1.11(0.42,1.90) | 1.47(0.59,2.53) | 0.45(0.02,0.88) |
| Cuba | male | 62.65(24.67,107.16) | 104.34(39.91,178.20) | 1.90(1.35,2.44) | 1.30(0.50,2.23) | 1.12(0.43,1.92) | -0.33(-0.85,0.20) |
| Cyprus | male | 20.31(7.95,33.74) | 29.49(11.04,50.62) | 0.65(0.39,0.90) | 10.39(3.82,17.83) | 3.88(1.52,6.57) | -2.91(-3.11,-2.71) |
| Czechia | male | 56.84(22.81,95.22) | 187.17(74.91,312.19) | 6.14(4.93,7.36) | 1.11(0.45,1.87) | 2.01(0.79,3.39) | 3.88(2.88,4.89) |
| Côte d'Ivoire | male | 38.54(15.23,69.74) | 119.98(46.23,210.12) | 3.21(2.92,3.50) | 2.62(1.03,4.81) | 2.85(1.05,5.23) | -0.06(-0.28,0.16) |
| Democratic People's Republic of Korea | male | 61.78(22.90,112.09) | 140.48(52.07,249.94) | 2.89(2.77,3.01) | 1.63(0.56,3.11) | 1.40(0.53,2.58) | -0.31(-0.45,-0.18) |
| Democratic Republic of the Congo | male | 349.21(137.05,593.92) | 644.26(236.30,1122.41) | 1.64(1.32,1.96) | 7.31(2.90,12.51) | 6.59(2.56,11.38) | -0.55(-0.73,-0.37) |
| Denmark | male | 36.67(14.23,60.35) | 96.52(38.97,164.96) | 3.14(2.72,3.56) | 1.11(0.43,1.83) | 1.71(0.67,2.91) | 1.42(0.88,1.96) |
| Djibouti | male | 0.66(0.22,1.22) | 4.63(1.49,9.04) | 6.48(6.37,6.59) | 1.67(0.57,3.21) | 2.27(0.77,4.38) | 0.94(0.85,1.04) |
| Dominica | male | 0.74(0.23,1.36) | 1.08(0.41,2.06) | 0.83(0.60,1.06) | 3.57(1.13,6.52) | 3.92(1.41,7.57) | 0.12(0.03,0.21) |
| Dominican Republic | male | 34.10(13.19,56.91) | 126.87(50.02,220.64) | 4.81(4.46,5.16) | 2.18(0.85,3.75) | 2.75(1.06,4.78) | 1.35(1.08,1.62) |
| Ecuador | male | 46.60(18.79,80.08) | 215.30(81.92,378.36) | 5.21(4.39,6.04) | 2.03(0.80,3.53) | 3.07(1.13,5.24) | 1.41(0.66,2.17) |
| Egypt | male | 266.79(101.16,452.61) | 1063.54(411.13,1851.02) | 4.87(4.70,5.04) | 2.54(0.93,4.39) | 4.24(1.62,7.32) | 2.05(1.83,2.27) |
| El Salvador | male | 19.62(7.34,35.79) | 86.52(29.97,161.68) | 4.80(4.21,5.40) | 1.53(0.58,2.82) | 3.23(1.12,5.98) | 2.29(1.76,2.82) |
| Equatorial Guinea | male | 2.98(1.13,5.44) | 9.71(3.57,18.06) | 4.46(3.75,5.18) | 4.92(1.93,9.00) | 6.73(2.39,12.67) | 1.19(0.77,1.61) |
| Eritrea | male | 2.65(0.74,5.87) | 7.32(2.33,15.39) | 3.53(3.44,3.62) | 0.89(0.26,1.90) | 1.06(0.32,2.32) | 0.48(0.35,0.62) |
| Estonia | male | 1.55(0.57,2.71) | 9.39(3.85,16.00) | 5.39(4.45,6.34) | 0.27(0.10,0.47) | 0.95(0.39,1.62) | 3.50(2.66,4.35) |
| Eswatini | male | 6.24(2.35,11.09) | 17.04(6.07,31.11) | 3.48(2.79,4.18) | 6.87(2.63,11.85) | 9.84(3.67,17.54) | 1.64(1.11,2.18) |
| Ethiopia | male | 232.70(85.23,454.35) | 348.78(113.84,649.41) | 1.05(0.77,1.33) | 2.93(1.03,5.60) | 2.03(0.63,3.79) | -1.44(-1.64,-1.25) |
| Fiji | male | 19.47(7.48,34.43) | 55.44(22.36,101.03) | 3.44(3.27,3.61) | 13.93(5.23,24.79) | 20.10(7.40,36.42) | 0.90(0.59,1.22) |
| Finland | male | 16.09(6.86,27.51) | 36.20(14.33,60.00) | 2.62(2.34,2.89) | 0.70(0.30,1.18) | 0.59(0.23,0.96) | -0.64(-0.86,-0.42) |
| France | male | 450.21(191.41,756.07) | 943.87(398.18,1565.48) | 2.65(2.21,3.09) | 1.55(0.65,2.64) | 1.33(0.56,2.20) | -0.41(-0.92,0.10) |
| Gabon | male | 4.73(1.63,9.13) | 9.07(2.93,19.21) | 2.01(1.81,2.21) | 2.64(0.84,5.26) | 3.26(0.95,6.89) | 0.57(0.47,0.67) |
| Gambia | male | 1.73(0.68,3.34) | 6.67(2.20,13.58) | 4.37(4.15,4.59) | 1.43(0.54,2.70) | 1.94(0.63,3.85) | 0.87(0.77,0.97) |
| Georgia | male | 14.22(5.25,23.89) | 31.67(11.92,54.03) | 4.03(3.29,4.77) | 0.70(0.26,1.14) | 1.41(0.53,2.40) | 3.63(2.98,4.28) |
| Germany | male | 478.09(168.49,869.22) | 977.77(369.87,1778.58) | 2.32(2.09,2.55) | 1.12(0.39,2.06) | 1.03(0.39,1.84) | -0.51(-0.71,-0.32) |
| Ghana | male | 45.77(16.82,79.74) | 207.51(77.28,359.68) | 5.59(5.30,5.89) | 2.32(0.84,4.25) | 4.10(1.47,7.28) | 2.32(2.05,2.58) |
| Greece | male | 76.40(28.61,136.28) | 171.88(64.44,309.99) | 2.49(1.79,3.20) | 1.29(0.47,2.30) | 1.20(0.46,2.16) | -0.53(-1.20,0.15) |
| Greenland | male | 0.11(0.04,0.19) | 0.22(0.08,0.39) | 2.92(2.70,3.14) | 1.17(0.40,2.08) | 0.88(0.31,1.58) | -0.62(-0.74,-0.49) |
| Grenada | male | 1.21(0.43,2.10) | 2.41(0.86,4.56) | 1.37(0.63,2.11) | 3.95(1.41,6.88) | 6.82(2.69,12.40) | 1.93(0.70,3.18) |
| Guam | male | 0.46(0.17,0.83) | 1.11(0.42,1.95) | 3.46(3.19,3.73) | 1.79(0.68,3.17) | 1.14(0.43,2.08) | -0.76(-1.05,-0.47) |
| Guatemala | male | 11.15(3.85,20.65) | 81.68(30.22,157.65) | 6.62(6.28,6.96) | 1.10(0.34,2.17) | 1.85(0.67,3.55) | 1.38(1.04,1.72) |
| Guinea | male | 12.42(3.91,24.78) | 24.02(7.67,49.14) | 2.22(1.95,2.49) | 0.96(0.28,1.91) | 1.13(0.35,2.37) | 0.65(0.52,0.78) |
| Guinea-Bissau | male | 4.74(1.63,8.58) | 7.17(2.64,12.79) | 1.42(1.36,1.48) | 3.12(1.12,5.75) | 3.11(1.12,5.52) | 0.01(-0.09,0.12) |
| Guyana | male | 6.84(2.62,11.94) | 15.14(5.85,26.99) | 3.07(2.82,3.32) | 4.48(1.73,7.60) | 6.09(2.29,10.85) | 1.23(0.89,1.58) |
| Haiti | male | 43.90(15.86,81.70) | 81.71(27.08,154.43) | 2.22(2.13,2.32) | 3.87(1.25,7.09) | 3.20(1.02,6.29) | -0.48(-0.61,-0.36) |
| Honduras | male | 6.65(2.31,12.54) | 34.23(12.57,62.47) | 6.12(5.82,6.41) | 0.83(0.28,1.55) | 1.37(0.50,2.52) | 2.09(1.84,2.34) |
| Hungary | male | 40.68(14.39,68.35) | 96.17(35.38,167.46) | 3.37(2.95,3.80) | 0.76(0.27,1.31) | 1.25(0.46,2.19) | 2.06(1.65,2.48) |
| Iceland | male | 0.92(0.36,1.54) | 2.21(0.84,3.80) | 3.16(3.01,3.31) | 0.72(0.28,1.21) | 0.75(0.28,1.29) | 0.41(0.17,0.65) |
| India | male | 4005.59(1644.77,6440.44) | 14053.98(5952.64,22330.44) | 4.22(4.06,4.39) | 2.39(0.98,3.91) | 3.23(1.37,5.34) | 1.18(0.94,1.42) |
| Indonesia | male | 1117.39(457.93,1770.18) | 4127.15(1754.11,6644.64) | 4.21(4.17,4.26) | 3.06(1.22,4.84) | 4.72(2.05,7.51) | 1.49(1.43,1.56) |
| Iran (Islamic Republic of) | male | 132.85(48.50,220.18) | 643.60(247.83,1094.12) | 5.95(5.71,6.19) | 1.45(0.53,2.53) | 1.90(0.72,3.23) | 1.28(1.08,1.49) |
| Iraq | male | 200.17(80.74,353.18) | 622.12(249.03,1066.50) | 3.55(3.16,3.94) | 5.66(2.27,9.98) | 6.86(2.74,11.59) | 0.20(-0.14,0.53) |
| Ireland | male | 27.70(11.49,44.14) | 41.56(16.71,69.41) | 1.46(1.30,1.61) | 1.77(0.74,2.85) | 1.15(0.47,1.94) | -1.19(-1.44,-0.93) |
| Israel | male | 48.84(19.36,83.23) | 127.20(48.03,220.99) | 2.66(1.83,3.50) | 2.40(0.96,4.08) | 2.14(0.81,3.71) | -0.75(-1.56,0.07) |
| Italy | male | 757.89(311.00,1201.76) | 1284.20(524.29,2169.07) | 2.03(1.89,2.16) | 2.19(0.91,3.53) | 1.73(0.71,2.89) | -0.54(-0.68,-0.39) |
| Jamaica | male | 40.76(15.62,67.32) | 75.46(30.21,134.56) | 2.03(1.53,2.53) | 4.87(1.86,8.03) | 5.29(2.09,9.47) | 0.34(-0.13,0.80) |
| Japan | male | 792.78(307.20,1258.08) | 1508.97(559.81,2752.89) | 2.30(1.98,2.63) | 1.27(0.49,2.05) | 0.76(0.29,1.35) | -1.50(-1.80,-1.20) |
| Jordan | male | 21.46(8.63,37.35) | 123.24(46.09,210.91) | 5.63(5.38,5.88) | 4.19(1.65,7.41) | 4.21(1.57,7.32) | -0.09(-0.19,0.01) |
| Kazakhstan | male | 11.98(4.40,20.19) | 34.39(12.69,59.40) | 2.38(1.91,2.86) | 0.28(0.11,0.48) | 0.59(0.22,1.03) | 1.47(1.02,1.92) |
| Kenya | male | 52.09(20.71,91.29) | 194.31(72.00,344.75) | 4.31(4.21,4.40) | 1.63(0.61,2.93) | 2.54(0.92,4.73) | 1.41(1.22,1.61) |
| Kiribati | male | 2.25(0.85,3.76) | 4.72(1.84,8.39) | 2.27(2.10,2.44) | 15.41(5.72,26.38) | 17.32(6.66,30.85) | 0.17(-0.09,0.43) |
| Kuwait | male | 8.26(3.62,13.24) | 54.47(21.61,91.64) | 5.76(5.07,6.45) | 2.99(1.29,4.84) | 4.31(1.68,7.25) | 0.66(0.00,1.33) |
| Kyrgyzstan | male | 2.46(0.99,4.30) | 7.56(2.64,13.60) | 2.46(1.97,2.96) | 0.26(0.10,0.45) | 0.49(0.20,0.87) | 1.21(0.73,1.70) |
| Lao People's Democratic Republic | male | 12.20(4.31,23.72) | 21.76(7.73,41.41) | 1.58(1.34,1.82) | 1.56(0.53,2.97) | 1.31(0.44,2.67) | -0.72(-0.87,-0.58) |
| Latvia | male | 3.10(1.15,5.40) | 12.17(4.82,21.99) | 4.13(3.34,4.92) | 0.27(0.10,0.48) | 0.86(0.34,1.55) | 3.45(2.66,4.24) |
| Lebanon | male | 46.50(18.81,81.45) | 157.24(62.08,271.80) | 4.99(4.60,5.38) | 5.46(2.17,9.43) | 5.27(2.09,8.95) | 0.46(0.14,0.78) |
| Lesotho | male | 4.46(1.56,8.29) | 8.35(2.90,17.45) | 2.04(1.75,2.33) | 2.32(0.78,4.38) | 3.41(1.21,6.58) | 1.80(1.49,2.11) |
| Liberia | male | 11.41(4.30,21.66) | 22.64(8.37,40.68) | 2.26(2.01,2.51) | 2.22(0.88,4.25) | 2.60(0.90,4.83) | 0.60(0.45,0.75) |
| Libya | male | 15.54(6.25,26.18) | 66.60(24.03,120.43) | 5.54(5.30,5.78) | 1.84(0.75,3.14) | 3.09(1.12,5.50) | 2.46(2.17,2.76) |
| Lithuania | male | 4.13(1.67,7.11) | 22.12(8.76,36.82) | 4.73(3.76,5.70) | 0.26(0.11,0.44) | 1.05(0.42,1.75) | 3.71(2.75,4.68) |
| Luxembourg | male | 2.65(1.10,4.40) | 4.48(1.78,7.93) | 1.80(1.63,1.97) | 1.49(0.63,2.54) | 0.96(0.38,1.70) | -1.36(-1.58,-1.14) |
| Madagascar | male | 27.01(8.60,51.24) | 44.99(16.69,87.72) | 1.73(1.43,2.03) | 1.40(0.43,2.71) | 1.37(0.49,2.75) | -0.04(-0.09,0.01) |
| Malawi | male | 34.43(13.31,62.28) | 75.34(27.86,141.25) | 2.22(2.04,2.41) | 2.54(0.99,4.60) | 3.13(1.19,5.75) | 0.51(0.24,0.79) |
| Malaysia | male | 105.41(41.91,179.66) | 307.30(130.56,505.98) | 3.25(3.00,3.51) | 2.63(1.07,4.46) | 2.35(0.99,3.85) | -0.77(-1.02,-0.52) |
| Maldives | male | 2.64(1.05,4.27) | 5.34(2.19,8.52) | 1.88(1.61,2.14) | 6.05(2.35,9.70) | 3.56(1.46,5.70) | -1.97(-2.13,-1.81) |
| Mali | male | 37.21(15.49,65.75) | 92.58(37.60,172.90) | 3.50(3.27,3.73) | 2.51(1.06,4.70) | 2.68(1.03,5.15) | 0.41(0.32,0.51) |
| Malta | male | 4.70(1.94,7.46) | 8.54(3.26,13.80) | 2.20(1.88,2.53) | 2.98(1.19,4.81) | 1.82(0.71,2.96) | -1.31(-1.67,-0.94) |
| Marshall Islands | male | 0.99(0.36,1.78) | 2.53(0.83,5.72) | 3.32(3.16,3.48) | 13.71(5.01,24.22) | 15.83(5.37,35.20) | 0.57(0.41,0.72) |
| Mauritania | male | 15.07(5.43,26.23) | 40.84(15.54,73.94) | 3.15(2.96,3.35) | 3.84(1.44,6.69) | 4.47(1.70,8.16) | 0.24(0.06,0.41) |
| Mauritius | male | 9.85(3.78,17.26) | 65.85(26.35,111.54) | 8.09(7.13,9.06) | 3.53(1.42,6.14) | 8.48(3.51,14.32) | 4.43(3.46,5.40) |
| Mexico | male | 685.63(255.18,1120.76) | 2608.98(972.95,4451.72) | 4.71(4.45,4.97) | 3.90(1.44,6.41) | 4.77(1.77,8.20) | 0.84(0.62,1.06) |
| Micronesia (Federated States of) | male | 3.37(1.39,5.73) | 5.99(2.38,9.97) | 1.80(1.72,1.88) | 15.92(6.57,27.07) | 19.01(7.76,31.30) | 0.49(0.27,0.71) |
| Monaco | male | 0.23(0.10,0.40) | 0.46(0.17,0.79) | 2.42(2.12,2.72) | 0.76(0.33,1.33) | 0.89(0.34,1.53) | 0.63(0.40,0.87) |
| Mongolia | male | 1.41(0.48,2.73) | 3.65(1.32,6.90) | 3.06(2.65,3.47) | 0.37(0.12,0.72) | 0.47(0.18,0.88) | 0.58(0.44,0.72) |
| Montenegro | male | 2.60(1.00,4.58) | 5.95(2.31,10.76) | 3.29(3.03,3.55) | 1.10(0.43,1.98) | 1.64(0.63,3.04) | 1.44(0.98,1.89) |
| Morocco | male | 104.83(40.48,195.97) | 402.48(166.30,661.56) | 4.73(4.36,5.11) | 1.72(0.64,3.33) | 2.82(1.15,4.70) | 1.84(1.55,2.12) |
| Mozambique | male | 33.84(10.76,66.76) | 78.10(26.26,150.85) | 3.19(3.03,3.36) | 1.70(0.54,3.37) | 2.33(0.78,4.47) | 1.52(1.34,1.70) |
| Myanmar | male | 110.40(30.94,232.17) | 238.80(78.14,490.00) | 2.43(2.17,2.70) | 1.30(0.36,2.71) | 1.45(0.45,3.01) | 0.28(0.09,0.47) |
| Namibia | male | 12.18(4.97,21.32) | 32.71(11.85,55.70) | 2.95(2.58,3.32) | 5.49(2.12,9.54) | 7.40(2.72,12.57) | 0.88(0.58,1.19) |
| Nauru | male | 0.18(0.06,0.32) | 0.21(0.08,0.37) | 0.08(-0.14,0.29) | 7.75(2.68,14.40) | 9.40(3.42,16.35) | 0.37(0.22,0.52) |
| Nepal | male | 41.42(14.30,80.31) | 164.34(63.58,324.50) | 5.05(4.77,5.33) | 1.23(0.42,2.42) | 1.90(0.70,3.86) | 1.62(1.35,1.89) |
| Netherlands | male | 95.01(31.49,172.52) | 152.58(50.61,282.65) | 1.48(1.22,1.75) | 1.32(0.44,2.42) | 0.98(0.33,1.83) | -1.14(-1.46,-0.82) |
| New Zealand | male | 20.87(8.03,33.80) | 53.50(21.06,88.73) | 2.52(2.03,3.00) | 1.38(0.53,2.28) | 1.34(0.53,2.24) | -0.75(-1.26,-0.23) |
| Nicaragua | male | 8.59(3.31,16.20) | 39.08(14.00,74.77) | 5.23(4.81,5.66) | 1.59(0.60,3.02) | 2.03(0.73,3.89) | 1.00(0.70,1.29) |
| Niger | male | 16.78(6.14,31.50) | 48.90(17.33,93.44) | 3.68(3.60,3.75) | 1.69(0.59,3.36) | 1.72(0.60,3.22) | 0.15(0.07,0.24) |
| Nigeria | male | 471.52(175.33,812.24) | 944.61(338.06,1676.07) | 2.17(2.10,2.25) | 2.89(1.05,4.98) | 3.02(0.99,5.49) | 0.06(0.01,0.10) |
| Niue | male | 0.05(0.02,0.09) | 0.08(0.03,0.16) | 1.12(0.90,1.33) | 6.01(2.38,10.38) | 8.92(3.22,17.24) | 1.20(1.01,1.39) |
| North Macedonia | male | 9.29(3.49,16.17) | 25.44(9.54,44.86) | 3.72(3.39,4.06) | 1.25(0.48,2.17) | 2.07(0.77,3.65) | 1.83(1.32,2.36) |
| Northern Mariana Islands | male | 0.40(0.14,0.70) | 1.26(0.50,2.20) | 4.14(3.72,4.56) | 5.35(1.99,9.98) | 5.85(2.23,10.09) | 0.32(0.03,0.60) |
| Norway | male | 24.50(9.95,39.92) | 45.27(17.99,74.99) | 1.78(1.37,2.20) | 0.85(0.35,1.42) | 0.93(0.37,1.55) | 0.12(-0.40,0.64) |
| Oman | male | 12.13(5.01,22.12) | 48.67(19.51,80.50) | 5.02(4.77,5.26) | 4.96(1.98,8.97) | 7.96(3.28,13.11) | 2.27(1.90,2.65) |
| Pakistan | male | 612.62(238.04,1079.53) | 1609.00(613.76,3092.12) | 2.83(2.63,3.03) | 2.28(0.88,4.14) | 3.24(1.25,6.27) | 0.88(0.61,1.14) |
| Palau | male | 0.25(0.09,0.46) | 0.67(0.26,1.18) | 2.91(2.54,3.28) | 5.93(2.17,11.22) | 6.34(2.62,11.55) | 0.20(0.00,0.41) |
| Palestine | male | 18.38(7.08,31.33) | 44.53(17.28,75.00) | 2.87(2.74,3.00) | 6.38(2.51,10.94) | 6.14(2.23,10.25) | -0.12(-0.43,0.20) |
| Panama | male | 7.46(2.60,13.38) | 40.47(15.28,71.44) | 5.44(5.08,5.79) | 1.12(0.39,2.03) | 1.94(0.73,3.42) | 1.58(1.22,1.93) |
| Papua New Guinea | male | 47.52(16.95,90.31) | 122.22(41.99,229.71) | 3.09(2.93,3.25) | 6.17(2.14,11.28) | 5.59(1.91,10.54) | -0.36(-0.41,-0.30) |
| Paraguay | male | 17.16(6.41,31.49) | 88.62(32.00,175.48) | 5.89(5.62,6.15) | 1.93(0.71,3.58) | 3.72(1.35,7.42) | 2.63(2.44,2.83) |
| Peru | male | 94.57(37.31,161.87) | 292.38(112.95,516.89) | 3.93(3.73,4.14) | 1.83(0.72,3.11) | 1.89(0.73,3.33) | 0.00(-0.21,0.22) |
| Philippines | male | 150.09(56.64,256.24) | 632.35(241.32,1194.43) | 4.95(4.86,5.04) | 1.38(0.51,2.45) | 2.00(0.76,3.70) | 1.56(1.46,1.65) |
| Poland | male | 181.44(72.29,313.52) | 397.61(169.55,680.41) | 2.89(2.51,3.27) | 1.15(0.45,1.95) | 1.40(0.60,2.40) | 0.73(0.48,0.99) |
| Portugal | male | 147.93(61.05,240.96) | 254.03(92.97,414.27) | 1.47(1.03,1.92) | 3.01(1.24,5.05) | 2.10(0.78,3.44) | -1.57(-2.01,-1.12) |
| Puerto Rico | male | 82.06(32.99,136.77) | 166.75(63.82,276.70) | 2.11(1.89,2.33) | 5.42(2.19,8.98) | 4.69(1.81,7.72) | -0.56(-0.75,-0.38) |
| Qatar | male | 4.21(1.61,7.02) | 28.98(11.23,50.61) | 7.11(6.62,7.60) | 12.16(4.66,19.83) | 11.05(4.05,19.39) | -0.56(-1.45,0.34) |
| Republic of Korea | male | 219.29(91.81,363.47) | 562.91(217.95,994.47) | 2.75(2.29,3.22) | 2.51(1.08,4.19) | 1.60(0.60,2.85) | -1.83(-2.37,-1.28) |
| Republic of Moldova | male | 4.79(1.92,8.36) | 12.83(4.73,22.51) | 2.40(1.41,3.40) | 0.34(0.14,0.56) | 0.57(0.21,1.00) | 0.80(-0.22,1.83) |
| Romania | male | 65.45(27.42,108.13) | 119.30(48.70,202.56) | 2.51(1.99,3.02) | 0.61(0.25,0.98) | 0.75(0.31,1.27) | 1.09(0.60,1.57) |
| Russian Federation | male | 110.02(42.24,181.79) | 745.13(287.14,1222.45) | 5.76(4.05,7.51) | 0.25(0.10,0.41) | 0.96(0.37,1.56) | 3.94(2.19,5.71) |
| Rwanda | male | 21.42(7.00,45.66) | 34.84(11.55,75.97) | 0.95(0.33,1.58) | 2.37(0.80,5.04) | 2.10(0.66,5.07) | -1.15(-1.46,-0.84) |
| Saint Kitts and Nevis | male | 0.80(0.31,1.38) | 1.31(0.52,2.37) | 1.81(1.50,2.13) | 5.74(2.16,10.06) | 5.85(2.30,10.27) | 0.31(0.09,0.54) |
| Saint Lucia | male | 2.00(0.76,3.60) | 4.56(1.57,8.66) | 2.63(2.33,2.92) | 6.80(2.56,12.40) | 4.66(1.60,8.79) | -1.54(-1.80,-1.29) |
| Saint Vincent and the Grenadines | male | 1.36(0.50,2.33) | 3.26(1.15,5.80) | 2.69(2.45,2.93) | 5.38(1.95,9.30) | 5.31(1.83,9.59) | -0.05(-0.26,0.16) |
| Samoa | male | 2.22(0.83,3.90) | 4.02(1.55,7.35) | 1.79(1.68,1.90) | 6.20(2.44,10.76) | 6.46(2.39,11.60) | 0.03(-0.06,0.11) |
| San Marino | male | 0.16(0.06,0.27) | 0.26(0.10,0.46) | 3.18(2.38,3.99) | 1.08(0.42,1.80) | 0.52(0.21,0.95) | -0.81(-1.44,-0.19) |
| Sao Tome and Principe | male | 0.18(0.06,0.36) | 0.38(0.12,0.75) | 2.60(2.48,2.71) | 1.10(0.33,2.18) | 1.10(0.33,2.21) | 0.69(0.37,1.01) |
| Saudi Arabia | male | 90.88(35.39,155.00) | 442.69(178.12,741.22) | 4.81(4.62,5.00) | 3.55(1.40,6.04) | 5.38(2.13,8.98) | 1.08(0.91,1.25) |
| Senegal | male | 40.11(14.99,68.82) | 106.07(41.05,191.09) | 3.25(3.18,3.33) | 2.96(1.07,5.08) | 3.54(1.37,6.56) | 0.49(0.42,0.56) |
| Serbia | male | 56.29(20.01,97.18) | 122.83(42.78,225.48) | 3.02(2.81,3.23) | 1.61(0.57,2.87) | 1.68(0.58,3.12) | 0.55(0.36,0.74) |
| Seychelles | male | 0.39(0.16,0.68) | 1.08(0.41,1.90) | 3.49(3.30,3.69) | 1.78(0.71,3.06) | 2.54(0.93,4.37) | 1.30(1.02,1.58) |
| Sierra Leone | male | 16.13(6.01,30.15) | 27.13(10.12,51.02) | 1.55(1.45,1.66) | 1.86(0.65,3.49) | 1.88(0.68,3.61) | 0.04(-0.10,0.19) |
| Singapore | male | 12.42(5.24,21.11) | 16.07(6.07,29.10) | 0.89(0.36,1.43) | 1.48(0.58,2.56) | 0.46(0.17,0.84) | -3.54(-4.15,-2.92) |
| Slovakia | male | 18.38(7.22,32.30) | 25.42(9.75,45.21) | 1.27(1.15,1.39) | 0.80(0.30,1.42) | 0.72(0.27,1.25) | -0.11(-0.21,-0.02) |
| Slovenia | male | 6.08(2.47,10.35) | 16.29(6.19,29.21) | 1.91(1.35,2.48) | 0.68(0.28,1.19) | 0.85(0.32,1.53) | -0.85(-1.48,-0.21) |
| Solomon Islands | male | 5.30(1.97,9.87) | 14.97(5.94,25.06) | 3.34(3.27,3.41) | 8.09(3.29,14.82) | 9.84(3.82,16.36) | 0.66(0.61,0.72) |
| Somalia | male | 16.94(5.80,32.50) | 36.49(12.18,72.52) | 2.78(2.70,2.86) | 2.32(0.78,4.59) | 2.27(0.70,4.50) | 0.08(-0.02,0.18) |
| South Africa | male | 422.88(171.68,672.14) | 1481.35(613.45,2330.15) | 4.17(3.75,4.58) | 5.78(2.33,9.22) | 9.64(3.90,14.92) | 1.80(1.34,2.26) |
| South Sudan | male | 24.13(8.81,48.54) | 36.58(12.21,73.54) | 1.19(1.03,1.35) | 2.07(0.73,4.21) | 2.56(0.83,5.23) | 0.63(0.52,0.74) |
| Spain | male | 419.96(171.75,694.26) | 594.57(232.67,1006.54) | 1.17(0.99,1.34) | 2.07(0.83,3.46) | 1.20(0.47,2.01) | -1.84(-1.99,-1.70) |
| Sri Lanka | male | 47.90(15.90,92.79) | 139.41(45.45,293.16) | 4.21(3.97,4.46) | 1.15(0.37,2.41) | 1.37(0.43,3.00) | 1.42(1.10,1.74) |
| Sudan | male | 100.23(40.27,181.88) | 311.73(120.90,538.95) | 3.79(3.53,4.06) | 2.32(0.92,4.30) | 3.29(1.31,5.68) | 1.32(1.04,1.59) |
| Suriname | male | 4.50(1.88,7.51) | 13.58(5.46,23.00) | 4.05(3.80,4.30) | 3.89(1.61,6.65) | 4.87(1.98,8.26) | 1.00(0.78,1.22) |
| Sweden | male | 53.27(19.99,90.59) | 108.16(41.10,196.98) | 2.52(2.32,2.72) | 0.84(0.30,1.44) | 0.94(0.36,1.72) | 0.64(0.41,0.87) |
| Switzerland | male | 61.40(23.50,100.96) | 78.20(29.97,141.63) | 0.65(0.48,0.81) | 1.51(0.56,2.52) | 0.84(0.32,1.50) | -2.03(-2.22,-1.83) |
| Syrian Arab Republic | male | 56.98(21.82,99.57) | 148.38(58.42,254.46) | 3.08(2.79,3.37) | 2.50(0.98,4.36) | 2.73(1.10,4.80) | 0.05(-0.19,0.29) |
| Taiwan (Province of China) | male | 251.31(106.47,396.26) | 645.52(252.58,1087.69) | 2.40(1.94,2.88) | 3.80(1.62,6.20) | 3.27(1.31,5.50) | -1.08(-1.45,-0.70) |
| Tajikistan | male | 5.79(2.12,10.99) | 13.11(4.06,25.18) | 2.45(2.18,2.73) | 0.57(0.21,1.04) | 0.61(0.20,1.13) | -0.04(-0.44,0.36) |
| Thailand | male | 196.72(77.76,338.55) | 803.56(321.66,1389.75) | 4.26(4.05,4.48) | 1.68(0.66,2.82) | 1.70(0.68,2.89) | -0.36(-0.55,-0.17) |
| Timor-Leste | male | 0.47(0.15,1.01) | 1.80(0.55,3.70) | 4.90(4.66,5.14) | 0.53(0.16,1.18) | 0.54(0.16,1.15) | 0.21(-0.07,0.49) |
| Togo | male | 5.34(1.83,10.12) | 18.04(5.34,34.40) | 3.74(3.61,3.87) | 1.34(0.46,2.59) | 1.83(0.59,3.55) | 0.86(0.67,1.06) |
| Tokelau | male | 0.03(0.01,0.05) | 0.04(0.01,0.06) | 1.04(0.89,1.19) | 4.14(1.53,7.75) | 5.08(2.11,9.08) | 0.64(0.51,0.77) |
| Tonga | male | 1.05(0.39,1.92) | 1.98(0.70,3.57) | 1.83(1.45,2.21) | 4.41(1.58,8.40) | 5.67(2.00,10.10) | 0.65(0.31,0.98) |
| Trinidad and Tobago | male | 44.05(17.35,74.19) | 106.13(45.50,186.70) | 2.79(2.60,2.98) | 12.42(4.87,19.78) | 11.89(5.13,20.87) | -0.25(-0.44,-0.07) |
| Tunisia | male | 27.75(10.69,47.25) | 126.31(51.38,227.70) | 5.13(4.97,5.30) | 1.50(0.58,2.53) | 2.45(1.03,4.42) | 1.71(1.55,1.88) |
| Türkiye | male | 475.43(195.74,780.51) | 1172.59(463.23,2023.98) | 3.09(2.84,3.33) | 3.91(1.63,6.50) | 3.16(1.26,5.45) | -0.82(-0.98,-0.66) |
| Turkmenistan | male | 3.33(1.39,5.78) | 17.14(6.73,29.87) | 4.75(4.38,5.11) | 0.52(0.22,0.88) | 1.24(0.47,2.22) | 2.00(1.60,2.41) |
| Tuvalu | male | 0.13(0.05,0.24) | 0.25(0.09,0.43) | 2.09(1.99,2.19) | 5.24(1.94,9.79) | 5.90(2.24,10.64) | 0.44(0.32,0.56) |
| Uganda | male | 45.13(15.11,89.98) | 99.27(32.99,210.83) | 2.19(2.02,2.36) | 1.94(0.62,4.16) | 2.36(0.80,5.41) | 0.33(0.17,0.50) |
| Ukraine | male | 24.40(9.17,45.03) | 35.48(13.07,68.32) | 0.09(-0.50,0.69) | 0.10(0.04,0.19) | 0.13(0.05,0.25) | -0.40(-0.94,0.15) |
| United Arab Emirates | male | 8.81(3.09,16.23) | 65.79(25.12,110.62) | 7.23(6.97,7.50) | 4.75(1.81,8.70) | 4.00(1.50,6.92) | 1.01(0.26,1.77) |
| United Kingdom | male | 612.80(254.82,938.31) | 600.52(248.78,938.66) | -0.04(-0.39,0.32) | 1.80(0.76,2.78) | 0.94(0.39,1.46) | -2.17(-2.50,-1.84) |
| United Republic of Tanzania | male | 27.22(9.79,51.99) | 60.10(19.17,121.56) | 1.99(1.75,2.22) | 0.72(0.26,1.48) | 0.68(0.20,1.43) | -0.67(-0.89,-0.46) |
| United States of America | male | 1631.81(637.69,2890.44) | 4942.79(2047.04,8234.30) | 3.20(2.96,3.43) | 1.31(0.52,2.34) | 1.87(0.77,3.14) | 0.70(0.47,0.94) |
| United States Virgin Islands | male | 0.69(0.26,1.18) | 2.09(0.81,3.80) | 3.61(3.30,3.93) | 2.75(1.05,4.75) | 2.95(1.14,5.31) | 0.38(0.13,0.63) |
| Uruguay | male | 20.06(7.62,35.76) | 32.62(10.76,57.49) | 1.54(1.42,1.67) | 1.25(0.47,2.22) | 1.37(0.46,2.41) | 0.29(0.16,0.42) |
| Uzbekistan | male | 6.64(2.29,12.64) | 47.12(15.68,91.67) | 6.21(5.68,6.75) | 0.16(0.05,0.32) | 0.52(0.18,0.96) | 3.68(3.04,4.33) |
| Vanuatu | male | 0.45(0.15,0.92) | 1.23(0.38,2.39) | 3.08(2.99,3.17) | 1.86(0.59,3.83) | 1.97(0.62,4.01) | 0.07(-0.03,0.17) |
| Venezuela (Bolivarian Republic of) | male | 70.79(25.63,132.40) | 362.26(128.21,668.76) | 5.41(5.18,5.64) | 1.81(0.67,3.31) | 3.00(1.02,5.63) | 1.54(1.30,1.77) |
| Viet Nam | male | 199.62(65.53,366.64) | 617.52(227.64,1122.91) | 3.82(3.74,3.90) | 1.48(0.49,2.77) | 1.89(0.71,3.47) | 0.93(0.88,0.97) |
| Yemen | male | 29.97(11.48,58.82) | 91.42(33.84,165.03) | 3.78(3.68,3.88) | 1.84(0.71,3.70) | 1.73(0.64,3.14) | -0.20(-0.36,-0.05) |
| Zambia | male | 35.21(13.58,62.92) | 76.38(28.38,144.45) | 2.02(1.81,2.24) | 2.95(1.14,5.57) | 3.13(1.08,5.75) | -0.15(-0.37,0.06) |
| Zimbabwe | male | 33.96(13.53,62.52) | 64.52(23.80,117.43) | 1.61(1.10,2.13) | 2.48(0.98,4.40) | 3.02(1.17,5.35) | 0.64(0.24,1.04) |

Supplementary table3 DLAY rate of diabetes and kidney diseases due to Low physical activity all ages.

| DALYs (Disability-Adjusted Life Years) | 25-29 years | 30-34 years | 35-39 years | 40-44 years | 45-49 years | 50-54 years | 55-59 years | 60-64 years | 65-69 years | 70-74 years | 75-79 years | 80-84 years | 85-89 years | 90-94 years | 95+ years |
| --- | --- | --- | --- | --- | --- | --- | --- | --- | --- | --- | --- | --- | --- | --- | --- |
| Afghanistan | 27.58 | 40.48 | 63.93 | 115.81 | 238.70 | 446.16 | 732.99 | 1040.64 | 1261.94 | 1396.01 | 1479.92 | 1565.25 | 1512.30 | 1423.10 | 1234.19 |
| Albania | 0.51 | 1.68 | 4.09 | 8.09 | 17.10 | 30.26 | 51.43 | 85.54 | 132.95 | 186.22 | 276.25 | 358.57 | 375.31 | 446.18 | 556.19 |
| Algeria | 15.37 | 23.96 | 39.82 | 69.85 | 131.65 | 224.94 | 338.51 | 495.43 | 616.06 | 758.96 | 941.13 | 1137.08 | 2105.09 | 2039.17 | 1522.29 |
| American Samoa | 121.03 | 199.34 | 338.19 | 593.09 | 961.23 | 1529.77 | 2114.01 | 2661.26 | 3053.12 | 3208.47 | 3249.35 | 3551.17 | 3762.46 | 4235.85 | 5173.53 |
| Andorra | 4.13 | 7.37 | 10.85 | 16.44 | 27.03 | 45.19 | 72.14 | 112.97 | 169.16 | 250.56 | 358.01 | 517.04 | 657.60 | 871.49 | 1120.05 |
| Angola | 5.94 | 9.31 | 16.72 | 30.36 | 56.45 | 117.48 | 185.87 | 312.68 | 418.19 | 632.07 | 774.39 | 1012.27 | 1093.07 | 1155.58 | 1173.84 |
| Antigua and Barbuda | 10.16 | 17.04 | 30.06 | 58.02 | 125.50 | 244.93 | 417.23 | 668.85 | 935.19 | 1277.00 | 1678.50 | 2170.90 | 2248.21 | 2511.46 | 3420.25 |
| Argentina | 1.57 | 2.37 | 5.24 | 11.07 | 23.67 | 50.62 | 94.59 | 154.41 | 219.55 | 303.65 | 381.17 | 574.82 | 688.05 | 827.90 | 956.40 |
| Armenia | 1.30 | 2.61 | 5.27 | 10.25 | 22.75 | 44.50 | 83.13 | 132.23 | 190.74 | 300.08 | 452.93 | 529.26 | 438.86 | 505.94 | 641.46 |
| Australia | 2.47 | 6.23 | 11.78 | 21.59 | 44.01 | 73.23 | 121.03 | 178.88 | 241.43 | 315.82 | 399.53 | 551.00 | 704.59 | 997.37 | 1374.47 |
| Austria | 1.72 | 3.10 | 5.42 | 8.71 | 14.90 | 26.82 | 49.87 | 89.71 | 149.54 | 229.56 | 356.94 | 540.25 | 825.66 | 1172.49 | 1684.36 |
| Azerbaijan | 1.79 | 3.14 | 6.13 | 10.80 | 25.29 | 51.52 | 98.46 | 172.60 | 247.48 | 353.60 | 453.22 | 486.95 | 373.43 | 404.78 | 458.62 |
| Bahrain | 14.01 | 26.72 | 44.95 | 75.70 | 143.35 | 245.73 | 364.91 | 521.36 | 697.55 | 917.67 | 1104.42 | 1473.03 | 1411.92 | 1668.40 | 1801.22 |
| Bangladesh | 19.50 | 30.16 | 46.52 | 86.36 | 177.67 | 360.91 | 720.53 | 1186.13 | 1785.58 | 2937.25 | 4476.30 | 5987.78 | 6403.48 | 6647.50 | 6938.34 |
| Barbados | 6.72 | 9.90 | 17.04 | 31.08 | 57.59 | 107.62 | 117.81 | 209.10 | 264.21 | 267.46 | 405.27 | 1113.53 | 1481.85 | 1704.87 | 1789.98 |
| Belarus | 27.04 | 47.44 | 75.70 | 128.36 | 260.66 | 438.70 | 664.46 | 938.58 | 1172.87 | 1571.47 | 2097.85 | 2663.60 | 3022.88 | 4053.86 | 4904.94 |
| Belgium | 0.71 | 1.81 | 3.89 | 6.77 | 14.16 | 23.48 | 38.77 | 61.37 | 92.61 | 131.86 | 176.60 | 239.00 | 256.69 | 320.58 | 374.80 |
| Belize | 4.58 | 8.02 | 12.90 | 20.38 | 31.87 | 49.03 | 81.41 | 123.36 | 171.53 | 237.73 | 357.50 | 493.89 | 615.83 | 835.23 | 1139.95 |
| Benin | 14.76 | 20.89 | 39.78 | 75.19 | 150.68 | 307.20 | 465.04 | 748.47 | 828.24 | 1136.37 | 1307.52 | 1586.50 | 1565.03 | 1741.93 | 2099.66 |
| Bermuda | 1.23 | 1.78 | 3.05 | 6.65 | 15.88 | 33.21 | 75.85 | 126.31 | 200.77 | 247.63 | 391.69 | 620.97 | 669.84 | 878.76 | 979.81 |
| Bhutan | 6.80 | 11.20 | 18.55 | 32.39 | 61.11 | 109.77 | 176.98 | 262.34 | 368.42 | 487.11 | 643.59 | 904.38 | 1026.87 | 1303.74 | 1511.08 |
| Bolivia (Plurinational State of) | 20.32 | 33.36 | 48.23 | 82.54 | 151.13 | 248.68 | 381.01 | 598.22 | 850.64 | 1081.82 | 1313.52 | 1414.72 | 1696.32 | 1983.57 | 2189.85 |
| Bosnia and Herzegovina | 3.26 | 5.50 | 11.94 | 29.83 | 61.66 | 120.27 | 218.90 | 428.04 | 578.93 | 804.82 | 1099.09 | 1365.10 | 1328.79 | 1429.14 | 1225.78 |
| Botswana | 1.09 | 2.10 | 4.65 | 9.60 | 28.52 | 67.09 | 167.44 | 312.47 | 438.24 | 635.76 | 967.95 | 1156.79 | 955.95 | 867.24 | 679.76 |
| Brazil | 4.80 | 8.03 | 15.28 | 32.67 | 86.85 | 190.72 | 429.81 | 859.67 | 1138.45 | 1455.12 | 1794.85 | 2067.03 | 1973.40 | 1863.69 | 1163.99 |
| Brunei Darussalam | 5.97 | 11.61 | 21.93 | 42.34 | 88.93 | 163.82 | 266.88 | 425.46 | 622.70 | 827.49 | 1059.44 | 1286.32 | 1418.52 | 1610.70 | 1744.90 |
| Bulgaria | 12.24 | 26.62 | 51.50 | 86.74 | 175.63 | 300.15 | 439.48 | 673.87 | 827.97 | 1076.74 | 1590.07 | 1904.82 | 1998.77 | 2347.93 | 4536.30 |
| Burkina Faso | 1.93 | 4.12 | 8.39 | 17.15 | 38.10 | 73.87 | 120.81 | 184.42 | 260.38 | 349.99 | 480.88 | 620.69 | 628.85 | 764.13 | 1040.60 |
| Burundi | 2.49 | 3.28 | 5.47 | 11.08 | 22.77 | 49.43 | 123.92 | 220.44 | 290.53 | 306.47 | 433.13 | 635.41 | 766.19 | 990.22 | 1082.10 |
| Cambodia | 1.62 | 2.81 | 5.60 | 10.90 | 22.78 | 44.19 | 92.93 | 166.78 | 225.85 | 318.78 | 423.57 | 592.26 | 692.33 | 755.99 | 842.41 |
| Cameroon | 4.89 | 6.83 | 11.10 | 19.31 | 35.63 | 66.51 | 111.37 | 180.73 | 267.54 | 355.65 | 539.81 | 861.91 | 920.76 | 1121.63 | 1089.45 |
| Canada | 0.41 | 0.96 | 2.34 | 5.18 | 12.18 | 25.84 | 47.78 | 83.21 | 152.10 | 235.08 | 371.59 | 567.09 | 583.48 | 605.36 | 644.27 |
| Cabo Verde | 8.61 | 10.87 | 17.01 | 34.61 | 64.20 | 132.16 | 225.13 | 381.71 | 528.37 | 571.73 | 813.55 | 1190.12 | 1292.97 | 1690.51 | 1795.77 |
| Central African Republic | 0.29 | 1.05 | 2.76 | 5.86 | 13.79 | 24.05 | 41.98 | 64.86 | 99.80 | 153.26 | 246.05 | 387.65 | 453.93 | 591.73 | 930.65 |
| Chad | 11.28 | 16.53 | 26.29 | 47.35 | 88.65 | 159.71 | 261.41 | 384.25 | 441.41 | 588.20 | 701.54 | 871.84 | 1011.66 | 1021.90 | 912.24 |
| Chile | 4.06 | 5.75 | 10.51 | 21.12 | 43.36 | 88.17 | 148.90 | 264.66 | 354.02 | 389.44 | 497.34 | 643.63 | 688.54 | 864.61 | 933.77 |
| China | 1.66 | 3.33 | 6.83 | 12.43 | 25.56 | 51.41 | 100.03 | 184.81 | 225.25 | 273.06 | 342.46 | 478.47 | 542.49 | 755.81 | 1228.75 |
| Colombia | 8.24 | 10.80 | 12.50 | 17.66 | 27.30 | 45.46 | 80.86 | 131.05 | 200.63 | 304.47 | 393.94 | 503.34 | 589.63 | 690.76 | 721.40 |
| Comoros | 16.12 | 25.87 | 42.62 | 67.84 | 117.10 | 176.49 | 266.01 | 358.45 | 435.53 | 465.22 | 485.73 | 558.81 | 577.29 | 596.27 | 516.54 |
| Congo | 2.06 | 3.48 | 8.03 | 14.09 | 26.10 | 51.13 | 102.04 | 186.64 | 253.42 | 357.27 | 423.63 | 485.57 | 575.93 | 629.58 | 701.67 |
| Costa Rica | 8.05 | 11.66 | 21.92 | 45.50 | 79.83 | 169.07 | 254.09 | 409.12 | 416.57 | 536.05 | 631.31 | 855.02 | 1023.29 | 1153.44 | 1352.93 |
| Côte d'Ivoire | 42.54 | 69.54 | 124.93 | 232.91 | 419.46 | 702.61 | 984.01 | 1343.84 | 1395.20 | 1575.43 | 1798.04 | 2235.21 | 2479.95 | 2881.08 | 3498.24 |
| Croatia | 6.76 | 10.01 | 17.58 | 30.81 | 59.73 | 101.13 | 170.12 | 246.41 | 333.22 | 390.42 | 522.68 | 720.02 | 763.06 | 851.00 | 756.97 |
| Cuba | 0.88 | 2.24 | 4.96 | 10.80 | 25.84 | 47.95 | 81.67 | 149.23 | 230.31 | 365.17 | 638.76 | 889.57 | 988.25 | 1058.38 | 1199.34 |
| Cyprus | 6.99 | 12.20 | 20.04 | 34.69 | 63.04 | 105.61 | 170.14 | 256.21 | 350.73 | 435.39 | 543.41 | 670.42 | 679.10 | 776.79 | 861.13 |
| Czechia | 3.72 | 6.45 | 9.74 | 15.31 | 28.89 | 52.40 | 105.40 | 195.25 | 319.40 | 502.24 | 686.76 | 1191.01 | 1599.70 | 3723.69 | 6855.46 |
| Democratic Republic of the Congo | 0.82 | 2.32 | 5.21 | 13.35 | 30.76 | 58.14 | 104.49 | 185.80 | 362.01 | 537.87 | 694.38 | 931.48 | 1172.92 | 1222.24 | 1180.70 |
| Denmark | 6.40 | 8.70 | 13.07 | 26.62 | 49.68 | 104.79 | 189.49 | 322.51 | 446.72 | 475.25 | 654.98 | 859.40 | 933.29 | 1195.91 | 1321.11 |
| Djibouti | 6.40 | 9.20 | 13.66 | 24.11 | 45.61 | 78.50 | 133.53 | 207.75 | 281.43 | 383.46 | 509.96 | 584.90 | 606.63 | 646.69 | 739.86 |
| Dominica | 9.09 | 12.44 | 17.69 | 29.95 | 63.16 | 127.07 | 204.91 | 354.37 | 676.44 | 947.03 | 1113.64 | 1364.67 | 1607.42 | 1716.01 | 1938.50 |
| Dominican Republic | 2.56 | 4.24 | 6.64 | 10.30 | 18.69 | 31.29 | 55.99 | 89.43 | 137.82 | 203.76 | 329.86 | 533.45 | 782.80 | 1091.07 | 1373.98 |
| Ecuador | 1.52 | 2.74 | 6.09 | 12.27 | 25.04 | 48.55 | 99.54 | 181.87 | 241.37 | 361.33 | 486.34 | 684.90 | 843.78 | 930.07 | 1042.96 |
| Egypt | 12.40 | 19.17 | 32.68 | 55.48 | 104.34 | 176.18 | 275.24 | 410.18 | 656.76 | 1097.03 | 1661.86 | 2493.55 | 2623.64 | 2836.44 | 3151.18 |
| El Salvador | 10.95 | 18.70 | 34.00 | 65.43 | 125.70 | 219.74 | 357.04 | 549.12 | 635.49 | 707.14 | 822.57 | 984.35 | 1016.22 | 1119.53 | 1632.72 |
| Equatorial Guinea | 3.54 | 6.75 | 14.09 | 32.45 | 61.62 | 111.28 | 188.43 | 387.41 | 457.77 | 507.77 | 560.64 | 606.47 | 730.65 | 1014.34 | 1900.94 |
| Eritrea | 11.92 | 20.65 | 34.43 | 62.38 | 130.92 | 254.85 | 421.27 | 645.45 | 960.85 | 1339.83 | 1660.57 | 2340.95 | 2366.41 | 1334.20 | 1043.49 |
| Estonia | 9.80 | 15.58 | 28.28 | 53.01 | 100.96 | 189.91 | 319.12 | 461.59 | 574.70 | 669.04 | 778.28 | 970.96 | 953.49 | 1121.36 | 983.71 |
| Ethiopia | 11.06 | 15.53 | 24.07 | 44.90 | 75.46 | 156.62 | 246.82 | 408.21 | 565.72 | 812.20 | 1090.61 | 1467.48 | 1738.88 | 1885.32 | 2155.63 |
| Micronesia (Federated States of) | 0.92 | 1.90 | 3.44 | 7.92 | 17.48 | 32.79 | 70.79 | 115.87 | 155.55 | 221.31 | 277.82 | 359.35 | 456.64 | 490.35 | 485.06 |
| Fiji | 1.02 | 2.55 | 5.22 | 8.85 | 19.21 | 34.34 | 57.67 | 104.22 | 163.45 | 242.13 | 368.52 | 499.86 | 555.10 | 695.42 | 865.57 |
| Finland | 8.50 | 12.88 | 26.43 | 63.95 | 150.90 | 336.09 | 592.88 | 1150.15 | 1473.39 | 1810.91 | 2282.76 | 2658.85 | 2431.15 | 1391.14 | 766.02 |
| France | 2.74 | 4.71 | 9.83 | 16.74 | 30.82 | 57.63 | 111.47 | 196.06 | 269.40 | 377.14 | 469.94 | 550.01 | 676.69 | 731.43 | 813.67 |
| Gabon | 39.26 | 72.66 | 158.43 | 325.29 | 646.69 | 1281.91 | 1954.93 | 2914.69 | 3023.18 | 3491.31 | 3506.42 | 4196.82 | 5055.93 | 5226.45 | 6983.29 |
| Georgia | 3.41 | 5.98 | 11.18 | 17.37 | 27.56 | 43.62 | 74.72 | 107.50 | 160.19 | 215.80 | 276.62 | 375.71 | 447.30 | 554.15 | 718.12 |
| Germany | 3.09 | 5.24 | 8.60 | 13.30 | 21.97 | 35.34 | 53.21 | 85.97 | 134.86 | 184.00 | 273.17 | 391.28 | 546.57 | 956.74 | 1476.22 |
| Ghana | 5.53 | 8.36 | 12.94 | 23.75 | 43.09 | 80.87 | 133.08 | 213.54 | 287.58 | 434.18 | 660.12 | 1027.41 | 1332.72 | 1456.01 | 1850.63 |
| Greece | 5.69 | 6.97 | 12.04 | 23.20 | 43.66 | 86.58 | 141.00 | 224.16 | 329.87 | 394.15 | 543.21 | 822.73 | 896.27 | 1165.26 | 1220.86 |
| Greenland | 1.75 | 3.96 | 7.74 | 15.20 | 31.74 | 54.89 | 98.48 | 143.44 | 227.26 | 367.29 | 550.18 | 663.48 | 467.78 | 420.48 | 402.61 |
| Grenada | 2.40 | 4.22 | 6.78 | 10.39 | 18.69 | 30.90 | 48.29 | 79.02 | 127.76 | 186.91 | 288.22 | 438.20 | 590.91 | 1010.23 | 1613.74 |
| Guam | 10.18 | 13.70 | 21.49 | 42.63 | 76.81 | 127.71 | 201.45 | 336.14 | 447.39 | 543.37 | 847.70 | 1311.91 | 1435.68 | 1846.42 | 1980.38 |
| Guatemala | 3.10 | 5.30 | 8.75 | 13.46 | 21.66 | 36.37 | 60.17 | 102.49 | 165.62 | 245.70 | 391.47 | 578.24 | 702.80 | 986.76 | 1319.25 |
| Guinea | 0.79 | 1.84 | 3.56 | 7.04 | 14.87 | 29.34 | 48.60 | 84.06 | 131.56 | 203.97 | 283.94 | 376.42 | 407.91 | 443.15 | 527.80 |
| Guinea-Bissau | 19.15 | 32.68 | 58.48 | 90.22 | 179.17 | 327.53 | 495.81 | 717.70 | 975.00 | 1303.13 | 1964.64 | 2710.69 | 2930.30 | 3388.46 | 3880.13 |
| Guyana | 6.04 | 11.76 | 22.60 | 48.24 | 73.88 | 129.09 | 181.54 | 288.82 | 305.77 | 445.28 | 430.62 | 468.47 | 411.74 | 389.48 | 509.32 |
| Haiti | 3.82 | 7.02 | 15.17 | 28.97 | 65.49 | 116.00 | 187.96 | 299.08 | 370.87 | 486.27 | 642.03 | 885.83 | 869.65 | 1134.55 | 1849.01 |
| Honduras | 3.88 | 4.82 | 7.95 | 14.21 | 27.17 | 53.42 | 88.66 | 139.98 | 229.87 | 269.60 | 393.84 | 630.86 | 692.12 | 889.08 | 932.89 |
| Hungary | 8.91 | 12.35 | 20.06 | 40.78 | 79.23 | 155.50 | 254.17 | 411.49 | 533.83 | 598.36 | 830.37 | 1212.97 | 1268.30 | 1571.88 | 1334.57 |
| Iceland | 23.94 | 40.18 | 71.58 | 125.43 | 265.54 | 471.02 | 753.51 | 1088.21 | 1392.01 | 1758.59 | 2062.09 | 2300.07 | 2276.70 | 2700.55 | 2849.86 |
| India | 15.98 | 26.66 | 45.72 | 84.57 | 179.58 | 366.50 | 602.25 | 919.64 | 1283.14 | 1650.84 | 1882.55 | 2147.54 | 1865.00 | 1855.10 | 1886.46 |
| Indonesia | 5.40 | 9.48 | 17.52 | 32.60 | 68.54 | 124.80 | 219.25 | 318.13 | 424.97 | 520.46 | 634.48 | 741.64 | 712.35 | 722.72 | 737.82 |
| Iran (Islamic Republic of) | 0.86 | 2.40 | 5.33 | 10.68 | 25.24 | 51.11 | 96.67 | 166.41 | 252.33 | 351.01 | 521.83 | 706.36 | 734.61 | 674.54 | 712.56 |
| Iraq | 4.37 | 7.17 | 11.09 | 16.63 | 26.65 | 38.44 | 63.33 | 92.98 | 135.20 | 188.61 | 268.95 | 364.86 | 465.40 | 585.55 | 575.25 |
| Ireland | 3.95 | 7.46 | 12.02 | 23.94 | 45.53 | 80.16 | 156.99 | 246.77 | 390.46 | 663.61 | 878.75 | 1066.56 | 1191.49 | 1421.03 | 1659.18 |
| Israel | 2.83 | 6.24 | 14.06 | 32.35 | 87.38 | 168.98 | 367.99 | 574.49 | 913.76 | 1204.51 | 1421.88 | 1508.34 | 1523.02 | 1639.00 | 1860.76 |
| Italy | 7.59 | 13.12 | 22.50 | 40.64 | 79.26 | 150.92 | 269.16 | 418.38 | 524.68 | 652.87 | 812.42 | 952.09 | 950.31 | 1040.30 | 1185.81 |
| Jamaica | 38.09 | 55.88 | 91.90 | 159.73 | 306.25 | 512.13 | 774.15 | 1055.45 | 1262.51 | 1495.48 | 1760.48 | 2073.13 | 2248.65 | 2527.56 | 2994.05 |
| Japan | 4.15 | 6.61 | 9.96 | 14.84 | 25.98 | 40.65 | 65.22 | 97.31 | 148.47 | 215.15 | 343.67 | 506.18 | 664.50 | 863.37 | 1029.91 |
| Jordan | 3.38 | 5.77 | 8.93 | 14.45 | 28.22 | 52.43 | 98.50 | 171.44 | 256.11 | 367.94 | 555.23 | 856.98 | 1095.24 | 1623.76 | 2470.00 |
| Kazakhstan | 3.73 | 6.53 | 9.76 | 15.06 | 30.26 | 51.17 | 82.80 | 138.40 | 221.63 | 301.13 | 442.06 | 622.33 | 849.74 | 1272.04 | 1856.78 |
| Kenya | 18.52 | 33.42 | 56.41 | 100.35 | 194.45 | 324.42 | 433.75 | 740.99 | 1430.56 | 1951.84 | 2196.53 | 2430.99 | 2526.45 | 2886.61 | 2530.71 |
| Kiribati | 9.93 | 16.32 | 25.11 | 39.25 | 63.50 | 93.28 | 140.20 | 198.22 | 249.70 | 296.57 | 345.71 | 408.44 | 483.46 | 645.29 | 817.06 |
| Kuwait | 10.95 | 16.60 | 28.01 | 53.57 | 123.78 | 258.10 | 411.77 | 691.18 | 886.25 | 1244.53 | 1419.10 | 1818.13 | 2297.18 | 2479.76 | 2261.32 |
| Kyrgyzstan | 2.64 | 5.24 | 9.30 | 14.21 | 31.37 | 59.94 | 112.71 | 209.09 | 248.04 | 285.83 | 333.95 | 379.04 | 340.47 | 394.78 | 495.00 |
| Lao People's Democratic Republic | 3.78 | 6.13 | 13.74 | 24.23 | 44.80 | 69.51 | 133.38 | 228.59 | 297.63 | 421.28 | 513.01 | 621.88 | 797.73 | 806.26 | 827.17 |
| Latvia | 63.87 | 117.78 | 234.79 | 495.87 | 988.68 | 1641.41 | 2110.92 | 2658.44 | 3216.03 | 3607.24 | 3540.45 | 3719.15 | 3666.82 | 3714.19 | 4579.87 |
| Lebanon | 39.55 | 60.73 | 94.91 | 151.18 | 264.09 | 425.66 | 644.68 | 882.91 | 1136.91 | 1415.93 | 1537.95 | 1818.75 | 1907.75 | 2196.25 | 2125.81 |
| Lesotho | 1.74 | 3.04 | 5.62 | 10.10 | 20.56 | 39.52 | 69.74 | 108.68 | 154.40 | 202.80 | 266.24 | 300.14 | 273.11 | 309.33 | 404.56 |
| Liberia | 1.22 | 2.56 | 5.92 | 13.27 | 28.66 | 64.23 | 112.57 | 204.44 | 306.07 | 409.29 | 507.39 | 642.06 | 624.16 | 658.00 | 732.16 |
| Libya | 1.78 | 4.21 | 10.31 | 18.91 | 35.76 | 58.86 | 96.72 | 146.81 | 234.84 | 309.58 | 333.26 | 388.98 | 402.03 | 464.20 | 539.52 |
| Lithuania | 27.65 | 41.00 | 55.51 | 89.02 | 156.36 | 258.73 | 366.22 | 511.00 | 728.20 | 964.28 | 1240.49 | 1529.14 | 1762.13 | 2121.56 | 2270.34 |
| Luxembourg | 0.85 | 1.49 | 3.37 | 9.20 | 26.81 | 62.96 | 128.48 | 252.27 | 432.57 | 734.22 | 1331.07 | 1957.88 | 1767.04 | 986.92 | 475.72 |
| North Macedonia | 25.05 | 27.21 | 40.46 | 66.17 | 115.17 | 210.13 | 338.06 | 480.34 | 632.41 | 683.82 | 845.31 | 1065.01 | 1090.33 | 1377.09 | 1470.48 |
| Madagascar | 15.90 | 25.83 | 41.50 | 74.65 | 158.38 | 268.55 | 421.33 | 646.55 | 823.35 | 940.38 | 1103.38 | 1236.18 | 1342.02 | 1427.36 | 1287.21 |
| Malawi | 1.58 | 4.12 | 8.31 | 15.04 | 30.93 | 51.10 | 83.90 | 134.36 | 218.26 | 280.69 | 355.00 | 415.47 | 421.00 | 505.92 | 639.40 |
| Malaysia | 3.31 | 5.48 | 8.76 | 13.00 | 22.01 | 35.58 | 59.36 | 93.28 | 135.07 | 188.51 | 301.11 | 433.69 | 554.13 | 800.68 | 967.67 |
| Maldives | 1.68 | 2.75 | 6.27 | 11.07 | 22.25 | 40.13 | 81.31 | 149.06 | 189.96 | 265.42 | 348.46 | 478.57 | 581.49 | 619.87 | 656.99 |
| Mali | 1.27 | 2.12 | 4.25 | 8.64 | 20.22 | 52.40 | 106.35 | 201.86 | 252.13 | 353.98 | 416.33 | 492.17 | 571.41 | 515.65 | 357.38 |
| Malta | 6.98 | 12.78 | 23.46 | 43.91 | 88.42 | 173.80 | 296.72 | 481.56 | 598.99 | 721.46 | 890.84 | 1008.47 | 1033.73 | 1118.73 | 1269.86 |
| Marshall Islands | 9.78 | 16.39 | 24.31 | 43.12 | 93.46 | 187.12 | 364.01 | 574.65 | 775.16 | 1015.08 | 1203.66 | 1384.23 | 1509.40 | 1689.30 | 1770.59 |
| Mauritania | 8.74 | 11.84 | 19.06 | 40.10 | 67.92 | 132.76 | 257.73 | 457.61 | 625.81 | 704.05 | 852.34 | 1062.59 | 1140.01 | 1331.24 | 1206.65 |
| Mauritius | 7.73 | 13.98 | 21.62 | 34.12 | 59.88 | 93.17 | 145.97 | 228.42 | 344.78 | 466.88 | 612.00 | 923.97 | 1095.35 | 1164.50 | 1538.38 |
| Mexico | 167.28 | 268.42 | 469.40 | 865.74 | 1531.10 | 2464.03 | 3364.51 | 4005.46 | 4484.10 | 4423.10 | 4274.09 | 4420.78 | 4510.43 | 4565.84 | 5416.43 |
| Republic of Moldova | 10.61 | 15.59 | 24.31 | 52.27 | 101.93 | 199.94 | 318.74 | 504.68 | 693.23 | 768.21 | 957.17 | 1176.53 | 1399.67 | 1753.95 | 1867.38 |
| Mongolia | 5.79 | 12.24 | 27.30 | 62.48 | 135.79 | 285.55 | 517.25 | 985.45 | 1376.02 | 1715.81 | 2132.68 | 2440.07 | 2129.27 | 2457.86 | 2277.79 |
| Montenegro | 11.92 | 20.57 | 38.01 | 65.98 | 146.56 | 221.48 | 384.53 | 640.18 | 849.09 | 873.28 | 1040.74 | 1436.59 | 1519.57 | 1716.42 | 1847.94 |
| Morocco | 85.45 | 150.28 | 268.90 | 508.51 | 962.16 | 1597.73 | 2226.02 | 2762.67 | 3276.59 | 3504.24 | 3459.84 | 3580.79 | 3596.37 | 3627.62 | 4952.04 |
| Mozambique | 4.92 | 7.71 | 11.73 | 17.52 | 25.40 | 38.92 | 61.74 | 90.71 | 134.66 | 190.84 | 276.61 | 392.25 | 504.25 | 676.36 | 806.31 |
| Myanmar | 1.43 | 2.37 | 4.42 | 8.05 | 15.70 | 27.99 | 52.08 | 77.68 | 103.48 | 121.88 | 156.38 | 197.78 | 183.57 | 198.42 | 190.36 |
| Namibia | 1.41 | 3.22 | 6.78 | 13.54 | 32.95 | 59.57 | 113.84 | 197.09 | 306.48 | 438.58 | 668.86 | 864.64 | 970.60 | 1122.80 | 863.49 |
| Nepal | 19.91 | 32.05 | 52.77 | 92.76 | 189.98 | 330.02 | 511.53 | 703.57 | 876.06 | 1049.45 | 1219.15 | 1425.09 | 1444.71 | 1531.48 | 1773.46 |
| Netherlands | 1.72 | 3.41 | 7.39 | 14.74 | 30.19 | 59.88 | 109.36 | 193.06 | 245.59 | 315.86 | 395.01 | 592.56 | 803.00 | 812.85 | 666.91 |
| New Zealand | 0.93 | 2.30 | 4.81 | 11.14 | 26.49 | 52.85 | 106.88 | 184.75 | 304.37 | 415.17 | 511.24 | 576.90 | 612.17 | 668.85 | 749.67 |
| Nicaragua | 4.88 | 8.41 | 15.96 | 34.66 | 85.02 | 174.90 | 344.10 | 712.39 | 940.69 | 1178.46 | 1539.16 | 1852.98 | 1836.75 | 1599.46 | 910.00 |
| Niger | 41.74 | 73.51 | 149.00 | 307.47 | 568.38 | 1025.35 | 1466.16 | 1882.61 | 1949.21 | 2244.31 | 2218.56 | 2342.96 | 2238.52 | 2211.95 | 2656.61 |
| Nigeria | 3.54 | 6.54 | 11.24 | 20.56 | 40.69 | 71.06 | 121.99 | 238.32 | 299.74 | 385.28 | 542.50 | 733.10 | 856.94 | 1025.17 | 1144.97 |
| Democratic People's Republic of Korea | 1.78 | 2.96 | 3.98 | 6.33 | 12.80 | 22.31 | 39.76 | 65.63 | 104.03 | 146.72 | 232.96 | 371.85 | 558.54 | 914.18 | 1340.14 |
| Northern Mariana Islands | 3.65 | 7.21 | 12.69 | 22.13 | 37.83 | 63.76 | 98.56 | 144.99 | 219.93 | 297.04 | 457.97 | 573.86 | 663.44 | 807.00 | 1037.38 |
| Norway | 6.54 | 10.66 | 20.69 | 35.21 | 76.95 | 144.57 | 247.92 | 375.35 | 489.01 | 554.94 | 643.90 | 750.89 | 715.52 | 819.35 | 1771.79 |
| Oman | 7.05 | 8.28 | 12.64 | 22.36 | 42.22 | 83.72 | 140.78 | 234.33 | 309.94 | 354.57 | 497.12 | 702.78 | 742.14 | 897.95 | 944.25 |
| Pakistan | 6.40 | 8.90 | 13.74 | 26.05 | 47.39 | 97.48 | 161.88 | 276.85 | 375.79 | 458.74 | 656.41 | 940.48 | 1115.04 | 1421.66 | 1502.99 |
| Palestine | 50.79 | 79.17 | 134.40 | 237.97 | 414.77 | 754.76 | 1074.51 | 1451.31 | 1648.82 | 2075.38 | 2302.38 | 2721.47 | 2765.70 | 3002.81 | 3565.10 |
| Panama | 1.02 | 2.91 | 6.19 | 13.07 | 33.63 | 71.82 | 137.40 | 253.55 | 433.74 | 625.95 | 940.99 | 1303.38 | 1453.99 | 1565.43 | 1127.83 |
| Papua New Guinea | 22.28 | 37.87 | 70.55 | 124.21 | 254.02 | 403.35 | 651.18 | 792.83 | 898.11 | 1068.29 | 1254.05 | 1501.22 | 1684.37 | 1909.88 | 2884.23 |
| Paraguay | 4.04 | 6.84 | 10.52 | 15.75 | 26.35 | 39.10 | 55.86 | 81.92 | 124.75 | 183.45 | 271.77 | 387.73 | 532.22 | 742.85 | 981.50 |
| Peru | 11.17 | 17.85 | 29.16 | 53.09 | 118.35 | 239.15 | 406.13 | 647.59 | 1011.21 | 1362.68 | 1651.72 | 1885.38 | 2483.56 | 2173.84 | 2094.08 |
| Philippines | 6.87 | 9.50 | 21.09 | 44.07 | 92.16 | 161.79 | 274.95 | 496.36 | 612.60 | 763.46 | 974.81 | 1132.14 | 1266.94 | 1477.09 | 1760.13 |
| Poland | 34.75 | 62.61 | 115.78 | 215.64 | 366.12 | 612.43 | 875.46 | 1186.71 | 1508.04 | 1836.15 | 1896.13 | 2355.24 | 2498.08 | 2729.25 | 3480.34 |
| Portugal | 16.22 | 24.91 | 41.70 | 69.41 | 137.32 | 259.42 | 458.17 | 721.08 | 987.61 | 1373.47 | 1698.34 | 2300.39 | 2642.64 | 3168.84 | 4172.00 |
| Puerto Rico | 5.94 | 10.12 | 17.18 | 30.96 | 60.35 | 112.02 | 200.91 | 310.23 | 391.61 | 465.27 | 579.50 | 770.22 | 838.17 | 985.19 | 1070.70 |
| Qatar | 26.79 | 47.11 | 85.78 | 162.84 | 269.03 | 455.36 | 663.32 | 889.94 | 946.32 | 1033.34 | 1121.56 | 1164.92 | 1161.08 | 1173.30 | 1301.37 |
| Romania | 3.60 | 6.52 | 10.83 | 22.11 | 53.28 | 114.13 | 225.55 | 448.56 | 558.78 | 732.34 | 915.56 | 1232.99 | 1282.32 | 1394.11 | 1355.15 |
| Russian Federation | 2.06 | 3.78 | 7.66 | 17.64 | 33.38 | 60.86 | 111.67 | 215.30 | 309.38 | 414.55 | 519.85 | 682.09 | 679.41 | 799.72 | 946.26 |
| Rwanda | 1.89 | 4.02 | 8.20 | 18.66 | 41.56 | 87.76 | 160.15 | 275.95 | 355.58 | 459.35 | 611.44 | 753.77 | 769.48 | 800.50 | 687.63 |
| Saint Lucia | 1.74 | 4.50 | 9.10 | 18.35 | 34.83 | 65.23 | 122.85 | 207.29 | 334.93 | 429.80 | 543.85 | 699.18 | 783.77 | 917.90 | 1139.69 |
| Saint Vincent and the Grenadines | 7.18 | 11.50 | 17.23 | 27.00 | 46.59 | 77.53 | 129.12 | 206.41 | 294.17 | 406.02 | 606.91 | 889.85 | 1146.79 | 1646.44 | 2360.63 |
| Samoa | 11.04 | 21.89 | 37.51 | 60.99 | 128.59 | 258.87 | 410.52 | 574.84 | 715.59 | 993.92 | 1175.19 | 1630.86 | 1778.05 | 1922.20 | 1614.97 |
| Sao Tome and Principe | 12.99 | 20.97 | 33.90 | 66.33 | 189.39 | 376.86 | 686.87 | 1102.67 | 1662.75 | 2574.53 | 3208.04 | 3861.24 | 4527.71 | 4972.90 | 4990.84 |
| Saudi Arabia | 11.72 | 18.69 | 31.54 | 49.29 | 81.23 | 126.89 | 187.36 | 260.97 | 337.65 | 423.08 | 608.35 | 825.84 | 938.08 | 1178.63 | 1432.34 |
| Senegal | 1.51 | 3.60 | 6.80 | 13.70 | 25.41 | 42.98 | 71.63 | 123.78 | 196.16 | 270.10 | 319.06 | 385.32 | 356.83 | 358.23 | 366.90 |
| Serbia | 1.43 | 3.40 | 7.07 | 13.26 | 26.37 | 49.32 | 87.50 | 143.78 | 211.43 | 273.55 | 336.39 | 400.66 | 389.19 | 387.97 | 438.75 |
| Seychelles | 0.87 | 1.54 | 3.15 | 8.03 | 16.25 | 30.66 | 65.85 | 125.23 | 218.02 | 343.28 | 465.26 | 688.99 | 581.89 | 635.13 | 667.96 |
| Sierra Leone | 1.08 | 2.00 | 4.54 | 8.46 | 18.31 | 36.97 | 76.32 | 140.54 | 192.17 | 287.81 | 388.10 | 586.96 | 721.14 | 785.97 | 799.24 |
| Singapore | 10.02 | 16.75 | 30.39 | 61.23 | 127.06 | 244.97 | 401.94 | 628.30 | 887.10 | 1187.72 | 1609.10 | 2205.55 | 2253.48 | 2418.90 | 2645.51 |
| Slovakia | 15.01 | 26.53 | 44.10 | 85.23 | 160.77 | 266.85 | 428.90 | 651.59 | 878.75 | 1164.12 | 1492.76 | 1899.01 | 2261.43 | 3518.04 | 4914.27 |
| Slovenia | 16.82 | 25.86 | 49.57 | 97.68 | 189.21 | 325.63 | 530.19 | 742.31 | 869.33 | 1271.08 | 1692.89 | 2087.55 | 2500.16 | 3239.47 | 5283.96 |
| Solomon Islands | 64.91 | 105.95 | 194.82 | 349.75 | 582.71 | 955.32 | 1330.79 | 1695.51 | 2022.67 | 2160.62 | 2175.19 | 2270.02 | 2332.02 | 2467.41 | 3030.43 |
| Somalia | 4.61 | 7.20 | 11.34 | 17.20 | 26.13 | 41.50 | 65.21 | 97.38 | 140.73 | 198.59 | 276.14 | 362.42 | 408.17 | 491.75 | 722.37 |
| South Africa | 3.47 | 4.95 | 8.40 | 14.09 | 27.89 | 52.93 | 90.21 | 136.86 | 191.44 | 251.07 | 366.04 | 549.57 | 634.81 | 778.09 | 1003.89 |
| Republic of Korea | 21.95 | 34.28 | 58.14 | 107.53 | 202.75 | 369.69 | 563.03 | 763.67 | 968.37 | 1182.34 | 1409.37 | 1699.53 | 1947.09 | 2231.21 | 2549.72 |
| South Sudan | 7.74 | 11.27 | 19.24 | 36.76 | 63.89 | 162.25 | 286.95 | 502.11 | 670.33 | 748.32 | 933.62 | 1202.95 | 1310.30 | 1643.01 | 1775.79 |
| Spain | 0.87 | 2.23 | 5.38 | 12.28 | 31.57 | 62.34 | 116.70 | 215.81 | 358.24 | 557.56 | 712.74 | 966.48 | 875.24 | 824.97 | 805.29 |
| Sri Lanka | 4.27 | 6.81 | 15.70 | 33.90 | 63.33 | 129.59 | 232.02 | 360.55 | 504.21 | 782.78 | 1193.31 | 1716.36 | 1819.02 | 1987.10 | 2167.59 |
| Sudan | 6.29 | 8.33 | 13.30 | 25.44 | 46.99 | 92.76 | 159.18 | 267.66 | 367.96 | 403.51 | 565.12 | 821.04 | 858.16 | 1057.98 | 1108.96 |
| Suriname | 7.98 | 14.24 | 24.07 | 39.41 | 64.08 | 94.99 | 135.94 | 187.84 | 237.24 | 272.75 | 313.12 | 363.90 | 403.14 | 478.46 | 621.76 |
| Eswatini | 0.73 | 1.92 | 4.62 | 8.14 | 20.21 | 34.30 | 61.83 | 115.14 | 173.51 | 240.86 | 343.54 | 445.76 | 459.83 | 482.46 | 470.33 |
| Sweden | 0.71 | 2.19 | 4.90 | 10.94 | 20.44 | 39.03 | 65.32 | 103.52 | 164.04 | 221.61 | 353.26 | 475.08 | 544.20 | 626.15 | 769.51 |
| Switzerland | 33.05 | 68.31 | 128.96 | 271.79 | 521.31 | 814.42 | 1091.45 | 1323.37 | 1498.48 | 1598.58 | 1667.46 | 1702.63 | 1844.42 | 2071.69 | 2700.12 |
| Syrian Arab Republic | 1.73 | 3.27 | 7.38 | 14.35 | 32.08 | 63.71 | 140.81 | 246.77 | 302.12 | 426.52 | 543.93 | 686.81 | 806.83 | 644.99 | 338.97 |
| Taiwan (Province of China) | 14.50 | 25.62 | 41.88 | 81.16 | 170.72 | 304.71 | 560.83 | 1062.39 | 1537.24 | 1801.80 | 2127.70 | 2890.80 | 3109.50 | 3310.31 | 2933.79 |
| Tajikistan | 1.95 | 3.39 | 7.63 | 14.12 | 28.38 | 59.49 | 128.83 | 227.42 | 294.22 | 413.35 | 510.32 | 678.50 | 782.52 | 833.68 | 902.71 |
| United Republic of Tanzania | 4.66 | 8.47 | 13.63 | 21.22 | 35.94 | 57.12 | 102.53 | 169.79 | 260.22 | 352.32 | 458.70 | 631.36 | 809.27 | 1169.03 | 1744.93 |
| Thailand | 2.86 | 5.39 | 9.90 | 19.76 | 42.08 | 84.89 | 150.32 | 280.92 | 408.17 | 571.65 | 703.37 | 883.23 | 900.02 | 915.46 | 1170.71 |
| Bahamas | 34.85 | 53.16 | 82.77 | 135.32 | 241.16 | 397.36 | 577.14 | 781.97 | 929.05 | 1002.28 | 1014.65 | 1007.85 | 1024.97 | 1086.28 | 1169.78 |
| Gambia | 34.76 | 58.24 | 96.38 | 158.28 | 283.05 | 462.68 | 680.19 | 880.28 | 994.94 | 1060.05 | 1224.38 | 1451.73 | 1517.86 | 1547.79 | 1361.43 |
| Timor-Leste | 3.17 | 7.21 | 10.59 | 15.37 | 27.81 | 40.88 | 63.13 | 96.15 | 131.33 | 173.83 | 245.10 | 384.12 | 541.21 | 830.44 | 971.51 |
| Togo | 5.17 | 8.74 | 13.44 | 20.76 | 33.53 | 53.79 | 86.66 | 134.23 | 191.54 | 256.51 | 349.53 | 478.14 | 612.72 | 839.35 | 1173.06 |
| Tonga | 15.94 | 23.44 | 34.84 | 58.71 | 116.25 | 205.54 | 321.89 | 488.70 | 623.55 | 767.01 | 959.43 | 1224.21 | 1474.13 | 1552.16 | 1640.42 |
| Trinidad and Tobago | 9.27 | 15.21 | 26.51 | 47.99 | 94.43 | 161.66 | 244.63 | 365.01 | 473.64 | 603.83 | 803.66 | 1037.63 | 1291.75 | 1566.10 | 1388.30 |
| Tunisia | 1.21 | 2.58 | 4.71 | 8.80 | 20.62 | 40.30 | 77.70 | 125.38 | 188.44 | 239.35 | 302.51 | 338.42 | 270.05 | 311.33 | 378.12 |
| Turkey | 2.53 | 5.28 | 10.35 | 18.91 | 37.18 | 70.37 | 125.33 | 223.30 | 327.14 | 476.21 | 764.66 | 1086.46 | 1034.04 | 1161.36 | 1282.70 |
| Turkmenistan | 1.14 | 2.41 | 4.92 | 10.63 | 21.54 | 41.90 | 78.51 | 131.79 | 197.87 | 274.18 | 343.19 | 430.71 | 443.42 | 452.48 | 489.44 |
| Uganda | 1.44 | 2.15 | 3.73 | 8.42 | 16.09 | 35.83 | 89.89 | 154.49 | 237.92 | 283.74 | 420.90 | 683.55 | 744.27 | 928.48 | 1005.72 |
| Ukraine | 42.65 | 70.26 | 119.48 | 204.91 | 370.41 | 621.40 | 876.61 | 1172.98 | 1259.99 | 1428.81 | 1575.74 | 1741.94 | 1795.33 | 1887.70 | 2261.87 |
| United Arab Emirates | 36.70 | 68.79 | 138.41 | 260.61 | 447.79 | 768.58 | 1084.18 | 1417.13 | 1742.47 | 1850.95 | 1849.75 | 2055.43 | 2291.62 | 2688.54 | 3517.49 |
| United Kingdom | 35.29 | 55.11 | 85.12 | 154.65 | 362.43 | 649.88 | 1350.97 | 2091.08 | 2074.34 | 2339.20 | 2420.55 | 2867.93 | 2728.54 | 3021.21 | 3312.85 |
| United States of America | 3.94 | 7.35 | 12.45 | 22.23 | 45.46 | 89.43 | 160.15 | 278.14 | 415.82 | 625.97 | 801.01 | 1142.01 | 1214.08 | 1335.50 | 1363.72 |
| Uruguay | 5.78 | 9.84 | 15.92 | 28.92 | 65.58 | 125.35 | 224.79 | 373.86 | 613.57 | 836.03 | 1134.51 | 1453.72 | 1729.65 | 1860.74 | 1878.96 |
| Uzbekistan | 3.25 | 4.87 | 8.05 | 14.45 | 33.76 | 64.74 | 126.56 | 201.18 | 244.81 | 311.39 | 392.61 | 446.91 | 325.52 | 369.31 | 406.25 |
| Vanuatu | 27.01 | 47.60 | 93.34 | 196.53 | 359.83 | 645.46 | 949.61 | 1258.60 | 1353.29 | 1560.49 | 1599.12 | 1839.14 | 1863.89 | 1915.68 | 2438.33 |
| Venezuela (Bolivarian Republic of) | 1.41 | 2.67 | 5.71 | 11.05 | 21.51 | 44.65 | 89.55 | 161.14 | 219.59 | 335.28 | 459.57 | 657.53 | 788.71 | 878.25 | 892.02 |
| Viet nam | 0.90 | 2.08 | 3.67 | 7.53 | 13.26 | 21.57 | 35.87 | 66.38 | 86.62 | 108.94 | 138.66 | 185.70 | 183.37 | 206.91 | 230.64 |
| United States Virgin Islands | 15.54 | 17.96 | 25.97 | 45.11 | 99.09 | 188.96 | 346.24 | 600.37 | 676.69 | 2017.09 | 2164.50 | 1885.04 | 1758.45 | 1544.30 | 1875.22 |
| Yemen | 24.18 | 34.12 | 45.49 | 59.13 | 86.67 | 123.62 | 171.65 | 227.37 | 286.67 | 358.33 | 439.37 | 544.88 | 642.41 | 844.33 | 1218.77 |
| Zambia | 0.75 | 1.12 | 2.01 | 3.93 | 8.40 | 17.87 | 37.46 | 64.36 | 93.34 | 139.00 | 177.15 | 243.71 | 299.75 | 326.63 | 363.50 |
| Zimbabwe | 3.04 | 7.08 | 13.97 | 28.64 | 53.68 | 99.28 | 160.37 | 248.12 | 337.02 | 482.60 | 613.12 | 827.23 | 894.83 | 1053.35 | 1352.40 |
| Monaco | 6.47 | 12.53 | 25.02 | 46.82 | 80.71 | 137.91 | 264.67 | 421.44 | 528.47 | 784.00 | 965.89 | 1408.65 | 1519.88 | 2019.97 | 3739.70 |
| San Marino | 2.33 | 4.51 | 9.28 | 18.87 | 35.11 | 70.29 | 135.72 | 232.62 | 284.12 | 349.98 | 399.25 | 475.60 | 555.60 | 709.13 | 905.63 |
| Saint Kitts and Nevis | 2.03 | 3.36 | 5.66 | 10.36 | 23.49 | 49.75 | 100.95 | 179.74 | 214.16 | 250.92 | 264.92 | 301.86 | 239.40 | 270.56 | 349.05 |
| Cook Islands | 2.63 | 5.23 | 11.99 | 24.32 | 52.61 | 104.33 | 211.73 | 299.49 | 435.12 | 592.24 | 720.51 | 853.57 | 872.14 | 973.73 | 1401.59 |
| Nauru | 7.54 | 11.19 | 19.35 | 32.77 | 69.32 | 128.79 | 226.48 | 364.99 | 490.77 | 626.52 | 797.65 | 1040.33 | 1054.72 | 1041.20 | 883.53 |
| Niue | 2.01 | 4.46 | 9.31 | 20.03 | 35.48 | 63.73 | 106.83 | 208.08 | 330.18 | 465.06 | 587.86 | 730.94 | 843.49 | 962.16 | 1046.16 |
| Palau | 7.33 | 12.17 | 20.91 | 39.29 | 80.56 | 147.07 | 239.65 | 360.28 | 458.66 | 560.02 | 657.47 | 754.30 | 767.15 | 812.22 | 908.18 |
| Tokelau | 3.91 | 6.30 | 13.98 | 21.53 | 39.64 | 80.12 | 171.45 | 321.67 | 385.25 | 512.13 | 603.73 | 712.10 | 829.90 | 740.71 | 539.11 |
| Tuvalu | 3.31 | 5.76 | 10.26 | 24.13 | 54.39 | 113.49 | 268.89 | 494.24 | 646.64 | 828.55 | 1068.80 | 1391.85 | 1392.37 | 1044.18 | 651.24 |

Supplementary table 4 Death rate of diabetes and kidney diseases due to Low physical activity all ages

| Deaths | 25-29 years | 30-34 years | 35-39 years | 40-44 years | 45-49 years | 50-54 years | 55-59 years | 60-64 years | 65-69 years | 70-74 years | 75-79 years | 80-84 years | 85-89 years | 90-94 years | 95+ years |
| --- | --- | --- | --- | --- | --- | --- | --- | --- | --- | --- | --- | --- | --- | --- | --- |
| Afghanistan | 0.10 | 0.17 | 0.32 | 0.81 | 2.48 | 5.85 | 11.99 | 20.85 | 30.60 | 41.27 | 53.93 | 69.47 | 85.97 | 94.45 | 80.60 |
| Albania | 0.00 | 0.01 | 0.01 | 0.02 | 0.06 | 0.12 | 0.29 | 0.65 | 1.34 | 2.81 | 7.07 | 13.10 | 17.96 | 27.64 | 40.14 |
| Algeria | 0.05 | 0.09 | 0.18 | 0.38 | 0.91 | 1.87 | 3.22 | 6.10 | 9.19 | 14.68 | 26.68 | 44.48 | 155.91 | 178.12 | 129.44 |
| American Samoa | 0.51 | 1.24 | 2.65 | 6.17 | 12.23 | 24.32 | 40.76 | 62.34 | 89.44 | 118.32 | 153.70 | 222.52 | 306.01 | 414.28 | 558.82 |
| Andorra | 0.00 | 0.01 | 0.01 | 0.02 | 0.10 | 0.24 | 0.50 | 1.10 | 2.31 | 5.17 | 11.19 | 24.42 | 45.10 | 75.50 | 115.48 |
| Angola | 0.04 | 0.06 | 0.14 | 0.30 | 0.73 | 1.98 | 3.76 | 7.73 | 12.05 | 22.70 | 35.39 | 60.92 | 87.05 | 110.58 | 121.61 |
| Antigua and Barbuda | 0.06 | 0.09 | 0.17 | 0.40 | 1.22 | 3.01 | 6.41 | 13.14 | 23.40 | 41.94 | 74.30 | 126.95 | 170.84 | 233.03 | 364.67 |
| Argentina | 0.01 | 0.02 | 0.04 | 0.09 | 0.24 | 0.63 | 1.45 | 2.91 | 5.04 | 8.79 | 14.51 | 29.31 | 48.82 | 72.65 | 93.95 |
| Armenia | 0.01 | 0.01 | 0.02 | 0.05 | 0.19 | 0.42 | 1.02 | 1.96 | 3.68 | 7.98 | 16.59 | 24.05 | 21.58 | 32.75 | 51.93 |
| Australia | 0.01 | 0.03 | 0.04 | 0.09 | 0.29 | 0.55 | 1.04 | 1.79 | 3.02 | 5.78 | 11.39 | 24.95 | 48.28 | 89.30 | 143.58 |
| Austria | 0.00 | 0.00 | 0.01 | 0.02 | 0.06 | 0.14 | 0.39 | 1.07 | 2.53 | 5.70 | 13.12 | 28.93 | 66.58 | 116.30 | 190.48 |
| Azerbaijan | 0.01 | 0.01 | 0.02 | 0.05 | 0.20 | 0.55 | 1.43 | 3.29 | 5.99 | 10.69 | 16.53 | 19.87 | 13.43 | 17.89 | 24.14 |
| Bahrain | 0.11 | 0.22 | 0.37 | 0.69 | 1.61 | 3.21 | 5.35 | 9.21 | 15.47 | 26.21 | 41.79 | 76.60 | 92.65 | 140.13 | 168.50 |
| Bangladesh | 0.13 | 0.21 | 0.31 | 0.67 | 1.64 | 3.98 | 10.32 | 21.78 | 44.83 | 106.34 | 224.04 | 406.06 | 554.26 | 678.28 | 766.51 |
| Barbados | 0.03 | 0.04 | 0.08 | 0.22 | 0.61 | 1.56 | 1.50 | 3.78 | 6.05 | 6.54 | 15.29 | 72.93 | 128.51 | 176.89 | 200.87 |
| Belarus | 0.17 | 0.32 | 0.52 | 1.00 | 2.72 | 5.45 | 10.02 | 17.77 | 27.16 | 49.32 | 91.35 | 159.01 | 241.80 | 406.38 | 545.83 |
| Belgium | 0.00 | 0.01 | 0.01 | 0.02 | 0.09 | 0.16 | 0.31 | 0.54 | 1.06 | 1.95 | 3.59 | 6.59 | 9.03 | 15.60 | 20.37 |
| Belize | 0.00 | 0.01 | 0.01 | 0.02 | 0.08 | 0.15 | 0.35 | 0.78 | 1.48 | 3.09 | 7.69 | 17.24 | 34.47 | 64.25 | 107.98 |
| Benin | 0.14 | 0.16 | 0.37 | 0.83 | 1.98 | 5.00 | 8.74 | 17.92 | 22.52 | 40.27 | 58.76 | 93.02 | 117.10 | 159.04 | 220.24 |
| Bermuda | 0.01 | 0.01 | 0.02 | 0.06 | 0.18 | 0.50 | 1.31 | 2.64 | 5.26 | 7.67 | 16.49 | 35.58 | 50.42 | 83.58 | 102.52 |
| Bhutan | 0.03 | 0.04 | 0.07 | 0.16 | 0.45 | 1.04 | 2.03 | 3.77 | 6.86 | 11.53 | 21.15 | 42.92 | 67.27 | 110.25 | 147.25 |
| Bolivia (Plurinational State of) | 0.09 | 0.16 | 0.23 | 0.56 | 1.54 | 3.46 | 6.41 | 13.11 | 23.01 | 37.41 | 61.12 | 86.85 | 138.87 | 196.85 | 239.08 |
| Bosnia and Herzegovina | 0.03 | 0.05 | 0.12 | 0.35 | 0.90 | 2.15 | 4.65 | 10.55 | 17.09 | 29.58 | 52.21 | 83.62 | 104.51 | 135.83 | 121.83 |
| Botswana | 0.01 | 0.01 | 0.02 | 0.04 | 0.18 | 0.53 | 1.67 | 4.36 | 8.61 | 18.46 | 41.23 | 63.67 | 62.25 | 64.48 | 49.29 |
| Brazil | 0.02 | 0.04 | 0.10 | 0.32 | 1.19 | 3.32 | 8.81 | 22.08 | 35.52 | 56.47 | 89.64 | 133.14 | 162.22 | 180.34 | 112.67 |
| Brunei Darussalam | 0.04 | 0.09 | 0.18 | 0.38 | 1.00 | 2.16 | 4.14 | 8.04 | 14.70 | 25.05 | 42.66 | 69.24 | 102.90 | 143.37 | 176.14 |
| Bulgaria | 0.03 | 0.13 | 0.33 | 0.60 | 1.81 | 3.63 | 5.65 | 11.09 | 16.59 | 29.93 | 66.86 | 105.16 | 145.89 | 213.32 | 497.32 |
| Burkina Faso | 0.02 | 0.03 | 0.06 | 0.13 | 0.34 | 0.75 | 1.53 | 2.75 | 4.78 | 8.44 | 15.54 | 27.14 | 35.50 | 56.46 | 90.72 |
| Burundi | 0.02 | 0.03 | 0.05 | 0.13 | 0.32 | 0.87 | 2.52 | 5.12 | 8.01 | 9.42 | 18.48 | 38.03 | 61.81 | 98.20 | 116.88 |
| Cambodia | 0.01 | 0.03 | 0.06 | 0.14 | 0.38 | 0.88 | 2.24 | 4.75 | 7.54 | 12.97 | 21.60 | 39.38 | 60.24 | 78.54 | 96.03 |
| Cameroon | 0.03 | 0.03 | 0.07 | 0.16 | 0.39 | 0.92 | 1.72 | 3.21 | 5.92 | 10.01 | 21.56 | 49.65 | 68.66 | 104.61 | 110.16 |
| Canada | 0.00 | 0.01 | 0.02 | 0.06 | 0.18 | 0.45 | 0.97 | 1.96 | 4.08 | 7.37 | 14.52 | 27.67 | 37.13 | 46.70 | 55.22 |
| Cabo Verde | 0.08 | 0.10 | 0.16 | 0.43 | 0.98 | 2.50 | 4.91 | 9.81 | 16.49 | 20.94 | 38.70 | 75.87 | 107.12 | 171.44 | 196.81 |
| Central African Republic | 0.00 | 0.01 | 0.01 | 0.02 | 0.09 | 0.17 | 0.31 | 0.55 | 1.02 | 2.13 | 4.88 | 12.12 | 22.79 | 43.34 | 90.06 |
| Chad | 0.10 | 0.14 | 0.25 | 0.56 | 1.31 | 2.91 | 5.76 | 10.09 | 13.37 | 22.29 | 33.33 | 53.71 | 82.33 | 96.86 | 88.68 |
| Chile | 0.03 | 0.04 | 0.08 | 0.22 | 0.59 | 1.55 | 2.99 | 6.31 | 10.43 | 13.34 | 22.25 | 38.21 | 53.26 | 82.10 | 95.16 |
| China | 0.01 | 0.02 | 0.03 | 0.06 | 0.15 | 0.38 | 0.82 | 1.76 | 2.79 | 5.03 | 9.56 | 19.77 | 32.38 | 62.87 | 126.97 |
| Colombia | 0.01 | 0.02 | 0.03 | 0.07 | 0.16 | 0.36 | 0.83 | 1.82 | 3.78 | 8.09 | 14.34 | 24.56 | 40.62 | 58.31 | 66.54 |
| Comoros | 0.05 | 0.09 | 0.16 | 0.31 | 0.70 | 1.32 | 2.65 | 4.61 | 7.22 | 10.11 | 14.50 | 23.12 | 31.35 | 39.00 | 34.14 |
| Congo | 0.01 | 0.02 | 0.07 | 0.15 | 0.36 | 0.88 | 2.20 | 4.79 | 7.60 | 13.28 | 19.95 | 29.91 | 47.36 | 62.68 | 77.41 |
| Costa Rica | 0.08 | 0.12 | 0.24 | 0.60 | 1.27 | 3.30 | 5.84 | 11.11 | 13.09 | 20.73 | 30.69 | 54.64 | 87.07 | 117.31 | 149.16 |
| Côte d'Ivoire | 0.13 | 0.33 | 0.86 | 2.19 | 4.89 | 10.44 | 17.64 | 29.60 | 39.11 | 54.65 | 83.89 | 136.42 | 202.91 | 284.64 | 387.40 |
| Croatia | 0.03 | 0.04 | 0.09 | 0.19 | 0.49 | 1.01 | 2.18 | 3.74 | 6.51 | 9.52 | 17.77 | 33.99 | 47.79 | 66.03 | 62.21 |
| Cuba | 0.01 | 0.01 | 0.02 | 0.04 | 0.14 | 0.29 | 0.68 | 1.53 | 3.23 | 8.13 | 22.19 | 44.12 | 66.56 | 85.49 | 107.54 |
| Cyprus | 0.03 | 0.04 | 0.08 | 0.16 | 0.42 | 0.85 | 1.80 | 3.64 | 6.43 | 9.52 | 15.92 | 25.66 | 33.49 | 50.10 | 63.29 |
| Czechia | 0.01 | 0.01 | 0.02 | 0.04 | 0.19 | 0.41 | 1.03 | 2.43 | 5.32 | 13.14 | 25.55 | 69.88 | 132.37 | 401.45 | 798.34 |
| Democratic Republic of the Congo | 0.01 | 0.01 | 0.02 | 0.04 | 0.14 | 0.33 | 0.76 | 1.91 | 5.15 | 12.18 | 22.77 | 44.87 | 82.45 | 101.73 | 104.51 |
| Denmark | 0.06 | 0.07 | 0.11 | 0.30 | 0.68 | 1.82 | 3.75 | 7.51 | 12.71 | 15.84 | 29.38 | 52.04 | 74.05 | 117.23 | 140.65 |
| Djibouti | 0.02 | 0.04 | 0.08 | 0.19 | 0.50 | 1.01 | 2.09 | 3.98 | 6.56 | 11.21 | 19.05 | 26.51 | 36.04 | 46.65 | 60.78 |
| Dominica | 0.09 | 0.12 | 0.19 | 0.38 | 0.97 | 2.43 | 4.67 | 9.66 | 21.44 | 36.94 | 54.29 | 86.87 | 135.05 | 169.65 | 206.29 |
| Dominican Republic | 0.00 | 0.00 | 0.00 | 0.01 | 0.09 | 0.21 | 0.55 | 1.21 | 2.49 | 5.11 | 11.93 | 29.24 | 62.04 | 105.79 | 149.90 |
| Ecuador | 0.01 | 0.02 | 0.07 | 0.16 | 0.42 | 0.98 | 2.41 | 5.18 | 7.96 | 14.60 | 24.51 | 45.07 | 73.64 | 97.45 | 119.48 |
| Egypt | 0.10 | 0.14 | 0.25 | 0.49 | 1.19 | 2.51 | 4.64 | 8.47 | 17.34 | 38.47 | 77.31 | 154.97 | 209.71 | 273.53 | 336.12 |
| El Salvador | 0.07 | 0.12 | 0.24 | 0.55 | 1.29 | 2.72 | 5.34 | 9.93 | 14.28 | 19.18 | 28.42 | 44.02 | 60.21 | 83.62 | 158.42 |
| Equatorial Guinea | 0.03 | 0.06 | 0.12 | 0.32 | 0.77 | 1.74 | 3.57 | 8.31 | 11.59 | 15.45 | 21.69 | 31.28 | 53.35 | 96.49 | 211.47 |
| Eritrea | 0.08 | 0.15 | 0.27 | 0.56 | 1.47 | 3.69 | 7.48 | 14.47 | 27.24 | 49.41 | 80.19 | 151.73 | 197.85 | 116.15 | 90.35 |
| Estonia | 0.10 | 0.16 | 0.32 | 0.68 | 1.53 | 3.42 | 6.86 | 11.69 | 17.23 | 24.68 | 36.41 | 58.56 | 73.55 | 105.11 | 98.26 |
| Ethiopia | 0.12 | 0.17 | 0.28 | 0.59 | 1.16 | 2.96 | 5.46 | 10.65 | 17.24 | 30.44 | 52.29 | 93.29 | 146.72 | 189.70 | 234.25 |
| Micronesia (Federated States of) | 0.01 | 0.02 | 0.04 | 0.11 | 0.29 | 0.65 | 1.69 | 3.26 | 5.08 | 8.89 | 14.12 | 23.70 | 40.07 | 51.44 | 55.13 |
| Fiji | 0.01 | 0.01 | 0.01 | 0.03 | 0.13 | 0.28 | 0.61 | 1.44 | 3.00 | 5.88 | 11.42 | 21.32 | 31.92 | 51.05 | 72.29 |
| Finland | 0.07 | 0.12 | 0.29 | 0.95 | 2.81 | 7.26 | 14.41 | 33.13 | 50.01 | 74.46 | 118.87 | 176.70 | 205.02 | 124.61 | 63.21 |
| France | 0.02 | 0.04 | 0.10 | 0.19 | 0.45 | 1.06 | 2.52 | 5.27 | 8.54 | 14.92 | 23.41 | 35.84 | 58.58 | 75.86 | 92.60 |
| Gabon | 0.34 | 0.76 | 2.03 | 4.91 | 11.43 | 27.02 | 47.75 | 84.75 | 104.70 | 148.13 | 185.21 | 289.69 | 458.43 | 557.57 | 806.56 |
| Georgia | 0.00 | 0.00 | 0.00 | 0.01 | 0.05 | 0.12 | 0.28 | 0.59 | 1.16 | 2.35 | 4.29 | 10.14 | 20.30 | 34.55 | 56.56 |
| Germany | 0.00 | 0.01 | 0.01 | 0.03 | 0.09 | 0.22 | 0.43 | 0.98 | 2.07 | 3.94 | 8.54 | 18.52 | 39.33 | 92.15 | 164.96 |
| Ghana | 0.06 | 0.09 | 0.16 | 0.33 | 0.69 | 1.55 | 3.01 | 5.70 | 8.97 | 16.75 | 32.61 | 67.18 | 116.06 | 149.66 | 205.96 |
| Greece | 0.05 | 0.05 | 0.11 | 0.27 | 0.62 | 1.56 | 2.88 | 5.45 | 9.72 | 13.84 | 25.07 | 51.14 | 72.44 | 115.95 | 130.23 |
| Greenland | 0.01 | 0.01 | 0.03 | 0.07 | 0.25 | 0.51 | 1.23 | 2.28 | 4.81 | 10.62 | 21.31 | 31.97 | 20.89 | 18.04 | 16.69 |
| Grenada | 0.00 | 0.01 | 0.01 | 0.02 | 0.09 | 0.19 | 0.39 | 0.87 | 1.89 | 3.78 | 9.06 | 20.57 | 41.41 | 95.87 | 176.08 |
| Guam | 0.09 | 0.12 | 0.20 | 0.53 | 1.14 | 2.36 | 4.25 | 8.35 | 13.54 | 19.75 | 40.19 | 83.81 | 119.28 | 187.51 | 216.38 |
| Guatemala | 0.00 | 0.01 | 0.01 | 0.02 | 0.07 | 0.18 | 0.44 | 1.06 | 2.43 | 5.44 | 12.57 | 27.66 | 47.26 | 85.28 | 131.04 |
| Guinea | 0.00 | 0.01 | 0.02 | 0.05 | 0.14 | 0.34 | 0.64 | 1.33 | 2.47 | 4.87 | 9.17 | 16.45 | 24.81 | 33.30 | 46.51 |
| Guinea-Bissau | 0.16 | 0.28 | 0.56 | 0.91 | 2.20 | 4.82 | 8.77 | 15.75 | 26.67 | 45.82 | 93.09 | 166.36 | 234.53 | 331.66 | 418.80 |
| Guyana | 0.02 | 0.05 | 0.19 | 0.43 | 0.92 | 1.98 | 3.11 | 5.69 | 7.38 | 11.88 | 13.14 | 16.26 | 15.26 | 16.29 | 36.81 |
| Haiti | 0.04 | 0.07 | 0.17 | 0.37 | 1.01 | 2.03 | 3.96 | 7.35 | 10.82 | 17.38 | 28.80 | 51.05 | 65.00 | 107.70 | 201.41 |
| Honduras | 0.04 | 0.04 | 0.08 | 0.17 | 0.40 | 0.98 | 1.88 | 3.54 | 7.09 | 9.87 | 18.64 | 39.84 | 56.71 | 88.21 | 99.24 |
| Hungary | 0.08 | 0.11 | 0.19 | 0.51 | 1.23 | 2.93 | 5.44 | 10.27 | 16.10 | 21.51 | 38.73 | 75.98 | 103.23 | 156.82 | 138.62 |
| Iceland | 0.19 | 0.31 | 0.59 | 1.12 | 3.18 | 6.86 | 13.36 | 23.86 | 37.45 | 59.64 | 90.38 | 126.78 | 161.92 | 243.39 | 283.85 |
| India | 0.13 | 0.21 | 0.35 | 0.78 | 2.24 | 5.75 | 11.55 | 21.89 | 37.99 | 61.29 | 88.75 | 129.39 | 138.26 | 164.06 | 179.89 |
| Indonesia | 0.02 | 0.04 | 0.08 | 0.21 | 0.62 | 1.51 | 3.39 | 5.96 | 9.88 | 15.52 | 24.50 | 35.64 | 43.04 | 52.64 | 58.26 |
| Iran (Islamic Republic of) | 0.01 | 0.01 | 0.02 | 0.04 | 0.13 | 0.35 | 0.90 | 1.93 | 3.76 | 7.19 | 15.64 | 30.58 | 42.58 | 43.20 | 51.10 |
| Iraq | 0.00 | 0.01 | 0.01 | 0.01 | 0.05 | 0.07 | 0.24 | 0.49 | 1.12 | 2.49 | 6.06 | 12.84 | 26.29 | 42.74 | 45.55 |
| Ireland | 0.02 | 0.03 | 0.05 | 0.17 | 0.48 | 1.09 | 2.83 | 5.51 | 10.92 | 23.21 | 40.33 | 62.77 | 92.09 | 134.19 | 172.59 |
| Israel | 0.02 | 0.05 | 0.13 | 0.36 | 1.16 | 2.77 | 7.15 | 13.11 | 24.23 | 38.02 | 57.02 | 78.01 | 103.33 | 139.07 | 180.24 |
| Italy | 0.03 | 0.05 | 0.09 | 0.19 | 0.52 | 1.29 | 2.95 | 6.07 | 9.83 | 16.36 | 29.53 | 47.04 | 61.37 | 84.23 | 110.15 |
| Jamaica | 0.11 | 0.19 | 0.41 | 0.96 | 2.62 | 5.50 | 10.60 | 18.11 | 27.41 | 42.83 | 67.80 | 106.55 | 155.43 | 215.62 | 287.26 |
| Japan | 0.00 | 0.01 | 0.01 | 0.01 | 0.06 | 0.12 | 0.28 | 0.56 | 1.17 | 2.91 | 8.26 | 20.37 | 41.06 | 69.01 | 95.20 |
| Jordan | 0.01 | 0.01 | 0.02 | 0.05 | 0.16 | 0.41 | 0.97 | 2.25 | 4.32 | 8.75 | 19.97 | 47.55 | 85.08 | 160.15 | 280.97 |
| Kazakhstan | 0.00 | 0.01 | 0.01 | 0.03 | 0.11 | 0.26 | 0.57 | 1.33 | 3.03 | 6.17 | 14.18 | 30.11 | 61.61 | 119.17 | 200.56 |
| Kenya | 0.14 | 0.26 | 0.45 | 0.94 | 2.45 | 4.96 | 7.83 | 16.57 | 39.74 | 69.69 | 102.90 | 151.45 | 205.36 | 285.84 | 279.87 |
| Kiribati | 0.01 | 0.01 | 0.02 | 0.04 | 0.13 | 0.26 | 0.47 | 0.84 | 1.47 | 2.59 | 4.86 | 9.75 | 20.72 | 42.82 | 70.80 |
| Kuwait | 0.05 | 0.08 | 0.14 | 0.32 | 0.93 | 2.31 | 4.49 | 10.22 | 17.29 | 35.42 | 51.71 | 90.48 | 166.07 | 219.24 | 213.59 |
| Kyrgyzstan | 0.01 | 0.01 | 0.03 | 0.05 | 0.15 | 0.37 | 0.90 | 2.31 | 3.58 | 5.47 | 8.77 | 12.09 | 11.05 | 18.42 | 30.29 |
| Lao People's Democratic Republic | 0.03 | 0.06 | 0.16 | 0.33 | 0.76 | 1.43 | 3.32 | 6.68 | 10.13 | 17.45 | 26.39 | 41.47 | 70.45 | 84.16 | 93.89 |
| Latvia | 0.52 | 1.18 | 2.84 | 7.35 | 17.18 | 33.68 | 50.32 | 74.60 | 109.59 | 152.43 | 188.76 | 256.19 | 324.03 | 387.99 | 519.53 |
| Lebanon | 0.06 | 0.10 | 0.17 | 0.33 | 0.81 | 1.80 | 3.66 | 7.02 | 14.00 | 28.82 | 42.44 | 75.97 | 109.53 | 161.27 | 174.06 |
| Lesotho | 0.01 | 0.01 | 0.03 | 0.05 | 0.15 | 0.37 | 0.89 | 1.75 | 3.11 | 4.64 | 7.24 | 8.08 | 6.61 | 9.95 | 20.53 |
| Liberia | 0.01 | 0.02 | 0.06 | 0.16 | 0.41 | 1.12 | 2.31 | 4.85 | 8.26 | 12.89 | 20.03 | 32.04 | 39.24 | 50.82 | 64.32 |
| Libya | 0.01 | 0.02 | 0.06 | 0.11 | 0.32 | 0.61 | 1.27 | 2.31 | 4.62 | 8.04 | 11.20 | 17.31 | 22.73 | 31.73 | 40.14 |
| Lithuania | 0.07 | 0.11 | 0.19 | 0.40 | 1.06 | 2.22 | 3.55 | 6.15 | 12.72 | 23.49 | 42.59 | 70.90 | 115.74 | 177.99 | 210.62 |
| Luxembourg | 0.01 | 0.01 | 0.03 | 0.13 | 0.48 | 1.33 | 3.13 | 7.33 | 15.00 | 30.93 | 70.51 | 131.45 | 150.11 | 88.66 | 38.47 |
| North Macedonia | 0.25 | 0.23 | 0.38 | 0.77 | 1.65 | 3.77 | 7.05 | 11.74 | 18.84 | 23.94 | 38.86 | 66.00 | 87.93 | 136.44 | 157.41 |
| Madagascar | 0.07 | 0.13 | 0.23 | 0.51 | 1.44 | 2.81 | 5.16 | 10.78 | 17.06 | 23.97 | 36.93 | 52.66 | 79.33 | 103.70 | 96.51 |
| Malawi | 0.01 | 0.02 | 0.03 | 0.06 | 0.25 | 0.48 | 0.96 | 1.96 | 4.15 | 6.62 | 10.81 | 15.99 | 19.75 | 30.01 | 43.91 |
| Malaysia | 0.00 | 0.00 | 0.00 | 0.01 | 0.05 | 0.13 | 0.32 | 0.71 | 1.56 | 3.17 | 7.98 | 17.87 | 35.19 | 68.47 | 94.88 |
| Maldives | 0.01 | 0.03 | 0.07 | 0.15 | 0.36 | 0.78 | 1.92 | 4.15 | 6.15 | 10.53 | 17.25 | 30.52 | 49.45 | 62.99 | 73.28 |
| Mali | 0.01 | 0.03 | 0.06 | 0.14 | 0.39 | 1.21 | 2.84 | 6.29 | 9.06 | 15.27 | 22.04 | 33.31 | 50.61 | 53.67 | 39.67 |
| Malta | 0.03 | 0.08 | 0.17 | 0.40 | 1.01 | 2.41 | 4.83 | 8.87 | 11.62 | 15.64 | 25.50 | 38.24 | 51.77 | 72.70 | 98.26 |
| Marshall Islands | 0.04 | 0.09 | 0.16 | 0.32 | 0.85 | 2.20 | 4.99 | 9.22 | 13.97 | 23.15 | 36.41 | 58.27 | 88.24 | 127.92 | 154.98 |
| Mauritania | 0.05 | 0.06 | 0.12 | 0.34 | 0.76 | 1.95 | 4.28 | 8.97 | 15.51 | 20.57 | 33.72 | 57.97 | 81.70 | 117.32 | 110.73 |
| Mauritius | 0.00 | 0.02 | 0.03 | 0.08 | 0.29 | 0.49 | 1.12 | 2.48 | 4.94 | 10.15 | 19.09 | 46.85 | 76.88 | 96.48 | 155.65 |
| Mexico | 0.95 | 2.00 | 4.55 | 10.75 | 23.05 | 45.38 | 74.61 | 105.15 | 144.20 | 174.72 | 211.98 | 287.02 | 378.24 | 450.98 | 585.69 |
| Republic of Moldova | 0.08 | 0.12 | 0.20 | 0.60 | 1.48 | 3.62 | 6.73 | 12.76 | 21.56 | 28.55 | 46.60 | 76.89 | 121.07 | 182.31 | 210.58 |
| Mongolia | 0.06 | 0.14 | 0.34 | 0.91 | 2.31 | 5.59 | 11.50 | 25.26 | 42.49 | 63.36 | 100.44 | 145.50 | 155.98 | 223.41 | 226.60 |
| Montenegro | 0.11 | 0.18 | 0.37 | 0.75 | 2.06 | 3.73 | 7.76 | 15.36 | 24.78 | 31.60 | 48.83 | 88.58 | 123.07 | 167.97 | 198.44 |
| Morocco | 0.49 | 1.21 | 2.83 | 6.80 | 15.20 | 30.13 | 49.12 | 71.69 | 103.17 | 135.63 | 168.94 | 231.94 | 303.74 | 366.32 | 558.52 |
| Mozambique | 0.01 | 0.01 | 0.02 | 0.03 | 0.07 | 0.13 | 0.29 | 0.62 | 1.36 | 3.03 | 7.03 | 15.74 | 30.69 | 53.16 | 71.91 |
| Myanmar | 0.01 | 0.01 | 0.02 | 0.05 | 0.14 | 0.29 | 0.70 | 1.37 | 2.24 | 3.09 | 5.00 | 7.28 | 7.18 | 8.76 | 8.38 |
| Namibia | 0.01 | 0.02 | 0.03 | 0.06 | 0.22 | 0.43 | 1.14 | 2.45 | 5.07 | 9.98 | 23.42 | 41.89 | 64.04 | 91.81 | 67.45 |
| Nepal | 0.05 | 0.09 | 0.19 | 0.42 | 1.29 | 2.88 | 5.61 | 9.80 | 15.40 | 23.58 | 36.71 | 55.88 | 75.23 | 100.67 | 138.02 |
| Netherlands | 0.01 | 0.03 | 0.09 | 0.21 | 0.53 | 1.26 | 2.70 | 5.59 | 8.12 | 12.42 | 19.10 | 38.12 | 70.02 | 84.57 | 74.50 |
| New Zealand | 0.01 | 0.02 | 0.06 | 0.15 | 0.40 | 0.91 | 2.12 | 4.29 | 8.10 | 13.08 | 20.66 | 30.51 | 42.65 | 56.93 | 71.08 |
| Nicaragua | 0.03 | 0.06 | 0.15 | 0.43 | 1.38 | 3.47 | 7.97 | 20.05 | 31.80 | 48.83 | 81.20 | 124.71 | 157.54 | 157.05 | 85.18 |
| Niger | 0.28 | 0.64 | 1.68 | 4.32 | 9.38 | 20.15 | 33.76 | 51.03 | 64.18 | 90.44 | 112.45 | 152.63 | 188.21 | 222.08 | 296.36 |
| Nigeria | 0.01 | 0.03 | 0.05 | 0.13 | 0.40 | 0.95 | 1.96 | 4.91 | 7.57 | 12.26 | 23.28 | 40.89 | 64.31 | 94.14 | 111.76 |
| Democratic People's Republic of Korea | 0.00 | 0.00 | 0.00 | 0.01 | 0.04 | 0.11 | 0.26 | 0.61 | 1.27 | 2.57 | 6.62 | 16.83 | 40.53 | 87.71 | 147.26 |
| Northern Mariana Islands | 0.01 | 0.03 | 0.05 | 0.12 | 0.26 | 0.56 | 0.96 | 1.59 | 2.76 | 5.04 | 11.97 | 22.49 | 39.18 | 62.39 | 96.97 |
| Norway | 0.05 | 0.08 | 0.17 | 0.32 | 0.84 | 1.95 | 4.13 | 7.64 | 11.96 | 16.43 | 24.11 | 34.95 | 42.63 | 64.14 | 185.27 |
| Oman | 0.06 | 0.06 | 0.09 | 0.20 | 0.48 | 1.27 | 2.45 | 4.90 | 8.04 | 10.81 | 20.53 | 39.63 | 54.36 | 82.03 | 93.49 |
| Pakistan | 0.06 | 0.08 | 0.12 | 0.29 | 0.64 | 1.67 | 3.20 | 6.62 | 10.87 | 15.96 | 30.48 | 58.00 | 91.50 | 142.28 | 163.59 |
| Palestine | 0.32 | 0.60 | 1.17 | 2.51 | 5.36 | 12.37 | 21.48 | 35.26 | 50.77 | 81.32 | 115.58 | 178.88 | 234.71 | 302.11 | 394.71 |
| Panama | 0.01 | 0.02 | 0.03 | 0.06 | 0.22 | 0.58 | 1.39 | 3.48 | 8.36 | 16.73 | 37.35 | 72.95 | 108.70 | 139.23 | 94.96 |
| Papua New Guinea | 0.09 | 0.23 | 0.61 | 1.43 | 3.44 | 7.14 | 13.30 | 19.55 | 27.35 | 39.94 | 62.03 | 95.32 | 142.41 | 192.07 | 323.45 |
| Paraguay | 0.00 | 0.01 | 0.01 | 0.02 | 0.08 | 0.15 | 0.28 | 0.55 | 1.18 | 2.73 | 6.74 | 15.29 | 33.93 | 61.69 | 95.05 |
| Peru | 0.06 | 0.10 | 0.17 | 0.37 | 1.14 | 3.03 | 6.30 | 12.81 | 26.73 | 46.47 | 73.51 | 107.97 | 202.39 | 202.81 | 213.70 |
| Philippines | 0.05 | 0.07 | 0.15 | 0.39 | 1.08 | 2.41 | 4.89 | 11.27 | 16.89 | 26.30 | 44.78 | 67.49 | 100.51 | 144.02 | 189.39 |
| Poland | 0.18 | 0.46 | 1.03 | 2.34 | 4.61 | 9.44 | 16.03 | 26.76 | 46.03 | 70.64 | 92.19 | 154.39 | 216.17 | 281.57 | 393.37 |
| Portugal | 0.10 | 0.15 | 0.28 | 0.48 | 1.21 | 3.00 | 6.73 | 13.55 | 23.69 | 45.08 | 73.97 | 138.24 | 213.92 | 313.41 | 456.21 |
| Puerto Rico | 0.04 | 0.07 | 0.12 | 0.25 | 0.57 | 1.36 | 3.09 | 5.92 | 9.09 | 13.66 | 21.96 | 39.14 | 57.41 | 84.61 | 106.83 |
| Qatar | 0.17 | 0.37 | 0.84 | 1.97 | 3.78 | 7.92 | 13.75 | 22.13 | 28.98 | 38.99 | 52.96 | 70.98 | 92.11 | 111.11 | 134.24 |
| Romania | 0.03 | 0.06 | 0.11 | 0.25 | 0.71 | 1.84 | 4.38 | 10.56 | 16.14 | 27.06 | 43.32 | 76.41 | 103.14 | 133.58 | 140.14 |
| Russian Federation | 0.02 | 0.03 | 0.07 | 0.20 | 0.47 | 1.06 | 2.29 | 5.16 | 8.68 | 13.99 | 21.60 | 37.12 | 47.13 | 69.32 | 92.92 |
| Rwanda | 0.02 | 0.04 | 0.10 | 0.27 | 0.72 | 1.75 | 3.66 | 7.20 | 10.59 | 15.93 | 26.41 | 41.52 | 55.23 | 71.12 | 66.34 |
| Saint Lucia | 0.01 | 0.02 | 0.03 | 0.07 | 0.20 | 0.45 | 1.00 | 2.11 | 4.59 | 8.20 | 15.31 | 28.53 | 44.95 | 66.16 | 94.01 |
| Saint Vincent and the Grenadines | 0.01 | 0.02 | 0.03 | 0.05 | 0.15 | 0.34 | 0.76 | 1.75 | 3.35 | 7.18 | 18.20 | 41.90 | 79.80 | 149.74 | 247.95 |
| Samoa | 0.08 | 0.16 | 0.27 | 0.46 | 1.25 | 3.01 | 5.65 | 9.49 | 14.85 | 27.90 | 45.12 | 84.42 | 123.97 | 163.48 | 157.63 |
| Sao Tome and Principe | 0.05 | 0.09 | 0.14 | 0.33 | 1.19 | 2.89 | 7.15 | 15.69 | 36.00 | 83.93 | 141.29 | 227.18 | 360.32 | 474.23 | 527.37 |
| Saudi Arabia | 0.01 | 0.02 | 0.04 | 0.09 | 0.24 | 0.48 | 0.90 | 1.60 | 2.86 | 5.64 | 14.79 | 31.95 | 53.38 | 91.83 | 135.15 |
| Senegal | 0.01 | 0.01 | 0.02 | 0.05 | 0.16 | 0.32 | 0.61 | 1.48 | 3.19 | 5.73 | 8.01 | 11.33 | 10.75 | 10.97 | 10.53 |
| Serbia | 0.01 | 0.02 | 0.03 | 0.06 | 0.15 | 0.35 | 0.83 | 1.84 | 3.38 | 5.78 | 9.96 | 15.32 | 17.45 | 18.26 | 23.36 |
| Seychelles | 0.00 | 0.01 | 0.01 | 0.03 | 0.11 | 0.26 | 0.79 | 2.04 | 4.97 | 10.42 | 19.15 | 37.07 | 35.56 | 46.04 | 51.11 |
| Sierra Leone | 0.01 | 0.02 | 0.06 | 0.12 | 0.32 | 0.77 | 1.90 | 4.10 | 6.48 | 11.75 | 19.81 | 39.13 | 63.34 | 82.82 | 91.68 |
| Singapore | 0.06 | 0.09 | 0.19 | 0.49 | 1.27 | 3.15 | 6.18 | 11.98 | 22.13 | 38.40 | 69.93 | 130.90 | 172.64 | 223.47 | 270.41 |
| Slovakia | 0.11 | 0.18 | 0.31 | 0.71 | 1.67 | 3.21 | 6.12 | 11.73 | 19.96 | 34.99 | 59.91 | 102.25 | 168.70 | 344.09 | 539.14 |
| Slovenia | 0.14 | 0.18 | 0.43 | 1.03 | 2.38 | 4.56 | 9.21 | 14.82 | 19.97 | 40.07 | 72.81 | 118.68 | 194.28 | 316.29 | 585.61 |
| Solomon Islands | 0.27 | 0.62 | 1.62 | 3.86 | 7.85 | 16.20 | 27.02 | 41.10 | 61.00 | 80.55 | 103.44 | 140.94 | 188.23 | 238.43 | 326.17 |
| Somalia | 0.00 | 0.00 | 0.01 | 0.01 | 0.05 | 0.10 | 0.23 | 0.52 | 1.10 | 2.52 | 5.70 | 11.69 | 19.14 | 30.42 | 64.80 |
| South Africa | 0.01 | 0.02 | 0.04 | 0.10 | 0.30 | 0.73 | 1.50 | 2.62 | 4.48 | 7.22 | 13.92 | 28.79 | 44.75 | 69.01 | 100.31 |
| Republic of Korea | 0.17 | 0.29 | 0.54 | 1.16 | 2.61 | 5.58 | 9.84 | 15.44 | 23.15 | 34.82 | 54.49 | 87.63 | 138.36 | 195.77 | 247.81 |
| South Sudan | 0.07 | 0.09 | 0.16 | 0.41 | 0.88 | 2.75 | 5.40 | 10.75 | 17.56 | 23.34 | 39.28 | 70.00 | 101.01 | 158.21 | 184.57 |
| Spain | 0.01 | 0.01 | 0.02 | 0.05 | 0.20 | 0.48 | 1.11 | 2.74 | 6.48 | 15.03 | 25.31 | 49.41 | 54.37 | 59.00 | 61.91 |
| Sri Lanka | 0.03 | 0.04 | 0.12 | 0.32 | 0.63 | 1.55 | 3.26 | 5.14 | 8.53 | 17.71 | 33.91 | 64.61 | 94.15 | 133.22 | 169.34 |
| Sudan | 0.05 | 0.05 | 0.10 | 0.25 | 0.59 | 1.51 | 2.96 | 5.94 | 10.16 | 12.96 | 24.24 | 47.72 | 65.02 | 99.64 | 113.33 |
| Suriname | 0.01 | 0.01 | 0.01 | 0.03 | 0.09 | 0.16 | 0.34 | 0.69 | 1.28 | 2.40 | 4.86 | 9.42 | 16.61 | 28.39 | 50.91 |
| Eswatini | 0.01 | 0.01 | 0.02 | 0.03 | 0.10 | 0.24 | 0.54 | 1.33 | 2.62 | 5.04 | 10.20 | 17.82 | 23.95 | 29.02 | 27.96 |
| Sweden | 0.00 | 0.01 | 0.02 | 0.03 | 0.08 | 0.22 | 0.44 | 0.89 | 1.97 | 3.62 | 9.49 | 18.26 | 29.81 | 42.28 | 61.81 |
| Switzerland | 0.28 | 0.76 | 1.72 | 4.40 | 9.64 | 17.35 | 26.66 | 37.51 | 50.34 | 64.36 | 85.13 | 112.93 | 160.69 | 215.75 | 306.80 |
| Syrian Arab Republic | 0.02 | 0.03 | 0.09 | 0.21 | 0.56 | 1.36 | 3.59 | 7.37 | 10.44 | 17.91 | 28.56 | 46.06 | 70.96 | 65.54 | 35.02 |
| Taiwan (Province of China) | 0.11 | 0.23 | 0.43 | 1.05 | 2.74 | 5.89 | 12.35 | 28.12 | 48.24 | 67.49 | 101.58 | 185.05 | 259.76 | 331.77 | 312.03 |
| Tajikistan | 0.02 | 0.04 | 0.10 | 0.21 | 0.51 | 1.30 | 3.34 | 6.90 | 10.43 | 17.81 | 27.24 | 46.35 | 69.44 | 87.75 | 103.00 |
| United Republic of Tanzania | 0.00 | 0.01 | 0.01 | 0.02 | 0.06 | 0.15 | 0.34 | 0.76 | 1.66 | 3.60 | 8.18 | 20.12 | 44.73 | 93.95 | 174.34 |
| Thailand | 0.02 | 0.03 | 0.07 | 0.15 | 0.41 | 1.04 | 2.12 | 4.80 | 8.53 | 15.29 | 23.98 | 42.04 | 55.48 | 67.79 | 107.35 |
| Bahamas | 0.15 | 0.25 | 0.42 | 0.84 | 2.13 | 4.53 | 8.01 | 13.53 | 19.71 | 26.55 | 34.74 | 43.40 | 57.81 | 75.79 | 91.33 |
| Gambia | 0.27 | 0.47 | 0.82 | 1.45 | 2.95 | 5.73 | 10.63 | 16.32 | 23.53 | 30.02 | 45.43 | 70.03 | 96.94 | 119.12 | 110.76 |
| Timor-Leste | 0.00 | 0.01 | 0.02 | 0.03 | 0.10 | 0.18 | 0.36 | 0.81 | 1.47 | 2.96 | 6.75 | 17.32 | 37.92 | 76.30 | 100.94 |
| Togo | 0.00 | 0.01 | 0.01 | 0.01 | 0.05 | 0.13 | 0.30 | 0.62 | 1.12 | 2.46 | 5.95 | 14.31 | 32.37 | 63.46 | 110.42 |
| Tonga | 0.08 | 0.12 | 0.18 | 0.35 | 0.98 | 2.15 | 3.95 | 7.63 | 11.70 | 18.36 | 31.59 | 56.58 | 99.29 | 128.22 | 148.21 |
| Trinidad and Tobago | 0.02 | 0.05 | 0.12 | 0.27 | 0.80 | 1.61 | 2.84 | 5.53 | 9.16 | 15.71 | 31.26 | 56.83 | 100.14 | 150.47 | 151.08 |
| Tunisia | 0.01 | 0.01 | 0.02 | 0.04 | 0.17 | 0.43 | 1.15 | 2.38 | 4.56 | 6.79 | 10.29 | 12.43 | 8.38 | 12.97 | 19.91 |
| Turkey | 0.02 | 0.05 | 0.11 | 0.22 | 0.49 | 1.05 | 2.13 | 4.53 | 7.65 | 13.15 | 26.15 | 48.00 | 55.79 | 81.50 | 110.32 |
| Turkmenistan | 0.01 | 0.01 | 0.03 | 0.09 | 0.25 | 0.62 | 1.42 | 2.79 | 4.72 | 7.43 | 11.52 | 18.33 | 24.61 | 30.89 | 38.86 |
| Uganda | 0.01 | 0.02 | 0.03 | 0.10 | 0.25 | 0.68 | 1.94 | 3.91 | 7.30 | 10.28 | 19.86 | 43.31 | 61.36 | 92.35 | 108.69 |
| Ukraine | 0.27 | 0.53 | 1.01 | 2.00 | 4.49 | 9.48 | 16.13 | 26.10 | 36.43 | 49.46 | 71.40 | 105.19 | 138.47 | 177.97 | 238.16 |
| United Arab Emirates | 0.17 | 0.46 | 1.22 | 3.00 | 6.24 | 13.29 | 22.42 | 35.45 | 54.21 | 70.42 | 89.34 | 131.00 | 192.18 | 271.87 | 396.47 |
| United Kingdom | 0.30 | 0.45 | 0.75 | 1.49 | 4.47 | 9.49 | 23.55 | 46.86 | 56.22 | 83.44 | 109.58 | 173.29 | 209.18 | 282.26 | 344.91 |
| United States of America | 0.02 | 0.03 | 0.05 | 0.12 | 0.30 | 0.70 | 1.57 | 3.43 | 6.54 | 13.07 | 22.97 | 46.11 | 67.79 | 94.98 | 106.89 |
| Uruguay | 0.02 | 0.04 | 0.07 | 0.15 | 0.49 | 1.25 | 2.83 | 6.24 | 13.00 | 23.23 | 44.95 | 79.29 | 131.04 | 168.95 | 183.07 |
| Uzbekistan | 0.03 | 0.04 | 0.07 | 0.15 | 0.46 | 1.06 | 2.64 | 5.06 | 7.10 | 10.55 | 15.81 | 21.42 | 14.12 | 19.35 | 23.29 |
| Vanuatu | 0.17 | 0.40 | 1.01 | 2.63 | 5.72 | 12.38 | 21.37 | 33.40 | 43.71 | 61.55 | 79.15 | 119.46 | 157.85 | 194.08 | 273.96 |
| Venezuela (Bolivarian Republic of) | 0.01 | 0.03 | 0.07 | 0.15 | 0.36 | 0.92 | 2.19 | 4.64 | 7.36 | 13.67 | 23.56 | 43.79 | 69.15 | 92.62 | 102.18 |
| Viet nam | 0.00 | 0.01 | 0.01 | 0.02 | 0.06 | 0.11 | 0.24 | 0.56 | 0.94 | 1.31 | 1.81 | 2.56 | 2.11 | 2.80 | 3.11 |
| United States Virgin Islands | 0.11 | 0.08 | 0.10 | 0.21 | 0.59 | 1.54 | 3.68 | 9.56 | 11.95 | 78.83 | 103.32 | 110.46 | 129.08 | 131.73 | 185.72 |
| Yemen | 0.01 | 0.02 | 0.03 | 0.05 | 0.18 | 0.31 | 0.55 | 1.01 | 1.63 | 2.97 | 6.27 | 14.47 | 29.03 | 57.73 | 108.70 |
| Zambia | 0.01 | 0.01 | 0.02 | 0.05 | 0.14 | 0.36 | 0.91 | 1.84 | 3.10 | 5.63 | 8.90 | 15.91 | 25.77 | 33.43 | 40.73 |
| Zimbabwe | 0.01 | 0.04 | 0.09 | 0.19 | 0.48 | 1.02 | 1.77 | 3.03 | 4.84 | 9.13 | 15.94 | 29.85 | 45.93 | 72.28 | 114.39 |
| Monaco | 0.04 | 0.08 | 0.15 | 0.26 | 0.50 | 0.81 | 2.53 | 5.25 | 6.78 | 16.10 | 26.30 | 53.50 | 80.09 | 154.53 | 377.15 |
| San Marino | 0.01 | 0.02 | 0.05 | 0.10 | 0.26 | 0.66 | 1.61 | 3.59 | 5.54 | 9.46 | 14.25 | 23.23 | 37.96 | 61.78 | 90.46 |
| Saint Kitts and Nevis | 0.02 | 0.02 | 0.03 | 0.07 | 0.22 | 0.64 | 1.75 | 4.09 | 5.80 | 8.29 | 9.85 | 12.02 | 8.35 | 11.60 | 19.94 |
| Cook Islands | 0.02 | 0.04 | 0.13 | 0.33 | 0.85 | 2.03 | 4.81 | 7.90 | 13.92 | 23.22 | 35.34 | 54.65 | 72.34 | 96.86 | 154.16 |
| Nauru | 0.06 | 0.09 | 0.16 | 0.32 | 0.82 | 1.92 | 4.16 | 8.29 | 13.59 | 22.00 | 36.49 | 62.50 | 81.13 | 92.98 | 80.77 |
| Niue | 0.01 | 0.04 | 0.09 | 0.24 | 0.50 | 1.06 | 2.05 | 4.86 | 9.11 | 15.38 | 24.47 | 40.47 | 63.41 | 89.27 | 109.44 |
| Palau | 0.02 | 0.03 | 0.07 | 0.21 | 0.70 | 1.63 | 3.29 | 6.30 | 9.79 | 14.54 | 21.91 | 32.08 | 42.86 | 55.95 | 70.66 |
| Tokelau | 0.03 | 0.05 | 0.14 | 0.26 | 0.63 | 1.55 | 3.95 | 8.65 | 12.00 | 19.57 | 28.87 | 44.83 | 69.36 | 72.05 | 54.60 |
| Tuvalu | 0.03 | 0.06 | 0.11 | 0.33 | 0.91 | 2.32 | 6.36 | 14.05 | 21.77 | 33.83 | 54.34 | 90.49 | 115.45 | 95.68 | 55.02 |

Supplementary table 5:The top three and the bottom three regions of diabetes and kidney diseases due to low physical activity death or DALY

| Measure | sex | Top three countries |  |  | Bottom three countries |  |  |
| --- | --- | --- | --- | --- | --- | --- | --- |
| 2021 ASR (per 100,000 people) |  |  |  |  |  |  |  |
| Age-standardized DALY rate |  |  |  |  |  |  |  |
|  | both | Kiribati(694.76) | Micronesia (Federated States of)(703.44) | Marshall Islands(1010.40) | Ukraine(17.83) | United Republic of Tanzania(20.20) | Belarus(20.58) |
|  | female | Fiji(812.15) | Kiribati(839.46) | Marshall Islands(1415.06) | Ukraine(21.39) | Mongolia(22.32) | Belarus(23.10) |
|  | male | Micronesia (Federated States of)(605.84) | American Samoa(606.79) | Marshall Islands(616.60) | Ukraine(12.46) | United Republic of Tanzania(14.42) | Uzbekistan(16.74) |
| ASDR |  |  |  |  |  |  |  |
|  | both | Kiribati(24.18) | Fiji(25.07) | Marshall Islands(30.46) | Ukraine(0.18) | Belarus(0.36) | Mongolia(0.48) |
|  | female | Kiribati(28.64) | Fiji(29.57) | Marshall Islands(45.64) | Ukraine(0.21) | Belarus(0.36) | Mongolia(0.48) |
|  | male | American Samoa(17.83) | Micronesia (Federated States of)(19.01) | Fiji(20.10) | Ukraine(0.13) | Belarus(0.37) | Singapore(0.46) |
| 1990-2021 increase times |  |  |  |  |  |  |  |
| DALY (cases) |  |  |  |  |  |  |  |
|  | both | Guatemala(9.12) | United Arab Emirates(12.22) | Qatar(12.51) | Netherlands(1.32) | Italy(1.34) | Germany(1.43) |
|  | female | United Arab Emirates(8.41) | Guatemala(9.92) | Qatar(12.91) | Netherlands(1.09) | Italy(1.19) | Germany(1.19) |
|  | male | Jordan(7.93) | Qatar(12.22) | United Arab Emirates(14.95) | Nauru(1.32) | Ethiopia(1.45) | Tokelau(1.53) |
| Deaths (case) |  |  |  |  |  |  |  |
|  | both | Russian Federation(6.66) | Qatar(7.54) | Guatemala(9.40) | United Kingdom(0.86) | Switzerland(0.98) | Belgium(0.99) |
|  | female | Honduras(7.11) | Qatar(8.49) | Guatemala(10.44) | United Kingdom(0.78) | Belgium(0.83) | Switzerland(0.86) |
|  | male | Uzbekistan(7.10) | Guatemala(7.33) | United Arab Emirates(7.47) | United Kingdom(0.98) | Afghanistan(1.07) | Nauru(1.19) |
| EAPC |  |  |  |  |  |  |  |
| DALY |  |  |  |  |  |  |  |
|  | both | Libya(3.02) | Lesotho(3.31) | Mauritius(3.40) | Cyprus(-2.54) | Ethiopia(-1.67) | Rwanda(-1.55) |
|  | female | Uzbekistan(3.17) | Lesotho(3.70) | United Arab Emirates(3.78) | Cyprus(-2.88) | Taiwan (Province of China)(-1.90) | Maldives(-1.89) |
|  | male | Uzbekistan(2.95) | Bosnia and Herzegovina(3.17) | Mauritius(3.93) | Cyprus(-2.07) | Ethiopia(-1.43) | Rwanda(-1.35) |
| Deaths |  |  |  |  |  |  |  |
|  | both | Georgia(3.58) | Mauritius(4.02) | Russian Federation(4.40) | Singapore(-3.89) | Cyprus(-3.40) | Switzerland(-2.69) |
|  | female | Lesotho(3.78) | Russian Federation(4.35) | United Arab Emirates(5.94) | Singapore(-4.10) | Cyprus(-3.80) | Guam(-3.36) |
|  | male | Czechia(3.88) | Russian Federation(3.94) | Mauritius(4.43) | Singapore(-3.54) | Cyprus(-2.91) | United Kingdom(-2.17) |

Supplementary table6.The top three and the bottom three countries of diabetes and kidney diseases due to low physical activity death or DALY

| Measure | sex | Top three countries |  |  | Bottom three countries |  |  |
| --- | --- | --- | --- | --- | --- | --- | --- |
| 2021 ASR (per 100,000 people) |  |  |  |  |  |  |  |
| Age-standardized DALY rate |  |  |  |  |  |  |  |
|  | both | North Africa and Middle East(159.48) | Southern sub-Saharan Africa(255.66) | Oceania(285.33) | Eastern Europe(37.83) | Central Asia(42.13) | East Asia(47.47) |
|  | female | Caribbean(201.67) | Southern sub-Saharan Africa(282.96) | Oceania(370.33) | Eastern Europe(44.39) | Western Europe(49.74) | East Asia(53.84) |
|  | male | Central sub-Saharan Africa(131.34) | Oceania(205.84) | Southern sub-Saharan Africa(213.71) | Eastern Europe(26.29) | Central Asia(26.69) | Southern Latin America(36.98) |
| ASDR |  |  |  |  |  |  |  |
|  | both | Central sub-Saharan Africa(4.93) | Oceania(9.41) | Southern sub-Saharan Africa(10.56) | High-income Asia Pacific(0.80) | Central Asia(1.04) | Eastern Europe(1.18) |
|  | female | North Africa and Middle East(5.74) | Southern sub-Saharan Africa(11.55) | Oceania(11.76) | High-income Asia Pacific(0.72) | Western Europe(1.24) | Central Asia(1.26) |
|  | male | Central sub-Saharan Africa(5.83) | Oceania(7.19) | Southern sub-Saharan Africa(8.80) | Eastern Europe(0.73) | Central Asia(0.74) | High-income Asia Pacific(0.89) |
| 1990-2021 increase times |  |  |  |  |  |  |  |
| DALY (cases) |  |  |  |  |  |  |  |
|  | both | South Asia(3.75) | Andean Latin America(4.03) | North Africa and Middle East(4.40) | Western Europe(1.61) | Central Europe(1.99) | Southern Latin America(2.00) |
|  | female | Andean Latin America(4.03) | South Asia(4.19) | North Africa and Middle East(4.32) | Western Europe(1.41) | Central Europe(1.89) | Southern Latin America(2.08) |
|  | male | Southeast Asia(3.98) | Andean Latin America(4.03) | North Africa and Middle East(4.54) | Southern Latin America(1.87) | Western Europe(2.01) | Eastern Sub-Saharan Africa(2.11) |
| Deaths (case) |  |  |  |  |  |  |  |
|  | both | Southern sub-Saharan Africa(3.65) | South Asia(3.93) | Eastern Europe(5.41) | Western Europe(1.29) | Southern Latin America(1.62) | Caribbean(1.91) |
|  | female | Southern sub-Saharan Africa(3.83) | South Asia(4.40) | Eastern Europe(5.39) | Western Europe(1.14) | Southern Latin America(1.66) | High-income North America(1.69) |
|  | male | East Asia(3.59) | Central Latin America(3.89) | Eastern Europe(5.51) | Southern Latin America(1.55) | Western Europe(1.67) | Eastern Sub-Saharan Africa(1.97) |
| EAPC |  |  |  |  |  |  |  |
| DALY |  |  |  |  |  |  |  |
|  | both | Central Asia(1.82) | Southern sub-Saharan Africa(2.00) | Eastern Europe(2.43) | Eastern Sub-Saharan Africa(-0.25) | Western Europe(-0.10) | Central sub-Saharan Africa(0.03) |
|  | female | Central Asia(1.92) | Southern sub-Saharan Africa(2.11) | Eastern Europe(2.38) | Eastern Sub-Saharan Africa(-0.33) | Western Europe(-0.24) | Tropical Latin America(-0.24) |
|  | male | Central Asia(1.76) | High-income North America(1.79) | Eastern Europe(2.48) | Central sub-Saharan Africa(-0.23) | Eastern Sub-Saharan Africa(-0.19) | Southern Latin America(-0.11) |
| Deaths |  |  |  |  |  |  |  |
|  | both | Central Asia(1.77) | Southern sub-Saharan Africa(2.14) | Eastern Europe(3.79) | High-income Asia Pacific(-1.90) | Western Europe(-1.45) | Southern Latin America(-0.81) |
|  | female | Central Asia(1.73) | Southern sub-Saharan Africa(2.26) | Eastern Europe(3.71) | High-income Asia Pacific(-2.33) | Western Europe(-1.77) | Caribbean(-0.88) |
|  | male | Southern sub-Saharan Africa(1.83) | Central Asia(2.08) | Eastern Europe(3.61) | High-income Asia Pacific(-1.45) | Western Europe(-0.88) | Australasia(-0.76) |
